# Covariation in levels of nucleotide diversity in homologous regions of the avian genome long after completion of lineage sorting

**DOI:** 10.1098/rspb.2016.2756

**Published:** 2017-02-22

**Authors:** Ludovic Dutoit, Nagarjun Vijay, Carina F. Mugal, Christen M. Bossu, Reto Burri, Jochen Wolf, Hans Ellegren

**Affiliations:** 1 Department of Evolutionary Biology, Evolutionary Biology Centre, Uppsala University, Norbyvägen 18D, 752 36 Uppsala, Sweden; 2 Division of Evolutionary Biology, Faculty of Biology II, Ludwig-Maximilians-Universität München, Grosshaderner Strasse 2, 82152 Planegg-Martinsried, Germany

**Keywords:** nucleotide diversity, linked selection, recombination rate, birds

## Abstract

Closely related species may show similar levels of genetic diversity in homologous regions of the genome owing to shared ancestral variation still segregating in the extant species. However, after completion of lineage sorting, such covariation is not necessarily expected. On the other hand, if the processes that govern genetic diversity are conserved, diversity may potentially covary even among distantly related species. We mapped regions of conserved synteny between the genomes of two divergent bird species—collared flycatcher and hooded crow—and identified more than 600 Mb of homologous regions (66% of the genome). From analyses of whole-genome resequencing data in large population samples of both species we found nucleotide diversity in 200 kb windows to be well correlated (Spearman's *ρ* = 0.407). The correlation remained highly similar after excluding coding sequences. To explain this covariation, we suggest that a stable avian karyotype and a conserved landscape of recombination rate variation render the diversity-reducing effects of linked selection similar in divergent bird lineages. Principal component regression analysis of several potential explanatory variables driving heterogeneity in flycatcher diversity levels revealed the strongest effects from recombination rate variation and density of coding sequence targets for selection, consistent with linked selection. It is also possible that a stable karyotype is associated with a conserved genomic mutation environment contributing to covariation in diversity levels between lineages. Our observations imply that genetic diversity is to some extent predictable.

## 1. Introduction

Understanding the evolutionary mechanisms governing the extent of genetic diversity (e.g. degree of polymorphism, heterozygosity or nucleotide diversity) within and between species is important to evolutionary biology in several respects [1]. For example, genomic scans for adaptively evolving loci require distinguishing signals of selection from other factors influencing genetic diversity [2]. Studies of population differentiation and speciation genetics are based on patterns of diversity and divergence, and the relationship between these parameters, such as the estimation of *F*_ST_ [3]. Moreover, genetic diversity is essential to conservation biology, including questions related to inbreeding and to the long-term adaptability of endangered species [4].

Genetic diversity is not a constant entity across the genome, but is known to vary considerably among chromosomes, genomic regions and functional categories of sequences [5–8]. As long as ancestral variation still segregates in diverging lineages (i.e. lineage sorting is not completed), levels of genetic diversity in homologous regions of diverging genomes might be correlated. However, once ancestral variation is no longer shared owing to fixation of previously segregating variants, there is no reason *a priori* to expect diversity levels of homologous regions to covary between species. Yet, if the patterns and processes that govern diversity levels within genomes are conserved over evolutionary time scales, then diversity levels might be correlated. One notable situation concerns orthologous sites and sequences evolving under purifying selection in parallel lineages—such sequences are expected to show reduced nucleotide diversity in both lineages. Genes and other functional elements common to species are examples of sequences that are likely to show similarly low levels of diversity in different lineages. On the other end of the diversity spectrum, some sites and sequences could show increased diversity in parallel lineages owing to balancing selection [9]. However, trans-species polymorphisms would have to represent a high proportion of all polymorphisms to cause covariation of genetic diversity between species.

Even for neutrally evolving sequences genetic diversity of homologous regions of diverging genomes could potentially covary. One possible reason for this would be the local mutation rate (*μ*; cf. *θ* = 4 *N*_e_*μ*), which varies across the genome [10,11]. Another factor would be the degree of linked selection [12], which reduces diversity levels through background selection [13] or selective sweeps [14,15]. If the patterns of mutation rate variation and/or the intensity of linked selection are conserved among species, then this might result in covariation in neutral diversity levels.

Compared with other vertebrates, avian genomes are recognized to have unusually stable karyotypes [16] with 2*n* (diploid number of chromosomes) = 76–80 in the majority of species [17]. Essentially, all species characteristically show a limited number of large chromosomes (macrochromosomes) and a large number of very small chromosomes (microchromosomes) [18]. If karyotypic stability is associated with conservation of evolutionary processes governing genetic diversity (see further below), we hypothesized that covariation in regional levels of genetic diversity might be detectable in diverging lineages of birds. Here, we test this hypothesis using whole-genome resequencing data from population samples of two distantly related passerine species, the collared flycatcher (*Ficedula albicollis*) and the hooded crow (*Corvus (corone) cornix*), both species with a karyotype of 2*n* = 80 [19]; for flycatcher karyotype information is from *F. parva* and *F. mugimaki* [20]. Genome assemblies with high sequence continuity are available for both species [21–23], and both genomes have been functionally annotated [24]. Phylogenetic analyses place the separation between the two lineages in the order of 25 million years ago (Ma) [25–27], which should be seen as a minimum time of divergence, because fossils put the early core corvids at 20–25 Ma [27]. Crow–flycatcher divergence thus corresponds to at least 4–12 million generations assuming a generation time of 6 years for hooded crows [28] and 2 years for flycatchers [29]. With an estimated long-term *N*_e_ of 200 000 for both species [30–32], this yields a range of 20–60 *N*_e_ generations as time to the most common ancestor. Because this is clearly beyond the expected time for complete lineage sorting (9–12 *N*_e_ generations [33]), the two species are thus not expected to share neutral ancestral polymorphism.

## 2. Material and methods

### (a) Identification of genomic regions of conserved synteny

We identified regions that shared the same ancestral localization between the hooded crow (assembly v. 2.7) and collared flycatcher (fAlb15). We referred to these as regions of conserved synteny, and did not proceed to a base-to-base alignment as the synteny approach is much simpler and sufficient for the question addressed in the study.

First, we obtained pairwise alignments using LastZ v. 1.02.00 [34] and repeat-masked genome assemblies. We then used the UCSC Genome Browser toolset [35] and the JCVI library [36] in order to obtain a chain file, an alignment that allows gaps in both sequences at the same positions. This chain file was then hierarchically reorganized to be used as a lift-over chain (i.e. the conversion file to translate genomic coordinates from one species to the other according to conserved synteny between the two genomes).

We used liftOver, a program from the UCSC Kent source utilities package [35], to convert regions from one genome into the other. We used non-overlapping 200 kb windows along autosomes of the flycatcher genome as reference and retrieved conserved syntenic, collinear sequences in the crow. Windows were retained for further analyses if more than 80% of the bases in collared flycatcher could be remapped to one window in the crow. We excluded alignments less than 180 kb or greater than 220 kb as large size discrepancies may not only indicate repeat region reductions/expansions or small rearrangements, but could also be a sign of spurious alignments.

### (b) Population re-sequencing data

We extracted re-sequencing data from 30 hooded crows sampled in two populations (Poland and Sweden) and 30 collared flycatchers also sampled in two populations (Czech Republic and Italy). Procedures for read mapping and variant calling are described in the original reports of polymorphism data [23,37] and were largely consistent between the two datasets. Briefly, raw reads were mapped to the respective reference genomes using BWA [38] v. 0.7.4 followed by local realignment using GATK [39,40] (v. 2.3.6 for hooded crows and v. 2.4.9 for collared flycatchers) and removal of duplicates using PICARD (http://picard.sourceforge.net), v. 1.46 for crows and v. 1.77 for flycatchers. Variant discovery was performed on a per-population basis to account for population structure. For both species, base quality score recalibration (BQSR) was conducted using an iterative approach. BQSR normally requires true variants to be excluded from error model building. In the absence of prior knowledge of segregating variants, a first round of variant calling was conducted using three different algorithms: GATK UnifiedGenotyper [39], samtools (v. 0.1.18 for both species) [41] and FreeBayes (v. 0.9.8 for crows and v. 0.9.6 for flycatchers) [42]. The single nucleotide polymorphisms (SNPs) detected by the three methods were used as true variants for the BQSR. A first round of BQSR was then run using GATK UnifiedGenotyper exclusively. In the crow, a second round of recalibration was performed but 99.5% of the variants were shared with the first round. On that basis, the calibration was considered to have achieved high consistency and the first round of recalibration was used. For the flycatcher, a second round of BQSR was performed on one population and, because the results were more than 99% identical to the first round, the second round was ignored also in this case. Subsequent variant quality score recalibration was performed with GATK to assign a probability of each SNP being a true variant based on a set of verified variants. Variable sites across populations within species were finally combined and populations then regenotyped individually using GATK UnifiedGenotyper. As a final conservative filtering step specific to this study, we considered only sites where all individuals within a given population had coverage of at least four.

Nucleotide diversity per 200 kb window was computed on a per-population basis using the Python package pyVCF 0.4.0 and biopython v. 1.68 [43]. Averages were then calculated for each window and species using the mean of both populations; note that per-window diversity levels were strongly correlated between the two collared flycatcher populations (Pearson's *r* = 0.99) and the two hooded crow populations (*r* = 0.96). Windows with fewer than 10 000 sites remaining after coverage based filtering were excluded as was an outlier window in which hooded crow diversity level was far higher (0.0042) than in all other windows (range = 0.0002–0.0023).

Although different software versions were used for GATK, PICARD and FreeBayes in the analyses of the two species, we believe that this has little impact on the results. For example, there were no major changes between v. 2.3.6 and 2.4.9 of GATK. UnifiedGenotyper and FreeBayes only help calibrating GATK.

### (c) Data analysis

Collared flycatcher gene annotations were retrieved from ENSEMBL genebuild for release 1.4 of the collared flycatcher genome assembly. Coordinates were then translated to the fAlb15 assembly version. Gene annotations for the hooded crow were obtained from release 100 of GenBank. These annotations were used to estimate coding sequence density. Lineage-specific synonymous substitution rate, *d*_S_, was obtained for the collared flycatcher and was based on data from three-species coding sequence alignments with chicken (*Gallus gallus*) and zebra finch (*Taenopygia guttata*) [44]. After excluding genes with *d*_S_ = 0 and more than 2 [44], we calculated the average *d*_S_ per 200 kb window, weighted by gene length. If the average *d*_S_ for a window was above 0.3, *d*_S_ was set as missing data. We further obtained data on recombination rate per 200 kb window in the collared flycatcher [22]. These data were originally generated by linkage analysis from the genotyping of a 50 K SNP chip on a large (more than 800 individuals) flycatcher pedigree. Finally, we extracted intergenic GC content as well as the repeat density for each window. We transformed certain candidate explanatory variables to reduce the skewness in their distribution: coding sequence density and *d*_S_ were transformed by the square root, and recombination rate was log-transformed to base 10 after adding a constant 1.

We performed a multiple linear regression of collared flycatcher nucleotide diversity (*y*) against recombination rate (*x*_1_), coding sequence density (*x*_2_), dS (*x*_3_), GC content (*x*_4_) and repeat density (*x*_5_). No interactions were incorporated to avoid over-parametrization:
2.1
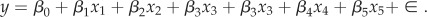


The underlying assumptions of such linear regression analysis are the lack of heteroscedasticity, multivariate normality and linear relationships between the explanatory variables and the response variable, as well as no collinearity between explanatory variables. In particular, the assumption of no collinearity between explanatory variables appeared problematic. A matrix of pairwise correlation coefficients as well as a correlation tree based on a nested agglomerative method described in [45] is provided as electronic supplementary material.

To handle the problem of collinearity we performed a principal component (PC) regression (PCR), a method derived from principal component analysis (PCA). PCs are calculated using the explanatory variables only. The PCs are then used as predictors for variation in the response variable. It is an efficient way to get around the problem of collinearity between explanatory variables [46].

To specifically investigate the effect of coding sequence density on the extent of linked selection in the flycatcher genome, we regressed nucleotide diversity against recombination rate within gene-rich and gene-poor regions using respectively the highest and the lowest 10% of windows from the distribution of coding sequence density. Because coding sequence density is correlated with GC content, we further regressed nucleotide diversity against recombination rate within GC-rich and GC-poor regions using respectively the highest and the lowest 10% of windows from the distribution of GC content.

## 3. Results

### (a) Levels of genetic diversity

The hooded crow and the collared flycatcher show moderate to moderately high levels of nucleotide diversity with genome-wide averages of *π* = 0.0039 (collared flycatcher) and 0.0011 (hooded crow) in the studied populations. Just as observed in many other species, diversity levels vary across the two genomes with *π* estimates in the 200 kb windows investigated here in the range of 0.0018–0.0060 for collared flycatcher and 0.0002–0.0023 for hooded crows. The chosen window size was considered to be a reasonable trade-off between capturing fine-scale variation in nucleotide diversity and limiting the noise in the estimation of genomic parameters [22]. We retrieved more than 600 Mb (collared flycatcher: 652 Mb; hooded crow: 637 Mb) of conserved synteny between the two species, distributed across all chromosomes. This corresponds to 66% of the flycatcher autosomal assembly (989 Mb) that was used as reference and has scaffolds anchored, ordered and oriented along chromosomes.

As indicated above, a divergence time of at least 25 million years probably means that lineage sorting is completed between the analysed lineages. To test this, we stringently investigated the overlap of segregating sites in the two species by considering the incidence of sites variable in at least one population of flycatchers and one population of crows. Out of 253 303 variable sites in flycatchers, which could be aligned between the two species, only 464 (0.2%) were also variable in crows, confirming that lineage sorting is essentially complete between the two species. This is especially so when considering that any species comparison is bound to include sites polymorphic in both species owing to independently derived mutations, in particular at highly mutable CpG sites.

### (b) Correlation of diversity levels between species

**Figure 1.**
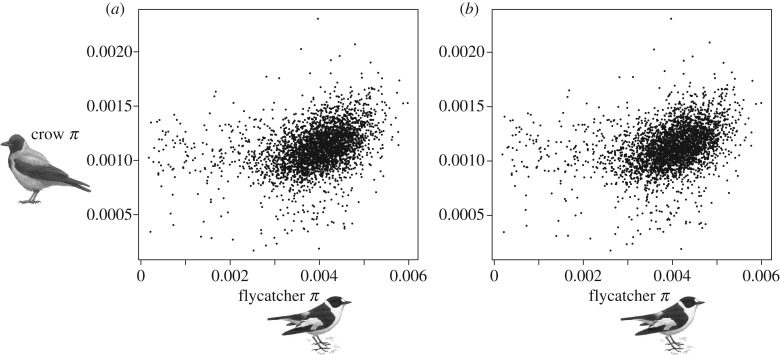
Correlation (Spearman's *ρ* = 0.407) between collared flycatcher and hooded crow nucleotide diversity in 200 kb windows of conserved synteny (*n* = 3259) spread across the genome. (*a*) All sequences, (*b*) excluding coding sequences (Spearman's *ρ* = 0.402).

Levels of genetic diversity in regions of conserved synteny (200 kb windows; *n* = 3259) of the collared flycatcher and hooded crow genomes were correlated (Spearman's *ρ* = 0.407; *p* < 0.0001; figure 1*a*). Because we analysed more than 60% of the two genomes the investigated regions should provide a representative picture of evolutionary processes affecting genetic diversity in these species. Nevertheless, to exclude biased sampling of genomic regions, we compared the distribution of nucleotide diversity, coding sequence density, recombination rate, *d*_S_, GC content and repeat density between investigated regions and the whole genome (electronic supplementary material, figure S1). The only difference found was a lower repeat density of investigated regions compared with the whole genome, which is probably owing to an expected inverse relationship between repeat density and the ability to identify syntenic regions.

### (c) How can (co)variation in diversity levels be explained?

Previous studies have indicated that genetic diversity within avian genomes varies in relation to chromosome size [47–49]. Together with a stable karyotype, this could potentially lead to an overall correlation between diversity levels in regions of conserved synteny of two species. However, when we regressed hooded crow diversity against collared flycatcher diversity and chromosome length, the effect of chromosome size was not significant (table 1). Another possible factor that could explain a correlation in diversity levels between species is the density of coding sequences (collared flycatcher: mean 0.02 per site, range 0.00–0.15; hooded crow: mean 0.02, range 0.00–0.15) if this density covaries between species and has a large direct effect on 200 kb window-based diversity estimates (given that diversity levels in coding sequences are much lower than in intergenic DNA and introns). When coding sequences were masked, however, the strength of correlation between diversity levels in the two species remained essentially unaltered (*ρ* = 0.402; *p* < 0.0001; figure 1*b*). This therefore suggests that there is some mechanism that affects regional diversity levels in similar ways in syntenic regions of the two genomes.

**Table 1.** Effect of chromosome length as a covariate of collared flycatcher nucleotide diversity in explaining hooded crow nucleotide diversity. A significant regression equation was found (*F*_2,3256_ = 203.3, *p*-value: < 2.2 × 10^−16^), with *R*^2^ = 0.111.

	*t*-value	*p*-value
flycatcher diversity	18.93	<2 × 10^−16^
chromosome length	−1.34	0.162

In order to identify this mechanism, we investigated the driving forces of variation in diversity levels across the avian genome, and given that recombination rate is likely to be a crucial parameter, we focused on the collared flycatcher because pedigree-based recombination rate data are available for this species. We performed a multiple linear regression analysis and, in addition to recombination rate, incorporated coding sequence density as a proxy for the density of targets for selection, *d*_S_ as a proxy for the local mutation rate, repeat density and GC content; data for all five explanatory variables were available for 2485 out of the windows used in the flycatcher-crow comparison. All parameters had a significant effect on collared flycatcher genetic diversity and in total explained 28.2% of the variation in genomic diversity (table 2). GC content had the strongest effect (*t*-value = −26.8) followed by recombination rate (*t*-value = 16.0) and *d*_S_ (*t*-value = 6.8).

**Table 2.** Factors explaining collared flycatcher genetic diversity. A significant regression equation was found (*F*_5,2479_ = 196.1, *p*-value: < 2.2 × 10^−16^), with *R*^2^ = 0.283. We analysed a full model including recombination rate coding sequence density, GC content, synonymous substitution rate and repeat density.

	*t*-value	*p*-value
recombination rate	16.047	<2 × 10^−16^
coding sequence density	−3.841	1.3 × 10^−4^
GC content	−26.772	<2 × 10^−16^
synonymous substitution rate	6.755	1.8 × 10^−11^
repeat density	4.063	5.0 × 10^−5^

**Figure 2.**
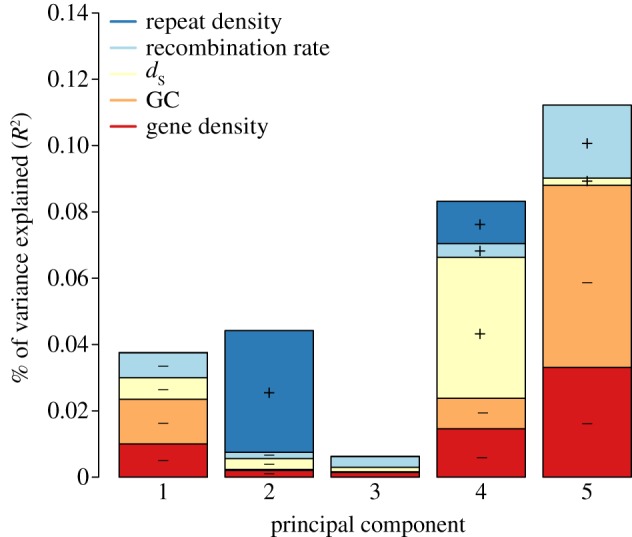
Amount of flycatcher nucleotide diversity explained by different components according to the principal component regression. The analysed explanatory variables are colour-coded according to the key. Plus or minus sign indicates the direction of correlation for individual variables.

However, the results of the multiple linear regression need to be interpreted with caution owing to collinearity of the explanatory variables (electronic supplementary material, table S1 and figure S2). In particular, the correlation between GC content and coding sequence density violates the assumption of independence between explanatory variables (Pearson's *r* = 0.278), and thus the respective effects of the two explanatory variables cannot be distinguished and interpreted separately. We therefore performed PCR (figure 2; electronic supplementary material, table S2) to handle the collinearity problem and treat explanatory variables as compounds of their collinearity. This clearly showed that GC content and coding sequence density were tightly linked together, and could therefore not be interpreted separately. PC5, which was mainly governed by GC content, coding sequence density and recombination rate, explained the most of the variance (11.22%). The positive relationship between diversity and recombination rate and the negative relationship between diversity and coding sequence density support a role of linked selection, because diversity should be most reduced in regions of low recombination and high density of target of selection in a linked selection scenario. The negative relationship between diversity and GC content is in agreement with the observed covariation between coding sequence density and GC content. PC4 explained 8.31% of the variance and was dominated by positive relationships between diversity and *d*_S_, and diversity and repeat density. This would indicate a role of mutation rate variation in explaining variation in diversity levels. PC2 explained 4.42% of the variation and was mostly linked to repeat density. Axes 1 and 3 explained less variance and were difficult to interpret.

To further investigate a role of linked selection on diversity levels we compared genomic regions corresponding to the 10% windows with the lowest coding sequence densities (or GC content) and the 10% windows with the highest coding sequence densities (or GC content). Specifically, we regressed diversity against recombination rate in these two categories of genomic regions to investigate whether the strength of the correlation depends on coding sequence density (or GC content). Recombination rate had a significant effect on diversity in regions with the highest coding sequence density (*F*_1,247_ = 22.55, *p* < 0.0001; *R*^2^ = 0.08), but not in regions with the lowest coding sequence density (*F*_1,247_ = 0.04, *p* = 0.85). Similarly, recombination rate had a significant effect on diversity in regions with the highest GC content (*F*_1,247_ = 70.44, *p* < 0.0001; *R*^2^ = 14.47 = 0.22), but was reduced in regions with the lowest GC content (*F*_1,247_ = 14.47, *p* < 0.0001; *R*^2^ = 0.06). This supports a role of linked selection in governing diversity levels.

## 4. Discussion

Linked selection affects diversity levels across the genome [1,12]. As predicted by theory, the influence of linked selection has been shown to be affected by several factors, including recombination rate [7,50] and density of targets of selection [6,51]. The extent to which these factors are conserved across species is probably related to general aspects of genome evolution and architecture such as karyotype stability, rate of chromosomal rearrangements and the evolution of base composition. The rate of interchromosomal [16] as well as intrachromosomal rearrangement is low in birds [22,52]. For example, collared flycatcher and zebra finch chromosomes are entirely syntenic and largely collinear [22]. It has been suggested that the stability in genome architecture is associated with stability in genomic features such as recombination rate variation [53]. Indeed, comparisons of broad-scale [22,54] as well as fine-scale (i.e. recombination hot-spots [55]) recombination rates in different avian species indicate that the genomic landscape of recombination rate variation in birds is well conserved. In comparing homologous 1 Mb windows of two distantly related bird species—zebra finch and chicken (*G. gallus*)—Backström *et al.* [54] found that recombination rates were correlated with Spearman's *ρ* = 0.50. Such conservation would promote the build-up over time of correlations between recombination rate and different genomic parameters; a strong correlation observed between recombination rate and base composition represents one such example [56].

We suggest that karyotypic stability and a conserved genomic landscape of recombination rate variation, via the effect they assert on the extent of diversity-reducing linked selection, can at least in part explain the correlation in regional levels of neutral genetic diversity between the collared flycatcher and hooded crow genomes. In the absence of pedigree-based recombination rate data for hooded crow, we cannot formally demonstrate conservation of the recombination landscape compared with collared flycatcher. Crows are difficult to breed in captivity, marked populations cannot easily be followed for many generations in the wild and brood sizes are small, factors that hinder gathering large pedigrees for linkage mapping and associated recombination rate estimation. Moreover, using population-scaled recombination rate data based on the extent of linkage disequilibrium [55] for comparing recombination rate profiles in the two species would be less suitable because linkage disequilibrium is the result of the combined effect of selection and recombination.

In the regression analysis of flycatcher diversity data the PC explaining most of the variance was recombination rate together with density of coding sequence, consistent with linked selection. The second strongest PC included mainly *d*_S_ and repeat density. With *d*_S_ considered a proxy for the neutral mutation rate and with some evidence for a link between open chromatin, mutation rate and the abundance of transposable elements [11], this would indicate that mutation rate variation contributes to regional variation in genetic diversity. Although theoretically expected (given *θ* = 4 *N*_e_*μ*), there is mixed evidence from empirical studies of a relationship between diversity and mutation rate, possibly because covariation of several genomic variables blurs potential effects of mutation rate variation on diversity. Nevertheless, a stable avian karyotype could allow for a stable genomic environment, leading not only to a stable recombination landscape, but also to a conserved landscape of mutation rate and chromatin structure. Further work should be devoted to analyses of the relationship between mutation and diversity. In the long term, direct estimates (in contrast to indirect estimates obtained from diversity or divergence data) of local mutation rates from pedigrees or mutation accumulation lines are likely to become available and will be quite informative in this respect.

In summary, together with similar results obtained in a comparison of *Drosophila melanogaster* and *D. simulans* [57], our study is one of the first to demonstrate a genome-wide correlation in regional levels of genetic diversity in two lineages long after sorting of ancestral variation. This covariation is seen despite that very different selection pressures (e.g. on life history, ecology, morphology and behaviour) are likely to have operated in the two investigated avian lineages for millions of years. We suggest that the correlation can be explained by a similar genomic architecture of factors governing diversity levels through linked selection, namely karyotypic stability and a conserved recombination rate landscape. More generally, karyotype stability may imply a conserved genomic environment, such that conservation in other factors such as mutation rate variation reinforces the correlation. Our observations imply that genetic diversity is to some extent predictable.

## Supplementary Material

Supplementary tables 1-2 and supplementary figures 1-2

## Supplementary Material

Supplementary Table 3

## Data accessibility

Accession numbers to all sequence data analysed are provided in the original reports of crow and flycatcher polymorphism data. Genomic and population genetic data used in the analyses are provided in electronic supplementary material, table S3.
