# Supplementary material for: Covariation in levels of nucleotide diversity in homologous regions of the avian genome long after completion of lineage sorting
Source: Proc Biol Sci. 2017 Feb 22;284(1849):20162756. doi: 10.1098/rspb.2016.2756 (PMC5326536; doi:10.1098/rspb.2016.2756)
Supplement: Supplementary tables 1-2 and supplementary figures 1-2 [file rspb20162756supp1.docx]

**Table S1.** Pairwise Pearson’s correlation coefficients between genomic features measured for 200 kb windows in the collared flycatchers genome. The values in italic are the non-significant correlations.

|  | dS | Recombimaton rate | Coding sequence density | % GC |
| --- | --- | --- | --- | --- |
| Recombination rate | 0.116 |  |  |  |
| Coding sequence density | 0.165 | 0.067 |  |  |
| % GC | 0.133 | 0.248 | 0.278 |  |
| % Repeats | *0.032* | *0.009* | -0.046 | *-0.033* |

**Table S2**. Percentage of diversity explained by five possible explanatory variables according to PCR analysis of chicken diversity level in 200kb windows. The percentage is listed for each PC separately, as well as for the total amount of variance explained.

|  | PC 1 | PC 2 | PC 3 | PC 4 | PC 5 | Total |
| --- | --- | --- | --- | --- | --- | --- |
| Coding sequence density | 1.00 | 0.20 | 0.14 | 1.46 | 3.31 | 6.11 |
| % GC | 1.35 | 0.03 | 0.02 | 0.92 | 5.49 | 7.81 |
| dS | 0.65 | 0.33 | 0.14 | 4.25 | 0.22 | 5.59 |
| Recombination rate | 0.75 | 0.19 | 0.32 | 0.41 | 2.20 | 3.87 |
| % Repeats | 0.01 | 3.67 | 0.01 | 1.3 | 0.00 | 4.97 |
| Total variance explained | 3.77 | 4.42 | 0.62 | 8.31 | 11.22 | 28.34 |

**Supplementary Figure Legends**

**Figure S1**. Distribution of (a) nucleotide diversity, (b) recombination rate, (c) gene density, (d) GC content and (e) repeat density in the sampled 652 Mb (black line) of the collared flycatcher genome compared to in the complete 989 Mb autosomal genome (red line).

**Figure S2.** Visual representation of Pearson’s pairwise correlations between genomic features measured for 200 kb windows in the collared flycatchers genome using AGNES, an agglomerative nesting hierarchical clustering described in Kaufman and Rousseeuw (1990).

**Figure S1**

**Figure S2**
