# Supplementary material for: Covariation in levels of nucleotide diversity in homologous regions of the avian genome long after completion of lineage sorting
Source: Proc Biol Sci. 2017 Feb 22;284(1849):20162756. doi: 10.1098/rspb.2016.2756 (PMC5326536; doi:10.1098/rspb.2016.2756)
Supplement: Supplementary Table 3 [file rspb20162756supp2.docx]

FLYscaf FLYstart FLYend FLYrecrate CROscaf CROstart CROend FLYmean_pi CROmean_pi FLYmean_pi_Coding_sequence_masked CROmean_pi_Coding_sequence_masked FLYintergenicgc CROintergenicgc FLYrep CROrep FLYcoding_sequence_density CROcoding_sequence_density FLYdS FLYchrom FLYchromLengt(bp)

N00200 200000 400000 4.188231 scaffold_2 43379933 43563278 0.00402933545203 0.00128213639277 0.00414779148945 0.00134668943639 0.426447275621 0.42675633918 0.12339 0.0625487468979 0.05186 0.070320979574 0.0855 Chr1 122177661

N00244 0 200000 2.862536 scaffold_2 42994194 43184810 0.00393730775154 0.00129883687422 0.00403087288689 0.00132341284354 0.436130590479 0.438106897085 0.130555 0.0665369119067 0.027745 0.0290059596256 0.0496666666667 Chr1 122177661

N00257 0 200000 2.958809 scaffold_2 42377290 42561062 0.00389606260995 0.0012844887026 0.0039226624961 0.00129342968703 0.421250584898 0.422153993427 0.11977 0.0601070892192 0.008135 0.00901660753542 0.0335 Chr1 122177661

N00257 200000 400000 1.11336 scaffold_2 42183671 42377290 0.00428508189014 0.00139969783352 0.00441509859201 0.00146057760985 0.404062522253 0.410860922553 0.08577 0.0728182668023 0.042865 0.0484559883069 0.08575 Chr1 122177661

N00257 400000 600000 3.343149 scaffold_2 41992385 42183671 0.0036996241386 0.00118387317815 0.0036996241386 0.00118387317815 0.410765591137 0.417780335257 0.07025 0.0401545330029 0 0 NA Chr1 122177661

N00083 0 200000 1.607386 scaffold_2 41783078 41965558 0.00353405331577 0.000917599135905 0.00354682744771 0.000922272020331 0.432904621989 0.439422128575 0.09829 0.0506247259974 0.005605 0.0103518193775 0.286 Chr1 122177661

N00083 200000 400000 1.645871 scaffold_2 41589998 41783078 0.00378397605886 0.00111694954812 0.00387063136131 0.00113956582523 0.381143582044 0.381837553556 0.09663 0.0732546094883 0.02094 0.0205303501139 0.08 Chr1 122177661

N00083 600000 800000 1.805436 scaffold_2 41218251 41404058 0.00454223825098 0.00112137186005 0.00459796852469 0.00113656233257 0.422781971206 0.420592064169 0.127755 0.0740391912038 0.01573 0.0150801638259 0.105 Chr1 122177661

N00083 800000 1000000 3.629098 scaffold_2 41023333 41218251 0.00439361258264 0.00109860101064 0.00439361258264 0.00109860101064 0.417454797942 0.421115177228 0.09829 0.0797053119773 0 0 NA Chr1 122177661

N00083 1000000 1200000 1.816143 scaffold_2 40831350 41023327 0.00423595368684 0.00125958131358 0.00423595368684 0.00125958131358 0.404689415435 0.407873799412 0.123865 0.0870260499956 0 0 0.029 Chr1 122177661

N00083 1200000 1400000 1.15592 scaffold_2 40642024 40831350 0.00423123715756 0.00128466737636 0.00434572448657 0.00132267052551 0.396213330847 0.400655523505 0.05704 0.0436707055555 0.039255 0.0435756314505 0.113 Chr1 122177661

N00083 1400000 1600000 0.9638132 scaffold_2 40454519 40642024 0.00366045111154 0.00121285263891 0.00380515980203 0.00126669784055 0.397953310269 0.398666273237 0.105275 0.0613636969681 0.041515 0.044974800672 0.0415 Chr1 122177661

N00083 2000000 2200000 5.339146 scaffold_2 39912421 40094480 0.00392027614429 0.00137819383152 0.00392027614429 0.00137819383152 0.471592818813 0.476692313404 0.124625 0.0480613427515 0 0 NA Chr1 122177661

N00083 2200000 2400000 2.320413 scaffold_2 39729589 39912421 0.00402342400813 0.00123846276096 0.00403699642532 0.00124162238362 0.453004378638 0.462213551623 0.10126 0.0494716461013 0.00621 0.00369191388816 0.059 Chr1 122177661

N00083 2600000 2800000 0.556962 scaffold_2 39356731 39549676 0.00470925908667 0.00118619009911 0.00484451348983 0.00122480048035 0.397425496548 0.399561418193 0.067965 0.0425561688564 0.035945 0.0378501645547 0.014 Chr1 122177661

N00083 2800000 3000000 0.556962 scaffold_2 39148385 39356731 0.00440455388558 0.00131625305011 0.00449230406521 0.00134573995524 0.387641056252 0.39030693204 0.091175 0.110407687213 0.02511 0.0290622330162 0.111 Chr1 122177661

N00083 3000000 3200000 0.556962 scaffold_2 38951540 39147776 0.00476771359552 0.00134600482207 0.00479843945818 0.00137159034172 0.348617474051 0.34872872752 0.07853 0.0696151572596 0.00973 0.0216168287164 0.069 Chr1 122177661

N00083 3200000 3400000 0.6496406 scaffold_2 38762527 38951540 0.00418822190859 0.00140949289044 0.00419266631857 0.00141041810151 0.382513846693 0.38901249452 0.086505 0.0590435578505 0.00109 0.00117452238735 0.069 Chr1 122177661

N00083 3400000 3600000 1.19932 scaffold_2 38557853 38762527 0.00438598559723 0.00130769136972 0.00439137510119 0.00130926553284 0.390270752261 0.390758308141 0.086265 0.0890440407673 0.002395 0.00237450775379 NA Chr1 122177661

N00098 1200000 1400000 2.546218 scaffold_2 36497767 36696813 0.00506691635115 0.00106999973654 0.00506879599338 0.00107037171813 0.35529335391 0.356595825618 0.105465 0.117319614561 0.000375 0.000376797323232 0.046 Chr1 122177661

N00098 1400000 1600000 1.602291 scaffold_2 36696813 36890440 0.0042665053068 0.00113131704812 0.00429454073063 0.00113786835423 0.375361872849 0.377774110139 0.079265 0.0869610126687 0.01006 0.00678107908504 0.0415 Chr1 122177661

N00098 1600000 1800000 1.40327 scaffold_2 36892009 37092479 0.00414658168432 0.00109425648008 0.00418545468807 0.00110699284998 0.383316923007 0.385284643739 0.08265 0.0971067990223 0.01391 0.0132089589465 0.091 Chr1 122177661

N00098 1800000 2000000 2.474053 scaffold_2 37093326 37286155 0.00364108905674 0.00105461939475 0.00369705931071 0.00107231273915 0.407294057638 0.408220242555 0.080565 0.056765320569 0.018335 0.0193435634682 0.034 Chr1 122177661

N00098 2000000 2200000 1.498547 scaffold_2 37286155 37484036 0.00446459750936 0.00111747040111 0.00448763893035 0.00112679983061 0.356735851057 0.354727762211 0.082 0.0817460999287 0.00782 0.00790374012664 NA Chr1 122177661

N00098 2200000 2400000 1.695157 scaffold_2 37484036 37675525 0.00436108241253 0.00135132842132 0.00438274209615 0.00135880004607 0.353792975671 0.351683865647 0.096555 0.0917598399908 0.00602 0.00628756743207 NA Chr1 122177661

N00098 2400000 2600000 2.23433 scaffold_2 37675525 37871007 0.0045163946744 0.00133920831559 0.00451871255457 0.00136346672604 0.343277801509 0.345081743124 0.084295 0.0708863220143 0.000525 0.0207742912391 NA Chr1 122177661

N00098 2800000 3000000 1.19932 scaffold_2 38064229 38262095 0.00394965992794 0.00121124621135 0.00394965992794 0.00121171212689 0.377721791315 0.376901912686 0.089335 0.102326827247 0 0.000313343373798 NA Chr1 122177661

N00020 400000 600000 1.699281 scaffold_2 34783656 34979234 0.00297445826579 0.000951590679012 0.00302659576375 0.000971216449895 0.376387032086 0.372923638915 0.080115 0.0529302886828 0.02187 0.0246039943143 0.031 Chr1 122177661

N00020 600000 800000 2.087543 scaffold_2 34592250 34783656 0.0039259350503 0.000824052195897 0.0040303625798 0.000849078793709 0.404504585238 0.406210893573 0.103565 0.0683102933032 0.027975 0.0288078743613 0.05475 Chr1 122177661

N00020 800000 1000000 1.749747 scaffold_2 34397946 34592250 0.00391847505816 0.000934601930599 0.00403851596057 0.000965437875369 0.399120838655 0.399927055967 0.09058 0.0511878293808 0.03172 0.0328299983531 0.052 Chr1 122177661

N00020 1000000 1200000 0.705497 scaffold_2 34213891 34397946 0.00351121027087 0.000827203269348 0.00355950610338 0.00083824975857 0.421078644459 0.423380049836 0.08983 0.0620847029421 0.01174 0.0138871532966 0.016 Chr1 122177661

N00020 1200000 1400000 0.4464061 scaffold_2 34025309 34213891 0.00405113403302 0.00111495260064 0.00405113403302 0.00111495260064 0.398717836129 0.39956842703 0.11948 0.0894358952604 0 0 NA Chr1 122177661

N00020 1400000 1600000 0.4464061 scaffold_2 33825512 34025309 0.00408237450116 0.00102412762093 0.00409724943839 0.00102912246103 0.399505135753 0.40152912358 0.11849 0.0980345050226 0.00584 0.0058609488631 0.099 Chr1 122177661

N00020 1600000 1800000 0.4464061 scaffold_2 33632565 33825512 0.00443315120713 0.00105157679587 0.0045107912617 0.0010734740034 0.39036489277 0.389594440105 0.088555 0.0716621662944 0.01995 0.0203942015165 0.061 Chr1 122177661

N00020 1800000 2000000 1.398656 scaffold_2 33433518 33632565 0.00395927017482 0.000994609499305 0.00400611851373 0.0010079136222 0.389298644121 0.388508716679 0.040605 0.0438137726266 0.02043 0.0205479107949 0.085 Chr1 122177661

N00020 2000000 2200000 1.657812 scaffold_2 33231991 33433518 0.00432725401965 0.000936354398374 0.00432725401965 0.000937381394719 0.393742537503 0.397738957377 0.09518 0.0976047874478 0 0.00258526152823 NA Chr1 122177661

N00020 2200000 2400000 1.657812 scaffold_2 33039828 33231991 0.00463977652255 0.000965074218808 0.00463977652255 0.000965074218808 0.390720376828 0.38859089393 0.085215 0.0821802324069 0 0 NA Chr1 122177661

N00020 2400000 2600000 0.6845693 scaffold_2 32846999 33039828 0.00423183935433 0.00120827922671 0.0042892013762 0.00123213405332 0.375639523417 0.374045325779 0.100645 0.0728261827837 0.016805 0.0195406292622 0.035 Chr1 122177661

N00020 2600000 2800000 1.86364 scaffold_2 32657063 32846999 0.00437646898956 0.00103501513658 0.00444809297395 0.00104948560666 0.384921824555 0.387425913847 0.098155 0.0526966978351 0.02091 0.021423005644 0.052 Chr1 122177661

N00020 2800000 3000000 1.286982 scaffold_2 32462295 32657063 0.00429213464757 0.00119486996522 0.00440114564252 0.00122948230158 0.384073242751 0.381347692343 0.086575 0.0612112872751 0.029005 0.0318840877352 0.039 Chr1 122177661

N00020 3000000 3200000 2.771786 scaffold_2 32258819 32462295 0.00407038002184 0.00117198950366 0.00421857454886 0.00121684899185 0.388224315877 0.383515268366 0.07644 0.0722738799662 0.041785 0.0474748864731 0.06 Chr1 122177661

N00020 3200000 3400000 3.763662 scaffold_2 32063616 32258819 0.00422892512454 0.00125816975442 0.00427210715336 0.00127403653527 0.384264110136 0.383190522808 0.07324 0.0539438430762 0.01759 0.0189187666173 0.0663333333333 Chr1 122177661

N00020 3400000 3600000 2.833724 scaffold_2 31873107 32063616 0.00339068993466 0.000986007046341 0.00342771608731 0.000998952763325 0.419705810498 0.419282326366 0.072515 0.0528426478539 0.019065 0.0119469421392 0.063 Chr1 122177661

N00020 3800000 4000000 1.835829 scaffold_2 31468369 31655562 0.00423649516208 0.00105052059664 0.00423649516208 0.00105052059664 0.42558731761 0.431320489753 0.086605 0.0597084292682 0 0 NA Chr1 122177661

N00020 4000000 4200000 2.146069 scaffold_2 31275858 31468369 0.00421671525375 0.00122408820123 0.00428536838573 0.00124802578451 0.400822298466 0.402899637405 0.08433 0.0581213541044 0.02207 0.0224402761401 0.0683333333333 Chr1 122177661

N00020 4200000 4400000 0.9184272 scaffold_2 31077163 31275197 0.00446414810201 0.000999326336169 0.00452231735113 0.00101428778424 0.398601200909 0.39649881157 0.10299 0.083051395215 0.01429 0.0149519779432 0.051 Chr1 122177661

N00020 4400000 4600000 2.107731 scaffold_2 30876013 31077163 0.00444136172415 0.00104158967265 0.00446050301575 0.00104705901951 0.408886626172 0.411241281961 0.15458 0.1260899826 0.005805 0.00578672632364 0.07 Chr1 122177661

N00020 4600000 4800000 2.107731 scaffold_2 30684598 30876013 0.00450029551915 0.00114242347105 0.00457503000311 0.00116649462594 0.401467860404 0.402874896297 0.082615 0.0519865214325 0.025155 0.0266645769663 0.062 Chr1 122177661

N00020 4800000 5000000 2.107731 scaffold_2 30488860 30684598 0.00486623954823 0.00109137767795 0.00490827713555 0.00110816850728 0.405990794043 0.407645946924 0.10888 0.0800764286955 0.020855 0.0217178064556 0.104666666667 Chr1 122177661

N00020 5000000 5200000 2.687013 scaffold_2 30299305 30488860 0.00465840700885 0.00128510173166 0.00469407801048 0.00131120599808 0.394245486909 0.394267828102 0.080815 0.0538629949091 0.021145 0.023138403102 0.0713333333333 Chr1 122177661

N00020 5200000 5400000 3.510569 scaffold_2 30103070 30299305 0.00457431364348 0.00109399096296 0.00460091896868 0.00110069642183 0.389535047136 0.391661332072 0.096375 0.0831757841364 0.005395 0.00646164038016 0.051 Chr1 122177661

N00020 5600000 5800000 3.164291 scaffold_2 29722737 29908944 0.00395645313248 0.00111747863646 0.00401219712422 0.00115219134508 0.395959620354 0.394353209051 0.080105 0.0628279280586 0.03129 0.0357559060615 0.0872 Chr1 122177661

N00020 5800000 6000000 2.487013 scaffold_2 29520585 29722737 0.00509980183533 0.00127649452346 0.00511326513065 0.00128266898183 0.399167120687 0.40325795886 0.0731 0.0669100478848 0.006285 0.00616367881594 NA Chr1 122177661

N00020 6000000 6200000 2.487013 scaffold_2 29338001 29520585 0.00434629560445 0.00117191624211 0.00431863654873 0.00117543314811 0.415054413543 0.416611303227 0.088235 0.0469865924725 0.01636 0.0179588572931 0.121 Chr1 122177661

N00020 6200000 6400000 2.482299 scaffold_2 29129797 29338001 0.00433019484919 0.000941383409653 0.00442842527161 0.000971123297595 0.379213254845 0.382745396163 0.08575 0.121198439992 0.02383 0.0451048010605 0.046 Chr1 122177661

N00020 6400000 6600000 2.468792 scaffold_2 28929345 29129797 0.00502036491708 0.00118154997442 0.00502036491708 0.00118154997442 0.382015439182 0.383815625018 0.103105 0.0845539081675 0 0 0.055 Chr1 122177661

N00020 6600000 6800000 2.468792 scaffold_2 28744230 28929345 0.00425741044413 0.0011453461168 0.00427703168277 0.00115623211752 0.400180205646 0.399115362566 0.102525 0.049688031764 0.0078 0.00971828322934 0.07 Chr1 122177661

N00020 6800000 7000000 2.468792 scaffold_2 28557334 28744196 0.00459021646805 0.00122039761466 0.00459021646805 0.00122048834544 0.405411008023 0.406766710967 0.07025 0.0389966927465 0 6.42185141976e-05 NA Chr1 122177661

N00020 7000000 7200000 1.82215 scaffold_2 28359564 28557334 0.00448122459628 0.00123013032542 0.00451180606067 0.00123970862354 0.393997070939 0.394522559998 0.05602 0.0616119735046 0.011695 0.0119836173333 0.189 Chr1 122177661

N00020 7200000 7400000 1.155914 scaffold_2 28164541 28359564 0.00464213848315 0.00124225578344 0.00464585849873 0.00124380724703 0.383197373766 0.387944216606 0.06952 0.0616440112192 0.005085 0.00338421622065 0.079 Chr1 122177661

N00020 7400000 7600000 1.85628 scaffold_2 27974759 28164541 0.004791124639 0.00125297381058 0.0048417019526 0.00126768077648 0.38390109307 0.385983547279 0.09887 0.0881853916599 0.015945 0.0168351055421 0.085 Chr1 122177661

N00020 7600000 7800000 0.5407446 scaffold_2 27777736 27974759 0.00410850094141 0.000980592983497 0.00418146664075 0.00100589458712 0.390245561912 0.388654044453 0.05214 0.0527451109769 0.021715 0.0285550417972 0.0723333333333 Chr1 122177661

N00020 7800000 8000000 0.6678779 scaffold_2 27591116 27777736 0.0041040195382 0.00104954398197 0.00416558639253 0.00108077338799 0.392056846326 0.390843060669 0.078465 0.0474707962705 0.027905 0.0354999464152 0.0813333333333 Chr1 122177661

N00020 8000000 8200000 1.342435 scaffold_2 27402800 27590895 0.0039525444325 0.00111335897342 0.00395392517903 0.00111370317294 0.383707356517 0.382834856202 0.094045 0.0533347510566 0.00029 0.000308354820702 0.064 Chr1 122177661

N00020 8200000 8400000 1.342435 scaffold_2 27200909 27402800 0.00462805115749 0.00121225852422 0.00462805115749 0.00121225852422 0.386634884744 0.388301597543 0.09133 0.0912026786731 0 0 NA Chr1 122177661

N00020 8400000 8600000 1.342435 scaffold_2 27004562 27200909 0.00480336313569 0.00113497025738 0.00483942452898 0.00114410044314 0.366254929553 0.367715299381 0.12098 0.101305342073 0.00914 0.0103337458683 0.17 Chr1 122177661

N00020 8800000 9000000 1.006114 scaffold_2 26621051 26822550 0.00528619191008 0.00121071526008 0.00530523002845 0.00121729706881 0.357083251659 0.357992502718 0.071715 0.105811939513 0.00379 0.00522583238626 0.066 Chr1 122177661

N00020 11000000 11200000 0.1319099 scaffold_2 24393604 24591325 0.0046634013079 0.00111119707746 0.00468542042828 0.00111737126841 0.365632641846 0.366311486579 0.07782 0.0637716782739 0.00477 0.00488061460341 0.05 Chr1 122177661

N00020 11200000 11400000 1.152778 scaffold_2 24189362 24393604 0.00458449663576 0.0012719904498 0.00466725669367 0.00130834702524 0.371036981378 0.374881880463 0.107765 0.106275888407 0.042385 0.0415830240597 0.115 Chr1 122177661

N00293 0 200000 1.81974 scaffold_2 23687688 23877468 0.00456183888587 0.00109536397721 0.0045649200197 0.00109617404421 0.379903172212 0.383270580231 0.13019 0.107756349457 0.00163 0.00255559068395 NA Chr1 122177661

N00293 200000 400000 5.338549 scaffold_2 23488618 23687688 0.00409371812951 0.00095588068321 0.00409318372184 0.000956162245616 0.392794347486 0.397630623018 0.073 0.0419651378912 0.016755 0.00923795649771 0.058 Chr1 122177661

N00350 0 200000 2.942408 scaffold_2 23191082 23390617 0.00435922033261 0.00114600862024 0.00440926737023 0.00116062348024 0.398868731996 0.399997823911 0.064495 0.0564261908938 0.02007 0.016779011201 NA Chr1 122177661

N00167 200000 400000 2.547215 scaffold_2 21969996 22165648 0.00490287866057 0.00113742900541 0.00499746143327 0.00116069918217 0.370589816405 0.370913509555 0.07926 0.0869042994705 0.01878 0.0224991311103 0.048 Chr1 122177661

N00167 400000 600000 1.541276 scaffold_2 22165648 22352685 0.00441217287013 0.00115494233381 0.00444734456169 0.00116671772606 0.405135951662 0.407569132797 0.09446 0.0842560562883 0.009995 0.0107091110315 NA Chr1 122177661

N00167 800000 1000000 1.541276 scaffold_2 22554304 22742686 0.00426929654098 0.00103130800678 0.00426929654098 0.00103130800678 0.382322310375 0.387839347559 0.09973 0.078208109055 0 0 NA Chr1 122177661

N00167 1000000 1200000 1.541276 scaffold_2 22742686 22932068 0.00444133786468 0.00115487748723 0.00448912510895 0.00116837944227 0.391539971292 0.39469901483 0.1319 0.0717333220686 0.012345 0.0125091085742 0.053 Chr1 122177661

N00167 1200000 1400000 1.426255 scaffold_2 22932068 23123876 0.00408246111813 0.00107770708451 0.00415832511228 0.00110237195259 0.407447185245 0.408204589771 0.108815 0.0582978812145 0.023195 0.0236017267267 0.0555 Chr1 122177661

N00005 200000 400000 2.962926 scaffold_2 21407822 21606081 0.00413463187713 0.00129801806419 0.00423390578413 0.00133408249512 0.392193037549 0.390924308297 0.096375 0.057833440096 0.02912 0.02905794945 0.0343333333333 Chr1 122177661

N00005 400000 600000 1.190722 scaffold_2 21210098 21407822 0.00423251906485 0.00099295667886 0.00431734343362 0.00101846631209 0.414010989011 0.410854691844 0.07279 0.0510155570391 0.023835 0.0239222350347 0.071 Chr1 122177661

N00005 600000 800000 1.735333 scaffold_2 21012800 21210098 0.00409097861941 0.00105750369961 0.00418803659439 0.0010837582002 0.393044029507 0.390688729563 0.107655 0.0746434327768 0.032215 0.031313039159 0.045 Chr1 122177661

N00005 800000 1000000 3.91403 scaffold_2 20825752 21012800 0.00406580083637 0.000983679289584 0.00408381471754 0.000990324846487 0.415936269117 0.414049303746 0.11143 0.0703616184081 0.020435 0.0142423335187 0.0795 Chr1 122177661

N00005 1000000 1200000 3.694796 scaffold_2 20639623 20825752 0.00394418297561 0.00108409328912 0.00394418297561 0.00108413329456 0.411558317015 0.41063103974 0.082105 0.0450064202784 0 4.83535612398e-05 NA Chr1 122177661

N00005 1200000 1400000 3.694796 scaffold_2 20450148 20639623 0.00420590551146 0.00099708039183 0.0044434109772 0.00105717387257 0.39512973515 0.393833542097 0.08131 0.0594748647579 0.06533 0.0708695078506 0.069 Chr1 122177661

N00005 1400000 1600000 3.694796 scaffold_2 20249980 20450148 0.00485811047228 0.00102131405165 0.00497904790444 0.00105875695935 0.386261110885 0.385044544022 0.11421 0.0891201390832 0.038865 0.0390721793693 0.0903333333333 Chr1 122177661

N00005 1600000 1800000 1.008003 scaffold_2 20055106 20249980 0.00499449664848 0.00119259790051 0.00499449664848 0.00119259790051 0.391893361416 0.390490394675 0.096985 0.102810020834 0 0.0157178484559 NA Chr1 122177661

N00005 1800000 2000000 0.5731463 scaffold_2 19867248 20055106 0.00457863089351 0.00141813028668 0.00462063678704 0.0014334225449 0.398802015805 0.401309220016 0.090305 0.0771274047419 0.008365 0.0101566076505 0.061 Chr1 122177661

N00005 2000000 2200000 0.5731463 scaffold_2 19664998 19867248 0.00513876958643 0.00137358022938 0.0051750096839 0.00138572471143 0.385991694512 0.383915055932 0.06715 0.0603807169345 0.008595 0.00838071693449 0.1335 Chr1 122177661

N00005 2200000 2400000 0.5731463 scaffold_2 19470037 19664998 0.00452345842073 0.00122047967108 0.00454956913282 0.00123139930582 0.404279116354 0.403409184395 0.07827 0.0578064330815 0.00997 0.0115561573853 0.0826666666667 Chr1 122177661

N00005 2400000 2600000 0.5731463 scaffold_2 19273774 19470037 0.00486067042727 0.0012298976269 0.00493176388697 0.00125489517496 0.407013343226 0.405490333245 0.09302 0.0661000799947 0.020925 0.0203604347228 0.089 Chr1 122177661

N00005 2800000 3000000 4.957738 scaffold_2 18896092 19091218 0.00359248602395 0.000922295599567 0.00359248602395 0.000922295599567 0.381433858598 0.381870058403 0.065655 0.0501419595543 0 0 NA Chr1 122177661

N00005 3000000 3200000 2.484663 scaffold_2 18698599 18896092 0.00403713689119 0.000974232203359 0.00403713689119 0.000974232203359 0.374522335695 0.371091724964 0.103015 0.0887778301003 0 0 NA Chr1 122177661

N00005 3200000 3400000 2.484663 scaffold_2 18503588 18698599 0.00418195132761 0.000902770298441 0.00418195132761 0.000902770298441 0.367673533599 0.367396726465 0.083845 0.074272733333 0 0 NA Chr1 122177661

N00005 3400000 3600000 2.484663 scaffold_2 18308322 18503588 0.00383500041545 0.000967118518995 0.00383500041545 0.000967118518995 0.374633954416 0.376632921465 0.092485 0.0878340315262 0 0 NA Chr1 122177661

N00005 3600000 3800000 2.484663 scaffold_2 18119059 18308322 0.00447820621603 0.00113276022896 0.00456647485257 0.00116291811706 0.390794155938 0.395277520532 0.06889 0.0475370251977 0.02913 0.0329647104822 0.101666666667 Chr1 122177661

N00005 3800000 4000000 2.484663 scaffold_2 17910100 18119059 0.00470137452733 0.00131076629829 0.00485641849835 0.00136321422828 0.392134607158 0.391780132323 0.114055 0.105867658249 0.04623 0.0458702424878 0.101 Chr1 122177661

N00005 4000000 4200000 3.543984 scaffold_2 17713973 17909649 0.00377261997339 0.000955163715797 0.00383733313449 0.000977441811541 0.424878070809 0.423345506861 0.07265 0.0693033381713 0.022415 0.0239528608516 0.0605 Chr1 122177661

N00005 4200000 4400000 3.744565 scaffold_2 17519792 17713973 0.00429633161774 0.0011724700307 0.00435972174129 0.00119700624041 0.404548762316 0.402806148649 0.096715 0.0527291547577 0.022 0.0245750099134 0.0542 Chr1 122177661

N00005 4400000 4600000 3.744565 scaffold_2 17329584 17519792 0.0037115701853 0.00109405748912 0.00378939007975 0.00112093376745 0.418933164664 0.420359239989 0.105115 0.0783037516824 0.026645 0.0297831847241 0.0653333333333 Chr1 122177661

N00005 4600000 4800000 3.129786 scaffold_2 17139259 17329584 0.00411624608026 0.00101734234711 0.00419937008571 0.00103861605976 0.418250431666 0.419671423132 0.09933 0.077604098253 0.02048 0.0203651648496 0.049 Chr1 122177661

N00005 4800000 5000000 0.9902213 scaffold_2 16949622 17139259 0.00378656511994 0.00091476343693 0.00378781175907 0.000915580905981 0.399802745689 0.406754999008 0.088685 0.0567294357114 0.004035 0.00571091084546 NA Chr1 122177661

N00005 5000000 5200000 1.793103 scaffold_2 16758459 16949622 0.0037291380544 0.00108763911156 0.00375130724942 0.00109677912461 0.410781966051 0.412650244603 0.06522 0.042126352903 0.008905 0.0115032720767 0.053 Chr1 122177661

N00005 5200000 5400000 4.776858 scaffold_2 16557384 16758459 0.00437709709044 0.00118158968237 0.00453196253985 0.0012521750743 0.386822375173 0.383576866018 0.072965 0.0671838866095 0.055455 0.0623747357951 0.055 Chr1 122177661

N00005 5400000 5600000 4.421593 scaffold_2 16363330 16557384 0.00424986822791 0.00102768532701 0.00432827605077 0.00105011331205 0.391535638531 0.389439397102 0.061725 0.0394117101425 0.02736 0.0282137961598 0.0903333333333 Chr1 122177661

N00005 5600000 5800000 2.62256 scaffold_2 16167988 16363330 0.00420289557097 0.00104022644181 0.00433763195318 0.00108183162117 0.380898526306 0.376443469449 0.06057 0.0636012736636 0.041395 0.043559500773 0.0475 Chr1 122177661

N00005 5800000 6000000 2.62256 scaffold_2 15973925 16167988 0.00400913250362 0.00110546225534 0.00412854075187 0.0011487860916 0.38580679343 0.383663701099 0.099945 0.0667978955288 0.042895 0.0432849126315 0.062 Chr1 122177661

N00005 6000000 6200000 2.62256 scaffold_2 15780418 15973925 0.00406641672361 0.00103217808282 0.00414022712281 0.00105281848983 0.40584702098 0.403902394959 0.09372 0.070472902789 0.020555 0.0218338354685 0.046 Chr1 122177661

N00005 6200000 6400000 2.62256 scaffold_2 15579819 15780418 0.00429245339803 0.00114455605288 0.00429245339803 0.00114458045651 0.405558282878 0.408271759687 0.09028 0.0822785756659 0.00011 0.00220838588428 0.046 Chr1 122177661

N00005 6800000 7000000 1.37407 scaffold_2 15012194 15206706 0.00433409644236 0.00116152169532 0.00435463083035 0.00116766616104 0.374439159329 0.378714367303 0.092465 0.0799590770749 0.00577 0.00566031915769 0.043 Chr1 122177661

N00005 7000000 7200000 0.7069388 scaffold_2 14807316 15012194 0.00436026511598 0.00124079678284 0.00436605701869 0.00124286755133 0.377529526604 0.375985035421 0.119425 0.143602534191 0.00111 0.0162975038804 0.045 Chr1 122177661

N00005 7200000 7400000 0.7069388 scaffold_2 14613121 14807316 0.00445008879002 0.000949497727728 0.00464321694417 0.00100006431591 0.375322906392 0.370751779235 0.053995 0.0684003192667 0.051645 0.054501918175 0.045 Chr1 122177661

N00005 7400000 7600000 0.7069388 scaffold_2 14412519 14613121 0.00389099273456 0.00125440154604 0.0040993992257 0.00132362884583 0.384674217655 0.380203952902 0.081115 0.0721478350166 0.06266 0.0639076380096 0.05475 Chr1 122177661

N00005 7600000 7800000 0.7069388 scaffold_2 14203627 14411676 0.00452612289223 0.00116218957654 0.00461080946232 0.00119649034036 0.395355683118 0.393801774042 0.095745 0.0961984917015 0.03705 0.0269503818812 0.0666666666667 Chr1 122177661

N00005 7800000 8000000 0.7069388 scaffold_2 14005918 14203627 0.00388996441434 0.00119012054763 0.0040011948993 0.00123853539644 0.404154752189 0.401763609397 0.083875 0.0764558012028 0.046255 0.0475800292349 0.06175 Chr1 122177661

N00005 8000000 8200000 1.130684 scaffold_2 13803060 14005918 0.00402999686865 0.00101166260336 0.00406668838784 0.00102495150566 0.389842980602 0.385730880204 0.053995 0.0777046012482 0.01193 0.0130140295182 0.0575 Chr1 122177661

N00005 8200000 8400000 1.510455 scaffold_2 13608874 13803060 0.00389345435277 0.000969165804293 0.00389455283396 0.000970172281931 0.40551902736 0.407680089174 0.098595 0.0645412130638 0.00438 0.00324431215433 0.023 Chr1 122177661

N00005 8400000 8600000 1.510455 scaffold_2 13418869 13608874 0.00442089807154 0.000898271597167 0.00442089807154 0.000898449495281 0.381792985698 0.382688239666 0.125385 0.0969290281835 0 0.000852609141865 NA Chr1 122177661

N00005 8600000 8800000 1.510455 scaffold_2 13209008 13418869 0.00435693157716 0.00107244593474 0.00437798147384 0.00107767133617 0.379721406537 0.381369422305 0.08744 0.0899357193571 0.00963 0.00538928147679 0.135 Chr1 122177661

N00005 8800000 9000000 0.8115878 scaffold_2 13005181 13209008 0.00488160698466 0.00115730913464 0.00500545583033 0.0012047676483 0.395868743279 0.393729508197 0.102655 0.0925049183867 0.043725 0.051018756102 0.0796 Chr1 122177661

N00005 9000000 9200000 0 scaffold_2 12802032 13005181 0.00473979290669 0.00142047417774 0.00474301126525 0.00142050794675 0.384928682674 0.386295586734 0.12657 0.102018715327 0.000975 0.000974654071642 0.057 Chr1 122177661

N00005 9200000 9400000 0.7587566 scaffold_2 12613336 12802032 0.00436335407618 0.00128081904919 0.00441152914321 0.00129653535484 0.400297119309 0.404558936614 0.075705 0.0368158307542 0.013435 0.0142610336202 0.057 Chr1 122177661

N00005 9400000 9600000 2.006711 scaffold_2 12421357 12613276 0.00470395554041 0.00124160675401 0.00479290192087 0.00127399718853 0.389413699918 0.388887928999 0.10036 0.0759591285907 0.03145 0.0339361918309 0.1416 Chr1 122177661

N00005 9800000 10000000 2.006711 scaffold_2 12041015 12232190 0.00468604296902 0.00125041170707 0.00468604296902 0.00125041170707 0.358240251393 0.35596473806 0.09872 0.0797018438603 0 0 NA Chr1 122177661

N00005 10000000 10200000 2.006711 scaffold_2 11848534 12041015 0.00491749110447 0.00127515537679 0.00491749110447 0.00127515537679 0.349106990014 0.346392186859 0.09777 0.0871514591051 0 0 NA Chr1 122177661

N00005 10200000 10400000 2.006711 scaffold_2 11655636 11848534 0.00424566617366 0.00117981541708 0.00425613540511 0.00118221420441 0.363988467417 0.362565034087 0.10576 0.0956826924074 0.00231 0.00267498885421 NA Chr1 122177661

N00005 10400000 10600000 1.433178 scaffold_2 11467565 11655636 0.00364543581011 0.00114304869403 0.00366499515139 0.00114788684137 0.379115327956 0.383277662026 0.08376 0.0347794184111 0.009125 0.00797571130052 0.018 Chr1 122177661

N00005 10600000 10800000 0.6343284 scaffold_2 11275174 11467565 0.0036148655656 0.00108429947817 0.00361512718807 0.00108429947817 0.367566633403 0.368967785806 0.09467 0.0674615756454 0.007965 0 NA Chr1 122177661

N00005 10800000 11000000 0.6343284 scaffold_2 11083083 11275174 0.00485475966023 0.00116252844691 0.00485475966023 0.00116252844691 0.348639911513 0.34676403644 0.114675 0.0869275499633 0 0 NA Chr1 122177661

N00005 11600000 11800000 0.6343284 scaffold_2 10305668 10496825 0.00498976637183 0.00145529313973 0.00498976637183 0.00145529313973 0.346396514926 0.344954837409 0.10705 0.103328677475 0 0 NA Chr1 122177661

N00005 11800000 12000000 0.6343284 scaffold_2 10110249 10305668 0.00473007875401 0.00147118581236 0.00473007875401 0.00147118581236 0.351819897859 0.351392344661 0.11337 0.11624765248 0 0 NA Chr1 122177661

N00005 12000000 12200000 0.6343284 scaffold_2 9917552 10110249 0.00429366025649 0.00112575027807 0.0043063409115 0.00113190796801 0.37787593817 0.377967086998 0.065285 0.0648064059119 0.005425 0.00590045511866 0.11 Chr1 122177661

N00005 12200000 12400000 1.472449 scaffold_2 9716858 9917552 0.00442114092726 0.00122520251435 0.00465437351264 0.00129681897792 0.383589868634 0.379851589268 0.14004 0.10807497982 0.058845 0.0632953650832 0.048 Chr1 122177661

N00005 12400000 12600000 1.820682 scaffold_2 9527911 9716362 0.00427313358648 0.00108682499564 0.00427671174407 0.00108824817469 0.385513608428 0.389241007764 0.11555 0.0784182625722 0.00083 0.00110373518846 NA Chr1 122177661

N00005 12600000 12800000 1.820682 scaffold_2 9336843 9527911 0.00477944710875 0.00135043612591 0.00478648364397 0.0013520120093 0.367143607099 0.369207104207 0.071915 0.05232168652 0.002145 0.00112525383633 NA Chr1 122177661

N00005 12800000 13000000 1.820682 scaffold_2 9133203 9336843 0.00481785620759 0.00118642020726 0.00485045010946 0.00119550645176 0.368721918357 0.366836873299 0.090525 0.0917796110784 0.00763 0.00879493223335 0.024 Chr1 122177661

N00005 13000000 13200000 4.638641 scaffold_2 8927331 9133203 0.00463524182184 0.00122305802865 0.00469423031929 0.00123716259832 0.368456998173 0.363182884594 0.09042 0.111103015466 0.014445 0.0140329913733 0.046 Chr1 122177661

N00005 13200000 13400000 5.443202 scaffold_2 8731187 8927331 0.00465654108071 0.0011847025075 0.00471385412325 0.00120327825466 0.388327409409 0.388918373846 0.08937 0.0752202463496 0.015435 0.0148411371237 0.047 Chr1 122177661

N00005 13400000 13600000 2.783282 scaffold_2 8533683 8731187 0.00347966198816 0.00107691346386 0.0035039759145 0.00108733330773 0.385984850605 0.383465886007 0.088095 0.0790262475697 0.01348 0.0134630184705 0.0473333333333 Chr1 122177661

N00005 13600000 13800000 2.783282 scaffold_2 8342536 8533683 0.00373576500704 0.000992434655686 0.00376029632182 0.000998816584281 0.387752872307 0.386323083491 0.069215 0.0461634239617 0.0141 0.0131155602756 0.043 Chr1 122177661

N00005 13800000 14000000 2.014228 scaffold_2 8150019 8342536 0.00312458660766 0.000834874737062 0.00313314508937 0.000838040726633 0.41863966464 0.429259401702 0.098995 0.0482502843905 0.00396 0.00821745612076 0.119 Chr1 122177661

N00005 14000000 14200000 1.410625 scaffold_2 7962200 8150019 0.00420389325375 0.00101321587099 0.00428300024931 0.00103960537447 0.403228345503 0.412764481443 0.07789 0.0809236552213 0.028515 0.0325046986727 0.047 Chr1 122177661

N00005 14200000 14400000 1.128762 scaffold_2 7763466 7962200 0.0046959350348 0.00108542876399 0.00484359295839 0.00113516471322 0.3714392448 0.372249037272 0.099735 0.102624613805 0.0359 0.0419253877042 0.0746666666667 Chr1 122177661

N00005 14400000 14600000 3.887419 scaffold_2 7568580 7763466 0.0044532473752 0.00114707892258 0.00455414549104 0.0011723068199 0.396594306512 0.396761019733 0.082905 0.055417013023 0.028235 0.0289092084603 0.0736666666667 Chr1 122177661

N00005 14600000 14800000 2.739449 scaffold_2 7372734 7568580 0.00465268062627 0.0010268120399 0.00471098532061 0.00104515089101 0.392740707316 0.393141978058 0.119025 0.065367686856 0.01641 0.017263564229 0.0505 Chr1 122177661

N00005 14800000 15000000 2.739449 scaffold_2 7173005 7372724 0.00396205352197 0.000959146410171 0.0039843316106 0.000961810699289 0.414231558147 0.414285323485 0.07879 0.0549472008171 0.007425 0.00549271726776 0.0295 Chr1 122177661

N00005 15000000 15200000 2.739449 scaffold_2 6972584 7173005 0.00439854157661 0.00112945065364 0.0044207060104 0.00113825659595 0.411543090932 0.411011502219 0.09616 0.0616152997939 0.009135 0.00925551713643 0.043 Chr1 122177661

N00005 15200000 15400000 2.739449 scaffold_2 6757077 6972584 0.00502641086604 0.00120797957578 0.00502652499969 0.00120797957578 0.387333710001 0.385090356577 0.071835 0.092781208963 2,00E-05 0 NA Chr1 122177661

N00005 15400000 15600000 2.739449 scaffold_2 6550206 6757077 0.00504694923121 0.00143199299361 0.00517048792429 0.00148115279972 0.368299055251 0.370415069063 0.09605 0.0983173088543 0.038445 0.0362834810099 0.112 Chr1 122177661

N00005 15600000 15800000 2.739449 scaffold_2 6345003 6550206 0.00411735833375 0.00105367133932 0.00417122853277 0.00107192730288 0.397111933022 0.396971457909 0.09243 0.0767337709488 0.02089 0.0207842965259 0.130666666667 Chr1 122177661

N00005 15800000 16000000 2.739449 scaffold_2 6148387 6345003 0.00349937778867 0.000945645155603 0.00351840096525 0.000949587533397 0.408497273189 0.409913714579 0.084045 0.048948203605 0.01172 0.0104365870529 0.068 Chr1 122177661

N00005 16000000 16200000 2.739449 scaffold_2 5955641 6148272 0.00443924944217 0.00106880882064 0.00449975202077 0.00108500542724 0.397549909256 0.400689152693 0.063155 0.0456520497739 0.023395 0.0229194677908 0.08475 Chr1 122177661

N00005 16200000 16400000 2.0884 scaffold_2 5752107 5955641 0.00462126861989 0.0011805694436 0.00462589132752 0.00118174653601 0.369307517534 0.368163784222 0.086715 0.101869957845 0.00083 0.000815588550316 0.096 Chr1 122177661

N00005 16400000 16600000 1.269103 scaffold_2 5548301 5752107 0.00493652582773 0.00133737260671 0.00494315485258 0.00133804672714 0.363895222674 0.361952448925 0.09826 0.0964888177973 0.00196 0.00192339774099 0.096 Chr1 122177661

N00005 16600000 16800000 0.3314011 scaffold_2 5357162 5547690 0.0045282252597 0.00129402738174 0.00453287660701 0.00129483064531 0.366436141391 0.363403056937 0.113915 0.096710194827 0.000885 0.000572094390326 0.096 Chr1 122177661

N00005 16800000 17000000 0.5011328 scaffold_2 5159656 5357162 0.00512945600544 0.00131318901318 0.00512945600544 0.00131394534146 0.365018557568 0.364357343803 0.106015 0.10122224135 0 0.000815165108908 NA Chr1 122177661

N00005 17000000 17200000 0.5011328 scaffold_2 4959371 5159656 0.00479916734994 0.0013923387067 0.00479916734994 0.0013923387067 0.354568909302 0.35373702286 0.110455 0.109778565544 0 0 NA Chr1 122177661

N00005 17400000 17600000 0.5011328 scaffold_2 4553508 4759330 0.00516773434244 0.00143742324833 0.00517450891303 0.00144582179596 0.359552974267 0.359254913629 0.1026 0.0860695163782 0.00636 0.00673883258349 0.17 Chr1 122177661

N00005 17600000 17800000 0.5011328 scaffold_2 4356754 4553508 0.00421167622044 0.0011546117781 0.00421167622044 0.00115490735508 0.360544530549 0.360153256705 0.116815 0.0991898512864 0 0.000269371906035 NA Chr1 122177661

N00005 17800000 18000000 0.5011328 scaffold_2 4149796 4356754 0.00511689185487 0.00144752574302 0.00511689185487 0.00144752574302 0.344598854108 0.342213395425 0.120505 0.13095410663 0 0 NA Chr1 122177661

N00026 400000 600000 0.566064 scaffold_2 1713429 1922509 0.00414335664163 0.00147424619376 0.00414335664163 0.00147424619376 0.33949899413 0.33740810588 0.114445 0.126587908934 0 0 NA Chr1 122177661

N00026 2600000 2800000 0.3493614 scaffold_75 292497 477138 0.00399610115084 0.000924008177888 0.00399860007809 0.000925441323592 0.382453798532 0.385442157559 0.114655 0.0422712182018 0.00466 0.00506929663509 0.077 Chr1 122177661

N00026 2800000 3000000 0.3493614 scaffold_75 477138 665927 0.00397231255396 0.000929440323426 0.00397231255396 0.000929440323426 0.382567831978 0.381390921267 0.096495 0.0633246640429 0 0 NA Chr1 122177661

N00026 3000000 3200000 0.3493614 scaffold_75 665927 857047 0.00439401296068 0.000976988976399 0.00446704119684 0.000996040677395 0.37145632211 0.36790883615 0.094705 0.0837641272499 0.018475 0.0201234826287 0.0335 Chr1 122177661

N00026 3200000 3400000 0.3493614 scaffold_75 857047 1044666 0.004556908705 0.00122286246086 0.004556908705 0.00122286246086 0.375036836297 0.376900594398 0.073755 0.0754347907195 0 0 NA Chr1 122177661

N00026 3400000 3600000 0.3493614 scaffold_75 1044666 1233987 0.00430879263745 0.00100832795927 0.00434237630425 0.00101700603705 0.386718026815 0.390764612807 0.13005 0.077788517914 0.010345 0.00990381415691 0.061 Chr1 122177661

N00026 3600000 3800000 0.2641937 scaffold_75 1233987 1426837 0.00418317087131 0.00113628482456 0.00418317087131 0.00113628482456 0.374508372522 0.375589340051 0.10273 0.0829712211563 0 0 NA Chr1 122177661

N00026 3800000 4000000 0 scaffold_75 1426837 1619741 0.00467129722078 0.00117200504614 0.0047031233184 0.00117986536446 0.379259516657 0.380005310968 0.08492 0.060143905777 0.010385 0.0103419317381 0.0825 Chr1 122177661

N00026 4200000 4400000 4.659292 scaffold_75 1809463 2001861 0.00500357572052 0.00132091529603 0.00535290221666 0.00140570962 0.354448075527 0.352178351042 0.057375 0.0544028524205 0.073155 0.0760195012422 0.044 Chr1 122177661

N00026 4400000 4600000 0.9892026 scaffold_75 2002921 2202972 0.00461979959653 0.00112634012433 0.00467248129506 0.00113908939241 0.393420934613 0.391456192451 0.105395 0.076585470705 0.02273 0.0193700606345 0.0775 Chr1 122177661

N00026 4600000 4800000 0.7144365 scaffold_75 2202972 2406157 0.00471002732645 0.00117140825333 0.00479462943377 0.00119697839055 0.379522756476 0.377653054999 0.075405 0.0772005807515 0.02357 0.0227871151906 0.099 Chr1 122177661

N00026 4800000 5000000 0.7144365 scaffold_75 2406608 2600304 0.00470678111212 0.0013988099339 0.00473942649781 0.00140673672848 0.385368412828 0.387774933505 0.091075 0.0510542293078 0.008515 0.00675284982653 0.067 Chr1 122177661

N00026 5000000 5200000 0.7144365 scaffold_75 2600304 2802530 0.00464409171071 0.00111425978667 0.00470280724389 0.00112860422785 0.390605240358 0.390312988366 0.06113 0.0589884584574 0.016975 0.0167040835501 NA Chr1 122177661

N00026 5400000 5600000 0.7144365 scaffold_52 5555145 5751000 0.00433486171294 0.0011284817552 0.00435110863296 0.0011339953766 0.401859644729 0.403303285652 0.059045 0.0578233897526 0.004255 0.00428378136887 NA Chr1 122177661

N00026 5600000 5800000 0.7144365 scaffold_52 5364837 5555145 0.003922025432 0.00110027809725 0.00392669143151 0.00110124512471 0.395340047578 0.398482003713 0.05232 0.0528196397419 0.00168 0.00178657754797 NA Chr1 122177661

N00026 5800000 6000000 0.7144365 scaffold_52 5171015 5364837 0.00399038312822 0.00103000703093 0.00405827253342 0.0010461735852 0.373528763625 0.372232956114 0.071615 0.0536781170352 0.02307 0.0236402472372 0.071 Chr1 122177661

N00026 6000000 6200000 0.7144365 scaffold_52 4974543 5171015 0.00428964486924 0.00120189872588 0.00434309087501 0.0012213902336 0.366098038659 0.368078175896 0.089065 0.0814823486298 0.01712 0.0186438780081 0.138 Chr1 122177661

N00026 6200000 6400000 0.7144365 scaffold_52 4784213 4974543 0.00379015441942 0.00106028097413 0.00379015441942 0.00106028097413 0.362319010424 0.36211826083 0.122045 0.100635737929 0 0 NA Chr1 122177661

N00026 6400000 6600000 0.7144365 scaffold_52 4595874 4784213 0.00307274093613 0.000877119499825 0.00307274093613 0.000877430105876 0.358650609021 0.362839647501 0.073735 0.0460393227106 0 0.00027609788732 0.037 Chr1 122177661

N00026 6600000 6800000 0.7144365 scaffold_52 4405701 4595874 0.00322809638681 0.000896855953321 0.00324588069788 0.000902850187072 0.353095773117 0.353647276085 0.05771 0.0503804430703 0.005445 0.00704095744401 0.037 Chr1 122177661

N00026 7400000 7600000 0.7144365 scaffold_52 3592625 3800971 0.00465246898786 0.00141865554353 0.00468556078522 0.001428928045 0.338398352706 0.33578247035 0.124965 0.131583999693 0.00853 0.00829389573114 0.057 Chr1 122177661

N00026 8600000 8800000 1.044659 scaffold_52 2389479 2580646 0.00320407447922 0.000908221588525 0.00320407447922 0.000908308528137 0.366340756533 0.364112838507 0.0961 0.0562858652382 0 0.000109851595725 NA Chr1 122177661

N00026 8800000 9000000 1.044659 scaffold_52 2198070 2389479 0.00342616789795 0.00100546669965 0.00342616789795 0.00100568326486 0.358439206619 0.354937845113 0.09523 0.0800902778866 0 0.000344811372506 NA Chr1 122177661

N00026 9200000 9400000 1.044659 scaffold_52 1808228 2005130 0.00334450160111 0.00113821241443 0.00335076128907 0.0011409045347 0.351937135129 0.346235552104 0.12245 0.104056840459 0.00165 0.00189942204752 NA Chr1 122177661

N00023 1400000 1600000 0.8406972 scaffold_53 245817 443578 0.00367999143755 0.0013154542454 0.00370262161788 0.00133018575882 0.368393912866 0.365070886987 0.124455 0.143562178589 0.01116 0.00984521720663 NA Chr1 122177661

N00023 1800000 2000000 0.8406972 scaffold_53 646636 837752 0.00362455503845 0.00130807484459 0.00362750091323 0.00130971280916 0.365052199213 0.367000827428 0.1107 0.109797191235 0.00082 0.00216099123046 0.07 Chr1 122177661

N00023 2000000 2200000 0.8406972 scaffold_53 840009 1043836 0.00365349777712 0.00131854631054 0.00365349777712 0.00131854631054 0.358116898202 0.356079084048 0.128745 0.153797092632 0 0 NA Chr1 122177661

N00023 2200000 2400000 0.8406972 scaffold_53 1043836 1244690 0.00410418212258 0.00148210448413 0.00410418212258 0.00148210448413 0.362851231855 0.360527843218 0.121 0.118200284784 0 0 NA Chr1 122177661

N00023 2600000 2800000 0.8406972 scaffold_53 1444215 1632331 0.003710556839 0.0012579984565 0.00372713736142 0.00126478075177 0.357109094347 0.357463953723 0.113965 0.0758787131344 0.017325 0.0203278827957 0.063 Chr1 122177661

N00023 4000000 4200000 0.1675325 scaffold_53 2823104 3030040 0.00431351043486 0.00136107023493 0.00431351043486 0.00136107023493 0.361342890743 0.363246628688 0.134565 0.137076197472 0 0 NA Chr1 122177661

N00023 4200000 4400000 0.1675325 scaffold_53 3030040 3224627 0.00399767709807 0.00110165899718 0.00399767709807 0.00110165899718 0.369283205231 0.370970570102 0.11603 0.110562370559 0 0 NA Chr1 122177661

N00023 4400000 4600000 0.1675325 scaffold_53 3224627 3430552 0.00388687295564 0.00104022415794 0.00388687295564 0.00104022415794 0.384235880399 0.385299577048 0.08132 0.110345999757 0 0 NA Chr1 122177661

N00023 4600000 4800000 0.1675325 scaffold_53 3430552 3618988 0.00340438953829 0.000975666233726 0.00341868291001 0.000987325718736 0.4188169978 0.418331627373 0.09496 0.0543367509393 0.02177 0.0219862446666 0.0725 Chr1 122177661

N00023 4800000 5000000 0.4671408 scaffold_53 3618988 3813125 0.0038034876118 0.00120431609029 0.00389020269759 0.00123789588632 0.402960012044 0.401699338266 0.08597 0.0536888898046 0.03105 0.0337957215781 0.0744 Chr1 122177661

N00023 5000000 5200000 5.185972 scaffold_53 3813125 4002942 0.00442547607996 0.00111824781768 0.00453312137292 0.00115085191435 0.383886670359 0.383369842093 0.069565 0.0587829330355 0.033275 0.0299498991134 0.043 Chr1 122177661

N00023 5200000 5400000 5.185972 scaffold_53 4002942 4189401 0.00422348224717 0.00109160933998 0.00426763747352 0.0011077560093 0.371189688208 0.371464157368 0.109805 0.0768104516274 0.014805 0.0155101121426 NA Chr1 122177661

N00023 5400000 5600000 5.185972 scaffold_53 4189401 4386585 0.00435458241221 0.00108960724838 0.00447576533522 0.00113833255698 0.369830352501 0.366514962484 0.090785 0.089905874716 0.045345 0.0462055744888 0.0675 Chr1 122177661

N00023 5600000 5800000 5.185972 scaffold_53 4386585 4577353 0.00433098308316 0.00100376369889 0.00433752123548 0.00100858328497 0.408239122106 0.408364267091 0.090465 0.0712121529816 0.004465 0.00388429925354 0.048 Chr1 122177661

N00023 5800000 6000000 4.086222 scaffold_53 4577353 4770276 0.0041672490062 0.00110091821996 0.00418385552117 0.00111071052773 0.393743643618 0.39780641713 0.07223 0.0565303255703 0.01095 0.0114553474702 0.0726666666667 Chr1 122177661

N00023 6000000 6200000 2.072595 scaffold_53 4770276 4962871 0.00397901943302 0.000991185650236 0.00399254520783 0.00099678544527 0.394269308394 0.400108589664 0.084815 0.0769438458942 0.00488 0.00506762896233 0.067 Chr1 122177661

N00023 6200000 6400000 2.072595 scaffold_53 4962871 5154527 0.00442385484353 0.0010518080967 0.00443944867815 0.00106169434878 0.402654266513 0.405753660831 0.10204 0.0591267687941 0.010525 0.0114684643319 0.0925 Chr1 122177661

N00023 6400000 6600000 0.835225 scaffold_53 5154527 5346011 0.00401768513269 0.00102521363303 0.00401768513269 0.00102524291487 0.434364867171 0.445400426326 0.092075 0.0662561885066 0.00088 0.0012377013223 NA Chr1 122177661

N00023 6600000 6800000 2.522646 scaffold_53 5346011 5535942 0.00399723311281 0.00112296815081 0.00402666818904 0.0011352413096 0.412923371216 0.417180473533 0.05824 0.0332541817818 0.019565 0.020939183177 0.148 Chr1 122177661

N00023 6800000 7000000 3.60827 scaffold_53 5535942 5730912 0.00446104623158 0.00108690441431 0.00456537427965 0.00111997518245 0.402999988381 0.404627664719 0.09194 0.0715186951839 0.02895 0.0308765451095 0.0534 Chr1 122177661

N00023 7200000 7400000 1.198381 scaffold_34 8882555 9069972 0.00452623952681 0.00106291769792 0.00462184690564 0.00108964649933 0.415840459248 0.416098439863 0.110965 0.0702711066766 0.02851 0.0303761131594 0.0793333333333 Chr1 122177661

N00023 7600000 7800000 3.213147 scaffold_34 8464612 8664507 0.00484653894026 0.00127254134408 0.00484653894026 0.00127254134408 0.398400707482 0.396760742518 0.06816 0.060967007679 0 0 NA Chr1 122177661

N00023 7800000 8000000 2.317839 scaffold_34 8269317 8464612 0.00425154935597 0.00125118803601 0.00434599376627 0.00128548400944 0.396821814436 0.396718124378 0.10338 0.0742568934177 0.028875 0.0289766763102 0.0693333333333 Chr1 122177661

N00023 8200000 8400000 0.2910415 scaffold_34 7898921 8086568 0.0041050335693 0.00101292561929 0.00422220852959 0.00104235630136 0.395111449754 0.39627947096 0.093835 0.0787169525758 0.031495 0.0301683480152 0.0385 Chr1 122177661

N00023 8400000 8600000 2.350092 scaffold_34 7705954 7898921 0.00438454535134 0.00113346227158 0.00449226992844 0.00116849531103 0.404266583798 0.403568519189 0.06283 0.0419864536423 0.03681 0.0407427176668 0.0698333333333 Chr1 122177661

N00023 8600000 8800000 2.350092 scaffold_34 7508229 7704312 0.00340713334191 0.000942837118306 0.00344378025694 0.000954719976871 0.395901182144 0.397755050844 0.12914 0.0928433367503 0.01381 0.0151007481526 0.068 Chr1 122177661

N00023 8800000 9000000 2.350092 scaffold_34 7313125 7508229 0.0034474468289 0.00069281505999 0.0034474468289 0.00069281505999 0.420484238088 0.425524107342 0.059825 0.0501681154666 0 0 NA Chr1 122177661

N00023 9200000 9400000 2.350092 scaffold_34 6924627 7123613 0.00443198023915 0.0012675845179 0.00450522100557 0.00129559065606 0.408797277697 0.407055523955 0.087345 0.0611550561346 0.02482 0.0275094730283 0.094 Chr1 122177661

N00023 9400000 9600000 2.350092 scaffold_34 6729776 6924627 0.00438856997855 0.00104472754752 0.00442628385879 0.00105687911164 0.41858336129 0.418358553073 0.0848 0.0691861986851 0.01677 0.0124094821171 0.0635 Chr1 122177661

N00023 9600000 9800000 2.350092 scaffold_34 6526156 6729660 0.00456566877253 0.000966410950713 0.00480619500338 0.00103909915229 0.400370381433 0.40014831954 0.07433 0.0799640301911 0.08304 0.091821290982 0.0837142857143 Chr1 122177661

N00023 9800000 10000000 2.350092 scaffold_34 6319240 6526156 0.00370018592555 0.00102317153517 0.00374046735499 0.00103528497696 0.422666644863 0.426822020118 0.090555 0.0692358251658 0.018525 0.015281563533 0.128 Chr1 122177661

N00023 10000000 10200000 3.309168 scaffold_34 6121369 6319240 0.00395211445609 0.00103304102116 0.00396385102243 0.00103523394448 0.419816673461 0.420881421909 0.081245 0.0775202025562 0.004025 0.00408346852242 0.03 Chr1 122177661

N00023 10200000 10400000 3.386513 scaffold_34 5931427 6121369 0.00470056034136 0.00108496827117 0.00472610003571 0.00109239013334 0.407320810397 0.409686163091 0.073425 0.0524791778543 0.00805 0.00808667909151 0.0705 Chr1 122177661

N00032 200000 400000 3.025455 scaffold_34 5464878 5658667 0.00513796493737 0.00156899632094 0.00519648234759 0.00159615642369 0.380104957468 0.380750901633 0.08201 0.0569691778171 0.029325 0.0302235937024 0.082 Chr1 122177661

N00032 400000 600000 0.8323055 scaffold_34 5262305 5464878 0.00540404265343 0.00153960685919 0.00540721334546 0.00154060120096 0.368369744679 0.368100581998 0.124955 0.119137298653 0.00126 0.00177713713081 NA Chr1 122177661

N00032 600000 800000 0.7763578 scaffold_34 5066272 5262262 0.0049969520141 0.0015213463956 0.00508769894747 0.00154663828679 0.372878045418 0.375723652286 0.093005 0.0778815245676 0.02085 0.0212970049492 0.0825 Chr1 122177661

N00032 800000 1000000 0.7763578 scaffold_34 4867726 5065692 0.0051692082939 0.00137340962189 0.00520214182656 0.00138406184547 0.374949630625 0.374361670591 0.098135 0.0907125465989 0.00662 0.00670822262409 0.099 Chr1 122177661

N00032 1000000 1200000 1.13228 scaffold_34 4672533 4867726 0.00413991496523 0.00111765194359 0.00424199113353 0.00114968804403 0.390890067128 0.391156638855 0.097805 0.0783173576921 0.03368 0.0303392027378 0.0722 Chr1 122177661

N00032 1200000 1400000 1.359769 scaffold_34 4482743 4672263 0.00463497611896 0.00150009057556 0.00469511831133 0.00151419782332 0.39960927875 0.39866959257 0.109095 0.0759075559308 0.017245 0.0170958210215 0.086 Chr1 122177661

N00032 1400000 1600000 1.577181 scaffold_34 4283245 4482743 0.00450282430223 0.00104722816993 0.00451877389251 0.00105429330693 0.383653351388 0.383742318013 0.068825 0.0612036210889 0.006935 0.0081755205566 NA Chr1 122177661

N00032 1600000 1800000 1.271161 scaffold_34 4083277 4283245 0.00405942079094 0.000913209975008 0.00410028820049 0.000926036901818 0.375220177232 0.37315525585 0.06912 0.0650554088654 0.01382 0.0147123539766 NA Chr1 122177661

N00032 1800000 2000000 1.124246 scaffold_34 3899435 4081382 0.00415385209387 0.00123439943587 0.00418461685715 0.00124485009579 0.354908944609 0.357052577658 0.089045 0.039555474946 0.012355 0.0176699808186 0.011 Chr1 122177661

N00032 2000000 2200000 2.282561 scaffold_34 3698580 3897685 0.00541753351938 0.00111323576616 0.00541753351938 0.00114510679754 0.341876980346 0.344735231099 0.07446 0.0651214183471 0 0.0282012003717 NA Chr1 122177661

N00032 2400000 2600000 2.282561 scaffold_34 3309957 3508183 0.00465945978648 0.00125014491034 0.00465945978648 0.00125014491034 0.397414962984 0.398828792979 0.068075 0.080917740357 0 0 NA Chr1 122177661

N00032 2600000 2800000 2.282561 scaffold_34 3106893 3309957 0.00428843760837 0.00108123375899 0.00430267047379 0.0010877678028 0.394795865208 0.394893917495 0.08821 0.0796349919237 0.005335 0.00745085293307 0.061 Chr1 122177661

N00032 2800000 3000000 2.282561 scaffold_34 2913259 3106893 0.0040602617831 0.00108563193839 0.00413440489748 0.00110393086922 0.409424208279 0.408559094037 0.08133 0.0538283565903 0.027925 0.0289308695787 0.063 Chr1 122177661

N00032 3200000 3400000 2.282561 scaffold_34 2523740 2715768 0.00431047887715 0.0012083474309 0.00448493044986 0.00132320637405 0.373825734994 0.374515282673 0.087515 0.0522892494845 0.08856 0.10452642323 0.068 Chr1 122177661

N00032 3400000 3600000 2.282561 scaffold_34 2325138 2523740 0.00424583119263 0.00113924426245 0.00433766140558 0.00116425699538 0.377096387002 0.376754899708 0.084405 0.0570890524768 0.02339 0.0235596821784 0.055 Chr1 122177661

N00032 3600000 3800000 2.125352 scaffold_34 2120549 2325138 0.0052486236052 0.00132246594198 0.00533582054094 0.00134860927635 0.391044890834 0.389908862008 0.11007 0.0748671727219 0.018175 0.0193558793484 0.0515 Chr1 122177661

N00032 3800000 4000000 2.420701 scaffold_34 1928208 2120549 0.00394167525913 0.00117292484689 0.00397522666614 0.00119500466586 0.428457366588 0.430577096692 0.11362 0.0719087454053 0.022815 0.0238482694797 0.0885 Chr1 122177661

N00032 4000000 4200000 5.156364 scaffold_34 1729045 1928208 0.00404618232542 0.00106834171486 0.00407585042731 0.00107662710674 0.430656612427 0.430711322784 0.070435 0.0720766407415 0.00827 0.00834492350487 0.0465 Chr1 122177661

N00032 4200000 4400000 5.156364 scaffold_34 1526847 1729045 0.00481371498377 0.00123033016004 0.00486695570032 0.00124869760137 0.424853091811 0.424614257081 0.08354 0.0601390716031 0.01667 0.0165234077488 NA Chr1 122177661

N00032 4400000 4600000 3.771072 scaffold_34 1333436 1526847 0.00457213523796 0.0012898930076 0.00462033383802 0.00131132388506 0.408159384299 0.410367349645 0.070895 0.0431671414759 0.024395 0.0238455930635 0.0605 Chr1 122177661

N00032 4600000 4800000 0 scaffold_34 1146578 1332612 0.00416035148712 0.000934480767616 0.00424961792149 0.000961464665534 0.401854074652 0.403588164265 0.06745 0.0318973950998 0.029755 0.0316877560016 0.0585 Chr1 122177661

N00032 5000000 5200000 0 scaffold_34 797235 988499 0.00433395625327 0.00112528178569 0.00439500214791 0.00115063284429 0.422311841976 0.422740042968 0.103025 0.0637234398528 0.019765 0.0218650660867 0.0615 Chr1 122177661

N00032 5200000 5400000 0 scaffold_34 597615 797235 0.00452594001545 0.00124723368622 0.00456547653644 0.00125533924379 0.422456788349 0.421978191601 0.08158 0.0750826570484 0.01146 0.00940787496243 0.0415 Chr1 122177661

N00032 5400000 5600000 0 scaffold_34 399270 597615 0.00418296482437 0.00108979223274 0.00424940702919 0.00111091408707 0.400687924351 0.399965294882 0.08901 0.0690009831354 0.01826 0.0198542942852 0.032 Chr1 122177661

N00032 5600000 5800000 0 scaffold_34 202552 399270 0.00484093950939 0.00112427479423 0.00492095793948 0.00114625801537 0.393510334912 0.392098284908 0.096095 0.0770646305879 0.023315 0.0218434510314 0.048 Chr1 122177661

N00032 5800000 6000000 1.43459 scaffold_34 19827 202552 0.00468742329438 0.000842354696281 0.00471007863214 0.000850192089806 0.399121086992 0.397429958085 0.07185 0.0406293610617 0.005895 0.0104255028048 0.033 Chr1 122177661

N00032 6200000 6400000 1.948328 scaffold_35 174424 374001 0.00494382706296 0.000846520130719 0.00504319980262 0.000859634777692 0.404155912765 0.401190174169 0.06289 0.0502663132525 0.03049 0.0317972511863 0.0666666666667 Chr1 122177661

N00032 6400000 6600000 1.948328 scaffold_35 374001 569984 0.00487190174528 0.00115071032054 0.00491424849688 0.00116117704555 0.380442792253 0.376997381469 0.07324 0.053320951307 0.012205 0.0125470066281 0.0616666666667 Chr1 122177661

N00032 6600000 6800000 1.948328 scaffold_35 569984 772397 0.00476795689072 0.00116939140647 0.00504297234514 0.00124317827614 0.361861978621 0.362993556716 0.08378 0.0912787222165 0.071365 0.0705043648382 0.0635 Chr1 122177661

N00032 6800000 7000000 1.948328 scaffold_35 772397 962855 0.00458377822287 0.000906428241309 0.00458377822287 0.000906428241309 0.394543432398 0.396621783125 0.121415 0.0841445358032 0 0 NA Chr1 122177661

N00032 7000000 7200000 1.946851 scaffold_35 962855 1156504 0.00466940240108 0.00110374075681 0.00466940240108 0.00110374075681 0.395001514037 0.39500089969 0.055205 0.0449937774014 0 0 NA Chr1 122177661

N00032 7200000 7400000 1.939048 scaffold_35 1156504 1347835 0.00425883612818 0.000940369421684 0.00426941062326 0.000942521876838 0.401178898403 0.403880404292 0.09852 0.0599536928151 0.00312 0.00327704344827 0.01 Chr1 122177661

N00032 7400000 7600000 1.939048 scaffold_35 1347835 1540205 0.00421637073258 0.00113324769321 0.00428395504333 0.00115771336313 0.391942316864 0.389504660777 0.0746 0.0468680147632 0.022605 0.0219005042366 0.084 Chr1 122177661

N00032 7600000 7800000 0.9331066 scaffold_35 1540205 1733631 0.00358029824117 0.000984664404594 0.00368773420784 0.00101359021784 0.398388691757 0.398652502697 0.075355 0.0586322417876 0.038965 0.0399997932026 0.0425 Chr1 122177661

N00032 8000000 8200000 0.04938272 scaffold_35 1934780 2124565 0.00425578889468 0.0010256284149 0.00435037372588 0.00105243479054 0.379160619328 0.379677991 0.10067 0.0744421318861 0.03241 0.035345259109 0.06 Chr1 122177661

N00032 8200000 8400000 0.04938272 scaffold_35 2124565 2321310 0.00429049111374 0.00115158141246 0.00447066929523 0.00120699246345 0.377297080176 0.37938990157 0.113515 0.0795242572874 0.056395 0.0571043736817 0.035 Chr1 122177661

N00032 8400000 8600000 0.3254079 scaffold_35 2321310 2523641 0.00427133539888 0.00111911991743 0.00430957232016 0.00113741012917 0.423474131633 0.422600505008 0.10426 0.0729843672003 0.01451 0.0151336176859 0.068 Chr1 122177661

N00181 200000 400000 1.440556 scaffold_35 2905019 3099534 0.00441393943386 0.00124239642225 0.00443748571596 0.00125223352919 0.387341372526 0.385094088013 0.08199 0.0808626584068 0.007025 0.00720767035961 0.0685 Chr1 122177661

N00181 400000 600000 1.440556 scaffold_35 3099534 3292968 0.00465811540989 0.00116505086527 0.00494100962829 0.00129168524441 0.375769428058 0.383304503029 0.098855 0.065996670699 0.082965 0.11137132045 0.0853333333333 Chr1 122177661

N00181 800000 1000000 1.440556 scaffold_35 3496323 3699988 0.00454783423448 0.00130147316079 0.004598641455 0.00132684683028 0.395058622251 0.393878662203 0.104535 0.0859008666192 0.02303 0.0272948223799 0.079 Chr1 122177661

N00181 1000000 1200000 1.440556 scaffold_35 3699988 3895080 0.0043095466278 0.000976161574153 0.00434843195618 0.000985184399456 0.391228599571 0.39133238818 0.090265 0.0761076825293 0.010175 0.0119994669182 0.06 Chr1 122177661

N00050 0 200000 1.28934 scaffold_37 7384888 7575622 0.0047302707659 0.00163709211768 0.0047302707659 0.00163709211768 0.377323821246 0.376901267606 0.119285 0.101198527793 0 0 NA Chr1 122177661

N00050 200000 400000 1.28934 scaffold_37 7575622 7770147 0.00471681781621 0.00159770632748 0.00471681781621 0.00159770632748 0.387277895815 0.386806185885 0.124 0.114720472947 0 0 NA Chr1 122177661

N00050 400000 600000 1.28934 scaffold_37 7770147 7964966 0.00415963672779 0.00153687688286 0.00415963672779 0.00153687688286 0.40952773279 0.412945922167 0.09621 0.078842412701 0 0 NA Chr1 122177661

N00050 600000 800000 1.28934 scaffold_37 7964966 8160589 0.00450512375672 0.00135235191855 0.00450838843331 0.0013731392542 0.41183100171 0.414086402153 0.061655 0.0679316849246 0.001885 0.0187707989347 NA Chr1 122177661

N00050 800000 1000000 1.694796 scaffold_37 8160589 8357850 0.00401549159066 0.00135538298989 0.00413340031398 0.00140945938407 0.397921714002 0.398522656663 0.085405 0.0797319287644 0.05053 0.0519007811985 NA Chr1 122177661

N00050 1400000 1600000 1.711899 scaffold_35 8567244 8758011 0.00448410014435 0.00119800580019 0.0045804128574 0.00126623498386 0.402287258102 0.402019887053 0.090925 0.0581494702962 0.042515 0.0662535973203 0.07 Chr1 122177661

N00050 1600000 1800000 0.311253 scaffold_35 8378172 8567244 0.00429563905188 0.00123416669649 0.00441141358041 0.00127767709703 0.424324111008 0.426400576217 0.09677 0.0646367521368 0.04747 0.0482197258187 0.086 Chr1 122177661

N00050 1800000 2000000 1.489568 scaffold_35 8181232 8377969 0.00406317694293 0.00123298579533 0.0041336466231 0.00126359553337 0.438859534756 0.439667790109 0.075595 0.0681214006516 0.03263 0.0303755775477 0.0535 Chr1 122177661

N00050 2000000 2200000 1.489568 scaffold_35 7984975 8181232 0.00425088049392 0.0011667515601 0.0043140003128 0.00118774307636 0.424858317591 0.428387383385 0.07644 0.0622092460396 0.020575 0.0208960699491 0.0415 Chr1 122177661

N00050 2200000 2400000 1.489568 scaffold_35 7791267 7984011 0.00342863271662 0.000979490919552 0.00344128777005 0.000990589349356 0.434385588653 0.432167235495 0.083745 0.0434929232557 0.021655 0.0144284646993 NA Chr1 122177661

N00050 2400000 2600000 1.489568 scaffold_35 7602830 7791267 0.0041653108567 0.00106021375446 0.00418953093347 0.00106844454395 0.403071515352 0.404701099404 0.11977 0.0822184602812 0.007185 0.00757812956054 0.174 Chr1 122177661

N00050 2600000 2800000 1.489568 scaffold_35 7410259 7602830 0.00493400803219 0.00133493884551 0.00493400803219 0.00133493884551 0.380792966237 0.376437219972 0.115005 0.118636762545 0 0 NA Chr1 122177661

N00050 3400000 3600000 1.590788 scaffold_35 6649532 6843782 0.00511466811113 0.0015746531576 0.00511466811113 0.0015746531576 0.349769487116 0.348022839268 0.13039 0.10557014157 0 0 NA Chr1 122177661

N00050 3600000 3800000 1.597781 scaffold_35 6451805 6649532 0.00523159457035 0.00165480225556 0.00523159457035 0.00165910244734 0.348561367743 0.347493699718 0.113025 0.116256252307 0 0.00290299250987 NA Chr1 122177661

N00050 3800000 4000000 1.597781 scaffold_35 6253796 6451805 0.00505708892833 0.00157015336147 0.00510360594602 0.00158956912353 0.353183910081 0.351565647522 0.089315 0.0725017549707 0.013825 0.0137670509926 0.07 Chr1 122177661

N00050 4000000 4200000 1.597781 scaffold_35 6062624 6253796 0.00496749060056 0.0011284382246 0.00497068366049 0.00113057773387 0.364856476106 0.365019328643 0.09263 0.0745558973071 0.002175 0.00246374992154 0.056 Chr1 122177661

N00050 4200000 4400000 0.2305256 scaffold_35 5868666 6062624 0.00493752921544 0.00116601884841 0.00503330019991 0.00119765096243 0.371312650268 0.371673907721 0.086 0.0665917363553 0.02966 0.0311974757422 0.0435 Chr1 122177661

N00050 4400000 4600000 0 scaffold_35 5670272 5868666 0.00466988238167 0.00113555558285 0.00472559373135 0.0011585894005 0.371902370532 0.372486296557 0.08904 0.0780719174975 0.02113 0.0220016734377 0.1035 Chr1 122177661

N00050 4600000 4800000 0 scaffold_35 5475988 5670206 0.00407101031493 0.00103572236917 0.004143920038 0.00105566938583 0.384411008585 0.38286831573 0.090555 0.0602003933724 0.020955 0.0247144960817 0.0846666666667 Chr1 122177661

N00050 4800000 5000000 0 scaffold_35 5282810 5475988 0.00476360841077 0.00123024084013 0.0048720003672 0.00126229889772 0.387327830026 0.388092928319 0.06487 0.0564453509199 0.04311 0.0430587333961 0.1168 Chr1 122177661

N00050 5200000 5400000 3.463918 scaffold_35 4883119 5088438 0.00444712943194 0.0011668726449 0.00446097218341 0.00117134376566 0.393259917739 0.392488164241 0.090855 0.112464019404 0.004975 0.0050652886484 0.065 Chr1 122177661

N00050 5600000 5800000 1.792883 scaffold_35 4481820 4685476 0.00501800077697 0.00134181401195 0.00501800077697 0.00134181401195 0.36809891325 0.370161397597 0.097135 0.118076560475 0 0 NA Chr1 122177661

N00050 5800000 6000000 1.440556 scaffold_35 4282881 4481820 0.00487889406629 0.00124072275559 0.00488348208576 0.00124171223785 0.37085243412 0.372529705104 0.102965 0.0988896093777 0.001235 0.001241586617 0.074 Chr1 122177661

N00050 6000000 6200000 1.440556 scaffold_35 4093475 4282881 0.00432393259678 0.00107372076666 0.00439910041456 0.00109166883973 0.382818966185 0.386943718209 0.10347 0.0890943264733 0.0187 0.0198462561904 0.0605 Chr1 122177661

N00043 200000 400000 2.601018 scaffold_37 792773 986377 0.00401656537697 0.000940294827941 0.00412323810286 0.000993950488569 0.445826979891 0.446535938374 0.083015 0.0430724571806 0.0773 0.0805458564906 0.0713333333333 Chr1 122177661

N00043 400000 600000 4.359293 scaffold_37 986377 1176587 0.00427418147254 0.00100003431122 0.0045033705863 0.00107519915042 0.437386546672 0.440735008721 0.064095 0.0439777088481 0.11085 0.113979286052 0.102 Chr1 122177661

N00043 600000 800000 4.930199 scaffold_37 1177234 1379054 0.00464607745919 0.00133507053647 0.00478899540372 0.0013873224469 0.425169508455 0.432536780836 0.09769 0.106674264196 0.04524 0.0450252700426 0.0955 Chr1 122177661

N00043 1000000 1200000 4.937159 scaffold_37 1560282 1746809 0.00426923461164 0.00147665949007 0.00442983502297 0.00154370588919 0.453452699863 0.461149785287 0.10466 0.0817039892348 0.05141 0.0549839969549 0.09325 Chr1 122177661

N00043 1600000 1800000 3.965 scaffold_37 2121527 2314804 0.00392357006184 0.00113379648163 0.00395147013749 0.0011548530879 0.440329433372 0.440989164095 0.097425 0.0768948193525 0.0161 0.0240328647485 0.071 Chr1 122177661

N00043 1800000 2000000 3.532468 scaffold_37 2314804 2506935 0.00386150033118 0.00122698667509 0.00390951398943 0.0012502241347 0.403965933647 0.400141888344 0.08195 0.0752923786375 0.02282 0.0238951548683 0.071 Chr1 122177661

N00043 2000000 2200000 1.943166 scaffold_37 2506935 2694524 0.00399359589982 0.00105525790069 0.0039955336423 0.00105646628237 0.406202249296 0.40308116938 0.10981 0.0832831349386 0.002465 0.00262808586857 0.071 Chr1 122177661

N00043 2200000 2400000 1.943166 scaffold_37 2694673 2884666 0.00382414973344 0.00124105557327 0.00382414973344 0.00124105557327 0.397581903729 0.395442276505 0.108265 0.0918560157479 0 0 NA Chr1 122177661

N00043 2400000 2600000 1.943166 scaffold_37 2884666 3081934 0.00421129594043 0.00125620555844 0.00421129594043 0.00125620555844 0.382237817306 0.383544093844 0.12563 0.124100208853 0 0 NA Chr1 122177661

N00043 3600000 3800000 5.379009 scaffold_37 4060383 4260985 0.00513219459738 0.00144418536187 0.00513219459738 0.00144418536187 0.385217471732 0.381251689646 0.11201 0.124719594022 0 0 NA Chr1 122177661

N00043 3800000 4000000 3.289413 scaffold_37 4260985 4458080 0.00412238508684 0.00101670686773 0.00421791972586 0.00104134112876 0.394375592065 0.393766984166 0.067565 0.0527106217814 0.02545 0.0256475303788 0.05 Chr1 122177661

N00043 4000000 4200000 1.178922 scaffold_37 4458080 4652821 0.00443972049133 0.00122529490274 0.00448625214074 0.0012690109429 0.393214646022 0.393206792045 0.11678 0.098243307778 0.022715 0.0438787928582 0.148 Chr1 122177661

N00043 4200000 4400000 1.178922 scaffold_37 4652821 4841049 0.00430121368112 0.0010690015419 0.00430121368112 0.0010702544781 0.388308806354 0.390362013014 0.10745 0.0885840576322 0 0.00166818964235 NA Chr1 122177661

N00043 4400000 4600000 1.178922 scaffold_37 4841049 5041393 0.00435582911015 0.00108374391935 0.00436069864875 0.00108535927251 0.382835231 0.384264525605 0.09283 0.10843349439 0.001545 0.00121291378828 0.053 Chr1 122177661

N00043 4600000 4800000 3.483023 scaffold_37 5041393 5237457 0.0045098352294 0.00113444439084 0.00451842226136 0.00113575408421 0.369048437357 0.368997014462 0.09692 0.10519524237 0.002 0.00204015015505 0.053 Chr1 122177661

N00043 4800000 5000000 6.692308 scaffold_37 5237745 5436729 0.00432009847063 0.00120995169847 0.00433766468536 0.00121493834452 0.375245393299 0.375666644899 0.076505 0.0841072649057 0.004545 0.00456820648896 0.053 Chr1 122177661

N00043 5000000 5200000 2.264064 scaffold_37 5436729 5637848 0.00415128230967 0.00112477063873 0.00420622617767 0.00114204474483 0.402933836234 0.40019320724 0.09812 0.0929051954316 0.016695 0.0155877863355 0.0826666666667 Chr1 122177661

N00043 5200000 5400000 5.809746 scaffold_37 5637848 5835527 0.00417356966262 0.00107033129936 0.0043059308808 0.00111706633286 0.42794107181 0.429156956659 0.094775 0.0677967816511 0.048135 0.0545986169497 0.09625 Chr1 122177661

N00043 5400000 5600000 4.565567 scaffold_37 5835527 6023989 0.00440148820944 0.00112167626254 0.00442088653905 0.00112904256329 0.416930661299 0.417064062704 0.110045 0.0724655368191 0.00943 0.0104477295158 0.081 Chr1 122177661

N00043 5600000 5800000 1.715543 scaffold_37 6023989 6223872 0.00445910152498 0.00124012735773 0.00447650933609 0.00124764752913 0.392404922385 0.393844831244 0.092505 0.0818428780837 0.00983 0.0089102124743 0.0435 Chr1 122177661

N00043 5800000 6000000 1.715543 scaffold_37 6223872 6428735 0.00462709089703 0.00120547123431 0.00464981689575 0.00120819122865 0.392569049896 0.392799016651 0.116545 0.128012379005 0.00432 0.00423697788278 0.044 Chr1 122177661

N00043 6200000 6400000 1.715543 scaffold_37 6615826 6805070 0.00546671597199 0.00143408388912 0.00550953973642 0.00144675039839 0.368781757912 0.369113798518 0.082545 0.0682135232821 0.009845 0.0104045570797 0.075 Chr1 122177661

N00043 6400000 6600000 1.357071 scaffold_37 6805070 6999395 0.0044974132045 0.00118110212047 0.0045551073825 0.00119382356849 0.392973397063 0.393089261877 0.07127 0.0599768429178 0.01474 0.0151961919465 0.100666666667 Chr1 122177661

N00043 6600000 6800000 2.601337 scaffold_37 6999395 7194742 0.0047113686989 0.00146569294943 0.0048145709376 0.00150139106524 0.381042153684 0.378580381064 0.082815 0.0817826739085 0.031885 0.032864594798 0.071 Chr1 122177661

N00089 400000 600000 2.267925 scaffold_23 13707549 13894641 0.00372302403145 0.00102585654886 0.00380003206641 0.00107228221108 0.423792554884 0.427660671499 0.11618 0.088095696235 0.053565 0.0559617728177 0.0735 Chr1 122177661

N00089 1000000 1200000 1.719955 scaffold_23 13164792 13352603 0.00305365239996 0.000946078666373 0.00310207167672 0.00097962578306 0.474216526913 0.480226093517 0.140255 0.0497840914536 0.052575 0.0563758246322 0.0924285714286 Chr1 122177661

N00089 1200000 1400000 5.216418 scaffold_23 12973154 13164792 0.00354035685775 0.00103674020854 0.00367317465204 0.00110219576841 0.441368826212 0.447706571243 0.117155 0.0490873417589 0.08435 0.0905822436051 0.079375 Chr1 122177661

N00089 1600000 1800000 0.6473407 scaffold_23 12600084 12788995 0.0043056763948 0.00111299020252 0.00434476510014 0.00112834604711 0.396000764849 0.392681850923 0.08481 0.0653852872517 0.019805 0.0208193276199 0.0693333333333 Chr1 122177661

N00089 1800000 2000000 1.445727 scaffold_23 12411206 12600084 0.00376735923502 0.00115341933924 0.00378755856543 0.00116171362478 0.416295611271 0.412233390537 0.09625 0.0400946642806 0.00637 0.00588739821472 0.039 Chr1 122177661

N00089 2000000 2200000 1.445727 scaffold_23 12225319 12411206 0.00346963120615 0.00100370265281 0.00347683140535 0.00100603061904 0.423288160182 0.416088111037 0.113695 0.0569109189992 0.00693 0.00863965742629 0.039 Chr1 122177661

N00089 2200000 2400000 1.445727 scaffold_23 12040802 12225319 0.00381782812893 0.00111395684251 0.00393760326909 0.0011589948663 0.402606150417 0.396522094926 0.091295 0.052580521036 0.053555 0.0569378431256 0.071 Chr1 122177661

N00089 2400000 2600000 1.445727 scaffold_23 11846041 12040802 0.00373533301266 0.00101631484226 0.0038546044936 0.00108214735637 0.429134189761 0.420180130809 0.082035 0.0401877172535 0.07857 0.0814074686411 0.09025 Chr1 122177661

N00089 2800000 3000000 1.445727 scaffold_23 6671246 6857748 0.00285848230397 0.00110230223068 0.00289104786757 0.0011136939098 0.378460065409 0.36897768012 0.10848 0.0533988911647 0.01125 0.0121017468982 0.069 Chr1 122177661

N00089 3000000 3200000 1.992325 scaffold_23 6857748 7054134 0.00236567387542 0.00103310648368 0.00239165992514 0.00104822714847 0.445178821398 0.43502024752 0.099875 0.0448962757019 0.021 0.0206684794232 0.0665 Chr1 122177661

N00073 200000 400000 0.2865854 scaffold_23 11000180 11184924 0.000297474193905 0.00111506586096 0.000297466453168 0.00112323486414 0.432485908202 0.427346368873 0.10125 0.0422097605335 0.00603 0.00913155501667 0.0613333333333 Chr1 122177661

N00073 400000 600000 0.2865854 scaffold_23 10797235 11000180 0.000548666376656 0.00123557943543 0.000567317097368 0.00131061641481 0.397474607284 0.394952963148 0.05799 0.0394983862623 0.07987 0.0781886718076 0.103428571429 Chr1 122177661

N00073 800000 1000000 0 scaffold_23 10421306 10614304 0.00146985809422 0.00127983283317 0.00150572590422 0.00131836528404 0.444866698869 0.438885973159 0.070945 0.0319951502088 0.06207 0.0650991201981 0.0864 Chr1 122177661

N00073 1000000 1200000 0 scaffold_23 10234485 10421306 0.00226101601049 0.0012005599657 0.00230088513993 0.00123128692069 0.431751522445 0.430932894996 0.0903 0.0470825014319 0.04581 0.0488274872739 0.0467142857143 Chr1 122177661

N00073 1200000 1400000 0 scaffold_23 10039668 10234485 0.00199148791612 0.000960674931388 0.00199310376807 0.000962876273807 0.407023032526 0.403856638172 0.073605 0.0348429551836 0.01002 0.0103019757003 0.043 Chr1 122177661

N00073 1400000 1600000 0.6623903 scaffold_23 9848709 10039668 0.00248589451511 0.00103898779672 0.00248589451511 0.00103898779672 0.392968593122 0.388380209624 0.07166 0.0350860655952 0 0 NA Chr1 122177661

N00073 1600000 1800000 0.875924 scaffold_23 9660523 9848709 0.00301876648071 0.00104008626072 0.00301876648071 0.00104008626072 0.418204570308 0.413789143251 0.08562 0.0512152870033 0 0 NA Chr1 122177661

N00073 1800000 2000000 0.875924 scaffold_23 9471492 9660523 0.00293799403368 0.00123544653059 0.00294345214311 0.0012383348788 0.456256408177 0.450419075224 0.08571 0.0578582348927 0.006945 0.00708878437928 0.064 Chr1 122177661

N00073 2000000 2200000 0.875924 scaffold_23 9285021 9471492 0.00310949968155 0.000961414928771 0.00311303738515 0.000963747811209 0.449882889229 0.443443182386 0.06849 0.0599986056813 0.001795 0.00192523234176 0.067 Chr1 122177661

N00073 2200000 2400000 0.875924 scaffold_23 9091554 9285021 0.00309230441705 0.00109951192605 0.00309521656178 0.00110083759972 0.438697887425 0.431596487716 0.07498 0.0494761380494 0.000775 0.000801170225413 0.067 Chr1 122177661

N00073 2400000 2600000 0.875924 scaffold_23 8899785 9091554 0.0032834307525 0.00112867365478 0.0032834307525 0.00112867365478 0.418121731994 0.410645783555 0.08751 0.0595612429538 0 0 NA Chr1 122177661

N00073 2600000 2800000 0.875924 scaffold_23 8705674 8899756 0.00359338858507 0.00119057993367 0.00359338858507 0.00119057993367 0.437793053466 0.427542555424 0.09401 0.0612112406096 0 0 NA Chr1 122177661

N00073 2800000 3000000 0.875924 scaffold_23 8516826 8705671 0.00320623341353 0.00107980641336 0.00320623341353 0.00107980641336 0.438503816966 0.430672644767 0.07749 0.0561253938415 0 0.000354788318462 NA Chr1 122177661

N00073 3000000 3200000 0.875924 scaffold_23 8336822 8516826 0.00328370379352 0.00111508320361 0.00328370379352 0.00111508320361 0.435937545771 0.427140870027 0.109955 0.0512155285438 0 0 NA Chr1 122177661

N00073 3200000 3400000 1.845562 scaffold_23 8146013 8336822 0.00356298626684 0.00106632107191 0.00357673110921 0.00106997108408 0.446580020763 0.430498351973 0.106095 0.0696141167345 0.006465 0.00656677620028 0.054 Chr1 122177661

N00073 3400000 3600000 3.819839 scaffold_23 7953491 8146013 0.00337404191097 0.00112755574745 0.00340236566447 0.00113684384206 0.454432588417 0.442810177639 0.070775 0.0346661680224 0.0243 0.0290148658335 0.109 Chr1 122177661

N00073 3600000 3800000 4.146882 scaffold_23 7765592 7953491 0.00367554588699 0.00113617448965 0.00375337982644 0.00117997583723 0.445441319108 0.43383519643 0.117505 0.0840664399491 0.04428 0.046524994811 0.0526666666667 Chr1 122177661

N00205 0 200000 0.7263682 scaffold_23 11357807 11555998 0.00335276484376 0.0010264079876 0.00345122722783 0.00106221166026 0.414069367504 0.414374690936 0.093835 0.0707196593185 0.058355 0.0554818331811 0.084375 Chr1 122177661

N00205 200000 400000 0.7263682 scaffold_23 11555998 11736283 0.00311282328935 0.00117431380417 0.00311282328935 0.00117431380417 0.392488700977 0.384904350202 0.12152 0.0472696009097 0 0 NA Chr1 122177661

N00205 600000 800000 2.288103 scaffold_23 6205249 6398947 0.00399840039222 0.00103296272357 0.00406365793101 0.001060345436 0.383473986831 0.379528907923 0.02711 0.0169852037708 0.0284 0.0306249935467 0.059 Chr1 122177661

N00205 800000 1000000 1.907426 scaffold_23 6006827 6205249 0.00408840559866 0.00125343940064 0.00418788061062 0.0012962545332 0.39657618895 0.392703838547 0.076965 0.0629718478798 0.03414 0.0487193960347 0.0982 Chr1 122177661

N00155 0 200000 1.634569 scaffold_23 5708073 5909455 0.00428940304034 0.00119197870321 0.00448963017364 0.00124788369678 0.386494590612 0.385893731202 0.078225 0.0530831951217 0.069695 0.0686406928127 0.062125 Chr1 122177661

N00155 200000 400000 3.491735 scaffold_23 5507103 5708073 0.00429234634647 0.00117859124464 0.0043655278731 0.00121153125755 0.425457847737 0.425865302454 0.068845 0.0711101159377 0.030045 0.0304821615166 0.0473333333333 Chr1 122177661

N00155 400000 600000 4.416108 scaffold_23 5315416 5507103 0.00425191006822 0.00134111093151 0.00428180028683 0.00134991782954 0.40620110698 0.408485487541 0.092455 0.0578912497978 0.0063 0.00651061365664 0.057 Chr1 122177661

N00155 600000 800000 4.080936 scaffold_23 5122141 5315416 0.00461985659599 0.00139023366685 0.00461985659599 0.00139023366685 0.389154759659 0.388848987409 0.081025 0.0950873108265 0 0 NA Chr1 122177661

N00155 800000 1000000 2.672646 scaffold_23 4924613 5122141 0.00441929633373 0.00136975726922 0.00441929633373 0.00136975726922 0.386928356248 0.386734888095 0.106795 0.103453687578 0 0 NA Chr1 122177661

N00155 1000000 1200000 3.079277 scaffold_23 4728413 4924613 0.0041131068406 0.00136225584891 0.0041131799133 0.00136225584891 0.399301728304 0.400160895833 0.07219 0.0548878695209 0.00505 0 NA Chr1 122177661

N00155 1200000 1400000 5.646617 scaffold_23 4529480 4728413 0.00393554677758 0.00129075691537 0.00393554677758 0.00129075691537 0.393761994614 0.396478534309 0.07534 0.0565567301554 0 0 NA Chr1 122177661

N00155 1400000 1600000 5.75684 scaffold_23 4337566 4529480 0.0044777382356 0.00104605603409 0.00454776615597 0.00106649794676 0.382393572344 0.383006686271 0.06726 0.0354585908271 0.0206 0.0215773731984 0.0705 Chr1 122177661

N00104 1000000 1200000 3.97732 scaffold_23 2342230 2541724 0.00506442688954 0.00104856563341 0.00526443314421 0.00109076452136 0.398788356956 0.401298529825 0.08215 0.0564678636951 0.046805 0.045460013835 0.06925 Chr1 122177661

N00104 1200000 1400000 11.0246 scaffold_23 2541724 2733187 0.00428514963952 0.00108961207343 0.00431840416106 0.00109955958854 0.41996608634 0.427152411802 0.105645 0.0797960963737 0.012865 0.0130364613528 0.116 Chr1 122177661

N00104 1400000 1600000 3.174141 scaffold_23 2733764 2929329 0.00425844978309 0.00113578794299 0.0042877538557 0.00114384178435 0.383039019756 0.388115553978 0.062585 0.0476874696393 0.007435 0.00714851839542 NA Chr1 122177661

N00104 1600000 1800000 3.015484 scaffold_23 2929329 3126381 0.00490119748801 0.00104768035852 0.00490119748801 0.00104768035852 0.374171386234 0.374502372329 0.105695 0.0922548362869 0 0 NA Chr1 122177661

N00104 1800000 2000000 2.102484 scaffold_23 3126381 3333971 0.00489404948085 0.00117649298323 0.00489404948085 0.00117649298323 0.389450127877 0.390956479573 0.051335 0.0626523435618 0 0 NA Chr1 122177661

N00104 2000000 2200000 1.579908 scaffold_23 3333971 3541392 0.00410116707631 0.0012242410212 0.00416275165159 0.00125545091165 0.392314899477 0.387813640511 0.090945 0.102241335255 0.02459 0.0314288331461 0.170333333333 Chr1 122177661

N00104 2200000 2400000 5.182882 scaffold_23 3541392 3738585 0.00385808584195 0.00097219420701 0.00400656840822 0.00102525828177 0.415374829725 0.413414423404 0.04657 0.0329575593454 0.056445 0.0563204576227 0.1405 Chr1 122177661

N00104 2400000 2600000 4.089554 scaffold_23 3738585 3927175 0.00429319392912 0.00119481704494 0.00431042075508 0.00120066256315 0.412522535413 0.412472388766 0.085345 0.0501087014158 0.00418 0.00462908955936 NA Chr1 122177661

N00104 2600000 2800000 3.251916 scaffold_23 3927175 4120445 0.00431606589999 0.00133122842561 0.00431606589999 0.00133122842561 0.396391371102 0.394865291159 0.057065 0.0572670357531 0 0 NA Chr1 122177661

N00041 2000000 2200000 0.8280396 scaffold_97 884721 1088378 0.00536839186411 0.000899431222823 0.0054265168717 0.000909555502342 0.347100627713 0.344926464823 0.08504 0.100084946749 0.01243 0.012506321904 0.084 Chr1 122177661

N00041 3200000 3400000 0.09640685 scaffold_30 10093337 10305332 0.00530737837414 0.00107319801988 0.00532048182112 0.00107952520416 0.363839059491 0.360888538529 0.10307 0.151022429774 0.00839 0.00732092738036 NA Chr1 122177661

N00041 3400000 3600000 1.211111 scaffold_30 9896807 10093337 0.00468575407643 0.0010919789821 0.00472316012891 0.00110378343099 0.384577398589 0.385800148106 0.115795 0.122627588663 0.01185 0.0125426143591 NA Chr1 122177661

N00041 3800000 4000000 1.600358 scaffold_30 9506937 9705654 0.00431170075253 0.00108308614691 0.00431339744935 0.00108321371483 0.383445020212 0.384041936108 0.06819 0.0712520821067 0.00038 0.00596325427618 NA Chr1 122177661

N00041 4000000 4200000 0.8431877 scaffold_30 9308954 9506937 0.00448305936485 0.0010053026302 0.00448923428285 0.00100646999686 0.373513050456 0.37081451912 0.076755 0.0650156831647 0.001825 0.00237899213569 NA Chr1 122177661

N00041 4200000 4400000 0.988059 scaffold_30 9120403 9308954 0.00452408974031 0.00124808465294 0.0045626771446 0.00126423210599 0.382180391075 0.37880350837 0.06987 0.0594162852491 0.0096 0.0175814501116 0.036 Chr1 122177661

N00041 4400000 4600000 2.601449 scaffold_30 8932329 9120403 0.00392057425403 0.00107331159071 0.00392057425403 0.00107331159071 0.405963200322 0.406015528508 0.081035 0.0403989918862 0 0 NA Chr1 122177661

N00041 4600000 4800000 1.101392 scaffold_30 8736033 8932329 0.00373097997225 0.00108945733712 0.0037493452123 0.0011029483003 0.40068119891 0.402692031393 0.056875 0.0391449647471 0.017425 0.0177741777723 0.088 Chr1 122177661

N00041 4800000 5000000 1.113022 scaffold_30 8517149 8735987 0.00417944461976 0.0011396023161 0.00420617311924 0.00115740791273 0.392366261248 0.392998122629 0.0713 0.0521207468538 0.020135 0.0218791983111 0.126 Chr1 122177661

N00041 5000000 5200000 0.9617624 scaffold_30 8313930 8517149 0.00514506542095 0.00190123760944 0.00514677961746 0.00190309690385 0.375615952576 0.375411536314 0.059035 0.0602109054764 0.000825 0.00111702153834 NA Chr1 122177661

N00041 5200000 5400000 0.55 scaffold_30 8123645 8313930 0.00469249217747 0.00162376373006 0.00469249217747 0.00162376373006 0.375194418772 0.373158126237 0.07671 0.0666999500749 0 0 NA Chr1 122177661

N00041 5400000 5600000 0.55 scaffold_30 7930292 8123645 0.00411612922966 0.00134618316436 0.00411612922966 0.00134618316436 0.368340071694 0.367767356437 0.082215 0.0521015965617 0 0 NA Chr1 122177661

N00041 5600000 5800000 0.6308219 scaffold_30 7738852 7930292 0.00490188582265 0.00155325770932 0.00490405283082 0.00155298747695 0.378404507117 0.382319080717 0.06847 0.0500731299624 0.00205 0.00199540325951 NA Chr1 122177661

N00041 5800000 6000000 2.346274 scaffold_30 7537747 7738852 0.00442862352775 0.00153742710163 0.00445902200259 0.00154783219711 0.380007242232 0.380289220028 0.036295 0.0408343899953 0.009755 0.0105765644812 NA Chr1 122177661

N00041 6000000 6200000 1.567475 scaffold_30 7350931 7537747 0.00430724586811 0.00140255205861 0.004328655477 0.00141452896936 0.386928320454 0.384859753905 0.057655 0.0482988609113 0.010585 0.0118619390202 NA Chr1 122177661

N00041 6200000 6400000 1.928322 scaffold_30 7162379 7350931 0.00410889740445 0.00151336958021 0.00414233167677 0.00152649244444 0.381472681436 0.380739768702 0.0519 0.0359794645509 0.01075 0.0114186007043 0.099 Chr1 122177661

N00041 6400000 6600000 2.350195 scaffold_30 6971751 7162379 0.0045554305002 0.00138116089751 0.0045986300654 0.00139144057232 0.37684588756 0.375834673426 0.04994 0.0301005098936 0.010445 0.0121807919088 0.099 Chr1 122177661

N00041 6600000 6800000 0.4793929 scaffold_30 6774013 6971751 0.00470594101733 0.00131212874019 0.00470594101733 0.00131212629903 0.375820866313 0.376851781919 0.07315 0.0541929219472 0 0.00207850792463 0.099 Chr1 122177661

N00041 6800000 7000000 0 scaffold_30 6582522 6774013 0.00540172603462 0.00175558449294 0.00540172603462 0.00175639190656 0.370082023396 0.370579126996 0.0649 0.0832571765775 0 0.00102876897609 0.099 Chr1 122177661

N00041 7000000 7200000 1.888621 scaffold_30 6395292 6582516 0.00494449321341 0.00180013061966 0.00494449321341 0.00180013061966 0.368778010667 0.370667271806 0.049815 0.0497105071999 0 0 NA Chr1 122177661

N00284 0 200000 0.7386439 scaffold_30 5762981 5961545 0.00480022719829 0.00128473804208 0.00483548638043 0.00129607987861 0.371108425794 0.364530135661 0.09217 0.0643167945851 0.00986 0.0100924638907 0.063 Chr1 122177661

N00284 200000 400000 1.872038 scaffold_30 5961545 6156453 0.00469804157269 0.00158231030493 0.00469804157269 0.00158231030493 0.373223105482 0.369394301528 0.123765 0.0791450325282 0 0 NA Chr1 122177661

N00055 0 200000 0.2257463 scaffold_30 5389224 5580311 0.00433520968799 0.00163618693872 0.00433520968799 0.00163618693872 0.372913050031 0.370027077129 0.074525 0.0624741609843 0 0 NA Chr1 122177661

N00055 200000 400000 2.238504 scaffold_30 5198560 5389224 0.00486790057588 0.00146194749821 0.00486790057588 0.00146194749821 0.364214845125 0.358776682197 0.071605 0.0463642848152 0 0 NA Chr1 122177661

N00055 400000 600000 0.353204 scaffold_30 5002725 5198560 0.00482768986172 0.00153460408783 0.00482768986172 0.00153460408783 0.365496884939 0.364034539536 0.079025 0.0598871498966 0 0 NA Chr1 122177661

N00055 600000 800000 0.7865173 scaffold_30 4813253 5002725 0.00509586370386 0.00151204637145 0.00509586370386 0.00151204637145 0.362922098694 0.359845132743 0.09593 0.0595602516467 0 0 NA Chr1 122177661

N00055 800000 1000000 2.160584 scaffold_30 4613264 4813253 0.00587574534929 0.00152497753071 0.00587574534929 0.00152497753071 0.368051680474 0.368863306256 0.070515 0.0563480991455 0 0 NA Chr1 122177661

N00055 1000000 1200000 1.550365 scaffold_30 4418713 4613264 0.00498049950653 0.00131946812892 0.00498049950653 0.00131946812892 0.36875182537 0.368213911783 0.067545 0.0484962811808 0 0 NA Chr1 122177661

N00055 1200000 1400000 1.572173 scaffold_30 4222316 4418713 0.00449491670506 0.00136557084333 0.00451529269843 0.00137380881229 0.3760985227 0.374083315666 0.07589 0.0463398117079 0.00426 0.00593186250299 0.067 Chr1 122177661

N00055 1400000 1600000 3.012853 scaffold_30 4033175 4222316 0.0046838204629 0.00151999729223 0.00468768853106 0.00152238716423 0.394641348114 0.394954296963 0.08692 0.0412073532444 0.001205 0.00129532993904 0.067 Chr1 122177661

N00055 1600000 1800000 4.775388 scaffold_30 3835572 4033175 0.0050693438096 0.00136134224089 0.00507245505104 0.00136328571644 0.416270355568 0.413724449164 0.092175 0.049255325071 0.005895 0.00400297566332 0.094 Chr1 122177661

N00055 1800000 2000000 0.9177215 scaffold_30 3636910 3835572 0.00548985698316 0.00141243572584 0.0055267530631 0.00142168826636 0.390539610138 0.390214681738 0.060565 0.0518720238395 0.008745 0.0083710020034 0.13 Chr1 122177661

N00055 2000000 2200000 0.4902253 scaffold_30 3440770 3636910 0.00432532385882 0.00119481188677 0.00437584368069 0.0012133767928 0.410288449202 0.406136325944 0.072785 0.0529468746814 0.023155 0.0248088100336 0.0955 Chr1 122177661

N00055 2200000 2400000 3.004525 scaffold_30 3248139 3440625 0.00356655123325 0.000957184587976 0.00360908632893 0.000979491028962 0.456850257476 0.458529203611 0.086825 0.0446577932941 0.04474 0.0438265640099 0.136 Chr1 122177661

N00055 2400000 2600000 6.554595 scaffold_30 3050165 3246888 0.00401589540293 0.00103434547952 0.00402719087653 0.00104409045808 0.481528804575 0.478412347897 0.09477 0.0488504140339 0.0296 0.0324771379046 0.18725 Chr1 122177661

N00055 2600000 2800000 3.756443 scaffold_30 2846374 3050165 0.00384080401408 0.00108526619136 0.00386110689304 0.00109288795481 0.475296206327 0.472583246632 0.06362 0.0396680913289 0.028045 0.0276312496626 0.161 Chr1 122177661

N00055 2800000 3000000 2.296053 scaffold_30 2655945 2846374 0.00380791181391 0.00112429226452 0.00388324682807 0.00115658808037 0.443678285083 0.441278153592 0.11934 0.0608730813059 0.046695 0.0500081395166 0.138 Chr1 122177661

N00055 3000000 3200000 3.862129 scaffold_30 2458234 2655945 0.0036980758247 0.00104489288316 0.00374522835282 0.00106031745557 0.410042945744 0.40764538078 0.06006 0.0309947347391 0.016245 0.0165241185367 0.0215 Chr1 122177661

N00055 3200000 3400000 4.289812 scaffold_30 2267700 2458234 0.00405836494349 0.00130898344882 0.00411248732076 0.00133967973965 0.410266518125 0.406254780318 0.09044 0.0367703402017 0.02338 0.0273704430705 0.074 Chr1 122177661

N00055 3400000 3600000 1.467031 scaffold_30 2058779 2267700 0.00428813066876 0.00123801998595 0.00438853004136 0.0012810940757 0.400565883068 0.395306662005 0.048915 0.0409006275099 0.035695 0.0459312371662 0.0941666666667 Chr1 122177661

N00055 3600000 3800000 3.282381 scaffold_30 1860978 2058779 0.00480298062143 0.00154694404161 0.00482556712179 0.00155416747618 0.377479968727 0.375061155564 0.042255 0.0289128973059 0.0073 0.00760865718576 0.133 Chr1 122177661

N00055 3800000 4000000 2.868902 scaffold_30 1660445 1860978 0.00519340994198 0.00165903482368 0.00520461043525 0.00166416289116 0.37233315497 0.370973449458 0.04915 0.0401031251714 0.00414 0.00407414241048 0.133 Chr1 122177661

N00055 4000000 4200000 10.15634 scaffold_30 1454918 1660445 0.00539757483929 0.00161111505336 0.00539757483929 0.00161111505336 0.385326260823 0.388470661574 0.053535 0.0465680908105 0 0 NA Chr1 122177661

N00055 4200000 4400000 4.466704 scaffold_30 1251918 1454918 0.00509418335955 0.00160846391578 0.00509418335955 0.00160846391578 0.385621216548 0.389928443367 0.049 0.0389113300493 0 0 NA Chr1 122177661

N00055 4400000 4600000 2.415011 scaffold_30 1040041 1251918 0.00490186919359 0.00151768697092 0.00490186919359 0.00151768697092 0.391748490578 0.400289725632 0.08185 0.0493777049892 0 0 NA Chr1 122177661

N00055 4600000 4800000 3.47773 scaffold_30 847064 1040041 0.00443376738081 0.00149931709251 0.00450048267643 0.00153241053719 0.39538183198 0.397937725458 0.064575 0.0219974401094 0.02472 0.027137949082 0.182333333333 Chr1 122177661

N00055 4800000 5000000 4.339394 scaffold_30 662761 847064 0.00463902105636 0.00137719776484 0.00466853112989 0.00138646368899 0.402045459691 0.41506724476 0.08402 0.0297227934434 0.010705 0.0116167398252 0.224 Chr1 122177661

N00055 5000000 5200000 1.996699 scaffold_30 464896 662761 0.00461075817196 0.00130190361344 0.00461241335087 0.00130363306205 0.391794706781 0.394554406804 0.080105 0.0447021959417 0.00392 0.00378540924368 0.224 Chr1 122177661

N00055 5200000 5400000 2.023053 scaffold_30 275475 464896 0.00439456041552 0.00149754934804 0.0044038543796 0.00150360457018 0.40441773414 0.413711126171 0.06645 0.0264701379467 0.003895 0.00440289091495 0.096 Chr1 122177661

N00055 5400000 5600000 6.679847 scaffold_30 79054 275475 0.00477499253631 0.00138958955561 0.00483979442838 0.00141041396702 0.395293437016 0.394009320336 0.09164 0.0738923027579 0.01999 0.0187403587193 0.0735 Chr1 122177661

N00275 200000 400000 3.361698 scaffold_133 157321 351918 0.00482855336778 0.00128170493433 0.00483936713331 0.00128319719964 0.403988523852 0.403128548279 0.07927 0.0635569921427 0.009865 0.008926139663 0.114 Chr1 122177661

N00123 2000000 2200000 5.130505 scaffold_77 120299 315356 0.00371907470498 0.00112522844577 0.00374956986149 0.00113894639943 0.434031158715 0.433185475782 0.09238 0.0857134068503 0.01001 0.0127193589566 0.047 Chr1A 75309072

N00069 400000 600000 4.670314 scaffold_62 4038263 4231314 0.00422445741384 0.00137062926042 0.00424879084773 0.00138496651234 0.43655685441 0.439640063518 0.13605 0.0815121392793 0.01408 0.0138927019285 0.053 Chr1A 75309072

N00069 600000 800000 3.410835 scaffold_62 3854192 4038263 0.00393739707398 0.00120057441522 0.00405429070611 0.00125200581834 0.430703317107 0.43162025949 0.13521 0.0775841930559 0.04415 0.0468351885957 0.07225 Chr1A 75309072

N00069 800000 1000000 4.022142 scaffold_62 3659529 3854192 0.00322440851492 0.000971425311233 0.00325048402339 0.000980004878641 0.430701476979 0.434427690238 0.11319 0.0824450460539 0.01853 0.0190585781582 0.0475 Chr1A 75309072

N00069 1000000 1200000 2.996081 scaffold_62 3471699 3658833 0.00353577467568 0.0011919892782 0.00353577467568 0.0011919892782 0.418865776066 0.424772667782 0.12109 0.0895721782252 0 0 NA Chr1A 75309072

N00069 1200000 1400000 1.259012 scaffold_62 3276362 3471699 0.00373255253131 0.00108083506473 0.00373255253131 0.00108083506473 0.38586371804 0.387034576348 0.11325 0.0911655241966 0 0 NA Chr1A 75309072

N00069 1600000 1800000 0.4205842 scaffold_62 2894257 3086584 0.00420332380814 0.00128020624685 0.00420332380814 0.00128020624685 0.373160652223 0.378194245766 0.101365 0.0978957712646 0 0 NA Chr1A 75309072

N00069 1800000 2000000 0.1742839 scaffold_62 2698166 2894257 0.00367722022361 0.00126322759 0.00367722022361 0.00126322759 0.383824633346 0.386418791299 0.089615 0.0893003758459 0 0 NA Chr1A 75309072

N00069 2000000 2200000 3.354964 scaffold_62 2502973 2698166 0.00321531271655 0.00102050760119 0.00321531271655 0.00102178288426 0.38776026727 0.392425664754 0.099815 0.0820777384435 0 0.00100413436957 NA Chr1A 75309072

N00069 2200000 2400000 0 scaffold_62 2309038 2502973 0.00296108262824 0.000902018914993 0.00297188487929 0.000905130811127 0.399865424602 0.403200242344 0.071625 0.0574780209864 0.003885 0.00362492587723 0.014 Chr1A 75309072

N00069 2400000 2600000 0.7487562 scaffold_62 2111234 2308283 0.00359376758918 0.00115820244725 0.0036327611728 0.00117771320836 0.396723722101 0.395427752988 0.07511 0.0713274363229 0.01718 0.0175286350096 0.0663333333333 Chr1A 75309072

N00069 2600000 2800000 1.522228 scaffold_62 1916105 2111234 0.00375285713699 0.00114055739657 0.00388446588026 0.0011919051861 0.397752440325 0.396734509743 0.08773 0.0613901572806 0.048845 0.0514890149593 0.0523333333333 Chr1A 75309072

N00069 2800000 3000000 0.8774876 scaffold_62 1721097 1916105 0.00395886321048 0.0011896895374 0.00399086797667 0.00119612905335 0.424747200413 0.424646092331 0.064605 0.0502594765343 0.00754 0.00843042336725 0.0373333333333 Chr1A 75309072

N00069 3000000 3200000 2.534886 scaffold_62 1530900 1721097 0.00365851920647 0.00107055861741 0.00371130740523 0.00109488005302 0.437142905872 0.435064492688 0.09533 0.0448587517153 0.024015 0.0250950330447 0.0546666666667 Chr1A 75309072

N00069 3200000 3400000 1.567474 scaffold_62 1336725 1530900 0.0033845552676 0.00106759978706 0.00347179693511 0.00110349840894 0.438449301832 0.437674493112 0.086395 0.0712810608987 0.03964 0.0401956997554 0.054 Chr1A 75309072

N00069 3400000 3600000 2.108352 scaffold_62 1146593 1336725 0.00389846846905 0.00105705796158 0.00391028632562 0.0010615490001 0.45207683576 0.456611223118 0.04401 0.0356857341216 0.00357 0.0041024130604 0.05 Chr1A 75309072

N00069 3600000 3800000 2.430303 scaffold_62 952714 1146593 0.00386732292533 0.00126683687664 0.00386732292533 0.00126683687664 0.431353622219 0.435382847525 0.073095 0.0595526075542 0 0 0.05 Chr1A 75309072

N00069 3800000 4000000 1.560016 scaffold_62 756849 952714 0.00384612689084 0.00123636673538 0.00390700251121 0.00126869796173 0.423023300993 0.422905071725 0.08002 0.0666632629617 0.026965 0.0281775712863 0.04175 Chr1A 75309072

N00069 4000000 4200000 4.618481 scaffold_62 555018 756849 0.00360203641987 0.00115945964207 0.00370140059697 0.00119642441651 0.421305687026 0.422065497649 0.09125 0.0947079487294 0.03488 0.0341077436073 0.078 Chr1A 75309072

N00303 0 200000 0.4091471 scaffold_62 29 188751 0.00389250167889 0.000868969348185 0.00389250167889 0.000868969348185 0.380534172225 0.384671717728 0.087555 0.0617998961435 0 0 NA Chr1A 75309072

N00029 0 200000 1.140157 scaffold_22 6909113 7103637 0.0042468128574 0.000897248046656 0.00425007754135 0.000898629927995 0.357028171901 0.355460671159 0.09998 0.0935771421521 0.00064 0.00135201825996 0.03 Chr1A 75309072

N00029 800000 1000000 0.5993062 scaffold_22 7693863 7905818 0.00514797816339 0.000955251284308 0.00514797816339 0.000955251284308 0.353717855779 0.355033471984 0.13155 0.147262390602 0 0 NA Chr1A 75309072

N00029 1000000 1200000 0.5993062 scaffold_22 7905818 8117826 0.00434873629241 0.000991888003275 0.00434873629241 0.000992568867145 0.376056726455 0.377239919683 0.07332 0.105684691144 0 0.000608467604996 0.053 Chr1A 75309072

N00029 1200000 1400000 0.989302 scaffold_22 8117826 8317952 0.00459127229557 0.000841172761075 0.00463608129768 0.000854405445549 0.356092074849 0.353690569264 0.092335 0.096054485674 0.01424 0.0150954898414 0.048 Chr1A 75309072

N00029 1400000 1600000 3.146993 scaffold_22 8317952 8519002 0.00397863306957 0.00105937186285 0.00400705970436 0.0010675477654 0.420697119387 0.420638855443 0.073005 0.084988808754 0.009055 0.0104501367819 NA Chr1A 75309072

N00029 1600000 1800000 3.146993 scaffold_22 8519002 8713427 0.00408244467277 0.00114039546974 0.00415501655501 0.00116842200937 0.427453068086 0.432260006019 0.048495 0.0330307316446 0.03798 0.039881702456 0.066 Chr1A 75309072

N00029 1800000 2000000 0.04697005 scaffold_22 8713427 8914873 0.00446679297715 0.00121507568935 0.00458114761001 0.00125572983474 0.402006904021 0.400996694693 0.074065 0.0551860051825 0.03729 0.0367145537762 0.07975 Chr1A 75309072

N00029 2000000 2200000 0 scaffold_22 8914873 9116593 0.00394419030187 0.000981775457491 0.00405204694848 0.0010151862205 0.390502328302 0.386780704833 0.07985 0.073091413841 0.03699 0.0388013087448 0.041 Chr1A 75309072

N00029 2200000 2400000 0 scaffold_22 9116593 9317950 0.00425139699084 0.00111264052941 0.00431271951469 0.00113301370959 0.394951532962 0.391679319769 0.066875 0.0625754257364 0.02608 0.0260929592713 0.0396666666667 Chr1A 75309072

N00029 2400000 2600000 0 scaffold_22 9317950 9512964 0.0045566028999 0.00112970672637 0.0047362883789 0.00117196449567 0.382513562387 0.380808728405 0.080375 0.0890807839437 0.04426 0.0471709723404 0.044 Chr1A 75309072

N00029 2600000 2800000 0 scaffold_22 9512964 9700184 0.0039873969765 0.000992283734996 0.00420692304534 0.00105113218695 0.416167588206 0.414525660572 0.113285 0.0573870313001 0.09337 0.0910319410319 0.0666666666667 Chr1A 75309072

N00029 2800000 3000000 0 scaffold_22 9700184 9885833 0.00372215255455 0.000874016018653 0.00379049463406 0.000899288849732 0.368429752066 0.369857777278 0.08695 0.0482361876444 0.02357 0.029329541231 0.0592 Chr1A 75309072

N00029 3000000 3200000 1.021471 scaffold_22 9885833 10087304 0.00388127308318 0.00089239987896 0.00397048941401 0.000914925031811 0.383310092236 0.383751437407 0.07939 0.0751522551633 0.024845 0.0250408247341 0.0385 Chr1A 75309072

N00029 3400000 3600000 1.387268 scaffold_22 10286644 10473764 0.00430614607793 0.000979884919898 0.00434785892278 0.00099497879264 0.398692134418 0.397207975011 0.065095 0.0439931594699 0.017235 0.0198108165883 0.0785 Chr1A 75309072

N00029 3600000 3800000 1.387268 scaffold_22 10473764 10664958 0.00411315647798 0.000948431408733 0.00423929998293 0.000984168127313 0.377929467993 0.378754632476 0.080015 0.0637520005858 0.03386 0.0378254547737 0.0303333333333 Chr1A 75309072

N00029 4000000 4200000 2.869102 scaffold_22 10863710 11061435 0.00396501234222 0.00230463710776 0.00397117751161 0.00230846939316 0.404677562193 0.41222969133 0.125685 0.107163990391 0.0032 0.00167404223037 NA Chr1A 75309072

N00029 4200000 4400000 2.869102 scaffold_22 11061435 11254594 0.00389607303557 0.000928342387541 0.00391105139612 0.000936323912161 0.394959467141 0.401637097511 0.06242 0.0403294695044 0.007275 0.00797270642321 NA Chr1A 75309072

N00029 4400000 4600000 5.956167 scaffold_22 11254594 11446773 0.0041635474269 0.00109686029033 0.00424405134831 0.00112144899183 0.388367517054 0.392413793103 0.07339 0.049386249278 0.026875 0.032974466513 NA Chr1A 75309072

N00029 4600000 4800000 2.007863 scaffold_22 11446773 11645327 0.00358760217245 0.000969719392414 0.00378902972085 0.0010290874809 0.383115413928 0.384395561343 0.063625 0.0635091713086 0.069175 0.0716228330832 0.0616666666667 Chr1A 75309072

N00029 4800000 5000000 0.9572271 scaffold_22 11645327 11848340 0.00420836039698 0.00101158948237 0.00429656767491 0.00104239884483 0.374789377441 0.373095077163 0.083775 0.0580701728461 0.03594 0.0423273386433 0.068 Chr1A 75309072

N00029 5000000 5200000 0.9572271 scaffold_22 11848340 12042106 0.00351003700272 0.000962884796116 0.00357608080311 0.0009844107859 0.37841718322 0.379641323567 0.085535 0.0547258032885 0.02352 0.0245450698265 0.0573333333333 Chr1A 75309072

N00029 5200000 5400000 1.168897 scaffold_22 12042106 12242120 0.00369852809784 0.000869330541049 0.00377421035751 0.000888162578752 0.379536312361 0.377778869135 0.05899 0.0595358324917 0.024645 0.0256832021758 0.033 Chr1A 75309072

N00029 5400000 5600000 1.400411 scaffold_22 12242120 12441095 0.00397286129121 0.00108968798286 0.00397799322411 0.00109517118916 0.371338347341 0.373068847564 0.06648 0.073521799221 0.002405 0.00499057670562 0.033 Chr1A 75309072

N00029 5600000 5800000 1.373297 scaffold_22 12441110 12637446 0.00463486646915 0.00099590475412 0.00463486646915 0.000996594271403 0.368514763341 0.369871215667 0.07951 0.071576277402 0 0.000707970010594 0.033 Chr1A 75309072

N00029 6000000 6200000 0.7948718 scaffold_22 12829050 13027764 0.004526294823 0.000914404112054 0.004526294823 0.000914404112054 0.381906546562 0.382027018787 0.09788 0.0800195255493 0 0 NA Chr1A 75309072

N00029 6200000 6400000 0.7948718 scaffold_22 13027764 13226655 0.00422814735824 0.000912930492951 0.00426143807928 0.000920001308144 0.386099543256 0.390518151614 0.08721 0.0706819313091 0.008315 0.00760718182321 0.0735 Chr1A 75309072

N00029 6400000 6600000 0.9094208 scaffold_22 13226655 13416675 0.00383254854243 0.000953896966013 0.00389599462783 0.000961278717628 0.377501194513 0.383994657778 0.088785 0.0660193663825 0.020105 0.0212240816756 0.0955 Chr1A 75309072

N00029 6600000 6800000 0.9193277 scaffold_22 13416675 13614150 0.00462089103023 0.00108435596733 0.00461954348557 0.00108484380095 0.368366302122 0.373699439095 0.071225 0.0673300417774 0.000515 0.000521585010761 0.081 Chr1A 75309072

N00029 6800000 7000000 0.9193277 scaffold_22 13614150 13815307 0.00460646583221 0.000989444868295 0.00460646583221 0.000989444868295 0.363200813822 0.361640794553 0.094475 0.0910532569088 0 0 NA Chr1A 75309072

N00029 7000000 7200000 0.5457806 scaffold_22 13815307 14009536 0.0036146095948 0.000867581717896 0.00368849143634 0.000883503094242 0.358848578126 0.361155133898 0.052495 0.0257119173759 0.023085 0.0236473441144 0.079 Chr1A 75309072

N00029 7200000 7400000 0.5892857 scaffold_22 14009536 14205093 0.00448287856915 0.00126828484585 0.00449073326026 0.00127251784924 0.356835235093 0.358321953351 0.081595 0.0724392376647 0.00294 0.00300679597253 0.079 Chr1A 75309072

N00029 7400000 7600000 0.5892857 scaffold_22 14205093 14404683 0.00392779699027 0.000927339377276 0.00393049666663 0.000928400660074 0.370874317856 0.372762521617 0.091265 0.0887870133774 0.000885 0.000886817976853 0.079 Chr1A 75309072

N00029 7600000 7800000 0.5549099 scaffold_22 14405531 14601286 0.00394538609231 0.00110307595641 0.00398713533891 0.00112612987229 0.360713819319 0.359029643635 0.080175 0.0761308778831 0.014085 0.023079870246 NA Chr1A 75309072

N00029 7800000 8000000 0.5180534 scaffold_22 14601286 14803139 0.00402916454666 0.00120427032621 0.00420467301826 0.0012541067324 0.360295263434 0.35804918129 0.0933 0.0809202736645 0.05497 0.0532962106087 NA Chr1A 75309072

N00029 8000000 8200000 0.5180534 scaffold_22 14803139 14999242 0.00467295319419 0.00136238400589 0.0047104342001 0.00137748989943 0.36042185702 0.358949936502 0.086535 0.0752512710157 0.01107 0.0118713125245 0.109 Chr1A 75309072

N00029 8200000 8400000 1.225393 scaffold_22 14999242 15194794 0.00438220735287 0.00111153108406 0.00441598964402 0.00111987609471 0.366018441449 0.365938906701 0.05654 0.0435587465227 0.009315 0.00954221894944 0.068 Chr1A 75309072

N00029 8400000 8600000 2.88764 scaffold_22 15194794 15387613 0.00344394633896 0.00112205231176 0.00344676990579 0.00112319083232 0.376218313026 0.376453687957 0.04813 0.0369724975236 0.0031 0.00267608482567 0.068 Chr1A 75309072

N00029 8600000 8800000 2.88764 scaffold_22 15387613 15577656 0.00405181114668 0.00112239237753 0.00405181114668 0.00112239237753 0.358594833685 0.358964763244 0.063915 0.0554295606784 0 0 NA Chr1A 75309072

N00034 800000 1000000 1.422874 scaffold_22 5866085 6076043 0.00561978750059 0.00119006735727 0.00561978750059 0.00119006735727 0.346890921567 0.346148857824 0.11301 0.114965850313 0 0 NA Chr1A 75309072

N00034 1000000 1200000 1.401637 scaffold_22 5662563 5866085 0.00476833148868 0.00107697526061 0.00477651497042 0.0010810303737 0.360961677112 0.357745253341 0.1009 0.106750130207 0.002645 0.00350330676782 0.033 Chr1A 75309072

N00034 1200000 1400000 0.6427578 scaffold_22 5461994 5662019 0.00411963238579 0.000920836957757 0.00427292209886 0.000963043584769 0.375959950573 0.376194804518 0.09149 0.0688363954506 0.044705 0.0447594050744 0.033 Chr1A 75309072

N00034 1400000 1600000 1.291943 scaffold_22 5265060 5461994 0.00457438322598 0.000902900286837 0.00457438322598 0.000902900286837 0.3791908017 0.382914572864 0.074645 0.0650319396346 0 0 NA Chr1A 75309072

N00034 1600000 1800000 1.291943 scaffold_22 5067346 5265060 0.00416133366071 0.00117006562162 0.00416693847224 0.00117248546167 0.366384863347 0.369452794117 0.107895 0.0680831908717 0.006075 0.00371749092123 0.011 Chr1A 75309072

N00034 1800000 2000000 1.291943 scaffold_22 4855969 5067346 0.00496057809599 0.00116123668974 0.00496057809599 0.00116123668974 0.356038408027 0.357118875363 0.076635 0.100990173955 0 0 NA Chr1A 75309072

N00034 2200000 2400000 2.32607 scaffold_22 4459925 4653265 0.00479424044991 0.00102796173407 0.00479424044991 0.00102796173407 0.367785265278 0.365528684752 0.086395 0.0858642805421 0 0 NA Chr1A 75309072

N00034 2400000 2600000 5.911043 scaffold_22 4247683 4459925 0.00450724464989 0.000988067171489 0.00460337316529 0.00101583365872 0.375884103214 0.372923789878 0.08611 0.10109686113 0.02893 0.0295700191291 0.0803333333333 Chr1A 75309072

N00034 2600000 2800000 2.421295 scaffold_22 4043590 4247683 0.00455630107144 0.000913388285191 0.00476742168774 0.000962604684048 0.392789998921 0.392596342393 0.0662 0.034621471584 0.069705 0.0654064568604 0.0718 Chr1A 75309072

N00034 2800000 3000000 0.02078775 scaffold_22 3836060 4043590 0.00409217085356 0.000962451748649 0.00418919940197 0.000992484223116 0.395339529506 0.389841591139 0.085765 0.090777237026 0.034095 0.0325302365923 0.06 Chr1A 75309072

N00034 3000000 3200000 0.02078775 scaffold_22 3644574 3836060 0.00378777568081 0.000708528729307 0.00378777568081 0.000708539198362 0.406340701049 0.406944331682 0.07556 0.0566882174154 0 1.04446278057e-05 NA Chr1A 75309072

N00034 3200000 3400000 0.02078775 scaffold_22 3438418 3644574 0.00433900372627 0.00100631676339 0.00438131448569 0.00101882961938 0.379344571272 0.385885129995 0.090435 0.087530801917 0.012525 0.0125827043598 0.068 Chr1A 75309072

N00034 3400000 3600000 0.02078775 scaffold_22 3238535 3438418 0.00463061390672 0.00111317498849 0.00464522629814 0.00111575705906 0.359767453159 0.363486608071 0.081775 0.0689503359465 0.004235 0.00423747892517 0.111 Chr1A 75309072

N00034 4000000 4200000 2.11701 scaffold_22 2624711 2833476 0.00514048539452 0.00106910938733 0.00514468822957 0.00107233818496 0.359020862008 0.359565616362 0.10833 0.106804301487 0.003465 0.00331952195052 NA Chr1A 75309072

N00034 4200000 4400000 2.11701 scaffold_22 2424387 2624711 0.00471679753524 0.00111381382532 0.00475357506552 0.00112310370824 0.372221721801 0.380246445709 0.08889 0.0901589425131 0.01405 0.0123699606637 0.074 Chr1A 75309072

N00034 4400000 4600000 1.506131 scaffold_22 2211062 2424387 0.00473714215129 0.00110450304215 0.0048055599399 0.00111905434065 0.389303096472 0.388789068565 0.08013 0.0863846243994 0.01824 0.0176397515528 0.0766666666667 Chr1A 75309072

N00034 4600000 4800000 0 scaffold_22 2012238 2211062 0.00430913883164 0.000937636858704 0.0043766635306 0.000964952818644 0.381115249316 0.38210846891 0.09805 0.0789693397175 0.028795 0.0391602623426 0.1218 Chr1A 75309072

N00034 4800000 5000000 0 scaffold_22 1807133 2012217 0.00454855738768 0.0010556113151 0.00459392667535 0.00106695633539 0.381776239908 0.37887637403 0.09983 0.0959460513741 0.01129 0.0122486395818 0.0803333333333 Chr1A 75309072

N00034 5000000 5200000 0 scaffold_22 1611530 1807133 0.00459409106303 0.00105125462457 0.00465265281017 0.0010643378217 0.364668810428 0.3633963128 0.03948 0.0297132457069 0.014565 0.0195549148019 0.059 Chr1A 75309072

N00034 5200000 5400000 2.084505 scaffold_22 1414221 1611530 0.00391557296911 0.00115965631833 0.00408114507842 0.00121707095568 0.382815066886 0.386485219817 0.067685 0.0620448129584 0.05765 0.0605091506216 0.05675 Chr1A 75309072

N00034 5400000 5600000 2.137681 scaffold_22 1218470 1414221 0.00471354934188 0.00116605774239 0.00471354934188 0.00116644464883 0.385425190148 0.385387521182 0.111485 0.0603623991704 0.000285 0.000653891934141 0.059 Chr1A 75309072

N00034 5600000 5800000 2.137681 scaffold_22 1018707 1218470 0.00460043170921 0.00121612601825 0.00464558793204 0.00123517897086 0.380504422464 0.378915852331 0.075985 0.0697226213062 0.01888 0.0211700865526 0.165 Chr1A 75309072

N00034 5800000 6000000 2.137681 scaffold_22 815549 1018707 0.00428726008614 0.000962527838967 0.00433240882427 0.000974938243385 0.38151174668 0.382019194633 0.09449 0.0796227566721 0.01423 0.0212297817462 0.059 Chr1A 75309072

N00034 6000000 6200000 2.137681 scaffold_22 624614 815549 0.00451524654902 0.000985759336002 0.00452580931894 0.000989802718726 0.358673104946 0.360138321637 0.108465 0.0670123340404 0.00519 0.00583968366198 NA Chr1A 75309072

N00034 6800000 7000000 2.185943 scaffold_11 10794 220753 0.00573690897485 0.00162252801147 0.00559723566257 0.00156170615816 0.387306117316 0.385891463284 0.08332 0.0820350639887 0.026935 0.0346305707305 0.062 Chr1A 75309072

N00034 7000000 7200000 6.988 scaffold_11 220753 432098 0.00445426493035 0.000957532081222 0.0046401136373 0.00100178294447 0.36870254317 0.371487896399 0.076835 0.0697532470605 0.049045 0.0477560387045 0.0835 Chr1A 75309072

N00034 7200000 7400000 4.968348 scaffold_11 432098 635160 0.00418585990844 0.00110095297778 0.00422349021239 0.0011163336477 0.401791513315 0.399444050798 0.066995 0.0541410997626 0.01556 0.0149510986792 0.045 Chr1A 75309072

N00034 7400000 7600000 4.317276 scaffold_11 635160 823643 0.00397924047844 0.000953313180927 0.00404902320948 0.00097592859303 0.390902817366 0.392569997986 0.10227 0.0800443541327 0.028955 0.0267079789689 0.07 Chr1A 75309072

N00034 7600000 7800000 4.317276 scaffold_11 823643 1025933 0.00478582552457 0.00107371921933 0.00482642036652 0.0010838039482 0.387817679249 0.388514059357 0.09163 0.0953532057937 0.01056 0.010173513273 0.066 Chr1A 75309072

N00034 7800000 8000000 1.654637 scaffold_11 1025933 1217978 0.00446084991715 0.00115454571655 0.00447498366928 0.00116069745827 0.366060865548 0.366320447288 0.07635 0.0661876122784 0.006085 0.00617042880575 0.146 Chr1A 75309072

N00034 8000000 8200000 0.7493404 scaffold_11 1217978 1406972 0.00379576645798 0.000957001755997 0.00379576645798 0.000957001755997 0.372751552228 0.372909615905 0.091755 0.071266812703 0 0 NA Chr1A 75309072

N00034 8200000 8400000 0.7493404 scaffold_11 1406972 1595687 0.00378324902696 0.000999266709579 0.00379100600955 0.00100213308346 0.360486725411 0.35946837927 0.09649 0.0698195691916 0.003535 0.00347614127123 0.133 Chr1A 75309072

N00117 200000 400000 1.866485 scaffold_11 3704105 3920843 0.0052504327858 0.00122095077354 0.0052504327858 0.00122095077354 0.356729953608 0.355439708375 0.097445 0.114211628787 0 0 NA Chr1A 75309072

N00117 600000 800000 1.866485 scaffold_11 3306445 3499345 0.00456023088105 0.00125472328574 0.00456790609423 0.00125659608938 0.349983644833 0.348773915825 0.07626 0.079300155521 0.001495 0.00149818558839 NA Chr1A 75309072

N00117 800000 1000000 0.7660142 scaffold_11 3107553 3306445 0.0043540593196 0.0010368956627 0.00438965950251 0.00104608963403 0.34732110435 0.345934965991 0.06928 0.0754731210908 0.0108 0.0106942461235 0.073 Chr1A 75309072

N00117 1000000 1200000 0.7493404 scaffold_11 2906159 3107553 0.0046021341698 0.00113679530767 0.00470473374106 0.00116641060139 0.361099283709 0.357908515424 0.0583 0.0477571327845 0.02766 0.0281289412793 0.04 Chr1A 75309072

N00117 1200000 1400000 0.7493404 scaffold_11 2709809 2906159 0.00454155188214 0.00107265640748 0.00458047023054 0.00108264981397 0.384174917583 0.382916053019 0.072675 0.0662490450726 0.010615 0.0115100585689 0.0315 Chr1A 75309072

N00117 1400000 1600000 0.7493404 scaffold_11 2507745 2709809 0.00468324314295 0.00135939278805 0.00478932399934 0.00138407180617 0.382303002862 0.378210352949 0.08596 0.0487716763006 0.024515 0.0235024546678 0.0785 Chr1A 75309072

N00117 1600000 1800000 0.7493404 scaffold_11 2303405 2507745 0.00399157525013 0.00121146704854 0.00400979608767 0.001216871467 0.377109977299 0.377310795193 0.116155 0.111617891749 0.00555 0.0054468043457 NA Chr1A 75309072

N00117 1800000 2000000 0.7493404 scaffold_11 2107605 2303405 0.00441725178523 0.00109485250562 0.00444072176367 0.00110664718693 0.377049088043 0.380879784905 0.077335 0.0620173646578 0.01753 0.0221859039837 0.115 Chr1A 75309072

N00117 2000000 2200000 0.7493404 scaffold_11 1909270 2107605 0.00428021359429 0.000967829390472 0.00428021359429 0.000967829390472 0.362907044461 0.363180775269 0.08905 0.0534348450853 0 0 NA Chr1A 75309072

N00117 2200000 2400000 0.7493404 scaffold_11 1719105 1909270 0.00319079011251 0.000788241815043 0.00321341712678 0.0007963326917 0.359392471114 0.35871212324 0.05953 0.0280072568559 0.00667 0.0108011463729 0.013 Chr1A 75309072

N00111 0 200000 1.555041 scaffold_11 6662351 6857187 0.00406318010797 0.00103449436419 0.00410294588957 0.00104729606124 0.402425146603 0.400318380385 0.03945 0.026032150116 0.01514 0.0156439261738 0.074 Chr1A 75309072

N00111 200000 400000 2.795699 scaffold_11 6470260 6662351 0.00364642826843 0.00105837510597 0.00382062538859 0.00111026972797 0.387735265018 0.386542779891 0.073325 0.0328177790735 0.053835 0.0568688798538 0.068 Chr1A 75309072

N00111 400000 600000 2.795699 scaffold_11 6273074 6470260 0.00391792433795 0.000900063332409 0.00396082612882 0.000911758644412 0.388630101037 0.387166702796 0.06243 0.0502774030611 0.01484 0.0155690566267 0.064 Chr1A 75309072

N00111 600000 800000 2.795699 scaffold_11 6067522 6273074 0.0046603531258 0.000979068352356 0.00470373083679 0.000989528391935 0.387794827306 0.387674873169 0.07336 0.0735385693158 0.01351 0.0137288861213 0.0665 Chr1A 75309072

N00111 800000 1000000 3.211522 scaffold_11 5860358 6067522 0.00426742562094 0.00116402757976 0.00430280690167 0.00117526661084 0.378302486768 0.377459237485 0.08104 0.0940752254253 0.01063 0.0102961904578 0.0695 Chr1A 75309072

N00111 1000000 1200000 2.319322 scaffold_11 5660702 5859623 0.00426617164141 0.0011500135164 0.00433819950865 0.00117071919309 0.371656615413 0.375198972167 0.09116 0.0728580692838 0.028995 0.030429165347 0.0856 Chr1A 75309072

N00111 1200000 1400000 1.167092 scaffold_11 5454136 5660702 0.00412565898174 0.00108822539627 0.00419758462996 0.00111302012223 0.390402534071 0.39019976601 0.061075 0.0852657262086 0.024295 0.0236002052613 0.083 Chr1A 75309072

N00111 1400000 1600000 1.167092 scaffold_11 5261386 5454136 0.0038600126954 0.0010255011268 0.00390016485656 0.00104097385747 0.39484425953 0.396684133947 0.070365 0.0456186770428 0.01483 0.0153929961089 0.094 Chr1A 75309072

N00111 1600000 1800000 1.167092 scaffold_11 5069561 5261386 0.00382400347741 0.000981638343457 0.00386357809241 0.000995276742468 0.372563915267 0.37745054751 0.079625 0.0819757591555 0.01377 0.0157643685651 0.06475 Chr1A 75309072

N00111 1800000 2000000 0.3716113 scaffold_11 4862339 5069561 0.00416018608365 0.00102685431342 0.00418908832672 0.0010359960964 0.394346177276 0.39018652236 0.05501 0.0707357326925 0.01131 0.0109303066277 0.069 Chr1A 75309072

N00111 2200000 2400000 0 scaffold_11 4138884 4341056 0.00444103194689 0.0011843103319 0.00452846977585 0.00120397579669 0.371361674777 0.367991190236 0.10465 0.104984864373 0.02358 0.0232574243713 0.0653333333333 Chr1A 75309072

N00111 2400000 2600000 1.532189 scaffold_11 4341056 4541032 0.00493975329126 0.00120717814649 0.00500872946297 0.00123671869796 0.372779156599 0.37453466061 0.06589 0.0556416770012 0.013915 0.0233778053366 0.039 Chr1A 75309072

N00193 0 200000 1.490085 scaffold_11 6857456 7046482 0.00492646937703 0.00108062373368 0.00492646937703 0.00108983382747 0.385735597888 0.383649030069 0.08059 0.0649275761006 0 0.00828457460878 0.085 Chr1A 75309072

N00193 200000 400000 1.490085 scaffold_11 7046482 7248342 0.00468280339664 0.00113070784584 0.00471631494235 0.00114162176731 0.370705090911 0.368952924394 0.099425 0.104334687407 0.012895 0.0129297532944 0.055 Chr1A 75309072

N00193 400000 600000 1.211228 scaffold_11 7248342 7450449 0.00444670877603 0.00109194589251 0.00450052466881 0.00110957050455 0.37410436738 0.372403828788 0.096725 0.0769592344649 0.014025 0.0163873591711 0.062 Chr1A 75309072

N00193 600000 800000 0.8306709 scaffold_11 7450449 7647315 0.00483096329947 0.00128849209547 0.00483096329947 0.00128849209547 0.367025552135 0.366505456349 0.112475 0.071038168094 0 0 NA Chr1A 75309072

N00193 800000 1000000 0.8306709 scaffold_11 7647315 7844668 0.00474908274558 0.00101026236358 0.00478376606685 0.00101669737411 0.356486680435 0.356065284017 0.109885 0.0612709206346 0.00751 0.00731177129306 0.039 Chr1A 75309072

N00002 0 200000 0.8306709 scaffold_11 8070704 8278796 0.00546446959114 0.00153340126287 0.00546446959114 0.00153340126287 0.353420947662 0.354725035295 0.09182 0.0843857524556 0 0 NA Chr1A 75309072

N00002 200000 400000 1.245805 scaffold_11 8278796 8485183 0.00455438361669 0.00123628842493 0.00456259064143 0.00123859965311 0.364595091074 0.365180869197 0.09232 0.114682610823 0.00194 0.00212222669063 0.045 Chr1A 75309072

N00002 400000 600000 1.333333 scaffold_11 8485183 8682260 0.0044375381088 0.00116054999698 0.00454906697449 0.00119047907553 0.365456768767 0.366588595359 0.118905 0.104608858466 0.025015 0.0258528392456 0.0393333333333 Chr1A 75309072

N00002 600000 800000 1.333333 scaffold_11 8682260 8890453 0.00383913118819 0.000949885989785 0.00390420963618 0.000966158997656 0.398969443907 0.394410374552 0.054955 0.0468699716129 0.02047 0.0172484185347 0.0405 Chr1A 75309072

N00002 800000 1000000 1.333333 scaffold_11 8890453 9104984 0.00440658313068 0.00124138077227 0.00442747679243 0.00124698597873 0.396170422868 0.394264443514 0.08016 0.0771683346463 0.01213 0.00507618945514 0.0865 Chr1A 75309072

N00002 1000000 1200000 1.333333 scaffold_11 9104984 9299433 0.00436597500274 0.00119999280946 0.00444388948741 0.00122939394756 0.400962456207 0.402500909167 0.071805 0.0599694521443 0.02859 0.0254256900267 0.046 Chr1A 75309072

N00002 1200000 1400000 1.333333 scaffold_11 9299433 9487402 0.00469798347065 0.00114505311605 0.0048571628407 0.0011895025436 0.394126828476 0.395183437391 0.062565 0.028440859929 0.035755 0.0382882283781 0.033 Chr1A 75309072

N00002 1400000 1600000 1.601646 scaffold_11 9489181 9679458 0.00449083284365 0.00123548328933 0.004576372539 0.00126597065995 0.395606103338 0.403863498005 0.08783 0.0461222323245 0.025525 0.0271236145199 0.066 Chr1A 75309072

N00002 1600000 1800000 4.171795 scaffold_11 9679458 9876807 0.00406091048295 0.000848853381216 0.00407799558364 0.000853419908958 0.407596762036 0.410408421329 0.081375 0.0496886226938 0.005145 0.00499622496187 0.06 Chr1A 75309072

N00002 1800000 2000000 3.657115 scaffold_11 9876807 10070546 0.00414939763476 0.000916002400971 0.00415171565875 0.000917157349608 0.409100565994 0.411856499271 0.06255 0.0441883152076 0.00179 0.00170332251121 0.082 Chr1A 75309072

N00002 2000000 2200000 0.604534 scaffold_11 10070546 10271456 0.00436386987331 0.000996897256229 0.00444592153322 0.00102743510783 0.388792803376 0.389083356411 0.065715 0.044467672092 0.03145 0.036717933403 0.0915 Chr1A 75309072

N00002 2200000 2400000 0.746289 scaffold_11 10271456 10472929 0.00514605409281 0.00125031792195 0.0051482666041 0.00125084173601 0.393197220783 0.392308639398 0.065275 0.069676830146 0.000665 0.000933127515846 0.069 Chr1A 75309072

N00002 2400000 2600000 1.494932 scaffold_11 10472929 10677461 0.00397517825296 0.00106240286971 0.00408775984635 0.00108701914708 0.403285635723 0.403895406708 0.080265 0.0487063149043 0.02597 0.025448340602 0.045 Chr1A 75309072

N00002 2600000 2800000 1.494932 scaffold_11 10677461 10882654 0.00449528546392 0.00098179703112 0.00453257115492 0.000991620829174 0.40926943807 0.411298984597 0.08351 0.0709478393512 0.013365 0.0110237678673 0.074 Chr1A 75309072

N00002 2800000 3000000 1.624767 scaffold_11 10882654 11079364 0.00435746506801 0.00102150093242 0.00447141884415 0.00105288712696 0.391216731111 0.39380769793 0.070385 0.0423262670937 0.03475 0.0388338162778 0.08375 Chr1A 75309072

N00002 3000000 3200000 2.147353 scaffold_11 11079364 11274443 0.00428553322775 0.000918410922083 0.00438629265831 0.000937940015243 0.379305100608 0.379354661823 0.091045 0.0750003844596 0.033885 0.0350114568969 0.062 Chr1A 75309072

N00002 3200000 3400000 2.147353 scaffold_11 11274535 11474441 0.00460158092449 0.00110431609766 0.00470773077452 0.00114067599737 0.397112635328 0.396769349626 0.04575 0.0329354796754 0.03492 0.0369473652617 0.048 Chr1A 75309072

N00002 3400000 3600000 2.147353 scaffold_11 11474454 11673094 0.00414346041799 0.00108163167743 0.00426283420122 0.00112429032301 0.391665241875 0.39102762602 0.056495 0.0303815948449 0.039455 0.0398056786146 0.026 Chr1A 75309072

N00002 3600000 3800000 2.076935 scaffold_11 11673094 11876016 0.00449785282112 0.00100377042514 0.00452935430519 0.00101512961143 0.388369606273 0.382721018343 0.059755 0.0591163107007 0.00835 0.0119060525719 0.063 Chr1A 75309072

N00002 3800000 4000000 2.04697 scaffold_11 11876016 12062129 0.00404758009416 0.00099081829269 0.00406435495787 0.000995825926306 0.383165210621 0.380701385633 0.06772 0.0503457576848 0.00454 0.00492711417257 0.178 Chr1A 75309072

N00002 4000000 4200000 2.04697 scaffold_11 12062129 12256909 0.00360800576905 0.00105792085258 0.0037794241194 0.00111481288947 0.371472540844 0.368969003293 0.09762 0.0673683129685 0.056845 0.0597340589383 0.0565 Chr1A 75309072

N00002 4200000 4400000 2.04697 scaffold_11 12256909 12455330 0.00422504625051 0.000841532831448 0.00426753554863 0.000848146778261 0.38913078716 0.389737570126 0.080045 0.0731323801412 0.0129 0.00860795984296 0.0365 Chr1A 75309072

N00002 4400000 4600000 2.04697 scaffold_11 12455330 12656378 0.00418095984345 0.0010952924476 0.00420963944299 0.00110555255314 0.374043682297 0.373097322237 0.06259 0.0589212526362 0.009455 0.00942560980462 0.03 Chr1A 75309072

N00002 4600000 4800000 2.04697 scaffold_11 12656378 12854093 0.00435124419315 0.0012795638581 0.00435124419315 0.0012795638581 0.366077709989 0.364201800772 0.12533 0.122909238045 0 0 NA Chr1A 75309072

N00002 4800000 5000000 1.971587 scaffold_11 12854093 13056623 0.00460811735049 0.00121463137112 0.00460811735049 0.00121463137112 0.370246845622 0.370317706932 0.089225 0.085083691305 0 0 NA Chr1A 75309072

N00002 5000000 5200000 1.325446 scaffold_11 13056623 13267429 0.00500715232585 0.00143960454087 0.00500715232585 0.00143960454087 0.381218957386 0.379643696377 0.084735 0.0878153373244 0 0 NA Chr1A 75309072

N00002 5200000 5400000 1.325446 scaffold_11 13267429 13461087 0.00478878657463 0.00115990343297 0.00480635856033 0.00116737034872 0.383580533222 0.388023627534 0.08951 0.0735471811131 0.00645 0.00995053134908 0.044 Chr1A 75309072

N00002 5400000 5600000 1.325446 scaffold_11 13461087 13649668 0.00459881693044 0.00116223395758 0.00463838060516 0.00118600342149 0.416258314438 0.420399931104 0.07623 0.0479157497309 0.01542 0.0250502436619 0.131 Chr1A 75309072

N00002 5600000 5800000 1.325446 scaffold_11 13649668 13854821 0.00426454558024 0.00111300681919 0.0043413678294 0.00114521940224 0.401618094537 0.40084982668 0.081265 0.0702792549951 0.024665 0.0403357494163 0.0605 Chr1A 75309072

N00002 5800000 6000000 1.325446 scaffold_11 13854821 14053309 0.00452083989782 0.00113446076308 0.0045860648414 0.00115517597026 0.396531340559 0.39527271722 0.07199 0.0502901938656 0.023655 0.0237394703962 0.0746666666667 Chr1A 75309072

N00002 6000000 6200000 1.325446 scaffold_11 14053309 14242808 0.00488377138687 0.00116350345287 0.0048916274546 0.00116614335542 0.398827330382 0.398865153538 0.099025 0.0636837133705 0.0021 0.00222164760764 0.118 Chr1A 75309072

N00002 6200000 6400000 1.325446 scaffold_11 14242822 14430674 0.00496560601718 0.00141400024288 0.00496560601718 0.00141670981752 0.36888659668 0.368298936937 0.0604 0.0377903881779 0 0.0022198326342 NA Chr1A 75309072

N00002 6400000 6600000 1.325446 scaffold_11 14430674 14622824 0.00481725155907 0.000971388008063 0.00488359578973 0.000986529661652 0.369430185793 0.371480603264 0.04368 0.0303825136612 0.02008 0.0236846213895 0.075 Chr1A 75309072

N00002 6600000 6800000 1.325446 scaffold_11 14622824 14826832 0.00457877924489 0.00108395164091 0.00462848714715 0.00109823476481 0.381457141954 0.379035623907 0.039375 0.0552380298812 0.01415 0.0138867103251 0.075 Chr1A 75309072

N00002 6800000 7000000 1.325446 scaffold_11 14826832 15018960 0.00453661020408 0.00105005396503 0.00453661020408 0.00105006109735 0.369619314575 0.369042498279 0.06943 0.0568162891406 0 5.20486342438e-06 0.033 Chr1A 75309072

N00002 7000000 7200000 1.325446 scaffold_11 15018967 15209277 0.00443280508581 0.00130110671994 0.00445999126912 0.0013082137751 0.377629576092 0.37492974492 0.06868 0.0339130891703 0.00636 0.00669959539698 0.033 Chr1A 75309072

N00002 7200000 7400000 1.325446 scaffold_11 15209277 15413054 0.00383067576608 0.0010049423633 0.00389086520311 0.00102193243902 0.369449049032 0.36715955562 0.077115 0.0732074768006 0.015585 0.0182405276356 0.028 Chr1A 75309072

N00002 7400000 7600000 1.325446 scaffold_11 15413054 15605941 0.00418850832834 0.00131030814272 0.00428770711265 0.0013595324573 0.372529093968 0.37110963958 0.06377 0.0457936512051 0.037695 0.043346622634 0.0383333333333 Chr1A 75309072

N00002 7600000 7800000 1.325446 scaffold_11 15605941 15795275 0.00455206904461 0.0011837774533 0.00464946038108 0.00122655697308 0.369514228058 0.374130337476 0.05579 0.0280245492093 0.039585 0.0394593681008 0.0346666666667 Chr1A 75309072

N00002 7800000 8000000 3.411019 scaffold_11 15795275 15997338 0.00438258185143 0.000980223597069 0.00445179050859 0.000998353509318 0.367576323576 0.363453493449 0.07132 0.0653311096044 0.023335 0.0227008408269 0.07 Chr1A 75309072

N00002 8000000 8200000 4.074357 scaffold_11 15997338 16194726 0.00493562149901 0.00132033762865 0.00496233028545 0.00132821334467 0.356090403085 0.355923357522 0.08156 0.0760127262042 0.00513 0.00560824366223 0.067 Chr1A 75309072

N00002 8200000 8400000 0 scaffold_11 16194808 16396627 0.00483749603417 0.00121868621958 0.00484260329908 0.00122023614515 0.362365266998 0.363453524391 0.09285 0.0931725952462 0.001245 0.00123377878198 0.067 Chr1A 75309072

N00002 8400000 8600000 0.4094689 scaffold_11 16396627 16593798 0.0043321100326 0.00117499914092 0.00434983179619 0.00118731325736 0.377367521748 0.377163158498 0.07235 0.0660796973186 0.011455 0.0117106471033 0.08 Chr1A 75309072

N00002 8600000 8800000 0.5755472 scaffold_11 16593798 16790173 0.00432893166987 0.00119822296362 0.00437956188847 0.00121216690676 0.364323100235 0.364209558421 0.072995 0.0685219605347 0.01212 0.0123640992998 0.036 Chr1A 75309072

N00002 9200000 9400000 0.5755472 scaffold_11 17186304 17392264 0.00538856510394 0.00126445545559 0.00545081115268 0.00127752510747 0.353255612124 0.356157357247 0.121195 0.098689065838 0.01093 0.0106331326471 0.043 Chr1A 75309072

N00002 9400000 9600000 0.5755472 scaffold_11 17392264 17586060 0.00456756318749 0.00106584362544 0.00459323187431 0.00108808075697 0.353531804943 0.353343942949 0.078955 0.0570600012384 0.00678 0.031115193296 0.018 Chr1A 75309072

N00002 9600000 9800000 0.5755472 scaffold_11 17586060 17787088 0.00418684254826 0.00103666844149 0.00419420008692 0.00103852916597 0.348943290572 0.347473611065 0.091785 0.0976082933721 0.001585 0.00159679248662 0.018 Chr1A 75309072

N00002 9800000 10000000 0.5755472 scaffold_11 17787088 17971071 0.00403782071154 0.00125489015083 0.0040941375793 0.00127417726133 0.361583151352 0.367261496225 0.109845 0.0530864264633 0.015955 0.0174309582951 0.064 Chr1A 75309072

N00002 10200000 10400000 0.5833718 scaffold_11 18177103 18377806 0.00524595293078 0.00142512727269 0.00524595293078 0.00142512727269 0.334778871268 0.335280893875 0.098285 0.0957833216245 0 0 NA Chr1A 75309072

N00002 10600000 10800000 0.5980149 scaffold_11 18572893 18773964 0.00363317175394 0.000990355273081 0.00371626884113 0.00101136623877 0.364445939883 0.364738927225 0.085625 0.0736108140905 0.02965 0.0295368302739 0.062 Chr1A 75309072

N00002 10800000 11000000 0.5980149 scaffold_11 18773964 18990845 0.00333557393948 0.000907495647557 0.00333557393948 0.000908116398672 0.381512958509 0.376916868442 0.120275 0.136535703911 0 0.0128088675357 NA Chr1A 75309072

N00002 11000000 11200000 0.7065489 scaffold_11 18990845 19183559 0.00335758348486 0.000896221996566 0.00341614732263 0.000919456589739 0.390871456885 0.394022928401 0.057015 0.0560000830246 0.03427 0.0346316302915 0.11 Chr1A 75309072

N00002 11200000 11400000 0.9388795 scaffold_11 19183559 19376996 0.00327796087776 0.000928353939404 0.00329327219718 0.00093321816385 0.387510901204 0.389246978961 0.090225 0.0684770752235 0.005525 0.00571762382585 0.045 Chr1A 75309072

N00002 11400000 11600000 0.9388795 scaffold_11 19376996 19584389 0.00457102187715 0.00140635195884 0.00457102187715 0.00140635195884 0.373153003479 0.371066946733 0.071415 0.0614967718293 0 0 NA Chr1A 75309072

N00002 11600000 11800000 0.9388795 scaffold_11 19584389 19779731 0.00516800174377 0.00166904145766 0.00523026282735 0.00169850441061 0.361323545275 0.363172707889 0.100085 0.0783139314638 0.020525 0.0200212959835 0.1035 Chr1A 75309072

N00002 11800000 12000000 0.9388795 scaffold_11 19779731 19968743 0.00472477596109 0.00108006313373 0.00472576988986 0.00108112366173 0.388363281856 0.389205721762 0.09329 0.0407963515544 0.00104 0.0011004592301 0.098 Chr1A 75309072

N00002 12000000 12200000 0.9388795 scaffold_11 19968743 20163567 0.00458497330685 0.00110951996699 0.00459262779948 0.00111176155353 0.392554626383 0.396423201373 0.054995 0.0392046154478 0.00234 0.00198640824539 0.25 Chr1A 75309072

N00002 12400000 12600000 0.9388795 scaffold_8 23013701 23214314 0.00429796375524 0.00121196766614 0.00432535280643 0.00121918239268 0.389501329013 0.385433569638 0.06554 0.0343447333922 0.00667 0.00640038282664 0.024 Chr1A 75309072

N00002 12600000 12800000 0.9388795 scaffold_8 22809708 23013701 0.0036586523761 0.000920886412968 0.00369359670159 0.000930360713867 0.407344796139 0.403600038661 0.11049 0.081174354022 0.01049 0.012745535386 0.0385 Chr1A 75309072

N00002 12800000 13000000 4.428349 scaffold_8 22612883 22809708 0.00370952734221 0.0010586983579 0.0038165276358 0.00109191160779 0.395258104943 0.392380503779 0.07097 0.0557297091325 0.03737 0.0391566112028 0.0423333333333 Chr1A 75309072

N00002 13000000 13200000 0.8129921 scaffold_8 22423670 22612883 0.0042943157184 0.00128091933466 0.00435163930104 0.00130218247085 0.391837859387 0.392955511476 0.095075 0.0724051730061 0.01524 0.0163942223843 0.0593333333333 Chr1A 75309072

N00002 13200000 13400000 0.8129921 scaffold_8 22227421 22423670 0.00381449253536 0.00096980821085 0.00392361807356 0.00100767269517 0.383027728086 0.379098881897 0.052135 0.0299976050833 0.04375 0.0491008871383 0.05225 Chr1A 75309072

N00002 13400000 13600000 0.8129921 scaffold_8 22023905 22227421 0.00397225870591 0.00112146997695 0.00404579325009 0.00114958983342 0.431501243855 0.434730484743 0.081675 0.0336435464534 0.03511 0.0341889581163 0.118666666667 Chr1A 75309072

N00002 13600000 13800000 0.8129921 scaffold_8 21828258 22023905 0.00386746659931 0.000998129288605 0.00389383144451 0.00101806605549 0.374590023396 0.375664401168 0.066925 0.0407775227834 0.01405 0.0262513608693 0.048 Chr1A 75309072

N00002 13800000 14000000 1.001439 scaffold_8 21629705 21828258 0.00397440382777 0.0010095512232 0.00400709531138 0.00104209600251 0.364015017847 0.363468624585 0.073935 0.0698906589173 0.01285 0.0447235750656 0.06 Chr1A 75309072

N00002 14000000 14200000 2.806557 scaffold_8 21433730 21629705 0.00388927581324 0.000916187010663 0.00388927581324 0.000916187010663 0.378046973401 0.379938604671 0.04403 0.0358055874474 0 0 NA Chr1A 75309072

N00002 14200000 14400000 2.806557 scaffold_8 21238358 21433730 0.00383435757343 0.00105883879669 0.00383435757343 0.00105883879669 0.395315928039 0.396863493795 0.080755 0.0668058882542 0 0 NA Chr1A 75309072

N00002 14400000 14600000 2.88594 scaffold_8 21048913 21238358 0.00417217400387 0.000994761640356 0.00428152364821 0.00103581924861 0.384424473277 0.390654876387 0.07038 0.0454168756103 0.03812 0.0444508960384 0.0876666666667 Chr1A 75309072

N00002 14600000 14800000 4.579439 scaffold_8 20852880 21048913 0.00420325308884 0.00104218642453 0.00422394132762 0.0010484914168 0.375885189717 0.378151169701 0.068895 0.0657542352563 0.005035 0.00563170486602 NA Chr1A 75309072

N00002 14800000 15000000 0.1366997 scaffold_8 20662209 20852880 0.00446096376474 0.00133236107061 0.00450577692731 0.00134491168934 0.355697681569 0.352140319093 0.05883 0.0480251322959 0.011395 0.0119525255545 NA Chr1A 75309072

N00002 15200000 15400000 0 scaffold_8 20273566 20473259 0.00462062378319 0.00132501271363 0.0046727235435 0.0013433939639 0.38647184282 0.386299161831 0.051615 0.0466015333537 0.017785 0.0201909931745 0.038 Chr1A 75309072

N00002 15400000 15600000 2.826983 scaffold_8 20086346 20273566 0.00429773325422 0.000837472608866 0.00448861797552 0.000885914996299 0.402842665133 0.398393840535 0.06361 0.0392479435958 0.058155 0.0664993056297 0.068 Chr1A 75309072

N00002 15600000 15800000 6.244216 scaffold_8 19884819 20086346 0.00442400653369 0.00126628690746 0.00462870025111 0.00131911827654 0.393144158006 0.39191069148 0.06914 0.0570891245342 0.053575 0.0550745061456 0.0583333333333 Chr1A 75309072

N00002 15800000 16000000 3.075509 scaffold_8 19685141 19884819 0.00446326583497 0.00108470384856 0.00455706377638 0.00112116787908 0.435823013791 0.44183869098 0.054815 0.0369394725508 0.048275 0.0618045052535 0.182714285714 Chr1A 75309072

N00002 16200000 16400000 0.8830767 scaffold_8 19297877 19491972 0.00368668064745 0.000823191645116 0.00373434450916 0.000833949384109 0.451740441164 0.453442082804 0.05439 0.0357608387645 0.01989 0.0217934516603 0.068 Chr1A 75309072

N00002 16400000 16600000 0.9699367 scaffold_8 19110694 19297877 0.00353724820717 0.000722194160861 0.00356927440759 0.000731959693865 0.440397951719 0.443291013336 0.104645 0.0614051489719 0.02049 0.0219090408851 0.05 Chr1A 75309072

N00002 16800000 17000000 2.204635 scaffold_8 18722181 18919775 0.00378303531851 0.000804214029351 0.00391333030191 0.000840436074776 0.38672409972 0.384858026363 0.070445 0.052769820946 0.04349 0.0524054374121 0.0703333333333 Chr1A 75309072

N00002 17000000 17200000 3.112664 scaffold_8 18519444 18722181 0.00377900635577 0.000813464072832 0.00388425665086 0.000854508106527 0.424331310407 0.421001003484 0.06423 0.0307639947321 0.049425 0.0642211337842 0.082875 Chr1A 75309072

N00002 17400000 17600000 4.308703 scaffold_8 18154157 18344213 0.00385319362768 0.00110433663266 0.00401152109358 0.00116521714961 0.490573051878 0.495996735891 0.08405 0.032285221198 0.069265 0.0755935092815 0.1204 Chr1A 75309072

N00002 17600000 17800000 4.308703 scaffold_8 17965121 18154157 0.00356652727264 0.000858705422781 0.00368663036198 0.000897237718934 0.462523223052 0.464213593948 0.096965 0.0368924437673 0.053945 0.0566400050784 0.06175 Chr1A 75309072

N00002 18000000 18200000 0 scaffold_8 17597051 17789756 0.00406818608176 0.00101734642416 0.00411658812056 0.00103043375493 0.405011537514 0.403116019578 0.060295 0.0340520484679 0.0193 0.0202329986248 0.05925 Chr1A 75309072

N00002 18200000 18400000 2.535836 scaffold_8 17401444 17597051 0.00397354032605 0.00101967245997 0.00412184976499 0.00106557658989 0.406055824614 0.403490437275 0.066675 0.0444462621481 0.057995 0.0614957542419 0.0643333333333 Chr1A 75309072

N00002 18400000 18600000 4.01341 scaffold_8 17210583 17401444 0.0040578310683 0.00104613782008 0.00407234255125 0.00105756505491 0.423622515034 0.427937390077 0.074605 0.0393532466035 0.01846 0.0159383006481 0.0755 Chr1A 75309072

N00002 18600000 18800000 4.178553 scaffold_8 17016459 17210583 0.0040191208701 0.000962521091127 0.00406281869034 0.000977913896183 0.458509087483 0.456994927564 0.07189 0.0419731717871 0.02445 0.0245925284869 0.0873333333333 Chr1A 75309072

N00002 18800000 19000000 10.65217 scaffold_8 16825433 17016459 0.00386145494565 0.000942927485547 0.00392350476242 0.000972420686013 0.500589459085 0.508368067431 0.099 0.0247191481788 0.038275 0.0428528053773 0.0953333333333 Chr1A 75309072

N00002 19000000 19200000 10.65217 scaffold_8 16642847 16825433 0.00370958292755 0.000966865486587 0.00384367146018 0.00101170918017 0.464710173463 0.471935970389 0.099095 0.0325435685102 0.06963 0.0719825178272 0.0793333333333 Chr1A 75309072

N00002 19200000 19400000 10.65217 scaffold_8 16456529 16642847 0.00385663792094 0.000989800820497 0.0039512818373 0.00103176829838 0.499228580462 0.497695423148 0.12522 0.0523030517717 0.062955 0.0741635268734 0.0753333333333 Chr1A 75309072

N00002 19400000 19600000 10.62185 scaffold_8 16261330 16456529 0.00405480494275 0.00100275184499 0.00420989809499 0.00105543057416 0.514298748859 0.516923543689 0.080655 0.0363167844098 0.09615 0.101127567252 0.086625 Chr1A 75309072

N00002 19600000 19800000 7.7338 scaffold_8 16069936 16261330 0.00329532669936 0.000936280258039 0.0033277094937 0.000950892513423 0.506866416979 0.507307532204 0.08563 0.0396511907374 0.0375 0.0431727222379 0.096 Chr1A 75309072

N00002 19800000 20000000 7.7338 scaffold_8 15878903 16069936 0.00393860242678 0.000945251481812 0.00400410102567 0.000983791306744 0.45757570329 0.460553021801 0.099655 0.0480702287039 0.06295 0.0695429585464 0.123888888889 Chr1A 75309072

N00002 20000000 20200000 5.453993 scaffold_8 15689733 15878903 0.00359183248976 0.000921511833708 0.0036482015338 0.000941988345001 0.450232628206 0.450214248196 0.06448 0.0275255061585 0.031345 0.0324470053391 0.0446666666667 Chr1A 75309072

N00002 20200000 20400000 3.90076 scaffold_8 15497238 15689733 0.00346387748434 0.00100238531168 0.00351925833911 0.0010314160315 0.439279746083 0.435717376111 0.069265 0.0236889269851 0.029315 0.0315852359802 0.0295 Chr1A 75309072

N00002 20400000 20600000 5.806452 scaffold_8 15306865 15497238 0.00328842535417 0.00100194672593 0.00332357366474 0.00102148658176 0.464689981416 0.466284694709 0.06407 0.0276719913013 0.03358 0.0344691736748 0.0514 Chr1A 75309072

N00002 20600000 20800000 5.089921 scaffold_8 15113902 15306865 0.0038968465495 0.00101130304909 0.00393280457482 0.0010219082011 0.43317525205 0.432769418556 0.110975 0.0752579510062 0.01373 0.0117535486078 0.077 Chr1A 75309072

N00002 20800000 21000000 4.543094 scaffold_8 14915694 15113902 0.00409957478153 0.00122514740419 0.00409957478153 0.00122514740419 0.407610972117 0.405962204479 0.101965 0.106716176945 0 0 NA Chr1A 75309072

N00002 21000000 21200000 4.543094 scaffold_8 14727735 14915694 0.00412482529219 0.00115346687188 0.00412482529219 0.00115346687188 0.399473590446 0.399487381929 0.12602 0.0742183135684 0 0 NA Chr1A 75309072

N00002 21200000 21400000 4.543094 scaffold_8 14527174 14727735 0.00404675904117 0.00110009639699 0.00406442565071 0.00110750921506 0.388755644865 0.388617337441 0.07313 0.0683432970518 0.00808 0.00862580461805 0.093 Chr1A 75309072

N00002 21400000 21600000 4.543094 scaffold_8 14342465 14527174 0.00354370588463 0.000937126223476 0.00355916637518 0.00094072301759 0.430100161179 0.429826859551 0.11283 0.045114206671 0.00943 0.00991830392672 0.093 Chr1A 75309072

N00002 21600000 21800000 4.731023 scaffold_8 14144886 14342465 0.00373180344364 0.00117461963808 0.0038224385062 0.00120907421806 0.422500236628 0.42268390291 0.057185 0.0314861397213 0.0424 0.0481984421421 0.125333333333 Chr1A 75309072

N00002 21800000 22000000 3.991209 scaffold_8 13944206 14144886 0.00340627405427 0.00127673938068 0.00344665894171 0.00129150573253 0.439689392861 0.436725685103 0.04985 0.0242276260714 0.01982 0.0149890372733 0.049 Chr1A 75309072

N00002 22000000 22200000 3.439353 scaffold_8 13745156 13944206 0.00335780033091 0.00104738128226 0.0034316252511 0.00107601860476 0.408248304448 0.402621743676 0.072485 0.0349912082391 0.028485 0.0292941471992 0.0466666666667 Chr1A 75309072

N00002 22200000 22400000 2.470891 scaffold_8 13553339 13745156 0.0034235857161 0.00120402211385 0.00353361841192 0.00125844739121 0.402727498427 0.40941909801 0.064 0.0214475255061 0.03786 0.0396471637029 0.0526666666667 Chr1A 75309072

N00002 22400000 22600000 1.52373 scaffold_8 13360797 13553339 0.00269144412174 0.000865384607814 0.00270631628934 0.000871962200382 0.423703838166 0.419623288414 0.06873 0.0156225654662 0.00961 0.00881885510694 0.037 Chr1A 75309072

N00002 22600000 22800000 2.410723 scaffold_8 13164441 13360797 0.00253736225235 0.00130835466501 0.00261974035387 0.0013622099261 0.403686364306 0.394479825241 0.065595 0.0373352482226 0.03993 0.0437979995518 NA Chr1A 75309072

N00002 22800000 23000000 9.443499 scaffold_8 12970198 13164441 0.00253777753261 0.00107044060364 0.00255849527399 0.00108810247234 0.403874708336 0.404866829645 0.05173 0.0300139515967 0.01562 0.0171589194977 0.0755 Chr1A 75309072

N00119 600000 800000 0.637105 scaffold_8 10786383 10968600 0.00233849146418 0.00120645842962 0.00241526691218 0.00125044310805 0.406387765374 0.404374797871 0.07631 0.0330978997569 0.03972 0.04418358331 0.0383333333333 Chr1A 75309072

N00119 800000 1000000 0.637105 scaffold_8 10968600 11177905 0.00294547455365 0.0014660661284 0.00298609364064 0.00148891978411 0.44358420228 0.434343722051 0.08056 0.0814696256659 0.023605 0.0225603783952 0.06 Chr1A 75309072

N00119 1000000 1200000 2.437434 scaffold_8 11177905 11387866 0.0027189973343 0.00118647231559 0.00276072883111 0.0012101782543 0.407416304026 0.398510201046 0.060635 0.0336824457876 0.03186 0.0319678416468 0.1625 Chr1A 75309072

N00119 1200000 1400000 0.7277623 scaffold_8 11387866 11577580 0.00248812946339 0.000924032959569 0.00250155364524 0.000931072146635 0.450465423646 0.445923743387 0.06854 0.0289857364243 0.01849 0.0180324066753 0.036 Chr1A 75309072

N00119 1400000 1600000 0.3973412 scaffold_8 11577580 11777567 0.00267427194828 0.00136303535239 0.00268106534993 0.00136872538404 0.415348797288 0.408619990813 0.07043 0.0586588128228 0.004395 0.00522533964708 NA Chr1A 75309072

N00119 1600000 1800000 0.3973412 scaffold_8 11777567 11982077 0.00266661600734 0.00129496949601 0.00276409006107 0.00134117494579 0.39600547921 0.396988112161 0.075425 0.0630238130165 0.041865 0.0383746516063 0.0572 Chr1A 75309072

N00119 1800000 2000000 1.469832 scaffold_8 11982077 12180534 0.00291419513613 0.0011918537692 0.00298028543801 0.00123694387589 0.367113532603 0.366401545143 0.083575 0.0827433650614 0.036835 0.0412079190958 0.1138 Chr1A 75309072

N00119 2000000 2200000 3.568443 scaffold_8 12180534 12379991 0.00221789507839 0.00106432512191 0.00222297459531 0.00106631497784 0.375585358345 0.372840434693 0.07946 0.0686463749079 0.00309 0.00311846663692 0.048 Chr1A 75309072

N00119 2200000 2400000 1.637026 scaffold_8 12379991 12570850 0.00226934359215 0.00113061954265 0.00229015451683 0.00114255817302 0.419938049181 0.420647184092 0.087435 0.0405796949581 0.01259 0.0133711273768 0.045 Chr1A 75309072

N00179 400000 600000 0.09578696 scaffold_8 9012457 9210056 0.000882498152852 0.00133863236342 0.000882399892998 0.00135362030489 0.407457153309 0.395951294442 0.09615 0.0565842944549 0.01194 0.0117763753865 0.0786666666667 Chr1A 75309072

N00179 1000000 1200000 0.09578696 scaffold_8 9567797 9761277 0.000746877219417 0.00142053654888 0.00075609717003 0.00143814176069 0.402624612785 0.395876638048 0.10007 0.0536799669216 0.01473 0.0147767211081 0.0805 Chr1A 75309072

N00157 800000 1000000 0.09578696 scaffold_8 1083656 1290000 0.00220120205055 0.0010019783247 0.00222595557036 0.00101185985181 0.398414342149 0.393405897003 0.08753 0.0940322955841 0.013385 0.0151591517078 0.036 Chr1A 75309072

N00157 1000000 1200000 0.09578696 scaffold_8 1290427 1479043 0.00180309912271 0.000855037616027 0.00180797025021 0.000857533393775 0.413808627795 0.41210936406 0.097705 0.051416634856 0.004225 0.00450120880519 0.03 Chr1A 75309072

N00157 1200000 1400000 0.09578696 scaffold_8 1479043 1676819 0.00105854971229 0.00103774062886 0.00105854971229 0.00103774062886 0.3970023894 0.395763456876 0.10284 0.0680062292695 0 0 NA Chr1A 75309072

N00157 1400000 1600000 0.09578696 scaffold_8 1676819 1880055 0.00100316870359 0.00107796899609 0.00101421097644 0.0011118719108 0.408374309618 0.405381165919 0.08846 0.0874697396131 0.02659 0.0574750536322 0.043 Chr1A 75309072

N00137 0 200000 5.991436 scaffold_8 2187723 2384906 0.00352162481683 0.000977999694262 0.00355240524911 0.00098788656326 0.410649080745 0.404687554618 0.120735 0.0882023298155 0.022035 0.0178869375149 0.1355 Chr1A 75309072

N00137 200000 400000 2.365057 scaffold_8 1986292 2187723 0.00285402226118 0.000938073816931 0.00290044489309 0.000950872160565 0.431412308191 0.422349637388 0.08177 0.0760458916453 0.02253 0.0230550411804 0.0745 Chr1A 75309072

N00137 800000 1000000 1.498155 scaffold_48 5515624 5696184 0.00296888954487 0.00073422043055 0.00296888727519 0.00073422043055 0.396747574492 0.389252507253 0.114225 0.0364144882588 0.00054 0 NA Chr1A 75309072

N00137 1000000 1200000 1.498155 scaffold_48 5696184 5887571 0.00271612648508 0.000571141872503 0.00272126919921 0.000572458269809 0.394519639799 0.389443063472 0.0637 0.0419202976169 0.00188 0.00230945675516 0.053 Chr1A 75309072

N00137 1200000 1400000 1.320932 scaffold_48 5887571 6081945 0.00269853255314 0.000326960563549 0.00271780200249 0.000328982655318 0.393150058272 0.389077174247 0.062045 0.040483809563 0.009035 0.00933252389723 0.053 Chr1A 75309072

N00137 1400000 1600000 0.8260456 scaffold_48 6081975 6278422 0.00328325990998 0.000328315424014 0.00328325990998 0.000328315424014 0.395981402092 0.388651090195 0.093005 0.0878099436489 0 0 NA Chr1A 75309072

N00151 200000 400000 4.95642 scaffold_8 3666882 3856666 0.00393914157372 0.00126072877583 0.00399010958241 0.00129270915685 0.434275805894 0.432945423955 0.12265 0.0807180795009 0.02114 0.0549255996291 0.14 Chr1A 75309072

N00151 600000 800000 3.815172 scaffold_8 3318031 3514472 0.00360041028337 0.00130979391685 0.00360955816061 0.00131374468306 0.391609225423 0.389008199922 0.125295 0.0917425588345 0.00378 0.00386375552965 NA Chr1A 75309072

N00151 800000 1000000 2.422608 scaffold_8 3124486 3318031 0.0037658472068 0.00106512533249 0.00378047064497 0.00106785144462 0.402471628725 0.402251481988 0.117395 0.0683871967759 0.0053 0.0055490971092 0.118 Chr1A 75309072

N00151 1000000 1200000 2.277259 scaffold_8 2922321 3123974 0.00378480819376 0.00125075077591 0.00383728617835 0.00127594920924 0.40554067771 0.402807955875 0.070545 0.0477404253842 0.027045 0.0273588788662 0.065 Chr1A 75309072

N00151 1200000 1400000 5.420483 scaffold_8 2724403 2922321 0.00347984740011 0.00112625349584 0.00347984740011 0.00112763269318 0.409973775665 0.40169768376 0.06637 0.0385058458554 0 0.00138441172607 NA Chr1A 75309072

N00151 1400000 1600000 0 scaffold_8 2524449 2724403 0.0030717792398 0.00096595900879 0.0030717792398 0.000966945409345 0.452832483045 0.448816307414 0.099965 0.0614341298499 0.002965 0.000780179441271 0.023 Chr1A 75309072

N00308 0 200000 7.82412 scaffold_8 4613300 4826036 0.00442517589601 0.0015884061943 0.00444045050086 0.00159956456975 0.408121238889 0.409918492944 0.11194 0.121770645307 0.008285 0.0117046480144 0.089 Chr1A 75309072

N00173 400000 600000 2.640726 scaffold_8 4047103 4241473 0.00412949937643 0.00151185431901 0.0041739096845 0.00152649781241 0.408420063351 0.405939689482 0.11154 0.0972269383135 0.01068 0.0148171014045 0.0455 Chr1A 75309072

N00173 800000 1000000 4.426718 scaffold_8 5170176 5360613 0.00390396333069 0.00124504441698 0.00393044081817 0.00125663579131 0.404159640247 0.405198459374 0.12518 0.0716509921916 0.00759 0.008149676796 0.0365 Chr1A 75309072

N00173 1000000 1200000 7.575901 scaffold_8 5360613 5556086 0.00446866706988 0.00101857351562 0.0044820928441 0.00102370689596 0.397087389356 0.397586884675 0.074735 0.0512346973751 0.00559 0.00676819816548 0.0485 Chr1A 75309072

N00173 1200000 1400000 3.67354 scaffold_8 5556086 5747977 0.00389882870495 0.00109844674812 0.00400277601593 0.00112816418136 0.395474236729 0.394119284452 0.08265 0.0390586322443 0.03805 0.0340141017557 0.043 Chr1A 75309072

N00299 0 200000 8.703524 scaffold_8 6007507 6196445 0.00435994523974 0.00103219770208 0.00443382147771 0.00106128255138 0.418444863505 0.41990648592 0.10545 0.0626290105749 0.045595 0.0354454900549 0.069 Chr1A 75309072

N00299 200000 400000 4.41761 scaffold_8 5803173 6007507 0.00414554676883 0.00104764084679 0.00427702130579 0.00108869486208 0.408271559003 0.40319076108 0.115065 0.0890355985788 0.04363 0.044300018597 0.0944 Chr1A 75309072

N00184 200000 400000 3.752309 scaffold_8 7069024 7268340 0.00474012140415 0.00125284689195 0.00474012140415 0.00125284689195 0.430453013932 0.433425180388 0.067135 0.049980934797 0 0 NA Chr1A 75309072

N00184 400000 600000 9.177123 scaffold_8 6863047 7069024 0.00406910323609 0.00111698638356 0.00409253549509 0.00112685273611 0.446239611002 0.445404818337 0.099395 0.0495249469601 0.01646 0.0140306927472 0.0835 Chr1A 75309072

N00184 600000 800000 6.614309 scaffold_8 6665873 6863047 0.0035251177057 0.000924004740996 0.00364257562336 0.000952808558666 0.413152023797 0.409439409423 0.067385 0.054515301206 0.037075 0.0365666872914 NA Chr1A 75309072

N00184 800000 1000000 1.935323 scaffold_8 6468274 6665873 0.00428636191151 0.00103856163262 0.00436021041994 0.00106409209079 0.425304625577 0.429864537646 0.08996 0.0590792463525 0.035065 0.0372319697974 0.065 Chr1A 75309072

N00133 200000 400000 3.965625 scaffold_8 7938257 8144832 0.004052332244 0.00112094719863 0.00413219096561 0.00114828061305 0.422104098846 0.420161286013 0.0365 0.0348735326153 0.02787 0.0299455403606 0.044 Chr1A 75309072

N00133 400000 600000 5.033147 scaffold_8 8144832 8336221 0.00414789161168 0.00108507904779 0.00431739155495 0.00112442630554 0.44316074306 0.445512170083 0.098665 0.0447935879283 0.062485 0.0699413236915 0.0668333333333 Chr1A 75309072

N00133 800000 1000000 3.51773 scaffold_8 8413494 8616957 0.00547563158191 0.00137698140922 0.00548498573542 0.00138177040544 0.422379222742 0.425230241329 0.050555 0.0573126317807 0.00442 0.00459051522881 0.075 Chr1A 75309072

N00133 1000000 1200000 3.127903 scaffold_48 10315 204396 0.0041503636671 0.00093269751319 0.00420300792024 0.000950341524556 0.448551118932 0.443447559245 0.07016 0.0545906090756 0.03497 0.0468206573544 0.089 Chr1A 75309072

N00133 1200000 1400000 2.789928 scaffold_48 204396 411717 0.00519979424923 0.00124645415858 0.00537086808651 0.00130451519577 0.45872548239 0.449275041628 0.037255 0.0248551762725 0.054045 0.0562027001606 0.106 Chr1A 75309072

N00133 1400000 1600000 6.972197 scaffold_48 411717 603023 0.00474106894806 0.00122514055815 0.00478439985092 0.00124078960301 0.441354360975 0.440504794322 0.05556 0.0439975745664 0.02388 0.0233500256134 0.101 Chr1A 75309072

N00133 1600000 1800000 4.385497 scaffold_48 603023 799923 0.00525488791516 0.00126008350224 0.00538347731992 0.0013032151517 0.39479194078 0.393094774196 0.051825 0.0479888268156 0.052295 0.0553326561706 0.119666666667 Chr1A 75309072

N00133 1800000 2000000 5.684809 scaffold_48 799923 992403 0.00483334303468 0.00136778648248 0.00486300283355 0.00137455579978 0.414669655955 0.412674466561 0.07131 0.0433239817124 0.004995 0.00535120532003 0.153 Chr1A 75309072

N00225 0 200000 3.79661 scaffold_48 1734923 1932243 0.00432294570121 0.000991921838267 0.00438017936204 0.00102374346481 0.475601946002 0.473658398675 0.076845 0.0235049665518 0.049505 0.0414149604703 0.15025 Chr1A 75309072

N00225 200000 400000 5.615215 scaffold_48 1527539 1734923 0.00439961571894 0.0012640345514 0.00448580681595 0.00129348779814 0.432591612813 0.433375163684 0.05311 0.0427950082938 0.03026 0.0286714500637 0.072 Chr1A 75309072

N00225 400000 600000 7.799059 scaffold_48 1323640 1527539 0.00549586200826 0.00150915373933 0.00550365733477 0.00151164480783 0.410987811908 0.401681202509 0.062715 0.0443503891633 0.002125 0.00278569291659 0.072 Chr1A 75309072

N00225 600000 800000 5.023341 scaffold_48 1131616 1323640 0.00429979571927 0.00138029367766 0.00434596682867 0.00140360863888 0.438135690506 0.428473540546 0.076405 0.0412708828063 0.025185 0.0273767862351 0.09 Chr1A 75309072

N00249 0 200000 9.458901 scaffold_48 2386575 2589436 0.00520419049773 0.00137271562917 0.00524204542476 0.00138311635907 0.415968475233 0.417591945334 0.064435 0.0348021551703 0.01548 0.0164694051592 0.2185 Chr1A 75309072

N00249 400000 600000 3.136132 scaffold_48 1983708 2176584 0.00493986269038 0.00131329009294 0.0050322514783 0.00135390894416 0.420983625329 0.418226408784 0.05457 0.0479997511354 0.03118 0.0381540471598 0.124 Chr1A 75309072

N00313 0 200000 1.557691 scaffold_48 2602954 2813227 0.00539739789399 0.00123648709298 0.00540219678092 0.00123809430669 0.419782119312 0.418910102606 0.07141 0.0460163691962 0.00096 0.00285343339373 NA Chr1A 75309072

N00223 0 200000 8.224989 scaffold_48 3619951 3824579 0.00490893438566 0.00128862153431 0.00490893438566 0.00128862153431 0.387052723446 0.403403755869 0.088295 0.0480774869519 0 0 NA Chr1A 75309072

N00223 200000 400000 7.47663 scaffold_48 3420442 3619951 0.00454743359327 0.00111605295304 0.00454743359327 0.00111605295304 0.38806233858 0.395580682275 0.08647 0.0354620593557 0 0 NA Chr1A 75309072

N00223 400000 600000 3.035195 scaffold_48 3225474 3420442 0.00405649894468 0.00115205263751 0.00408434196695 0.00116149355862 0.398373230766 0.401021403385 0.051785 0.040314307989 0.012775 0.0140638463748 0.102333333333 Chr1A 75309072

N00223 600000 800000 3.663644 scaffold_48 3028095 3225474 0.004447882254 0.00130468809561 0.00448114886883 0.00131367622621 0.414429063289 0.414383768664 0.077875 0.0561305914003 0.00997 0.0101530557962 0.0886666666667 Chr1A 75309072

N00208 400000 600000 9.813358 scaffold_48 4381604 4575977 0.00532395902432 0.00134467165474 0.00541866314561 0.00137178156779 0.389278814142 0.432634022376 0.070245 0.0259140930067 0.02068 0.0210986093748 0.2375 Chr1A 75309072

N00208 600000 800000 7.417857 scaffold_48 4172268 4380570 0.00576337198785 0.00126149565061 0.00577521364056 0.00126295365189 0.387026151028 0.413532417603 0.05921 0.0409789632361 0.00171 0.00102255379209 0.298 Chr1A 75309072

N00004 600000 800000 3.340247 scaffold_20 3745425 3933163 0.00421855814393 0.00122455969569 0.00441138543424 0.00127367307759 0.461315834369 0.459484811314 0.102945 0.0547411818598 0.063125 0.0683079610947 0.0753333333333 Chr2 157769492

N00004 800000 1000000 3.727273 scaffold_20 3933163 4121078 0.00391993573196 0.00110957907038 0.00396018693944 0.00112458617642 0.452447903297 0.455704500381 0.0695 0.0537157757497 0.021445 0.0209509618711 0.075 Chr2 157769492

N00004 1200000 1400000 6.3705 scaffold_20 4295381 4478604 0.00365803028939 0.00114450635184 0.0036622112459 0.00114751161349 0.474931951362 0.482113147204 0.1395 0.0628141663437 0.014165 0.00934926292005 0.094 Chr2 157769492

N00004 1400000 1600000 4 scaffold_20 4478604 4688920 0.00424271837406 0.00128860765476 0.00445689984434 0.00137785725871 0.387075180227 0.387283236994 0.09556 0.0894986591605 0.065025 0.0718918199281 0.06725 Chr2 157769492

N00004 1600000 1800000 4.989297 scaffold_20 4688920 4883752 0.00378942748326 0.00103489730555 0.00386182123126 0.00105869387732 0.416668503538 0.417300526931 0.06533 0.0412149954833 0.02688 0.0273774328652 0.049 Chr2 157769492

N00004 1800000 2000000 5.015122 scaffold_20 4883752 5068263 0.00394800499362 0.00110978622589 0.0039901340224 0.0011266127562 0.441533695646 0.443173466174 0.12671 0.0773775005284 0.016095 0.0184216659169 0.0776666666667 Chr2 157769492

N00004 2000000 2200000 3.464738 scaffold_20 5068263 5252355 0.00364066533718 0.0010138584057 0.00364066533718 0.0010138584057 0.439493290089 0.445686716833 0.08901 0.0495078547683 0 0 NA Chr2 157769492

N00004 2200000 2400000 3.881713 scaffold_20 5252355 5448437 0.00370328303799 0.00111701562854 0.00371329156643 0.00111941253126 0.427915833993 0.431540678488 0.08723 0.0733927642517 0.003635 0.00312624310238 NA Chr2 157769492

N00004 2400000 2600000 5.227273 scaffold_20 5448437 5637421 0.00353110253744 0.00107073353471 0.00360182980146 0.00110998647219 0.415921414817 0.419203508812 0.082295 0.049840198112 0.03336 0.0364951530288 0.06125 Chr2 157769492

N00004 2600000 2800000 1.482361 scaffold_20 5637421 5836837 0.00389226580651 0.00115755204228 0.0038973462493 0.00115976282022 0.419351445731 0.41527711947 0.05721 0.0335028282585 0.003825 0.00445300276808 0.01 Chr2 157769492

N00004 2800000 3000000 0 scaffold_20 5836837 6031702 0.00321628690798 0.001009883217 0.00326621032581 0.00106646364077 0.393513564365 0.396992091988 0.053725 0.0388833294845 0.032805 0.084032535345 0.074 Chr2 157769492

N00004 3000000 3200000 0.5384029 scaffold_20 6031702 6228318 0.00329556285292 0.00114292259366 0.00332363266076 0.00115604713831 0.40895163985 0.411010537619 0.06687 0.055468527485 0.01033 0.0119268014811 0.0535 Chr2 157769492

N00004 3200000 3400000 2.576642 scaffold_20 6228318 6421311 0.00418393108743 0.00123420332975 0.00418393108743 0.00123420332975 0.409483239912 0.408666463229 0.098825 0.0695828346106 0 0 NA Chr2 157769492

N00004 3400000 3600000 2.071282 scaffold_20 6421311 6609934 0.00434400047631 0.00135051416885 0.00434400047631 0.00135051416885 0.411088908179 0.414087966064 0.103865 0.059754112701 0 0 NA Chr2 157769492

N00004 3600000 3800000 1.972015 scaffold_20 6609934 6800377 0.0041388811057 0.00122892807437 0.0041388811057 0.00122926457759 0.405600600346 0.410677495931 0.075305 0.0568569073161 0 0.000267796663569 NA Chr2 157769492

N00004 3800000 4000000 2.090816 scaffold_20 6800377 6992486 0.00423827807036 0.00117305904348 0.00424691933801 0.00117792215691 0.407699505414 0.412122529967 0.07668 0.05926323077 0.003485 0.00362814860314 0.055 Chr2 157769492

N00004 4000000 4200000 2.524855 scaffold_20 6992486 7183663 0.0039876507228 0.00112745291628 0.00408308768127 0.00115492749557 0.422178932096 0.430562262849 0.10146 0.049922323292 0.02974 0.031520528097 0.0606666666667 Chr2 157769492

N00004 4400000 4600000 7.855718 scaffold_20 7378518 7567286 0.0036906649575 0.0012142370922 0.00371477697849 0.00123239151108 0.405211981097 0.406035200711 0.08218 0.0409073571792 0.01867 0.0194206645194 0.072 Chr2 157769492

N00004 4600000 4800000 3.750965 scaffold_20 7567286 7751395 0.00353791376446 0.000888519239874 0.00353900266662 0.000889453055882 0.413588840027 0.417787969739 0.095985 0.0534031470488 0.00346 0.00384554801775 NA Chr2 157769492

N00004 4800000 5000000 0.2054632 scaffold_20 7751395 7951310 0.00367663808335 0.00104731854314 0.00368351038564 0.00105279617776 0.394263056746 0.395763437316 0.055995 0.0456143861141 0.003565 0.00696295925768 0.036 Chr2 157769492

N00004 5000000 5200000 0.2054632 scaffold_20 7951310 8141927 0.00427083001614 0.00114209216547 0.00435246162515 0.00116989004185 0.389722388396 0.394342865192 0.08247 0.0701878636218 0.031885 0.0294989429065 0.0476666666667 Chr2 157769492

N00004 5200000 5400000 0.2054632 scaffold_20 8141927 8335578 0.00324722653943 0.000936069832751 0.0032704669276 0.000944135768466 0.409679376381 0.410118695356 0.09464 0.082075486313 0.010015 0.0103691692788 0.063 Chr2 157769492

N00004 5400000 5600000 0.5684232 scaffold_20 8335578 8530332 0.00304097234759 0.000942377468372 0.00305007845403 0.000946437107872 0.388798584619 0.393914636165 0.058045 0.0385101204597 0.00557 0.00507306653522 0.08 Chr2 157769492

N00004 5600000 5800000 1.128944 scaffold_20 8530765 8730559 0.00331979678585 0.000903044874533 0.00331805133222 0.000906161909471 0.373768944018 0.373966212674 0.058445 0.0694265093046 0.00681 0.00699720712334 0.08 Chr2 157769492

N00004 5800000 6000000 1.128944 scaffold_20 8730559 8927487 0.00405276031622 0.000920435919898 0.0040536249957 0.000921690628656 0.378077421227 0.37670749125 0.04331 0.0514655102372 0.000975 0.00233080110497 0.08 Chr2 157769492

N00004 6000000 6200000 1.128944 scaffold_20 8927818 9131173 0.00359874633536 0.00125503554088 0.00368132434023 0.00128903240626 0.372263190991 0.373436163014 0.07717 0.0894544023997 0.028875 0.0301885864621 0.104 Chr2 157769492

N00004 6200000 6400000 1.128944 scaffold_20 9131173 9338467 0.00442526030298 0.00121223622546 0.00447762554818 0.00122440967749 0.375854783256 0.374872724425 0.085095 0.0843680955551 0.02182 0.0209991606125 NA Chr2 157769492

N00004 6400000 6600000 1.128944 scaffold_20 9338505 9528189 0.00337982508337 0.000950056755279 0.00342905485621 0.000961203187211 0.390866198411 0.38831510789 0.081545 0.0493821302798 0.012665 0.0131481832943 NA Chr2 157769492

N00004 6600000 6800000 1.128944 scaffold_20 9528189 9724954 0.00352660048976 0.00105524534014 0.00357865175417 0.00107481796861 0.39659399209 0.393127302081 0.044895 0.047137448225 0.01981 0.0199832287246 NA Chr2 157769492

N00004 6800000 7000000 1.128944 scaffold_20 9724954 9917959 0.00377118339065 0.000998557468835 0.00379844331644 0.00101065196224 0.411168491046 0.409976639188 0.099105 0.0615217222352 0.01047 0.0108650034973 NA Chr2 157769492

N00004 7000000 7200000 1.128944 scaffold_20 9917959 10113085 0.00379516950803 0.00107815483862 0.00386106237206 0.00110579004538 0.406198140673 0.409352529988 0.07939 0.0562662074762 0.021915 0.0268851921323 0.0623333333333 Chr2 157769492

N00004 7200000 7400000 1.128944 scaffold_20 10113085 10301441 0.00343122446909 0.000992866872511 0.00343991974002 0.000998071678667 0.408613409706 0.410664440843 0.070405 0.0507443351951 0.00723 0.0086856803075 0.124 Chr2 157769492

N00004 7400000 7600000 1.128944 scaffold_20 10301441 10495700 0.00377836182769 0.00103425954121 0.00377836182769 0.00103425954121 0.38722986912 0.390436019369 0.07884 0.0725938051776 0 0 NA Chr2 157769492

N00004 7600000 7800000 0.7772374 scaffold_20 10495700 10690621 0.00458016117135 0.00119137666049 0.00458016117135 0.00119137666049 0.365554799183 0.367186985444 0.07632 0.0846291574535 0 0 NA Chr2 157769492

N00004 7800000 8000000 0.3605419 scaffold_20 10692539 10890297 0.00496306293462 0.00103194998387 0.00496306293462 0.00103194998387 0.359138723612 0.361079000205 0.095845 0.0839106382548 0 0 NA Chr2 157769492

N00004 8200000 8400000 0.3605419 scaffold_20 11086127 11280502 0.00395629770663 0.00111940116127 0.00405077722748 0.00115015901359 0.378676321035 0.381219687186 0.10214 0.0798919614148 0.02661 0.0275498392283 0.07 Chr2 157769492

N00004 8400000 8600000 0.3605419 scaffold_20 11280502 11476474 0.00388820717902 0.00102050602043 0.00388820717902 0.00102050602043 0.384525824431 0.386020878597 0.056435 0.0533239442369 0 0 NA Chr2 157769492

N00004 8600000 8800000 0.3605419 scaffold_20 11476474 11665396 0.00369490002111 0.000914842648878 0.00370409635899 0.000918208111804 0.380494087861 0.387041446279 0.057055 0.0337017393422 0.00424 0.00450979769429 NA Chr2 157769492

N00004 8800000 9000000 0.3605419 scaffold_20 11665396 11859633 0.0044452700563 0.00104827637056 0.0044452700563 0.00104827637056 0.366953166111 0.36905954003 0.08099 0.0870019615212 0 0 NA Chr2 157769492

N00004 9000000 9200000 0.3605419 scaffold_20 11859633 12060702 0.0046801508104 0.00103127909486 0.0046801508104 0.00103127909486 0.363760585897 0.36700144253 0.07918 0.0763568725164 0 0 NA Chr2 157769492

N00004 9200000 9400000 0.3605419 scaffold_20 12060702 12254206 0.0043818902734 0.000970143374069 0.0043818902734 0.000970143374069 0.36282795869 0.36630770836 0.08894 0.0802980816934 0 0 NA Chr2 157769492

N00004 9400000 9600000 0.3605419 scaffold_20 12254206 12450033 0.00459278525127 0.000974803374776 0.00459278525127 0.000974803374776 0.378921610993 0.381024997485 0.055705 0.0531846987392 0 0 NA Chr2 157769492

N00004 9600000 9800000 0.3605419 scaffold_20 12450033 12649711 0.00419842939181 0.00107113135613 0.00421868067346 0.00108186655941 0.385329117311 0.386165091817 0.0563 0.0456885585793 0.018265 0.0151794388966 0.0673333333333 Chr2 157769492

N00004 9800000 10000000 0.3605419 scaffold_20 12649711 12852712 0.0037000325088 0.00104927372649 0.0037550065723 0.00106686033737 0.376327685793 0.374815037326 0.084655 0.0642361367678 0.016105 0.0172560726302 0.027 Chr2 157769492

N00004 10000000 10200000 0.3605419 scaffold_20 12852712 13044500 0.00381726501139 0.000990900278987 0.00381949702072 0.00099195639227 0.390022877196 0.387847919962 0.063495 0.0518176319686 0.00092 0.00177279078983 0.057 Chr2 157769492

N00004 10200000 10400000 0.3605419 scaffold_20 13044500 13240495 0.0041239005587 0.00101779730901 0.00417262331263 0.0010308075802 0.376001382563 0.374597724655 0.05431 0.0623281206153 0.013775 0.0145666981301 0.057 Chr2 157769492

N00004 10400000 10600000 0.5587534 scaffold_20 13240495 13435490 0.00363664147978 0.000667364404471 0.0036476540536 0.000669216081973 0.382641997201 0.384628661088 0.034845 0.0352573142901 0.004455 0.00460524628837 0.057 Chr2 157769492

N00004 10600000 10800000 3.425197 scaffold_20 13435490 13629556 0.00365803217984 0.000766502929435 0.00365803217984 0.000766744606276 0.398390982478 0.399420068018 0.072215 0.0517401296466 0 0.000252491420445 NA Chr2 157769492

N00004 10800000 11000000 4.744415 scaffold_20 13629556 13818775 0.00358680049891 0.000792944717025 0.0036262213812 0.000803508522163 0.399785046833 0.405807797208 0.0461 0.0236339902441 0.01381 0.0154371389765 0.063 Chr2 157769492

N00004 11000000 11200000 2.019231 scaffold_20 13818775 14018951 0.00413966892618 0.000980463675753 0.00424725748976 0.00101484594355 0.380800930974 0.381358744345 0.06691 0.0624850131884 0.02902 0.0341999040844 0.043 Chr2 157769492

N00004 11200000 11400000 2.661884 scaffold_20 14018951 14212085 0.00380479726889 0.00087364931378 0.00389715886825 0.000897972441588 0.410267272373 0.41688577113 0.055435 0.0383153665331 0.023835 0.0260337382336 0.051 Chr2 157769492

N00004 11400000 11600000 2.71102 scaffold_20 14212085 14411110 0.00414998382238 0.00112189920363 0.00417962262084 0.00113390096913 0.394216242254 0.394992609845 0.090345 0.0583645270695 0.01046 0.0106619771386 0.059 Chr2 157769492

N00004 11600000 11800000 2.012985 scaffold_20 14411110 14601841 0.00382194746469 0.00117358940183 0.00395211350769 0.0012318075331 0.392084742379 0.391689968286 0.09055 0.0764689536572 0.04373 0.0494099019037 0.102 Chr2 157769492

N00004 11800000 12000000 0 scaffold_20 14601841 14797012 0.00399706600879 0.00103178496909 0.00402288437978 0.00104145316727 0.421039694548 0.42601583085 0.05117 0.0345850561815 0.020855 0.0210994461267 0.085 Chr2 157769492

N00004 12000000 12200000 4.576719 scaffold_20 14797012 14988690 0.00416416112781 0.000949765863993 0.00421646165745 0.000967623977347 0.443079565826 0.448706950073 0.098835 0.0642535919615 0.015275 0.0160894834045 0.08 Chr2 157769492

N00004 12200000 12400000 0.9766355 scaffold_20 14988690 15180633 0.00404755684728 0.000992549833869 0.00404755684728 0.000992549833869 0.426361369104 0.435190596966 0.06753 0.051885195084 0 0 NA Chr2 157769492

N00004 12600000 12800000 0.9766355 scaffold_20 15373219 15573684 0.00356237424154 0.00079898692806 0.00360694626103 0.000810047903045 0.36566427289 0.365206812652 0.069855 0.0795949417604 0.0189 0.0186466465468 0.061 Chr2 157769492

N00004 12800000 13000000 0.9766355 scaffold_20 15573684 15765127 0.00352345197676 0.000910483256614 0.00355001847221 0.000922145009323 0.398304221334 0.404674099716 0.05752 0.0451570441332 0.0138 0.0137429939982 0.0685 Chr2 157769492

N00004 13200000 13400000 4.726825 scaffold_3 43516963 43713206 0.00392213534654 0.000983041580949 0.00400062830404 0.00100708036642 0.392406763723 0.390890493651 0.07539 0.05023873463 0.030235 0.0278736056828 0.039 Chr2 157769492

N00004 13400000 13600000 2.395709 scaffold_3 43321563 43516963 0.00467851081898 0.00102248600428 0.00478045149601 0.00106241010505 0.397034107761 0.399527359685 0.07894 0.0575588536336 0.030865 0.0422722620266 0.0496666666667 Chr2 157769492

N00004 13600000 13800000 2.395709 scaffold_3 43125491 43321563 0.00444271591069 0.00110330247527 0.00450620342371 0.00111451693074 0.399780370929 0.401140363763 0.066385 0.0602023746379 0.014595 0.0154994083806 0.0495 Chr2 157769492

N00004 13800000 14000000 2.395709 scaffold_3 42924218 43125491 0.00426002609955 0.00116194223402 0.00427132726388 0.00116418180877 0.398437585779 0.394767658044 0.091685 0.0793847162809 0.00737 0.00710477808747 0.04 Chr2 157769492

N00004 14000000 14200000 2.395709 scaffold_3 42709421 42924218 0.00387112779036 0.000948712734695 0.0039463023764 0.000974098912926 0.378229781091 0.379168223621 0.07388 0.0539672341792 0.02885 0.0272163950148 0.0913333333333 Chr2 157769492

N00004 14200000 14400000 2.395709 scaffold_3 42515930 42709421 0.00401722092239 0.000898568061795 0.00414376253509 0.000934972002384 0.395672118994 0.395726673448 0.062935 0.0532686274814 0.047845 0.0493046188195 0.1 Chr2 157769492

N00004 14400000 14600000 2.395709 scaffold_3 42316312 42515930 0.00405413351879 0.00108495086908 0.00406066436916 0.00108718524559 0.393257092897 0.395503159672 0.09781 0.0555360739012 0.00178 0.00289052089491 0.1 Chr2 157769492

N00004 14800000 15000000 2.395709 scaffold_3 41936285 42128393 0.00452539358378 0.000926582294453 0.0045776389256 0.000935787990799 0.376436982628 0.37564622287 0.10829 0.084358798176 0.013455 0.0146063672518 0.086 Chr2 157769492

N00004 15000000 15200000 2.395709 scaffold_3 41747565 41934999 0.00374399890344 0.000979967965314 0.00381206573331 0.000999596114347 0.39327540148 0.403592848252 0.095535 0.0454453300895 0.01867 0.021863696021 0.08775 Chr2 157769492

N00004 15200000 15400000 2.430384 scaffold_3 41555323 41747565 0.00351744461444 0.00107441413371 0.00356272290515 0.00108787150846 0.375439637985 0.375714646271 0.07455 0.0487302462521 0.01355 0.0143360972108 0.034 Chr2 157769492

N00004 15400000 15600000 2.606913 scaffold_3 41363424 41555323 0.00333847112111 0.000900149666077 0.00339218955064 0.000917335752831 0.38543696241 0.389303258437 0.109025 0.0627361268167 0.02067 0.0201095367876 0.033 Chr2 157769492

N00004 15600000 15800000 2.606913 scaffold_3 41167689 41363424 0.00402582436215 0.00110406245809 0.00402840020546 0.00110563068455 0.374829504615 0.374476882246 0.103525 0.0711778680359 0.0016 0.00131810866733 0.049 Chr2 157769492

N00004 15800000 16000000 2.606913 scaffold_3 40976930 41167689 0.00466400625298 0.00109160834334 0.00472537767262 0.00110805340937 0.372396421633 0.371654000943 0.061855 0.0577482582735 0.01446 0.014043898322 0.049 Chr2 157769492

N00004 16000000 16200000 2.606913 scaffold_3 40771704 40976930 0.0048894276286 0.00111678719375 0.00491642602819 0.00112357128455 0.368834710081 0.370255204581 0.070235 0.0800483369554 0.00673 0.00723592527263 0.055 Chr2 157769492

N00004 16200000 16400000 2.606913 scaffold_3 40572332 40771704 0.00450784242199 0.00121713076407 0.004512202419 0.00122075142944 0.373018463514 0.373660748346 0.078155 0.0651395381498 0.00433 0.0044339225167 0.165 Chr2 157769492

N00004 16400000 16600000 2.606913 scaffold_3 40376934 40572332 0.00525879419489 0.00144964438537 0.00528288606305 0.00147834580686 0.353837237712 0.358669448558 0.06372 0.0716537528532 0.01125 0.0276256665882 0.16 Chr2 157769492

N00004 16800000 17000000 2.606913 scaffold_3 39983354 40172730 0.00418075004057 0.00112327431155 0.00418535367054 0.00112417528747 0.379109299552 0.382222972783 0.110155 0.081625971612 0.00094 0.000807916525853 0.025 Chr2 157769492

N00004 17000000 17200000 0.9727286 scaffold_3 39778534 39983354 0.00433211867729 0.00131797052029 0.0045141913634 0.00140835705684 0.370506711621 0.372752570374 0.079335 0.0687384044527 0.06814 0.0761497900596 0.078 Chr2 157769492

N00004 17200000 17400000 0 scaffold_3 39577546 39778534 0.00407630304593 0.00121760835288 0.00424251549753 0.00127049515281 0.374974909531 0.372112932175 0.10376 0.0700390073039 0.050005 0.0509831432722 0.0565 Chr2 157769492

N00004 17400000 17600000 1.40844 scaffold_3 39379613 39577546 0.00368480986026 0.00114763551954 0.003780190748 0.00116899210353 0.394250398501 0.390564720989 0.06414 0.0529421571946 0.040285 0.0414584733218 0.0625 Chr2 157769492

N00004 17600000 17800000 1.451777 scaffold_3 39179862 39379613 0.004003502351 0.00116332680109 0.00410486745271 0.00119577656102 0.395723793768 0.394061519693 0.044545 0.0417269500528 0.035 0.0346030808356 0.0645 Chr2 157769492

N00004 17800000 18000000 1.502438 scaffold_3 38976059 39179862 0.00331482209012 0.00105429486442 0.00333514500312 0.00106137755645 0.39074366349 0.39244202561 0.104415 0.0825257724371 0.010695 0.0113737285516 0.045 Chr2 157769492

N00004 18000000 18200000 3.997519 scaffold_3 38780319 38976059 0.00411397162105 0.00120190914764 0.00417385451735 0.00122006833208 0.377980960093 0.371971587637 0.06836 0.0661540819454 0.02002 0.0196536221518 0.0285 Chr2 157769492

N00004 18400000 18600000 0.6934524 scaffold_3 38394034 38598769 0.00407775246017 0.00118695115182 0.00423350659904 0.0012436870476 0.359429225542 0.358480785584 0.09153 0.109111778641 0.05 0.0529806823455 0.0923333333333 Chr2 157769492

N00004 18600000 18800000 0.8814873 scaffold_3 38198447 38394034 0.0045641303604 0.000971720378456 0.00464252686348 0.000990411819905 0.389084662144 0.388030919759 0.06483 0.043750351506 0.02248 0.0241120319858 0.076 Chr2 157769492

N00004 18800000 19000000 1.274914 scaffold_3 37993580 38198447 0.00465049319096 0.00132689673186 0.00465049319096 0.00132689673186 0.382155998276 0.381199141274 0.121955 0.0967603371944 0 0 NA Chr2 157769492

N00004 19000000 19200000 1.274914 scaffold_3 37795359 37993580 0.0045788508417 0.00122765670265 0.00461585184855 0.00124519503617 0.3751567023 0.373452063819 0.115205 0.0864086045374 0.019485 0.0205074134426 0.113 Chr2 157769492

N00215 0 200000 0 scaffold_3 37471723 37669888 0.00426298535338 0.00118407789096 0.00433325182385 0.00121044265954 0.370135537381 0.370002338754 0.086245 0.0878510332299 0.02554 0.0262659904625 0.068 Chr2 157769492

N00215 200000 400000 0.5972598 scaffold_3 37277770 37471723 0.0042673902932 0.00102220455407 0.00429469466646 0.00103387460425 0.382915392925 0.384826006581 0.118625 0.0728527014277 0.015385 0.0154161059638 0.059 Chr2 157769492

N00215 400000 600000 0.8514129 scaffold_3 37082473 37277770 0.00424789175743 0.00108594887812 0.00426249157173 0.00109210772502 0.37550744249 0.378052644976 0.032775 0.0358377240818 0.004715 0.00539690829864 0.045 Chr2 157769492

N00215 600000 800000 0.8514129 scaffold_3 36889068 37082473 0.0036622830682 0.0010344722766 0.00366246571426 0.00103502996937 0.399768294408 0.403055664414 0.056045 0.0350508001344 0.00841 0.00522737261188 0.036 Chr2 157769492

N00003 0 200000 0 scaffold_3 16625113 16826127 0.000805524165347 0.00137326052771 0.000820555956565 0.0014253659501 0.397601840448 0.391105897018 0.146145 0.0864168664869 0.03556 0.0404897171341 0.0435 Chr2 157769492

N00003 200000 400000 1.057423 scaffold_3 16826127 17019121 0.00206790380081 0.00112126715365 0.0020899063504 0.00114661025404 0.418580584858 0.415308839482 0.06598 0.026415328974 0.02141 0.0248712395204 0.2065 Chr2 157769492

N00003 400000 600000 1.23571 scaffold_3 17019121 17212732 0.00327050197689 0.00123544876298 0.00332468480371 0.00125061524265 0.445319071544 0.444074431104 0.118185 0.0493308747953 0.025955 0.0250863845546 0.0733333333333 Chr2 157769492

N00003 600000 800000 1.23571 scaffold_3 17212732 17406960 0.00343342043095 0.00123528202113 0.00347060503938 0.00125912930796 0.379972572836 0.379445827591 0.078975 0.0485511872645 0.02124 0.0219020944457 0.0655 Chr2 157769492

N00003 800000 1000000 1.23571 scaffold_3 17406960 17597639 0.0032473299889 0.000962645214191 0.00326187902614 0.000969827340426 0.426865855089 0.429804566553 0.0889 0.0403033370219 0.005095 0.0102423444637 0.088 Chr2 157769492

N00003 1000000 1200000 1.23571 scaffold_3 17597639 17794921 0.00385241430747 0.000883984316295 0.0038822281787 0.000905853807035 0.407330003929 0.408237546854 0.06605 0.0472521568111 0.013365 0.0338348151377 0.117 Chr2 157769492

N00003 1200000 1400000 1.23571 scaffold_3 17794921 17994121 0.00364898498107 0.000911866464064 0.00371650259464 0.000936221743695 0.396492529875 0.39702526691 0.06989 0.0432178714859 0.02952 0.0318524096386 0.087 Chr2 157769492

N00003 1400000 1600000 1.23571 scaffold_3 17994121 18184075 0.00377156260875 0.00119908596506 0.0038462190037 0.00122739790865 0.382890612488 0.381257025722 0.063385 0.046737631216 0.023165 0.0249007654485 0.051 Chr2 157769492

N00003 1600000 1800000 1.408884 scaffold_3 18184075 18383024 0.00384985139533 0.00105383931379 0.00385666549493 0.0010564275419 0.38253539135 0.382640997831 0.075805 0.0566828684738 0.002195 0.00224178055683 NA Chr2 157769492

N00003 1800000 2000000 3.55625 scaffold_3 18383024 18577293 0.00398688218541 0.00131211373468 0.00402976344754 0.00132960915791 0.402197644276 0.403451149566 0.06604 0.0482732705681 0.01598 0.0165234803288 0.065 Chr2 157769492

N00003 2000000 2200000 3.55625 scaffold_3 18577293 18774723 0.0044201740671 0.00122955442894 0.00442536916448 0.00123100015021 0.387261605657 0.388938943134 0.039705 0.0433166185483 0.001065 0.00111431899914 0.02 Chr2 157769492

N00003 2200000 2400000 3.55625 scaffold_3 18774723 18966308 0.00401465441076 0.00109090776506 0.00402842056455 0.00109308097215 0.38948906232 0.389305108341 0.0557 0.0462301328392 0.003255 0.00339796956964 0.02 Chr2 157769492

N00003 2400000 2600000 3.55625 scaffold_3 18966308 19153797 0.00387677911843 0.000990857629148 0.00388055979023 0.000991742220702 0.393298701872 0.394047109208 0.064255 0.031260500616 0.001605 0.00171210044323 0.02 Chr2 157769492

N00003 2600000 2800000 3.55625 scaffold_3 19153797 19349822 0.00375663311418 0.00101780607819 0.00375663311418 0.00101865160215 0.39226859779 0.392960682497 0.06657 0.0553296773371 0.000375 0.00102537941589 0.02 Chr2 157769492

N00003 2800000 3000000 3.55625 scaffold_3 19349822 19541866 0.00383397518973 0.00104386292212 0.00385321695094 0.00104960610692 0.385760182752 0.382847175545 0.09373 0.0659119785049 0.005815 0.00607673241549 0.083 Chr2 157769492

N00003 3000000 3200000 1.334321 scaffold_3 19541866 19739887 0.00394822784918 0.00093371381275 0.00395397818861 0.000935076982763 0.387654805268 0.38358859226 0.098755 0.0945859277551 0.0015 0.00125744239247 NA Chr2 157769492

N00003 3200000 3400000 0.2480447 scaffold_3 19739887 19938299 0.00431710495155 0.00106642125666 0.00439223102927 0.00109124963735 0.398386415195 0.401351223923 0.07496 0.0426990303006 0.02333 0.0230681611999 0.0625 Chr2 157769492

N00003 3400000 3600000 0.2480447 scaffold_3 19938300 20133698 0.00464111161515 0.00128404572543 0.00464324412451 0.0012887138441 0.37325676316 0.371488392554 0.100965 0.0864696670386 0.00039 0.0170165508347 0.032 Chr2 157769492

N00003 3600000 3800000 0.2480447 scaffold_3 20133698 20337227 0.00455970973358 0.00107449814916 0.00456945646222 0.00107646010828 0.399525131621 0.401303538175 0.056345 0.0773403298793 0.003885 0.00383237769556 0.053 Chr2 157769492

N00003 3800000 4000000 5.660955 scaffold_3 20337227 20530213 0.00505354885662 0.00127685273645 0.00510948051615 0.00129764753227 0.388655971922 0.387082870547 0.070785 0.0578694827604 0.01678 0.0174883152146 0.119 Chr2 157769492

N00003 4000000 4200000 7.783738 scaffold_3 20530213 20724102 0.00347812372998 0.000990682565895 0.0035845678148 0.00101951907331 0.401467185324 0.40145559614 0.03995 0.030192532841 0.032005 0.0320905260226 NA Chr2 157769492

N00003 4200000 4400000 1.824038 scaffold_3 20724102 20917159 0.00342966276665 0.00100262443183 0.00342966276665 0.00100365453768 0.397087603716 0.397913179273 0.06505 0.0325914108269 0 0.000922007490016 NA Chr2 157769492

N00003 4400000 4600000 4.464546 scaffold_3 20917159 21109562 0.0038342485959 0.000932727481811 0.00383440623682 0.000932769960516 0.391329346826 0.394787480598 0.059005 0.0378996169498 3.5e-05 3.63819690961e-05 0.02 Chr2 157769492

N00003 4600000 4800000 3.889838 scaffold_3 21109562 21299132 0.00373739747241 0.00105526415484 0.00380335586143 0.00107432018172 0.412964536366 0.416787647569 0.088025 0.0524977580841 0.023325 0.0256264176821 0.05875 Chr2 157769492

N00003 4800000 5000000 3.330935 scaffold_3 21299132 21494330 0.00394575572302 0.00102556170857 0.00395312585028 0.0010277198388 0.396750005379 0.396355788959 0.05738 0.0352001557393 0.00188 0.00194161825429 NA Chr2 157769492

N00003 5000000 5200000 3.374676 scaffold_3 21494330 21693224 0.00448556043561 0.00119131290874 0.00448929001026 0.00119300562166 0.38424202311 0.387212146135 0.074725 0.0769505364667 0.00113 0.00114131145233 NA Chr2 157769492

N00003 5200000 5400000 5.528889 scaffold_3 21693224 21887194 0.00503832810963 0.00124861962427 0.00504138600534 0.00125059080402 0.372858361961 0.370922909881 0.069785 0.0530442851987 0.00874 0.00153632004949 NA Chr2 157769492

N00003 5400000 5600000 2.656002 scaffold_3 21887194 22079708 0.00487835207145 0.00128286460126 0.00488042163027 0.00128334320657 0.363709346912 0.362251854715 0.06682 0.0722596798155 0.000825 0.000857080524014 0.095 Chr2 157769492

N00003 5600000 5800000 2.30291 scaffold_3 22079936 22271619 0.00469483461051 0.00122089167825 0.00471406813831 0.00122722558466 0.364198851481 0.36565063447 0.073165 0.068028985356 0.005095 0.00533693650454 0.095 Chr2 157769492

N00003 5800000 6000000 2.30291 scaffold_3 22271619 22465472 0.00518455973854 0.00133832165892 0.00518455973854 0.00133832165892 0.373576060886 0.371337753448 0.062 0.0558258061521 0 0 NA Chr2 157769492

N00003 6000000 6200000 2.308764 scaffold_3 22465472 22652150 0.00425286327791 0.00108740874225 0.00432287623557 0.00111325945326 0.382263362912 0.384505418753 0.09069 0.0579554098501 0.02441 0.0339729373574 0.0675 Chr2 157769492

N00003 6200000 6400000 3.007972 scaffold_3 22652150 22846260 0.00513403928009 0.00158941566107 0.00521590269766 0.00161967678452 0.365342182138 0.364053668529 0.04527 0.0577095461336 0.022505 0.0231930348771 0.0825 Chr2 157769492

N00003 6600000 6800000 3.007972 scaffold_3 23035595 23223679 0.00318837911581 0.000907564161268 0.00320010139114 0.000910519868714 0.379033420387 0.381733008094 0.09453 0.053438889007 0.003195 0.00339741817486 0.007 Chr2 157769492

N00003 6800000 7000000 3.007972 scaffold_3 23223679 23418921 0.00332059047992 0.000891026530087 0.00333884186953 0.000897957147457 0.394842838916 0.395029712534 0.03938 0.0199444791592 0.0081 0.00880445805718 0.007 Chr2 157769492

N00003 7000000 7200000 3.007972 scaffold_3 23418921 23618254 0.00367204618645 0.000911572161846 0.00367204618645 0.000911572161846 0.364577336935 0.364386714896 0.0877 0.0903563383885 0 0 NA Chr2 157769492

N00003 7400000 7600000 3.334523 scaffold_3 24265015 24453771 0.00427416904174 0.00127427454491 0.00432620394552 0.00129275666576 0.388179271709 0.385815976221 0.07303 0.0339432918689 0.013535 0.0145637754561 0.035 Chr2 157769492

N00003 7600000 7800000 1.956187 scaffold_3 24453771 24646286 0.00452887922834 0.00138044667274 0.00465274535055 0.00143154944038 0.403143436525 0.402970163662 0.06646 0.0403708801912 0.041425 0.0428070539958 0.0666666666667 Chr2 157769492

N00003 7800000 8000000 0 scaffold_3 24646286 24841619 0.00422905051016 0.00108984630021 0.00426982007073 0.00109421807559 0.401388404153 0.401476455217 0.05724 0.0366297553409 0.010615 0.0078378973343 0.118 Chr2 157769492

N00003 8000000 8200000 3.966392 scaffold_3 24841619 25031507 0.00460150751699 0.0011310925825 0.00460150751699 0.0011310925825 0.387108687498 0.393707420495 0.10365 0.0589558055275 0.00651 0 0.037 Chr2 157769492

N00003 8200000 8400000 5.52673 scaffold_3 25031507 25229699 0.00365123020603 0.000924221805221 0.00366214259625 0.000925652658727 0.407011768926 0.408030830833 0.063115 0.0372265278114 0.006165 0.00647352062646 0.084 Chr2 157769492

N00003 8400000 8600000 5.587209 scaffold_3 25229699 25416938 0.0043043971973 0.00105800871062 0.0043078893496 0.00105789238391 0.394035349028 0.397470065769 0.094965 0.0413856087674 0.00745 0.00155416339545 0.084 Chr2 157769492

N00003 8600000 8800000 1.111882 scaffold_3 25416938 25609152 0.00396630270823 0.000949732511727 0.00401822126917 0.000962414596466 0.38135737277 0.380317453515 0.0521 0.0342014629528 0.0145 0.0156856420448 0.052 Chr2 157769492

N00003 8800000 9000000 4.558027 scaffold_3 25609152 25803645 0.00411037137598 0.000919323612507 0.00412863697334 0.00092450979058 0.383646272312 0.381534338575 0.06542 0.0581306268092 0.005375 0.00596936650676 0.052 Chr2 157769492

N00003 9200000 9400000 4.405086 scaffold_3 25993773 26189062 0.00406932952111 0.000879961516603 0.00408377190301 0.000883429934636 0.380348421076 0.381502670546 0.06969 0.0459831326905 0.00913 0.0049465151647 0.0545 Chr2 157769492

N00003 9400000 9600000 3.626168 scaffold_3 26189062 26380573 0.00358247751886 0.000842114588312 0.00359632202747 0.000845509949875 0.411068879713 0.41160189818 0.059505 0.0370004856118 0.003465 0.00364992089227 0.081 Chr2 157769492

N00003 9800000 10000000 7.180898 scaffold_3 26569297 26756411 0.00405098531091 0.00109883024652 0.00405098531091 0.00109883024652 0.418279431441 0.418939714122 0.07719 0.0351817608517 0 0 NA Chr2 157769492

N00003 10000000 10200000 6.2029 scaffold_3 26756411 26951456 0.00421072961739 0.00108718681049 0.0042190201369 0.00109672760834 0.402513958797 0.401708665992 0.066495 0.0455125740214 0.00412 0.0122279473968 0.117 Chr2 157769492

N00003 10200000 10400000 0.6427907 scaffold_3 26951456 27144825 0.00426794919167 0.00107329445991 0.00436125875297 0.00110351450623 0.385824175199 0.385900633456 0.06479 0.0490668100885 0.029065 0.0310753016254 0.0335 Chr2 157769492

N00003 10400000 10600000 0.6427907 scaffold_3 27145834 27342990 0.00459804753687 0.00116740222972 0.00460135108952 0.00116809018891 0.406971954084 0.409187532165 0.099215 0.0707054312321 0.002555 0.00149120493416 NA Chr2 157769492

N00003 10600000 10800000 0.6427907 scaffold_3 27342990 27527146 0.0044703413824 0.00116787373737 0.00449556936063 0.00117462754402 0.393248110324 0.397187392155 0.120235 0.0374247920242 0.010465 0.00970915962553 0.0823333333333 Chr2 157769492

N00003 10800000 11000000 0.6427907 scaffold_3 27527146 27726215 0.00392344271708 0.00100979655736 0.00404973094703 0.00104265596872 0.397231468089 0.399827263894 0.07878 0.0749639572209 0.04074 0.0447734202714 0.0705 Chr2 157769492

N00003 11200000 11400000 2.462966 scaffold_3 27917025 28128192 0.00449143117121 0.00119035887467 0.00454315267627 0.0012089890097 0.411050806573 0.411390011454 0.058175 0.0503866607945 0.016565 0.0155374656078 0.0845 Chr2 157769492

N00003 11400000 11600000 2.988016 scaffold_3 28128192 28316897 0.0050230162615 0.00122528190052 0.00506571329082 0.00126131197233 0.376191237703 0.376642628205 0.11075 0.0640576561299 0.01165 0.0332847566307 0.052 Chr2 157769492

N00003 11600000 11800000 2.988016 scaffold_3 28316897 28514349 0.00450230784824 0.00108326456965 0.00450230784824 0.00108326456965 0.376482490529 0.379377748819 0.07908 0.0679405627697 0 0 NA Chr2 157769492

N00003 11800000 12000000 2.966481 scaffold_3 28514349 28711325 0.00469832246848 0.00125492693422 0.00481328234844 0.00128256197677 0.393660549653 0.393619424381 0.10897 0.0859749411096 0.027225 0.0269423686134 0.091 Chr2 157769492

N00003 12000000 12200000 2.122324 scaffold_3 28711325 28906554 0.00484547766166 0.00123296297242 0.00491463476085 0.00125028132006 0.374224787844 0.375424707241 0.07374 0.0557550363932 0.01679 0.0164114962429 0.091 Chr2 157769492

N00003 12200000 12400000 2.122324 scaffold_3 28906554 29095764 0.00451750834427 0.0012267728849 0.00453280244133 0.00123211463239 0.381914761642 0.383373090949 0.077375 0.0503937424026 0.00731 0.00725648750066 0.0615 Chr2 157769492

N00003 12400000 12600000 2.122324 scaffold_3 29095764 29283707 0.00462808531073 0.00122471088252 0.00463949433653 0.00122797902851 0.384878088445 0.389683834121 0.07378 0.0528670926824 0.006875 0.00283064546166 0.0796666666667 Chr2 157769492

N00003 12600000 12800000 0.933661 scaffold_3 29283707 29478307 0.0041061631828 0.00106414280268 0.00413582265444 0.00107531455126 0.378389263907 0.377910666981 0.033375 0.0273535457348 0.013435 0.013170606372 0.058 Chr2 157769492

N00003 12800000 13000000 1.087329 scaffold_3 29478307 29674738 0.00438999849745 0.00128274783071 0.00438999849745 0.00128356838478 0.371705392275 0.373666849918 0.087185 0.0648675616374 0 0.000539629691851 NA Chr2 157769492

N00003 13000000 13200000 1.087329 scaffold_3 29674738 29866739 0.00441584487451 0.00108307872348 0.00447935910904 0.00109620947737 0.372123647675 0.371919213376 0.09116 0.0628746725277 0.01435 0.0140988849016 0.033 Chr2 157769492

N00003 13200000 13400000 1.087329 scaffold_3 29866774 30064806 0.00450083667245 0.00115049596218 0.00454397004347 0.00115973515855 0.370182801666 0.369781928136 0.052895 0.0395390643936 0.01128 0.0114375454472 0.048 Chr2 157769492

N00003 13400000 13600000 1.087329 scaffold_3 30064806 30251674 0.0043414514558 0.00110676865165 0.00439200475379 0.00111909434151 0.394412813035 0.398046107107 0.06424 0.059405569707 0.012445 0.0123937752852 0.082 Chr2 157769492

N00003 13600000 13800000 1.087329 scaffold_3 30251674 30445258 0.00407608896606 0.001115682418 0.00413875095982 0.00113947082641 0.368159061814 0.364437291101 0.088255 0.0603717249359 0.02015 0.0239895859162 0.0643333333333 Chr2 157769492

N00003 13800000 14000000 1.087329 scaffold_3 30445258 30644653 0.00366412701569 0.000974136992113 0.0036642405228 0.00097301871826 0.381987934974 0.37953632352 0.083935 0.0829960630908 0.000505 0.000526592943655 0.072 Chr2 157769492

N00003 14000000 14200000 1.087329 scaffold_3 30644653 30843052 0.00382995073413 0.00104898985057 0.00384081919792 0.00104867007093 0.369440112461 0.366977547866 0.061975 0.0590627976956 0.006235 0.00570063357174 0.0965 Chr2 157769492

N00003 14200000 14400000 1.087329 scaffold_3 30843052 31039007 0.00434935549748 0.00118238919042 0.00435754066184 0.00118394745041 0.358814113107 0.354731923318 0.056765 0.0637391237784 0.003655 0.00447041412569 0.159 Chr2 157769492

N00003 14400000 14600000 1.087329 scaffold_3 31039007 31228130 0.00449074569644 0.00114275659477 0.00452647000984 0.00115410316061 0.349220079001 0.347466037026 0.112445 0.0680403758401 0.009015 0.00953347821259 0.022 Chr2 157769492

N00003 14600000 14800000 1.087329 scaffold_3 31228130 31422322 0.00392558767697 0.0010832689852 0.0039361790743 0.00108724352155 0.354394335847 0.354072452293 0.055915 0.0599097800115 0.00319 0.00330600642663 0.022 Chr2 157769492

N00003 14800000 15000000 1.087329 scaffold_3 31422322 31616040 0.00342514342499 0.000993714115802 0.00344575650105 0.00100014504747 0.368584428054 0.37016428336 0.065195 0.0425154089966 0.00715 0.0077174036486 0.053 Chr2 157769492

N00003 15000000 15200000 1.087329 scaffold_3 31616040 31807395 0.00500167188441 0.00132906651821 0.00500167188441 0.00132906651821 0.360634612949 0.362644646359 0.06699 0.0510255807269 0 0 NA Chr2 157769492

N00003 15200000 15400000 1.087329 scaffold_3 31807395 32013862 0.00514311936581 0.00141210984703 0.00514311936581 0.00141210984703 0.382062464756 0.386270665536 0.05619 0.0638068068989 0 0 NA Chr2 157769492

N00003 15400000 15600000 1.087329 scaffold_3 32013862 32211649 0.00476654083262 0.00116835313652 0.00487038255608 0.00121300021102 0.38260518732 0.380343016122 0.076115 0.0663693771583 0.03999 0.0443406290606 0.08 Chr2 157769492

N00003 15600000 15800000 4.441751 scaffold_3 32211649 32412255 0.00453809348285 0.0013426419552 0.00454614310336 0.00134907872156 0.365450427094 0.370283234231 0.064155 0.0731683000508 0.00412 0.00488021295475 NA Chr2 157769492

N00003 15800000 16000000 5.437257 scaffold_3 32412255 32615179 0.00498607409653 0.00150669358189 0.00504451285706 0.00153065881893 0.360905157419 0.363139743264 0.052105 0.0649454968363 0.02437 0.0239547811003 0.19 Chr2 157769492

N00003 16000000 16200000 5.437257 scaffold_3 32615179 32809337 0.00452824373865 0.00138763855467 0.00458301466655 0.00140504646796 0.355292527361 0.353834275441 0.082375 0.0758196932395 0.01426 0.015162908559 0.061 Chr2 157769492

N00003 16200000 16400000 5.437257 scaffold_3 32809337 33010693 0.0050367973643 0.00134910909305 0.00504165655282 0.00135086199981 0.360789384909 0.363169664988 0.09098 0.108414946662 0.00124 0.0169252468265 NA Chr2 157769492

N00003 16600000 16800000 0.7926375 scaffold_3 33219696 33408295 0.00509327021826 0.0011175483853 0.00509327021826 0.0011175483853 0.348183336212 0.348704295944 0.098335 0.0791573656276 0 0 NA Chr2 157769492

N00003 16800000 17000000 0.7926375 scaffold_3 33408301 33606625 0.00473362386333 0.00123675333818 0.00473362386333 0.00123675333818 0.358966687762 0.358710515907 0.115125 0.117903027369 0 0 NA Chr2 157769492

N00003 17000000 17200000 0.7926375 scaffold_3 33606625 33799023 0.00437721302678 0.00109736788558 0.00438952927026 0.00110071342001 0.364636030066 0.362310743005 0.082505 0.0903959500619 0.004065 0.00396054013035 0.071 Chr2 157769492

N00003 17200000 17400000 0.7926375 scaffold_3 33799023 33991596 0.00383654556328 0.00110953185045 0.00386557841746 0.00111661074576 0.385425197925 0.389032110691 0.070575 0.0503964730258 0.01009 0.0115384815109 0.05 Chr2 157769492

N00003 17400000 17600000 0.7926375 scaffold_3 33991596 34186349 0.00362254043523 0.000986873564855 0.00367815572093 0.00101098694298 0.37537316783 0.373403129699 0.07777 0.0466436974013 0.024335 0.024774971374 0.0856666666667 Chr2 157769492

N00003 17600000 17800000 0.7926375 scaffold_3 34186349 34391292 0.00467447076557 0.00122212039395 0.00479382280581 0.0012500626264 0.388795201177 0.386191016256 0.08822 0.0896346789107 0.028725 0.0265830011271 0.08875 Chr2 157769492

N00003 18000000 18200000 0.7926375 scaffold_3 34599976 34790036 0.00351511331449 0.000904954612725 0.00351671322637 0.000904954612725 0.412192617413 0.40904034194 0.05987 0.0240345154162 0.000735 0 0.085 Chr2 157769492

N00003 18200000 18400000 0.7926375 scaffold_3 34790036 34986077 0.00360629928259 0.00102890785452 0.0036446329505 0.00104109676773 0.394120177188 0.38850627487 0.070345 0.0613596135502 0.014835 0.0159303411021 0.0475 Chr2 157769492

N00003 18400000 18600000 0.9713187 scaffold_3 34986077 35176561 0.00411361887497 0.0010684735076 0.00416133267148 0.00108226232362 0.408910018579 0.409285558226 0.068195 0.0365227525671 0.016995 0.0176077780811 0.0716666666667 Chr2 157769492

N00003 18600000 18800000 2.222409 scaffold_3 35176561 35374078 0.00443421776694 0.00111261510956 0.00452512874756 0.00113500434259 0.387549762317 0.387870813797 0.074905 0.0636400917389 0.025265 0.0258053737147 0.067 Chr2 157769492

N00003 18800000 19000000 2.222409 scaffold_3 35374078 35580562 0.00462967624803 0.00121711933923 0.00479368723203 0.00126400587123 0.380704049301 0.385980291219 0.100425 0.0883264562872 0.038585 0.0497956258112 0.0386666666667 Chr2 157769492

N00003 19000000 19200000 2.222409 scaffold_3 35580562 35780407 0.00486196848385 0.00134348943431 0.00488366532824 0.00135300172774 0.387010074602 0.387957066894 0.077895 0.0901648777803 0.00827 0.0092621781881 0.081 Chr2 157769492

N00003 19200000 19400000 2.222409 scaffold_3 35780407 35978470 0.00470669988859 0.00112443961486 0.00471772923919 0.00112724873422 0.388027207937 0.38711079243 0.074675 0.0532911245412 0.00363 0.0031353660199 0.034 Chr2 157769492

N00003 19400000 19600000 2.222409 scaffold_3 35978470 36176878 0.00483397171277 0.000957058475132 0.00491645948131 0.000975753913661 0.385042640381 0.383133858447 0.098945 0.0676434417967 0.02224 0.0214557880731 0.0625 Chr2 157769492

N00003 19600000 19800000 1.935933 scaffold_3 36177267 36387179 0.00401908335365 0.000618698312927 0.0040484221671 0.000638399540453 0.380955149721 0.376770303623 0.084185 0.0488680971074 0.010225 0.0324278745379 0.049 Chr2 157769492

N00003 19800000 20000000 0.8514129 scaffold_3 36387179 36584493 0.00360409197655 0.00112692843964 0.00371655966899 0.00117522167331 0.374893668039 0.371261282687 0.065275 0.0489727034068 0.05165 0.0519679292904 0.0514285714286 Chr2 157769492

N00113 0 200000 2.48862 scaffold_3 14104110 14299768 0.00375051409056 0.00109900891303 0.0037634863003 0.00110148650081 0.430727394856 0.425565281401 0.05924 0.0463410645105 0.00335 0.00869885207863 0.024 Chr2 157769492

N00113 200000 400000 2.48862 scaffold_3 14299768 14503762 0.00344976534286 0.00112505867192 0.00348257669581 0.00114052183193 0.395442039473 0.388762493108 0.050245 0.0516828926341 0.01536 0.0154465327412 0.033 Chr2 157769492

N00113 400000 600000 2.48862 scaffold_3 14503766 14698209 0.00394579531039 0.00135277484543 0.00398614163224 0.00137132971658 0.366666111213 0.365430731345 0.055345 0.0462243433808 0.014725 0.0168172677854 NA Chr2 157769492

N00113 1000000 1200000 2.48862 scaffold_3 15044292 15227460 0.00360276609089 0.0011867580326 0.00360764174347 0.00118929497093 0.403897626087 0.399706015378 0.112555 0.0539832285115 0.0039 0.00313919461915 NA Chr2 157769492

N00113 1200000 1400000 2.48862 scaffold_3 15227460 15425532 0.00336475705487 0.00106905306731 0.00348485777593 0.00115104006776 0.424506446117 0.417818432577 0.071485 0.0505927137607 0.08058 0.087165273234 0.0877142857143 Chr2 157769492

N00113 1400000 1600000 2.48862 scaffold_3 15425532 15620501 0.00335738824187 0.00102785633735 0.0034609831673 0.00107303398227 0.419667713879 0.414794128346 0.089365 0.0671645235909 0.058995 0.0602044427576 0.0985 Chr2 157769492

N00113 1600000 1800000 2.48862 scaffold_3 15620501 15817222 0.00317318255622 0.00118355072093 0.00317318255622 0.00118402071121 0.3901119206 0.388178260616 0.05833 0.0342261375247 0 0.00120475190752 NA Chr2 157769492

N00113 1800000 2000000 2.48862 scaffold_3 15817248 16003895 0.00280627622523 0.000926476273299 0.00297129160125 0.000996299606392 0.375727548818 0.373792193029 0.099095 0.0388755243856 0.0841 0.0891201037252 0.06 Chr2 157769492

N00113 2000000 2200000 2.48862 scaffold_3 16003895 16200040 0.00263052214665 0.000976711234823 0.00264408291028 0.000980779322525 0.391194516641 0.388197767145 0.09876 0.0676744245329 0.00774 0.00790741543246 0.064 Chr2 157769492

N00113 2200000 2400000 1.852064 scaffold_3 16200620 16394932 0.00138767841546 0.00116200793625 0.00139569426132 0.00117393591431 0.396452616783 0.391194856918 0.085395 0.0438161307588 0.012765 0.0138128370867 0.124 Chr2 157769492

N00113 2400000 2600000 0.9703825 scaffold_3 16394932 16586796 0.000407630813896 0.00119415262298 0.000412526361264 0.00123494631007 0.374259039715 0.37025198102 0.0586 0.030876037193 0.04501 0.0440728849602 0.105 Chr2 157769492

N00096 0 200000 2.48862 scaffold_3 13892299 14080170 0.00357427779609 0.00107231233465 0.0036597654324 0.00110255375992 0.428852529396 0.424528085375 0.09379 0.0477827871252 0.034755 0.0408950822639 0.106 Chr2 157769492

N00096 200000 400000 2.48862 scaffold_3 13701119 13892299 0.00392667262821 0.00115608329038 0.0039655813496 0.00117558148584 0.400216804591 0.395105124836 0.06491 0.0304477455801 0.02302 0.0201067057224 0.044 Chr2 157769492

N00096 400000 600000 2.48862 scaffold_3 13513131 13701119 0.00385561575774 0.00111297840847 0.00401535707437 0.00117569785645 0.398241097179 0.390768478546 0.095035 0.0551790539822 0.06725 0.0740685575675 0.0585 Chr2 157769492

N00096 600000 800000 2.48862 scaffold_3 13323284 13513131 0.00416886710924 0.00106400347985 0.00420885410008 0.00107579698676 0.352162797125 0.34781182186 0.11327 0.0662744209811 0.014025 0.0148066601 0.05 Chr2 157769492

N00096 800000 1000000 2.48862 scaffold_3 13125639 13323284 0.00385334633632 0.00101035084274 0.00386158215231 0.0010132129385 0.383562744363 0.377471215578 0.05535 0.0395911862177 0.00225 0.00543398517544 0.083 Chr2 157769492

N00096 1000000 1200000 2.48862 scaffold_3 12944231 13125639 0.00412564795408 0.00102471988506 0.0041556143905 0.00103356504617 0.396748716048 0.390327894257 0.119985 0.0682935702946 0.00879 0.00971291233022 0.067 Chr2 157769492

N00096 1400000 1600000 2.544858 scaffold_3 12588889 12771734 0.00375340677412 0.000919696871441 0.00375771225966 0.00092068534058 0.421533143083 0.415865177572 0.125785 0.0439279170882 0.00263 0.000880527222511 0.042 Chr2 157769492

N00096 1600000 1800000 2.565826 scaffold_3 12403636 12588857 0.00422837835577 0.00111290822432 0.00426896975946 0.00112802088936 0.398693421529 0.392886781942 0.102545 0.045702161202 0.01193 0.0132976282387 0.0455 Chr2 157769492

N00096 1800000 2000000 2.583882 scaffold_3 12220475 12403636 0.00417535612143 0.00103818715238 0.00424641346822 0.00106346046856 0.420111002285 0.41175886442 0.10952 0.0376280976846 0.034185 0.0376663154274 0.099 Chr2 157769492

N00096 2000000 2200000 2.583882 scaffold_3 12037315 12220475 0.00372032971013 0.00116241685834 0.00375159536417 0.00117229367628 0.415978763487 0.406113208421 0.125 0.0297335662808 0.011435 0.013889495523 0.092 Chr2 157769492

N00096 2200000 2400000 3.685098 scaffold_3 11835314 12037315 0.00412288657969 0.00104162371943 0.00415227380414 0.00104819882296 0.391100012325 0.382026399454 0.091585 0.0675491705487 0.00718 0.00711382616918 0.057 Chr2 157769492

N00238 0 200000 4.797327 scaffold_3 10038085 10227207 0.00423260806541 0.00125000846082 0.00436502377543 0.00129293748507 0.3711479693 0.36572632762 0.077245 0.0471177335265 0.031555 0.0364526601876 0.045 Chr2 157769492

N00238 400000 600000 4.797327 scaffold_3 10397450 10582174 0.00415194546465 0.00111961809772 0.00415194546465 0.00111961809772 0.399830938292 0.390730762705 0.12492 0.0621088759447 0 0 NA Chr2 157769492

N00175 0 200000 3.584012 scaffold_3 9753893 9953645 0.00418431328789 0.00124251639249 0.00428834310284 0.00128069633448 0.392460869812 0.385973713839 0.11192 0.063874204013 0.029685 0.0395740718491 0.0496666666667 Chr2 157769492

N00175 200000 400000 1.410156 scaffold_3 9563845 9753893 0.00450330003558 0.0012599796959 0.00452957411228 0.00126790703918 0.389936928878 0.382747522562 0.08782 0.0604321013639 0.008005 0.00843997305944 NA Chr2 157769492

N00175 600000 800000 1.410156 scaffold_3 9201142 9382442 0.00509902779197 0.0014206252944 0.00511624545373 0.00142763325319 0.355511240029 0.349551011274 0.101195 0.0511913954771 0.005115 0.00564258135687 NA Chr2 157769492

N00175 1200000 1400000 3.607335 scaffold_3 8622634 8815394 0.00473489219298 0.00131597216663 0.00473489219298 0.00131684369716 0.361002449245 0.357721021162 0.12529 0.0980130732517 0 0.000503216434945 NA Chr2 157769492

N00081 400000 600000 3.607335 scaffold_3 8026292 8220551 0.00439462776296 0.00109886051742 0.00439891024476 0.00110129921086 0.38231246681 0.381668448962 0.089555 0.0836512079234 0.001195 0.00199218569024 0.063 Chr2 157769492

N00081 600000 800000 1.997931 scaffold_3 7824143 8026292 0.00436506344522 0.00123604125376 0.00437495805799 0.00123596853296 0.397530822363 0.396317128774 0.091285 0.115578113174 0.002895 0.00289390499087 0.0855 Chr2 157769492

N00081 800000 1000000 1.263203 scaffold_3 7625521 7824143 0.00433495773794 0.00109334238752 0.00434262156578 0.00109745184532 0.416502308242 0.417673095163 0.04874 0.029815428301 0.00236 0.00328261723273 NA Chr2 157769492

N00081 1000000 1200000 1.263203 scaffold_3 7422713 7625506 0.00425284186303 0.00111069189666 0.00432167960598 0.00113224766009 0.415617002615 0.414295142851 0.08288 0.0754168043276 0.023155 0.0229396478182 0.072 Chr2 157769492

N00081 1400000 1600000 1.263203 scaffold_3 7066370 7265978 0.00427891805986 0.00099710562376 0.00434479107509 0.00101816622576 0.399081611355 0.401100102793 0.0615 0.0783134944491 0.024015 0.0247835758086 0.058 Chr2 157769492

N00081 1600000 1800000 1.263203 scaffold_3 6871930 7066370 0.0047569799 0.00102285877156 0.00479727931678 0.00103275515882 0.407778624489 0.409033819473 0.11436 0.0938438592882 0.01399 0.0149506274429 0.105 Chr2 157769492

N00081 1800000 2000000 1.263203 scaffold_3 6674274 6871930 0.00405450012922 0.000971862236767 0.00405382259291 0.000971862236767 0.409370149675 0.413481347067 0.062965 0.0492522362084 0.000185 0 0.163 Chr2 157769492

N00081 2000000 2200000 1.263203 scaffold_3 6481920 6674274 0.0035241019136 0.000921344932149 0.00354462916935 0.000927703811466 0.400967493519 0.403115438407 0.05929 0.0224377969785 0.00757 0.00788650093058 NA Chr2 157769492

N00081 2200000 2400000 1.263203 scaffold_3 6284257 6481920 0.0031401492998 0.000801282726428 0.00316730590859 0.000813214306101 0.404163940016 0.403757257093 0.06503 0.0566368010199 0.022395 0.0226951933341 0.042 Chr2 157769492

N00081 2400000 2600000 1.263203 scaffold_3 6094287 6284257 0.00393447593042 0.00100157219104 0.00393447593042 0.00100157219104 0.385731764387 0.386429904036 0.104805 0.0650576406801 0 0 NA Chr2 157769492

N00081 2600000 2800000 1.263203 scaffold_3 5888063 6094287 0.0038911600463 0.000913988620642 0.00389612090084 0.000914999289469 0.368671491556 0.367718789964 0.11496 0.116727442005 0.001125 0.00110559391729 NA Chr2 157769492

N00081 2800000 3000000 1.263203 scaffold_3 5693175 5888063 0.00374040194903 0.000940497474241 0.00379724059701 0.000954978236363 0.360413286448 0.364290958212 0.116385 0.121495423012 0.016545 0.0168917532121 0.055 Chr2 157769492

N00081 3200000 3400000 1.390582 scaffold_3 5347693 5538899 0.00396356176811 0.00116648095585 0.00398552948838 0.00117147307958 0.374094389213 0.376587185529 0.08256 0.0773825089171 0.007495 0.00788678179555 0.036 Chr2 157769492

N00081 3400000 3600000 1.390582 scaffold_3 5137494 5347693 0.00416398133861 0.00101418193736 0.0041764357156 0.001016591538 0.382465050119 0.378554184549 0.1024 0.13584270144 0.00287 0.00264511248864 0.039 Chr2 157769492

N00076 2600000 2800000 1.55013 scaffold_3 1863479 2066308 0.00427447237235 0.00109777834027 0.00432125457698 0.00111426530144 0.373547965562 0.374050199658 0.11005 0.105113174152 0.013385 0.0151704144871 0.0945 Chr2 157769492

N00076 2800000 3000000 1.704431 scaffold_3 1671875 1863479 0.00517036098832 0.00103259543612 0.00527011198828 0.00106033158964 0.376176110398 0.381000420829 0.09202 0.0907079184151 0.03048 0.0309127158097 0.054 Chr2 157769492

N00076 3200000 3400000 1.798246 scaffold_3 1285432 1473211 0.00454127418352 0.00118702361024 0.00460179259564 0.00121043445365 0.416166098454 0.418325642716 0.099745 0.0642510610878 0.02779 0.0334542201205 0.084 Chr2 157769492

N00076 3400000 3600000 1.798246 scaffold_3 1085973 1285026 0.00459289929113 0.00106569378649 0.00464572649246 0.00108074635523 0.397466750404 0.399016398927 0.09022 0.0779591365114 0.013075 0.0131422284517 0.062 Chr2 157769492

N00076 3600000 3800000 1.828722 scaffold_3 895675 1085973 0.00384637169117 0.000930178320716 0.00390997143077 0.000948721077124 0.414552759908 0.420999200322 0.08305 0.0271521508371 0.022755 0.0258121472638 0.073 Chr2 157769492

N00076 3800000 4000000 2.269458 scaffold_3 699666 895675 0.0043080393833 0.00121331460375 0.00436342099674 0.00123447649073 0.365528382426 0.367305683005 0.094965 0.0489263248116 0.02448 0.0249733430608 0.0505 Chr2 157769492

N00009 400000 600000 0.434968 scaffold_33 2950815 3156876 0.00422061693245 0.00124095299177 0.00422061693245 0.00124095299177 0.353295321338 0.348187811268 0.13154 0.147174865695 0 0 NA Chr2 157769492

N00009 600000 800000 0.5854939 scaffold_33 2762088 2950815 0.00420422758336 0.00122149309903 0.00423908523257 0.00123044358096 0.350757661935 0.348306171789 0.13294 0.102423076719 0.009425 0.0125578216153 0.071 Chr2 157769492

N00009 2200000 2400000 1.00583 scaffold_33 1180583 1377011 0.00437483135071 0.00127633796992 0.00437483135071 0.00127633796992 0.360367067786 0.355547578718 0.108505 0.109969047183 0 0 NA Chr2 157769492

N00009 2400000 2600000 1.00583 scaffold_33 979066 1180583 0.00392457221049 0.00117231604195 0.00392457221049 0.00117231604195 0.380347882159 0.378447307919 0.06483 0.0862706372167 0 0 NA Chr2 157769492

N00009 2600000 2800000 1.00583 scaffold_33 784369 979066 0.0036629882954 0.000987598002427 0.00368844721242 0.0010004177504 0.398832837663 0.398711579828 0.07925 0.0583265278869 0.01185 0.0121881693092 0.098 Chr2 157769492

N00009 2800000 3000000 1.00583 scaffold_33 588346 784369 0.00432795767658 0.00124232374931 0.00439849641777 0.00127255953563 0.376501623617 0.376869753429 0.11216 0.0969325028185 0.031225 0.0315422169847 0.076 Chr2 157769492

N00009 3000000 3200000 1.00583 scaffold_33 390422 588346 0.0043658342216 0.0011995561114 0.0043658342216 0.0011995561114 0.383251486742 0.386627185744 0.07296 0.0832996503708 0 0 NA Chr2 157769492

N00009 3200000 3400000 1.00583 scaffold_33 192728 389798 0.0038509537309 0.0010847516596 0.0038568462762 0.00108410491635 0.400414225494 0.404970631586 0.087125 0.0882732024154 0.00329 0.00333891510631 0.08 Chr2 157769492

N00009 3400000 3600000 1.00583 scaffold_33 88 192728 0.00385232146577 0.000885597653742 0.00385474465741 0.000891154092534 0.42287881835 0.424014277547 0.051565 0.055050872093 0.011725 0.0122041112957 0.08 Chr2 157769492

N00009 3600000 3800000 1.00583 scaffold_106 908266 1105087 0.00444414308412 0.00106777245199 0.00452080545194 0.00109780930897 0.380258369312 0.379836994845 0.059885 0.0603441705916 0.025295 0.0328471047297 0.0565 Chr2 157769492

N00009 3800000 4000000 1.00583 scaffold_106 713577 908266 0.00382483532222 0.00103964702821 0.00387774293814 0.00105582704152 0.42016053527 0.419837064437 0.093 0.0527405246316 0.029105 0.0232370601318 0.0325 Chr2 157769492

N00009 4000000 4200000 1.00583 scaffold_106 514484 713577 0.00342463443162 0.000888352264355 0.00346214679594 0.000894930416099 0.411143984221 0.409515409921 0.05272 0.0436831028715 0.023425 0.023672354126 0.0773333333333 Chr2 157769492

N00009 4200000 4400000 1.00583 scaffold_106 322603 514484 0.00396533522061 0.00108518457758 0.0040433884696 0.00112233200853 0.41002728909 0.409493663706 0.073515 0.0366425023843 0.045145 0.0461536056201 0.04925 Chr2 157769492

N00009 4800000 5000000 1.00583 scaffold_85 824682 1016967 0.00416217721517 0.00102699024312 0.00416683640797 0.00102956106709 0.381981113473 0.383769508053 0.03648 0.0457862027719 0.003345 0.003619627116 0.119 Chr2 157769492

N00009 5000000 5200000 1.00583 scaffold_85 633202 824682 0.00368318746416 0.00101955577239 0.00368665268098 0.0010202689973 0.405097185349 0.41735095179 0.056365 0.0343273448924 0.00172 0.00181742218508 0.119 Chr2 157769492

N00009 5200000 5400000 1.404249 scaffold_85 441546 633202 0.00426399141352 0.00116183626081 0.00426399141352 0.00116249842119 0.423335123523 0.430951675067 0.072425 0.0304973494177 0 0.00336540468339 NA Chr2 157769492

N00009 5400000 5600000 4.645933 scaffold_85 252062 441546 0.00449387562802 0.00106072258498 0.00449334205 0.00106423916999 0.396456592988 0.398346710754 0.05516 0.037517679593 0.00268 0.00282873487999 0.072 Chr2 157769492

N00009 5600000 5800000 4.61264 scaffold_85 58561 252062 0.00408690078935 0.000934321061037 0.00415648442021 0.000951799762095 0.398842464998 0.395565996976 0.05278 0.0361548519129 0.02097 0.0216174593413 0.0646666666667 Chr2 157769492

N00009 6000000 6200000 2.972935 scaffold_85 1746371 1949801 0.00481518377525 0.00119599645345 0.00483214429774 0.00119994019493 0.423390142696 0.423047719483 0.044715 0.0329253305805 0.00685 0.00252666764981 0.05 Chr2 157769492

N00009 6200000 6400000 2.972935 scaffold_85 1550203 1746371 0.004475187252 0.00125827493172 0.0045537094659 0.00128224706167 0.386348991201 0.38539946723 0.069715 0.0538008237837 0.023075 0.021547856939 0.032 Chr2 157769492

N00009 6400000 6600000 2.972935 scaffold_85 1352796 1550203 0.00463884805548 0.000965181380052 0.0046557536064 0.000967574627837 0.380716250394 0.379519539347 0.071885 0.0753012811096 0.003455 0.00198067950985 NA Chr2 157769492

N00009 6600000 6800000 2.972935 scaffold_85 1157912 1352796 0.00427920960143 0.00112515381313 0.00431878596173 0.00113887306539 0.384891655009 0.385757938684 0.088455 0.0771535888015 0.010935 0.0113400792266 0.099 Chr2 157769492

N00009 7000000 7200000 2.972935 scaffold_42 89501 297037 0.00388084216836 0.00115276236143 0.00390687674008 0.00116236356523 0.426963686851 0.432446456171 0.083575 0.0743678205227 0.01083 0.00847081952047 0.297666666667 Chr2 157769492

N00009 7200000 7400000 2.972935 scaffold_42 297037 489248 0.00400321630339 0.00112523802651 0.00402089130064 0.00113008904273 0.436167997984 0.433035048471 0.05595 0.0440141303047 0.00535 0.00559281206591 0.0935 Chr2 157769492

N00009 7400000 7600000 2.972935 scaffold_42 489248 676616 0.0035699230366 0.000901539105909 0.00360085376825 0.000913073661117 0.439574039592 0.440059980329 0.050815 0.00675675675676 0.019115 0.0204036975364 0.057 Chr2 157769492

N00009 7600000 7800000 2.972935 scaffold_42 676616 886029 0.00450699792915 0.00139468231393 0.0046385169731 0.00146648327547 0.418415606633 0.415985214672 0.070995 0.0621690152951 0.049935 0.0596811086227 0.0695 Chr2 157769492

N00009 7800000 8000000 2.972935 scaffold_42 886029 1073870 0.00435129715458 0.00112173265994 0.00439278842906 0.00113482868126 0.404752490906 0.403738101534 0.079185 0.0313190411039 0.013175 0.015598298561 0.062 Chr2 157769492

N00009 8000000 8200000 2.972935 scaffold_42 1073908 1263333 0.00479544868422 0.00130382553278 0.00482267366772 0.00131271448736 0.379361160014 0.377236865845 0.079935 0.0513606968457 0.00553 0.00585983898641 0.034 Chr2 157769492

N00009 8200000 8400000 2.972935 scaffold_42 1263333 1456838 0.00507160601306 0.0014411635486 0.00507160601306 0.0014411635486 0.382581086629 0.38252015182 0.073845 0.0559675460582 0 0 NA Chr2 157769492

N00009 8400000 8600000 2.972935 scaffold_42 1456838 1640539 0.00443860608843 0.00107647526243 0.00443860608843 0.00107647526243 0.389263728706 0.391511714801 0.08494 0.0398092552572 0 0 NA Chr2 157769492

N00009 8600000 8800000 2.42801 scaffold_42 1640539 1830043 0.00427337472593 0.000940576968929 0.00427511484261 0.000945160717575 0.387105846541 0.390237980883 0.047425 0.0296827507599 0.00036 0.0134086879433 0.053 Chr2 157769492

N00009 8800000 9000000 2.075148 scaffold_42 1830043 2030490 0.00415752416878 0.0012689898712 0.00416752515141 0.00127280672647 0.414055660632 0.420851086493 0.054755 0.0642913089246 0.00294 0.00372667089056 0.053 Chr2 157769492

N00009 9200000 9400000 2.075148 scaffold_42 2217723 2413355 0.00477841310008 0.00125345585383 0.00478772105681 0.00126429623887 0.421734081462 0.427426302989 0.071315 0.0362568495952 0.0117 0.0135253946185 0.226 Chr2 157769492

N00009 9400000 9600000 2.075148 scaffold_42 2413355 2605030 0.00435988592423 0.00111029971267 0.00437837062423 0.00111529707555 0.414557515385 0.416683177035 0.085965 0.0510134341985 0.00547 0.00590061301683 0.0275 Chr2 157769492

N00009 9600000 9800000 2.075148 scaffold_42 2605030 2785178 0.0037282899705 0.00100012459675 0.0037523672974 0.00103880611243 0.414495239623 0.418221759217 0.096685 0.0507027555121 0.01474 0.0464062881631 0.08 Chr2 157769492

N00009 9800000 10000000 2.075148 scaffold_42 2785178 2978969 0.00401563798831 0.00109021933967 0.0040182459288 0.00109021933967 0.403323533727 0.401033494873 0.06506 0.0467875185122 0.001185 0 NA Chr2 157769492

N00009 10000000 10200000 2.075148 scaffold_42 2978969 3173916 0.0041077638353 0.00118847872573 0.0041221366699 0.00119470564151 0.406942809154 0.403263416083 0.08171 0.0688546117663 0.009175 0.0083407285057 0.055 Chr2 157769492

N00009 10200000 10400000 2.075148 scaffold_42 3173916 3372310 0.00444986934649 0.00106680142849 0.00451635254838 0.00109346112247 0.412868186823 0.414292581867 0.098675 0.0779761484722 0.021905 0.0250914846215 0.07125 Chr2 157769492

N00009 10400000 10600000 2.075148 scaffold_42 3372310 3558562 0.00374380845398 0.000994206292208 0.00381152199958 0.00101895687423 0.39145651296 0.392099381928 0.071915 0.0364184008762 0.03032 0.0356613620256 0.0466666666667 Chr2 157769492

N00009 10600000 10800000 2.075148 scaffold_42 3558747 3749502 0.00306642248146 0.000909914166072 0.00306642248146 0.000909914166072 0.412061862412 0.41499834679 0.06243 0.0295300254253 0 0 NA Chr2 157769492

N00009 10800000 11000000 2.075148 scaffold_42 3749502 3942768 0.00332949746832 0.000946956048076 0.00334497056586 0.00095512107626 0.43375349058 0.433913197978 0.054905 0.039075678081 0.016405 0.0175095464282 0.034 Chr2 157769492

N00009 11000000 11200000 2.075148 scaffold_42 3942768 4141232 0.0044173055135 0.00119372227505 0.004442322662 0.00120276950465 0.396924378612 0.396577612519 0.07742 0.0691309255079 0.006785 0.00629837149307 0.038 Chr2 157769492

N00009 11200000 11400000 2.075148 scaffold_42 4141232 4337052 0.00395534349599 0.00096995278071 0.00401377323941 0.00098154551562 0.400296503772 0.399062962354 0.06437 0.0431314472475 0.023525 0.0236135226228 0.0365 Chr2 157769492

N00009 11400000 11600000 2.075148 scaffold_42 4337052 4538213 0.00362613113175 0.000899102397872 0.00362613113175 0.000899400203553 0.424389587315 0.426440245067 0.052505 0.0628948951337 0 0.000223701413296 NA Chr2 157769492

N00009 11600000 11800000 2.075148 scaffold_42 4538213 4731112 0.00366406472834 0.000871322975937 0.00366406472834 0.000871322975937 0.429865962899 0.434439186981 0.066935 0.0472838117357 0 0 NA Chr2 157769492

N00009 11800000 12000000 2.075148 scaffold_42 4731112 4917710 0.00404468848906 0.00108679671586 0.00406809720234 0.00109401103432 0.403776021427 0.408167388167 0.12392 0.0829215747221 0.009795 0.0121919849087 0.0625 Chr2 157769492

N00009 12000000 12200000 2.075148 scaffold_42 4917710 5107296 0.0041958487125 0.00114209903445 0.00431793699694 0.00118355308511 0.389350014729 0.389614770007 0.052615 0.0267319316827 0.043205 0.044671020012 0.05625 Chr2 157769492

N00009 12200000 12400000 2.238523 scaffold_42 5107296 5296139 0.00438263165075 0.0010532103049 0.00446193410631 0.00107935673728 0.399264606705 0.402470134745 0.081685 0.0423950053748 0.02896 0.0331227527629 0.068 Chr2 157769492

N00009 12400000 12600000 2.269458 scaffold_42 5296139 5487600 0.0035998327848 0.000982620397858 0.0035998327848 0.000982620397858 0.422832448991 0.424024585783 0.06291 0.0398984649615 0 0 NA Chr2 157769492

N00009 12600000 12800000 2.269458 scaffold_42 5487600 5680496 0.00386094241418 0.00100539802966 0.00386094241418 0.00100539802966 0.407707285413 0.410178416915 0.05307 0.041291680491 0 0 NA Chr2 157769492

N00009 12800000 13000000 2.269458 scaffold_42 5680784 5869224 0.00371542347975 0.0010010728413 0.00371542347975 0.0010010728413 0.408213561821 0.413149026425 0.109295 0.0588197834855 0.001665 0 NA Chr2 157769492

N00009 13000000 13200000 2.269458 scaffold_42 5869449 6063203 0.00452657267665 0.00106618757364 0.00458183207786 0.00108001697832 0.4057387336 0.407773698023 0.089095 0.0464764598408 0.013495 0.0140797093221 0.0503333333333 Chr2 157769492

N00009 13200000 13400000 2.269458 scaffold_42 6063203 6255044 0.00456418630522 0.000927200537547 0.00457745804571 0.000930574740003 0.373285500883 0.372887894033 0.07655 0.0711735239078 0.003115 0.00324748098686 0.052 Chr2 157769492

N00009 13400000 13600000 2.269458 scaffold_42 6255045 6445035 0.00411737080647 0.000994846234044 0.00412672045037 0.000999946321642 0.378217799667 0.378887342774 0.062895 0.0445181325333 0.00421 0.00445286594031 0.052 Chr2 157769492

N00009 13600000 13800000 2.269458 scaffold_42 6445035 6642359 0.00459611001705 0.00101094926115 0.00459611001705 0.00101094926115 0.400469259131 0.404846491819 0.06855 0.0490513064807 0.00574 0 NA Chr2 157769492

N00009 13800000 14000000 2.269458 scaffold_42 6642359 6836382 0.00454615287281 0.00101438739074 0.00454615287281 0.00101438739074 0.398476236546 0.398964742651 0.07841 0.0620081124403 0 0 NA Chr2 157769492

N00009 14000000 14200000 2.269458 scaffold_42 6836382 7035961 0.00419691155664 0.00114703610075 0.0042074756366 0.00114831133738 0.387300892222 0.390524691691 0.06966 0.074266330626 0.003435 0.00345727756928 0.096 Chr2 157769492

N00009 14200000 14400000 2.269458 scaffold_42 7035961 7233316 0.00449533365308 0.00115609458679 0.00449533365308 0.00115609458679 0.396897170032 0.395504402408 0.065325 0.0827240252337 0 0 NA Chr2 157769492

N00009 14400000 14600000 2.269458 scaffold_42 7233316 7438459 0.0048216628936 0.00122711671614 0.0048216628936 0.00122711671614 0.391851352673 0.389963599371 0.05629 0.0752158250586 0 0 NA Chr2 157769492

N00009 14600000 14800000 2.269458 scaffold_42 7438459 7640459 0.00482523334209 0.00115145217894 0.00497475973208 0.00119898191147 0.391977332475 0.39347183062 0.08707 0.116262376238 0.04368 0.0439158415842 0.0544 Chr2 157769492

N00009 15000000 15200000 2.269458 scaffold_3 151294 342954 0.00448908047673 0.00113565030417 0.00448908047673 0.00113565030417 0.361462767892 0.360583690618 0.069315 0.0630021913806 0 0 NA Chr2 157769492

N00009 15200000 15400000 2.269458 scaffold_3 342954 538129 0.00430716177825 0.00107166097056 0.00430716177825 0.00107166097056 0.372993494816 0.374999306769 0.09842 0.0701165620597 0 0 NA Chr2 157769492

N00060 400000 600000 1.450763 scaffold_33 8131122 8324404 0.00355810569005 0.00105465908858 0.00363213467825 0.00108320738657 0.385563212194 0.382209177814 0.07324 0.0520586500554 0.026 0.0260034560901 0.047 Chr2 157769492

N00060 600000 800000 1.450763 scaffold_33 7939708 8131122 0.00422653502777 0.00102395674045 0.0043520872118 0.00105252982984 0.401584319476 0.397704122698 0.06116 0.0432047812595 0.038105 0.0410262572226 0.0535 Chr2 157769492

N00060 800000 1000000 1.450763 scaffold_33 7734590 7939708 0.00405606768013 0.00112113828387 0.00405606768013 0.00112113828387 0.402993324085 0.400006581942 0.085715 0.0912304137131 0 0 NA Chr2 157769492

N00060 1000000 1200000 1.450763 scaffold_33 7537215 7734590 0.00432695873485 0.00121604232352 0.00432912218781 0.0012181200547 0.396236205184 0.395115029452 0.08273 0.0792653578214 0.00736 0.00676377454085 0.23 Chr2 157769492

N00060 1200000 1400000 1.450763 scaffold_33 7346972 7536118 0.00421006744247 0.00123348551344 0.00430323008435 0.00126444739946 0.386795171268 0.380376384249 0.13433 0.0936895308386 0.03131 0.0305478307762 0.04525 Chr2 157769492

N00060 1400000 1600000 1.450763 scaffold_33 7153281 7346972 0.00399081216305 0.00115824192805 0.00403496715718 0.00117713328618 0.408951742236 0.406935884961 0.057205 0.0546179223609 0.018145 0.0187360280034 0.071 Chr2 157769492

N00060 1600000 1800000 0.7381903 scaffold_33 6960927 7153151 0.00397890975892 0.00130564046498 0.00398461476182 0.00131002214864 0.390227940049 0.391812213068 0.081235 0.08553042284 0.003645 0.00380805726652 0.06 Chr2 157769492

N00060 1800000 2000000 0.434968 scaffold_33 6753084 6960927 0.00453145477181 0.00151926798888 0.00453145477181 0.00151926798888 0.365512263934 0.362997431326 0.11934 0.108904317201 0 0 NA Chr2 157769492

N00060 2000000 2200000 0.434968 scaffold_33 6535244 6753084 0.00510744183076 0.00166432434027 0.00510744183076 0.00166432434027 0.351258518656 0.345100254165 0.132335 0.146603011385 0 0 NA Chr2 157769492

N00060 2400000 2600000 0.434968 scaffold_33 6124909 6329690 0.00449673728327 0.00160298798107 0.0045201870392 0.00161238047177 0.349584462409 0.348555166126 0.114205 0.12245764988 0.005585 0.00552785658826 0.165 Chr2 157769492

N00060 3600000 3800000 0.434968 scaffold_33 4903048 5108867 0.00402544658688 0.00167416608045 0.00405116696884 0.00169307746171 0.360497987562 0.356166526639 0.126245 0.121592272822 0.01077 0.0104655061 0.053 Chr2 157769492

N00060 4000000 4200000 0.434968 scaffold_33 4501947 4708412 0.0041179296151 0.00139796335707 0.0041179296151 0.00139796335707 0.353792981516 0.349812542207 0.1386 0.146078027753 0 0 NA Chr2 157769492

N00122 600000 800000 0.707063 scaffold_19 15237721 15432317 0.00395867603282 0.0012983520101 0.00395867603282 0.00129898544581 0.371181288668 0.364968542072 0.11301 0.0900737939115 0 0.00066291187897 0.024 Chr2 157769492

N00122 800000 1000000 0.707063 scaffold_19 15036531 15237721 0.00418742024617 0.00114612985398 0.00419863877359 0.00115049116412 0.382011566766 0.376362046545 0.10094 0.0980913564292 0.00669 0.00406580843978 0.024 Chr2 157769492

N00122 1000000 1200000 0.707063 scaffold_19 14835472 15036531 0.00390751400225 0.00129626607682 0.00393886943762 0.00130539783485 0.377737074809 0.375701428163 0.081935 0.087715546183 0.007655 0.00786833715477 0.024 Chr2 157769492

N00122 1200000 1400000 0.707063 scaffold_19 14625457 14835472 0.00393572096361 0.00112597592169 0.00396476597708 0.00113453063726 0.395103549574 0.393803737654 0.07528 0.0966978549151 0.00984 0.00967073780444 0.092 Chr2 157769492

N00122 1400000 1600000 0.707063 scaffold_19 14429617 14625457 0.00399691666335 0.00119497486091 0.00409741976405 0.00122687923455 0.401648750556 0.400608600333 0.08754 0.0762612336601 0.035665 0.0325214460784 0.142285714286 Chr2 157769492

N00122 1600000 1800000 0.707063 scaffold_19 14226512 14429617 0.00476325517881 0.001047592009 0.00480572927223 0.00105438008697 0.384714190391 0.382373852956 0.088585 0.0983136801162 0.011185 0.010664434652 0.141 Chr2 157769492

N00122 1800000 2000000 0.707063 scaffold_19 14030636 14226512 0.00451439407049 0.00131859685804 0.00452927747665 0.00132298421004 0.37727426109 0.378622122676 0.07028 0.0666697298291 0.003275 0.00334395229635 0.111 Chr2 157769492

N00122 2000000 2200000 0.707063 scaffold_19 13846238 14030636 0.00487056794834 0.00119475473063 0.00491327779502 0.00120646922706 0.375000746335 0.373252311389 0.104105 0.0764216531633 0.012875 0.0139860519095 0.111 Chr2 157769492

N00116 0 200000 0.707063 scaffold_19 13510123 13702201 0.00514596031248 0.0014335558414 0.00522238794609 0.00145491420065 0.373822793415 0.371475886814 0.09714 0.0736471641729 0.02897 0.0307583377586 0.085 Chr2 157769492

N00116 200000 400000 0.707063 scaffold_19 13314977 13510123 0.00452975152425 0.00106371731062 0.00455239592104 0.00107219831384 0.380123539002 0.379411714919 0.104325 0.0808112900085 0.00944 0.00877291873777 0.065 Chr2 157769492

N00116 400000 600000 0.707063 scaffold_19 13110352 13314977 0.00444811587788 0.00130435543283 0.00444996676849 0.00130594020964 0.387072654938 0.384006715863 0.105875 0.117082467929 0.000885 0.000879657910812 0.028 Chr2 157769492

N00116 600000 800000 0.707063 scaffold_19 12908838 13110352 0.0039902741727 0.000999411439081 0.00406614102228 0.00102347135397 0.397979523153 0.397533983763 0.11843 0.104662703336 0.025625 0.02231606737 0.03925 Chr2 157769492

N00116 800000 1000000 0.707063 scaffold_19 12700652 12908838 0.00515429699691 0.00126816080616 0.00527772500097 0.0013383245481 0.404148066409 0.404361670848 0.071465 0.0688999260277 0.124775 0.108004380698 0.1085 Chr2 157769492

N00116 1000000 1200000 0.707063 scaffold_19 12508235 12700652 0.00474943514643 0.00135110378521 0.00479902409732 0.00137536008812 0.361750155523 0.358510596906 0.08359 0.104013678625 0.0165 0.016656532427 0.107 Chr2 157769492

N00116 1200000 1400000 0.707063 scaffold_19 12304591 12508235 0.00494129947446 0.00133587367047 0.00494129947446 0.00133587367047 0.372957821975 0.366992350565 0.107905 0.120366914812 0 0 NA Chr2 157769492

N00116 1400000 1600000 0.707063 scaffold_19 12102597 12300629 0.00436759566517 0.00109972022327 0.00437305991002 0.00110349076232 0.38213938597 0.379876887649 0.091065 0.101039225984 0.005655 0.00902884382322 0.112 Chr2 157769492

N00116 1600000 1800000 0.707063 scaffold_19 11909885 12102597 0.0032648227664 0.00104074250015 0.00329439448199 0.00105770254035 0.393734142678 0.389692600787 0.065275 0.0396290838142 0.02391 0.0260180995475 0.0553333333333 Chr2 157769492

N00116 1800000 2000000 0.707063 scaffold_19 11713167 11909885 0.00348342715033 0.000939558331655 0.00351650801333 0.000949650747386 0.405453354899 0.404018324175 0.081965 0.0681940646001 0.00994 0.0103549243079 0.0565 Chr2 157769492

N00116 2000000 2200000 0.5704416 scaffold_19 11525453 11713167 0.0033433406596 0.000974715518166 0.00338462181862 0.000991404240252 0.392279960219 0.391981017607 0.055315 0.0347603268803 0.017475 0.0224863355956 0.037 Chr2 157769492

N00116 2200000 2400000 0.3914204 scaffold_19 11331860 11524733 0.00477481091559 0.00128452124143 0.00477481091559 0.00128452124143 0.380518794242 0.380311602794 0.10517 0.0972401528467 0 0 0.037 Chr2 157769492

N00082 0 200000 0.3914204 scaffold_19 6798968 6988330 0.00409785046676 0.00111512236528 0.00412727750077 0.00111957778545 0.404013446294 0.400240303636 0.063335 0.053812274902 0.01082 0.0124629017438 0.0345 Chr2 157769492

N00082 200000 400000 0.3914204 scaffold_19 6988330 7176964 0.00475899967241 0.00114288786767 0.00475899967241 0.00114288786767 0.383357224034 0.382850801643 0.09436 0.0664355312404 0 0 NA Chr2 157769492

N00082 400000 600000 0.3914204 scaffold_19 7176964 7368476 0.00423642536049 0.00113033799236 0.00423642536049 0.00113033799236 0.383115390803 0.38178520606 0.103945 0.0887933915368 0 0 NA Chr2 157769492

N00082 600000 800000 0.3914204 scaffold_19 7368476 7557585 0.00479545209874 0.00106205770302 0.00480691888032 0.00106474067015 0.380559957739 0.380482321227 0.100605 0.0887001676282 0.003605 0.00478031188362 0.054 Chr2 157769492

N00082 800000 1000000 0.3914204 scaffold_19 7557585 7751255 0.00409107887545 0.0010017983677 0.0041145766886 0.00101071693476 0.390391488654 0.389420005787 0.070955 0.0504827799866 0.01062 0.0103216812103 0.053 Chr2 157769492

N00082 1000000 1200000 0.3914204 scaffold_19 7751255 7950998 0.00434858180849 0.00130877740073 0.00439169189373 0.00132282912091 0.391461522378 0.391483672619 0.064185 0.0715970021478 0.01206 0.0120955427725 0.052 Chr2 157769492

N00082 1200000 1400000 0.3914204 scaffold_19 7950998 8145290 0.0041586869044 0.0013093917706 0.00419248388216 0.00131878919584 0.400657145901 0.403774742308 0.0739 0.0608465608466 0.012165 0.0153325921808 0.049 Chr2 157769492

N00082 1400000 1600000 0.3914204 scaffold_19 8145290 8347100 0.00488212151762 0.00105625139121 0.00493407709972 0.00106830735746 0.391026814067 0.39154263141 0.06261 0.0542440909767 0.015915 0.0134829790397 0.049 Chr2 157769492

N00082 1600000 1800000 0.3914204 scaffold_19 8347100 8542198 0.00440049773583 0.00113652047164 0.00440049773583 0.00113652047164 0.403576560573 0.406402200383 0.08012 0.0922869532235 0 0 NA Chr2 157769492

N00082 1800000 2000000 0.3914204 scaffold_19 8542198 8741217 0.00398745738814 0.00102748686206 0.0040192302675 0.0010385302403 0.391045415125 0.391318684942 0.079695 0.0623407815334 0.010525 0.0110793441832 0.029 Chr2 157769492

N00082 2000000 2200000 0.3914204 scaffold_19 8741649 8936935 0.00354021529028 0.00128408320781 0.00354833477328 0.00128406136423 0.420557792791 0.420833515451 0.08232 0.0693649314339 0.002515 0.00165398441261 0.082 Chr2 157769492

N00082 2200000 2400000 0.3914204 scaffold_19 8936935 9140252 0.00380158443689 0.00111181009777 0.00385325530696 0.00112291653303 0.387876530012 0.384942957497 0.06217 0.057211153027 0.021705 0.0218230644757 0.063 Chr2 157769492

N00082 2400000 2600000 0.3914204 scaffold_19 9140252 9333403 0.00436000610881 0.00123910473673 0.00437763929656 0.00124120954708 0.394059696305 0.388643067847 0.06458 0.0406417776765 0.00661 0.00605225963107 0.062 Chr2 157769492

N00082 2600000 2800000 0.3914204 scaffold_19 9333403 9536077 0.00456315355052 0.00116315426491 0.00456315355052 0.00116315426491 0.387515513367 0.385803591916 0.106565 0.110650601458 0 0 NA Chr2 157769492

N00082 2800000 3000000 0.3914204 scaffold_19 9536942 9739774 0.00482450124212 0.00143423643666 0.00482450124212 0.00143423643666 0.367037666908 0.363588481199 0.09813 0.111565236255 0 0 NA Chr2 157769492

N00082 3000000 3200000 0.3914204 scaffold_19 9739774 9933749 0.00441122810069 0.00135335400822 0.00441122810069 0.00135335400822 0.359613150161 0.356470156556 0.098805 0.0910632813507 0 0 NA Chr2 157769492

N00301 0 200000 2.155754 scaffold_19 6608494 6796723 0.00451340003796 0.00105356057072 0.00475333949585 0.00112297758524 0.390517144533 0.391262743366 0.11212 0.0672903750219 0.07043 0.0648837320498 0.0725 Chr2 157769492

N00301 200000 400000 2.755627 scaffold_19 6413466 6605677 0.00450295107125 0.00106477813255 0.00453593759805 0.00107513007191 0.3866270851 0.381535749199 0.106395 0.0584565919744 0.00884 0.00977051261374 0.0685 Chr2 157769492

N00027 0 200000 0.1791082 scaffold_24 2530757 2735367 0.00387657095854 0.00119375820306 0.00387657095854 0.00121556486393 0.353513617314 0.350189873772 0.10564 0.102981281462 0 0.0178143785739 NA Chr2 157769492

N00027 400000 600000 0.1791082 scaffold_24 2133363 2333872 0.00389271747418 0.00150905136515 0.00392598151429 0.00152697017813 0.352629242743 0.350548007326 0.134185 0.13119610591 0.010555 0.0160840660519 0.059 Chr2 157769492

N00027 600000 800000 0.1791082 scaffold_24 1930670 2133363 0.00435192895729 0.00167548488879 0.0043846565245 0.00168613753553 0.349821892537 0.346406774317 0.10353 0.0993127537705 0.008385 0.00723754643722 0.0665 Chr2 157769492

N00027 800000 1000000 0.1791082 scaffold_24 1731137 1930670 0.00382294821103 0.00132500334335 0.00382294821103 0.00132500334335 0.354549115977 0.352706012848 0.13413 0.134920038289 0.00033 0 0.073 Chr2 157769492

N00027 1000000 1200000 0.1791082 scaffold_24 1536213 1731137 0.00406027952121 0.00119599620635 0.0040763638857 0.00120044160078 0.358596458408 0.360204041327 0.10828 0.108514087542 0.00531 0.00507890254663 0.042 Chr2 157769492

N00027 1200000 1400000 0.1791082 scaffold_24 1347687 1536143 0.00392096493265 0.00112717123093 0.00403725931436 0.00117255188766 0.350997074104 0.353085880812 0.11062 0.076537759477 0.041485 0.0489557244131 0.092 Chr2 157769492

N00027 1400000 1600000 0.1791082 scaffold_24 1144782 1347687 0.00459904898931 0.0014477890513 0.00459904898931 0.0014477890513 0.358541743288 0.357711744021 0.136385 0.130617776792 0 0 NA Chr2 157769492

N00027 2400000 2600000 0.1791082 scaffold_24 150234 350539 0.00453793642782 0.00112727543376 0.00457753898348 0.00113724862042 0.354317822893 0.354667295788 0.10192 0.11124535084 0.00795 0.00835725518584 0.021 Chr2 157769492

N00027 2800000 3000000 0.1791082 scaffold_19 68226 264372 0.00507089162159 0.00141153489541 0.00510533655959 0.0014235766738 0.375791412782 0.376036685442 0.069305 0.081342469385 0.00991 0.0094980269799 0.0825 Chr2 157769492

N00027 3000000 3200000 0.1791082 scaffold_19 264372 465112 0.00433600330061 0.00135975763808 0.00439680784305 0.00138909478682 0.400303078641 0.398046370245 0.092475 0.0643568795457 0.029125 0.0335508618113 0.0483333333333 Chr2 157769492

N00027 3200000 3400000 0.7377036 scaffold_19 465112 653913 0.003063967454 0.000872889177498 0.00309097123824 0.000893189810965 0.371493791916 0.370246497274 0.06829 0.036895991017 0.022775 0.0272403218203 NA Chr2 157769492

N00027 3400000 3600000 0.789313 scaffold_19 653913 848645 0.00322881678197 0.000920059531546 0.00323648681874 0.000925663553921 0.388622689577 0.387921754542 0.071485 0.0436805455703 0.01003 0.0089148162603 NA Chr2 157769492

N00027 3600000 3800000 0.789313 scaffold_19 848645 1033408 0.00288733712546 0.000824304506251 0.00292970504424 0.000833080735933 0.399100362192 0.405075687624 0.081955 0.040673727965 0.0226 0.0225369798066 0.0495 Chr2 157769492

N00027 3800000 4000000 0.789313 scaffold_19 1033408 1228326 0.00365772323024 0.00103069945927 0.00365772323024 0.00103069945927 0.396131244418 0.397048035126 0.08668 0.0678182620384 0 0 NA Chr2 157769492

N00027 4000000 4200000 0.789313 scaffold_19 1228326 1421833 0.00355424077867 0.000889033542241 0.0035522133182 0.000892157904494 0.404800679742 0.405622728525 0.05784 0.0399003653615 0.008365 0.0088007152196 0.105 Chr2 157769492

N00027 4200000 4400000 0.789313 scaffold_19 1421833 1613431 0.00401915301392 0.000967205189428 0.00402422403229 0.000970208099528 0.396348528955 0.40161808267 0.0672 0.0497447781292 0.009485 0.00944686270212 0.105 Chr2 157769492

N00027 4400000 4600000 1.291472 scaffold_19 1613431 1817832 0.00484651938256 0.00126097712457 0.00497576229926 0.00130131882547 0.373319342266 0.375979305248 0.08839 0.0847109358565 0.032365 0.0322454391123 0.048 Chr2 157769492

N00027 4600000 4800000 3.004456 scaffold_19 1817832 2026800 0.00481034024419 0.00138908450387 0.00481854662736 0.00139022656757 0.36743410534 0.373552557617 0.1004 0.14947264653 0.005695 0.00515389916159 0.031 Chr2 157769492

N00027 4800000 5000000 3.402083 scaffold_19 2026800 2222731 0.00397133008382 0.0014522146816 0.00405119513269 0.00148979493857 0.425679084315 0.424706429228 0.068705 0.0494153554057 0.02797 0.0320316846237 0.0668 Chr2 157769492

N00027 5000000 5200000 3.303855 scaffold_19 2222731 2414702 0.00475208261896 0.00135103420114 0.0048132597702 0.00137391806455 0.400764657833 0.397616128666 0.05705 0.0506326476395 0.017145 0.0192529079913 0.056 Chr2 157769492

N00027 5200000 5400000 3.888923 scaffold_19 2414702 2609442 0.00450196712782 0.00124805393373 0.00454038911345 0.00126115897105 0.427247837039 0.422151638139 0.045995 0.0332956762863 0.015175 0.0171972886926 0.077 Chr2 157769492

N00027 5400000 5600000 0.9509804 scaffold_19 2609442 2798943 0.00378356640722 0.00104802482692 0.00379644636477 0.00105409834184 0.408989292858 0.407319946089 0.107715 0.0820734455227 0.009215 0.00982580566857 0.125666666667 Chr2 157769492

N00027 5600000 5800000 0.9509804 scaffold_19 2798943 3006176 0.00364685139166 0.000862436645619 0.00364685139166 0.000862436645619 0.392476917945 0.39066639144 0.127925 0.123045074867 0 0 NA Chr2 157769492

N00027 5800000 6000000 1.052257 scaffold_19 3010535 3207139 0.00398542737044 0.00109753831708 0.00405152487804 0.00111207938995 0.378352867599 0.380481792844 0.088675 0.0878466358772 0.02689 0.0268712742365 0.0395 Chr2 157769492

N00027 6000000 6200000 2.589384 scaffold_19 3207139 3408215 0.00379055450142 0.00118726685208 0.0038763606051 0.00122442941343 0.387730698073 0.387782287321 0.08902 0.0663480475044 0.029055 0.0357078915435 0.0435 Chr2 157769492

N00027 6200000 6400000 1.789298 scaffold_19 3408215 3602978 0.00387290287634 0.00119503317462 0.00406293063983 0.00129860637838 0.371953530626 0.368466486921 0.07612 0.0571720501327 0.0772 0.0861970702854 0.139 Chr2 157769492

N00027 6400000 6600000 0.5964326 scaffold_19 3602978 3795650 0.00399100406701 0.00134752339616 0.00405508086533 0.00138261771028 0.390549246786 0.390738052607 0.081975 0.0851083707025 0.02842 0.0294593921276 0.0663333333333 Chr2 157769492

N00027 6600000 6800000 0.03587571 scaffold_19 3795650 3990921 0.00406403284186 0.00115788159129 0.0041426369681 0.0011861735743 0.383595720826 0.384601665027 0.10783 0.0816762345663 0.023885 0.0280994105628 0.0696666666667 Chr2 157769492

N00027 6800000 7000000 0.9013772 scaffold_19 3990921 4180165 0.00419891066534 0.00122227681415 0.00425160682793 0.00123817182358 0.388186881703 0.390535123386 0.108805 0.0951945636321 0.014125 0.0149172496882 0.045 Chr2 157769492

N00027 7000000 7200000 0.9013772 scaffold_19 4180165 4379653 0.00462160669269 0.00124775092827 0.00465337767998 0.00125709408043 0.370078960946 0.369209584137 0.085115 0.0959355951235 0.007125 0.00714328681424 0.04 Chr2 157769492

N00027 7200000 7400000 0.9013772 scaffold_19 4379653 4579928 0.00448669771765 0.00129823439547 0.00450865620225 0.00130876344619 0.385002769804 0.38855594152 0.07456 0.0754113094495 0.00838 0.0101260766446 0.073 Chr2 157769492

N00027 7400000 7600000 0.9013772 scaffold_19 4579928 4772350 0.00440433245261 0.00130978661844 0.00443598748265 0.00132016373987 0.37424744001 0.373013043601 0.10323 0.0688019041482 0.012745 0.0125401461371 0.061 Chr2 157769492

N00027 7600000 7800000 0.9013772 scaffold_19 4772350 4959048 0.00413556312522 0.000983032183368 0.00413556312522 0.000983032183368 0.369727932251 0.371256814696 0.102445 0.067499384032 0 0 NA Chr2 157769492

N00027 8000000 8200000 0.9013772 scaffold_19 5147995 5339663 0.00464108781526 0.00134466182708 0.00464724069664 0.00134994930767 0.369729201403 0.371239987811 0.07304 0.0604743619175 0.00591 0.0061825656865 0.042 Chr2 157769492

N00027 8200000 8400000 0.9013772 scaffold_19 5339663 5530645 0.00437192933548 0.000991923799567 0.00437192933548 0.000991923799567 0.362578230779 0.36278078262 0.117855 0.092846446262 0 0 NA Chr2 157769492

N00027 8400000 8600000 0.9013772 scaffold_19 5530645 5724700 0.00361243426584 0.000968544371403 0.00361243426584 0.000968544371403 0.361363014884 0.361479524728 0.086635 0.0744943443869 0 0 NA Chr2 157769492

N00027 8600000 8800000 0.9013772 scaffold_19 5724700 5919162 0.00388005828713 0.00101013880104 0.00388391996161 0.00101056836951 0.377300579446 0.37842912277 0.08223 0.0586027090126 0.007165 0.0040882023223 0.033 Chr2 157769492

N00027 8800000 9000000 0.9013772 scaffold_19 5919162 6114943 0.00456823646491 0.00111989078686 0.00456823646491 0.00111989078686 0.366121611944 0.364975818243 0.066415 0.0585194681813 0 0 NA Chr2 157769492

N00027 9000000 9200000 2.349722 scaffold_19 6114943 6302828 0.00418342212029 0.00120612067648 0.00418794485031 0.00120612067648 0.377279205502 0.376369127962 0.09472 0.0556829975783 0.003535 0 NA Chr2 157769492

N00010 200000 400000 0.8653143 scaffold_38 4884378 5082491 0.0034436593048 0.00136628239097 0.00346079285167 0.00137359686603 0.352300977536 0.347479846918 0.073315 0.0954505761863 0.00519 0.00523943406036 0.036 Chr2 157769492

N00010 400000 600000 0.8653143 scaffold_38 5082491 5282426 0.00424129994385 0.0016134965313 0.00424129994385 0.0016134965313 0.365031386733 0.363166571379 0.09921 0.10026258534 0 0 NA Chr2 157769492

N00010 800000 1000000 6.654067 scaffold_38 5479005 5667066 0.00375288356831 0.00112824439348 0.00376730694087 0.00113486527303 0.394709137062 0.398511505888 0.11751 0.0850894124779 0.00574 0.00566305613604 0.1565 Chr2 157769492

N00010 1000000 1200000 6.801739 scaffold_38 5667131 5856350 0.00348798451607 0.00110295191823 0.00348798451607 0.00110295191823 0.401917640381 0.405718062242 0.06937 0.0552692911388 0 0 NA Chr2 157769492

N00010 1200000 1400000 6.09111 scaffold_38 5856350 6053302 0.00437228439997 0.00112819974868 0.00453812142892 0.00119879292868 0.383066796626 0.385003317071 0.0912 0.0776432836427 0.05708 0.0687578699379 0.066 Chr2 157769492

N00010 1400000 1600000 0 scaffold_38 6053302 6239215 0.00365137319629 0.00113405115132 0.0036733072338 0.00115073965701 0.396016125207 0.39895482093 0.089 0.0338007562677 0.01873 0.0179815289947 NA Chr2 157769492

N00010 1600000 1800000 0 scaffold_38 6239215 6431270 0.00423269908547 0.00129657006108 0.00425809134303 0.0013088241854 0.401811193961 0.404916917144 0.1041 0.0678347348416 0.01392 0.0100127567624 0.079 Chr2 157769492

N00010 1800000 2000000 0 scaffold_38 6431270 6621825 0.00438477961614 0.00120800063773 0.00442466635052 0.0012212170757 0.410429081556 0.409328618799 0.07889 0.0647372149773 0.01176 0.0130828369762 NA Chr2 157769492

N00010 2200000 2400000 0.963494 scaffold_38 6807963 7004108 0.00416795720599 0.00125080865553 0.0041941188912 0.00125901784166 0.388938750618 0.385608514317 0.101365 0.100135104132 0.0079 0.008075658314 0.048 Chr2 157769492

N00010 2400000 2600000 2.934277 scaffold_38 7004108 7190802 0.00413819223496 0.00116911239949 0.00423597482368 0.00120290628796 0.367448170863 0.364923293538 0.10872 0.0807203230955 0.029935 0.0360107984188 0.056 Chr2 157769492

N00010 2600000 2800000 2.934277 scaffold_38 7190802 7386233 0.00471670605815 0.00142887215037 0.00471886933695 0.00142887215037 0.378052844041 0.377970581286 0.08013 0.0632397112024 6,00E-04 0 0.052 Chr2 157769492

N00010 2800000 3000000 2.934277 scaffold_38 7386233 7578213 0.00429631304647 0.00116160269898 0.00432143966618 0.00116771392933 0.404147744821 0.403128156196 0.09136 0.0640535472445 0.012575 0.00867798729034 0.116 Chr2 157769492

N00010 3000000 3200000 2.934277 scaffold_38 7578213 7774708 0.00419705816847 0.00108274275552 0.00434168754823 0.00112093472568 0.393303054798 0.388967564322 0.066495 0.0630245044403 0.048085 0.0474770350391 0.0906666666667 Chr2 157769492

N00010 3200000 3400000 2.911703 scaffold_38 7774708 7961415 0.00496406821905 0.00110215747514 0.00504970335478 0.00113090673803 0.385110536275 0.382526214168 0.107165 0.0675711140986 0.02652 0.0296186002667 0.05525 Chr2 157769492

N00010 3400000 3600000 2.913183 scaffold_38 7961415 8156834 0.00454948920544 0.0011340256927 0.00463511620441 0.00115766337248 0.373167683953 0.372961327541 0.127465 0.104345022746 0.029575 0.03229982755 0.073 Chr2 157769492

N00010 3600000 3800000 2.441463 scaffold_38 8156834 8360895 0.00493673190889 0.00116881375881 0.00493673190889 0.00116881375881 0.354302249645 0.355925054009 0.121915 0.129083950387 0 0 NA Chr2 157769492

N00010 4000000 4200000 0.2326907 scaffold_24 13372824 13566070 0.0052685081811 0.000706673931688 0.0052685081811 0.000706673931688 0.358967621383 0.356674852041 0.113005 0.113797957008 0 0 NA Chr2 157769492

N00010 4200000 4400000 0 scaffold_24 13184861 13372824 0.00476175514827 0.00127960652942 0.00476175514827 0.00127960652942 0.362736595371 0.359164829825 0.13574 0.0920553513191 0 0 NA Chr2 157769492

N00010 4400000 4600000 0 scaffold_24 12990364 13184008 0.0039023269662 0.00117487392725 0.0039023269662 0.00117487392725 0.380790209597 0.38270047098 0.096545 0.0672574414906 0 0 NA Chr2 157769492

N00010 4600000 4800000 0.9771624 scaffold_24 12800116 12990364 0.00405267628172 0.00125856614027 0.00409169779944 0.00127297168882 0.387749927975 0.392886720236 0.093095 0.0534933350153 0.01332 0.01630503343 0.062 Chr2 157769492

N00010 4800000 5000000 1.722892 scaffold_24 12601913 12800116 0.0045094463449 0.00111804298584 0.00451130616374 0.00111807322922 0.399526603082 0.401661975462 0.068675 0.0594844679445 5,00E-04 0.000504533231081 0.059 Chr2 157769492

N00010 5000000 5200000 1.722892 scaffold_24 12417163 12601913 0.00457390303124 0.0011843567515 0.00460189341805 0.00119434276337 0.404116400488 0.401788912835 0.110175 0.0648876860622 0.010175 0.0111393775372 0.0895 Chr2 157769492

N00010 5200000 5400000 1.722892 scaffold_24 12223530 12417163 0.00372178280934 0.00115246935349 0.00374903718066 0.00116383871338 0.422133233036 0.421954915186 0.07261 0.0362748085295 0.013275 0.0128490494905 0.092 Chr2 157769492

N00010 5400000 5600000 1.722892 scaffold_24 12031217 12223530 0.00394921303179 0.00105163758936 0.00397597117148 0.00105855823196 0.400381472941 0.401569775762 0.08276 0.0597463510007 0.00875 0.0108209013431 0.0605 Chr2 157769492

N00010 5600000 5800000 4.376635 scaffold_24 11841976 12031217 0.00348254691503 0.000972676537215 0.00348679369964 0.000973592408232 0.407527166412 0.409401263359 0.07819 0.0443878440718 0.001715 0.00144788919949 0.05 Chr2 157769492

N00010 5800000 6000000 3.246928 scaffold_24 11649638 11841976 0.00302303821719 0.000893439953174 0.003053424702 0.000906984243458 0.403132253733 0.403626162453 0.04215 0.0242541775416 0.018565 0.0193201551435 0.05 Chr2 157769492

N00010 6000000 6200000 4.74808 scaffold_24 11464056 11649638 0.00380188504547 0.00112989830585 0.00380050999238 0.00112995399796 0.420681984487 0.420445728807 0.103285 0.0660839952151 0.00582 0.0064661443459 NA Chr2 157769492

N00010 6200000 6400000 4.74808 scaffold_24 11267459 11464056 0.00454207866211 0.00121787685628 0.00465495465445 0.00126812004195 0.389122745915 0.38571596382 0.09182 0.0909830770561 0.04898 0.0513995635742 0.06925 Chr2 157769492

N00010 6600000 6800000 2.434247 scaffold_24 10883536 11086858 0.00420503188692 0.00130239923784 0.00425630335759 0.00132567304771 0.416112290116 0.414264899054 0.07552 0.0723089483676 0.018455 0.0207700101317 0.0373333333333 Chr2 157769492

N00010 6800000 7000000 2.434247 scaffold_24 10689139 10883536 0.00406591030161 0.00117581064774 0.00410102073241 0.00118776317164 0.411984770733 0.411592677726 0.0814 0.0374645699265 0.01359 0.0139199679007 0.095 Chr2 157769492

N00010 7000000 7200000 2.434247 scaffold_24 10488831 10689139 0.00396497324534 0.00108905462854 0.00398871411092 0.00110624458934 0.419094217505 0.419530785448 0.05549 0.0529085208778 0.01959 0.0179573456876 0.078 Chr2 157769492

N00010 7200000 7400000 2.851215 scaffold_24 10295646 10488831 0.00434090910607 0.00109780008846 0.00449019907994 0.00115344528663 0.384853454188 0.385091677615 0.07458 0.0614178119419 0.0503 0.0523487848435 0.053 Chr2 157769492

N00010 7400000 7600000 3.420253 scaffold_24 10107783 10295646 0.00451644341052 0.000886533118127 0.00460257184656 0.00091226832047 0.381818181818 0.383615327196 0.080945 0.0558651783481 0.028375 0.0310226068997 0.04 Chr2 157769492

N00010 7600000 7800000 2.2584 scaffold_24 9916566 10107783 0.00419613770953 0.00125319936453 0.00425903107888 0.00128595917353 0.40680538499 0.407389708156 0.084635 0.063268433246 0.025985 0.0294482185161 0.06325 Chr2 157769492

N00010 8000000 8200000 0.8254469 scaffold_24 9547242 9740293 0.00453290676932 0.00122307637402 0.00453290676932 0.00122307637402 0.383212383451 0.383601340675 0.0855 0.0796421670957 0 0 NA Chr2 157769492

N00010 8200000 8400000 0.8254469 scaffold_24 9346456 9547242 0.00490874204023 0.00114246886712 0.00490874204023 0.00114246886712 0.376418702722 0.376361010504 0.113375 0.113583616388 0 0 NA Chr2 157769492

N00010 8400000 8600000 0.8254469 scaffold_24 9140193 9346456 0.00479859282125 0.00123435513652 0.00482619813372 0.00124661736302 0.38450625584 0.386464034673 0.083965 0.106630854782 0.01338 0.0131918957835 0.094 Chr2 157769492

N00010 8600000 8800000 1.75549 scaffold_24 8951829 9140193 0.00441350899503 0.00123041171541 0.00465312558611 0.0013101127421 0.371082166585 0.372322259689 0.127225 0.0733792019707 0.06084 0.0661591386889 0.187 Chr2 157769492

N00010 8800000 9000000 0.9751623 scaffold_24 8756839 8951016 0.00452201323758 0.000979790017902 0.00462114155446 0.0010038936361 0.400071083053 0.408756450995 0.075935 0.06300952224 0.03656 0.0306678957858 0.0865 Chr2 157769492

N00010 9000000 9200000 1.28863 scaffold_24 8563539 8756839 0.00457580290127 0.00124597590304 0.00460569320514 0.0012614058174 0.388945187074 0.391551146221 0.09456 0.075421624418 0.014625 0.0158768753233 NA Chr2 157769492

N00010 9200000 9400000 2.593175 scaffold_24 8360767 8563539 0.0048036420664 0.00148058571966 0.0048036420664 0.00148058571966 0.369680011082 0.368723170334 0.09486 0.109960941353 0 0 NA Chr2 157769492

N00010 9400000 9600000 3.230952 scaffold_24 8160415 8360767 0.00532326061798 0.00164336856194 0.00536547780059 0.0016563307343 0.381922831203 0.38597816579 0.096625 0.112382207315 0.00774 0.00775634882607 NA Chr2 157769492

N00010 9600000 9800000 2.485809 scaffold_24 7968049 8160415 0.00426074824745 0.00130317948106 0.00431843195039 0.00132212532482 0.407240000458 0.409892100235 0.07123 0.0599534221224 0.01731 0.0188390879885 0.05 Chr2 157769492

N00010 9800000 10000000 1.928571 scaffold_24 7777413 7968049 0.00492318514462 0.00139121673631 0.00492919281788 0.00139381723219 0.38693639936 0.386921640112 0.09793 0.080173734237 0.0118 0.00857655427097 0.1075 Chr2 157769492

N00010 10000000 10200000 1.928571 scaffold_24 7582860 7777413 0.0051910170315 0.00123034258023 0.0051910170315 0.00123060602884 0.373793701242 0.374821068293 0.112105 0.107271540403 0.000625 0.000673338370521 0.094 Chr2 157769492

N00010 10200000 10400000 1.097804 scaffold_24 7389033 7582860 0.00464553396895 0.00133790617296 0.00478844638494 0.00140458502781 0.383418873207 0.383454806313 0.067875 0.0451485087217 0.05505 0.0569167350266 0.094 Chr2 157769492

N00010 10400000 10600000 1.080935 scaffold_24 7196988 7389033 0.00452200431366 0.00114160251284 0.0045224844952 0.00114469879042 0.38414712112 0.387386647391 0.082335 0.0490093467677 0.00456 0.00535811919081 0.1155 Chr2 157769492

N00010 10600000 10800000 1.080935 scaffold_24 7000000 7195729 0.00464506260433 0.00135823026936 0.00468551753697 0.00137377177789 0.370184925196 0.367915020668 0.06185 0.0529507635557 0.01031 0.0105962836371 0.083 Chr2 157769492

N00010 10800000 11000000 1.080935 scaffold_24 6792155 7000000 0.00406115450415 0.00111593225206 0.00409885824386 0.00112576840758 0.381167386831 0.380600842445 0.07244 0.104419158508 0.01108 0.0108782987322 0.083 Chr2 157769492

N00010 11000000 11200000 1.080935 scaffold_24 6601535 6792155 0.00427440359701 0.00136436622492 0.00432248107118 0.00139056896145 0.406586881052 0.405264549682 0.06533 0.0393085720281 0.027175 0.0317752596789 0.071 Chr2 157769492

N00010 11200000 11400000 8.503731 scaffold_24 6403263 6601535 0.00445728612493 0.00134085443278 0.0045838291725 0.00139758721465 0.390101284924 0.391307368933 0.096255 0.0600185603615 0.040945 0.0413875887669 0.0525 Chr2 157769492

N00010 11400000 11600000 1.853747 scaffold_24 6215860 6403263 0.00408422091443 0.0013711709874 0.00416863189792 0.00140282305931 0.398797643819 0.39708862984 0.12283 0.0734299877803 0.023455 0.0241991857121 0.091 Chr2 157769492

N00010 11600000 11800000 0.3034282 scaffold_24 6021194 6215860 0.0040955885562 0.00111640934202 0.00412508532686 0.00112533434237 0.403056502404 0.40280100566 0.085675 0.0613871965315 0.011405 0.0112500385275 0.098 Chr2 157769492

N00010 11800000 12000000 0.3034282 scaffold_24 5829403 6021194 0.0047358984486 0.00125625547324 0.00483303399915 0.00129258468479 0.401790093183 0.401400737999 0.0834 0.0735123128823 0.026535 0.0283485669296 0.0525 Chr2 157769492

N00010 12000000 12200000 0.3034282 scaffold_24 5629273 5829403 0.00473633999185 0.0013888654872 0.00480341079123 0.00141141653409 0.381284661362 0.378179469907 0.08549 0.0733673112477 0.017285 0.0185979113576 0.061 Chr2 157769492

N00010 12200000 12400000 0.3034282 scaffold_24 5429282 5629273 0.00462112821017 0.00135985604006 0.00462112821017 0.00135985604006 0.376586218318 0.373248465977 0.126455 0.103489657035 0 0 NA Chr2 157769492

N00010 12400000 12600000 0.3034282 scaffold_24 5231725 5429282 0.00421401851502 0.00127529599653 0.00422016520629 0.00127809181946 0.379866160128 0.377376071562 0.055855 0.056085079243 0.002425 0.00245498767444 0.029 Chr2 157769492

N00010 12600000 12800000 0.4277174 scaffold_24 5035814 5231725 0.00400722382846 0.0012460261852 0.0041348690165 0.00129584647134 0.408091990186 0.411061242715 0.08396 0.0450714865424 0.037965 0.042131375982 0.0495 Chr2 157769492

N00010 12800000 13000000 1.981584 scaffold_24 4845819 5035814 0.00381112164942 0.00110450229864 0.00389751151161 0.00113018336937 0.387546537924 0.385220897712 0.063975 0.0359062080581 0.029565 0.03148503908 0.062 Chr2 157769492

N00010 13000000 13200000 0 scaffold_24 4654604 4845819 0.00383564959749 0.00105311746939 0.00387551903391 0.00109468914662 0.375762244424 0.375301923828 0.08892 0.0518787752007 0.027165 0.046094710143 0.044 Chr2 157769492

N00010 13200000 13400000 0.54371 scaffold_24 4467546 4654604 0.00314980453122 0.000935759778812 0.00315267575218 0.000937037739429 0.407422859245 0.409939365746 0.052995 0.0198227287793 0.001125 0.00179623432304 0.147 Chr2 157769492

N00010 13400000 13600000 1.517857 scaffold_24 4275938 4467546 0.00443631902108 0.00158319306379 0.00450357665385 0.00161508817093 0.385621767031 0.38704888811 0.121565 0.0859619640099 0.02288 0.0242056699094 0.086 Chr2 157769492

N00010 13600000 13800000 1.517857 scaffold_24 4076061 4275938 0.00454924881473 0.00111823299653 0.00454924881473 0.00111823299653 0.368287103231 0.368499273851 0.115175 0.12291559309 0 0 NA Chr2 157769492

N00010 13800000 14000000 1.436297 scaffold_24 3873289 4076061 0.00448407869253 0.00127172542246 0.00448407869253 0.00127172542246 0.364697538687 0.363577105609 0.1255 0.142983252126 0 0 NA Chr2 157769492

N00010 14200000 14400000 1.288435 scaffold_24 3467755 3675556 0.00357964435856 0.00116798460946 0.00358398517934 0.00116823988377 0.360713636177 0.360056332455 0.13829 0.139522908937 0.002525 0.00243020967175 0.084 Chr2 157769492

N00010 14800000 15000000 0.1791082 scaffold_24 2882466 3076352 0.00406822582711 0.00139305659942 0.00406822582711 0.00139305659942 0.346110589894 0.343820440538 0.114895 0.1138916683 0 0 0.105 Chr2 157769492

N00095 0 200000 0.8653143 scaffold_38 4479209 4687157 0.00281158108928 0.00177084579065 0.00281158108928 0.00177084579065 0.353347605534 0.348550107101 0.131525 0.117231230885 0 0 NA Chr2 157769492

N00095 200000 400000 0.8653143 scaffold_38 4282991 4479209 0.0023894323655 0.0011724138544 0.00241418597273 0.00118810536907 0.380284861791 0.376939261392 0.083565 0.0650042299891 0.01283 0.0144380230152 0.034 Chr2 157769492

N00095 400000 600000 0.8653143 scaffold_38 4082920 4282991 0.0034560569739 0.00120802836132 0.00347567128712 0.00121745632492 0.365743432528 0.361535394349 0.052485 0.0456937787086 0.00703 0.0086919143704 0.035 Chr2 157769492

N00095 600000 800000 0.8653143 scaffold_38 3884423 4082920 0.00393060686821 0.00126247488321 0.00401397376512 0.00129020552333 0.369220144141 0.366552487967 0.07539 0.0579101951163 0.02397 0.026176718036 0.046 Chr2 157769492

N00095 800000 1000000 0.8653143 scaffold_38 3684474 3884423 0.00473803636748 0.00124698277561 0.00475386318124 0.00125447003272 0.38245484133 0.383352050971 0.09003 0.0829411499932 0.010615 0.0106527164427 0.08 Chr2 157769492

N00095 1000000 1200000 0.8653143 scaffold_38 3492623 3684474 0.00402271872157 0.000969409884916 0.00409687636824 0.00098871869324 0.384900923838 0.382969510892 0.08846 0.0759599897837 0.02235 0.0226582087141 0.0306666666667 Chr2 157769492

N00095 1200000 1400000 0.8653143 scaffold_38 3293344 3492623 0.00496501096683 0.000917565892577 0.0049766763326 0.00093473819791 0.379204040756 0.379095467331 0.084945 0.0747444537558 0.006285 0.0313881542962 0.16 Chr2 157769492

N00095 1400000 1600000 0.8653143 scaffold_38 3094641 3293344 0.00435758477728 0.000812150810732 0.0043420304295 0.000812488972821 0.365180336314 0.366377761811 0.101805 0.0802202281797 0.007125 0.021529619583 0.16 Chr2 157769492

N00095 1600000 1800000 0.8653143 scaffold_38 2897814 3094104 0.00408298027436 0.000522763457913 0.00408962144642 0.000524202733385 0.389325085707 0.390042261105 0.071985 0.0942432115747 0.005725 0.00577716643741 NA Chr2 157769492

N00095 1800000 2000000 0.8653143 scaffold_38 2704080 2897814 0.00442434825197 0.000600188123174 0.00452016781365 0.000617780657765 0.39704492224 0.392636598587 0.097215 0.101185130127 0.028255 0.0349086892337 0.0843333333333 Chr2 157769492

N00095 2000000 2200000 0.8653143 scaffold_38 2498909 2704080 0.00383699796072 0.000460934031716 0.00390844270697 0.000471347327853 0.392036115423 0.393029539625 0.09105 0.0648288500812 0.03363 0.034654020305 0.0985 Chr2 157769492

N00095 2200000 2400000 3.691867 scaffold_38 2307764 2498909 0.00484126199873 0.00111380489059 0.00485172821049 0.00111743840704 0.384385263786 0.386242694597 0.076615 0.0748646315624 0.004 0.00356274032802 0.104 Chr2 157769492

N00095 2600000 2800000 3.734694 scaffold_38 1927096 2130198 0.00473440943751 0.000418520804923 0.00474472503883 0.00041983000586 0.398433611717 0.396964903985 0.073855 0.0867347441187 0.005545 0.00548000512058 0.071 Chr2 157769492

N00095 2800000 3000000 3.734694 scaffold_38 1727457 1927096 0.00456134293525 0.000372261867224 0.00466921561503 0.000384524634224 0.387856874259 0.385191399278 0.0812 0.0859150767135 0.030705 0.0300843021654 0.07 Chr2 157769492

N00095 3000000 3200000 3.734694 scaffold_38 1540549 1727457 0.0036466491694 0.000374340928887 0.00366977018761 0.000376812085738 0.377190094103 0.374172038231 0.092405 0.0659308322811 0.007365 0.00789693325058 0.038 Chr2 157769492

N00070 200000 400000 2.131896 scaffold_5 33378248 33568880 0.00394206474566 0.00120840217138 0.00394313832719 0.00120840217138 0.385512738818 0.386507607765 0.094075 0.059119140543 6,00E-04 0 NA Chr2 157769492

N00070 400000 600000 1.472103 scaffold_5 33568880 33762274 0.00471034015617 0.00119038330825 0.00472146751487 0.00119457828904 0.365149052399 0.368288821772 0.07 0.0677373651716 0.004555 0.00653070932914 NA Chr2 157769492

N00070 600000 800000 1.472103 scaffold_5 33762274 33953863 0.00466979375814 0.00120796860312 0.00475552586397 0.00123030237848 0.362184133316 0.361765287948 0.083665 0.0619607597513 0.02284 0.0234721200069 0.071 Chr2 157769492

N00070 800000 1000000 1.472103 scaffold_5 33953863 34149529 0.00505017206353 0.00120675207931 0.00509472959293 0.0012193708799 0.38142142233 0.381378259602 0.079795 0.0822166344689 0.010325 0.0113560863921 0.061 Chr2 157769492

N00070 1000000 1200000 1.742448 scaffold_5 34149529 34340250 0.00497639035076 0.00120861958453 0.00515681848669 0.00126792560312 0.364906334171 0.364595944955 0.05394 0.0557044059123 0.04958 0.0532348299348 0.0426666666667 Chr2 157769492

N00070 1200000 1400000 3.834682 scaffold_5 34340250 34530472 0.00395297088998 0.00104499782131 0.00410944889262 0.00110316573727 0.383583021223 0.390332738039 0.09626 0.0610286927905 0.052205 0.0568230804008 0.05625 Chr2 157769492

N00070 1400000 1600000 3.834682 scaffold_5 34530472 34716372 0.003967449432 0.00102027969584 0.00400515690695 0.00103637509612 0.421362454123 0.425093408631 0.102665 0.0611188811189 0.015455 0.0190371167294 0.03925 Chr2 157769492

N00070 1600000 1800000 3.834682 scaffold_5 34716372 34918431 0.00374789991615 0.000967480101389 0.00380451510753 0.000980570166633 0.380695748852 0.378843144441 0.11778 0.107498304951 0.01817 0.0168218193696 0.04725 Chr2 157769492

N00070 1800000 2000000 3.834682 scaffold_5 34918431 35106994 0.00406875757322 0.00120041037809 0.00407405657584 0.00120313472633 0.390754737524 0.390325850184 0.098605 0.0724850580443 0.00214 0.00203645465972 0.035 Chr2 157769492

N00070 2000000 2200000 7.133477 scaffold_5 35106994 35297789 0.00358424563597 0.000956767445518 0.00359649923338 0.000960495045562 0.387669155723 0.387341002102 0.092895 0.0735553866716 0.002885 0.00415629340392 0.008 Chr2 157769492

N00070 2400000 2600000 0 scaffold_5 35486038 35679385 0.00466763628331 0.000918919313109 0.00466763628331 0.000918919313109 0.37961794969 0.380362338732 0.082235 0.0856698060999 0 0 NA Chr2 157769492

N00070 2600000 2800000 0 scaffold_5 35679385 35875875 0.00462385283173 0.00102072554106 0.00465734649734 0.00102876470379 0.380956982402 0.384468988765 0.090815 0.0692554328465 0.00879 0.00813781871851 0.041 Chr2 157769492

N00070 3000000 3200000 0 scaffold_38 126395 332530 0.00522650318395 0.000821801980217 0.00522966097404 0.000821386183274 0.375381766715 0.37484118262 0.06429 0.0668590971936 0.001225 0.00117883911029 0.142 Chr2 157769492

N00070 3200000 3400000 1.368542 scaffold_38 332530 534024 0.00536626188684 0.00102175210583 0.00540905047231 0.00102929448696 0.366299650607 0.363094465299 0.103325 0.0930995463885 0.0109 0.010744736816 0.066 Chr2 157769492

N00070 3400000 3600000 1.719231 scaffold_38 534024 722538 0.00442410876813 0.000777994194995 0.00444154022227 0.000781957380964 0.406319290466 0.406008389072 0.112135 0.0743764388852 0.00571 0.00610034268012 0.073 Chr2 157769492

N00070 3600000 3800000 1.719231 scaffold_38 722538 913473 0.00328048036468 0.000565306747302 0.00341645763381 0.000589767352183 0.41256100026 0.417020418678 0.069835 0.0430931992563 0.036765 0.0385262000157 0.029 Chr2 157769492

N00070 3800000 4000000 1.719231 scaffold_38 913473 1102477 0.00411147733514 0.000562864673077 0.00414382357834 0.000569584804871 0.384817018444 0.390407486612 0.12802 0.0670779454403 0.013175 0.0133647965122 0.0366666666667 Chr2 157769492

N00070 4000000 4200000 1.719231 scaffold_38 1102608 1291852 0.004586894995 0.000533415710514 0.0045993264354 0.000534879070937 0.373202868346 0.371219106052 0.10175 0.0945657458096 0.002265 0.00240958762233 0.039 Chr2 157769492

N00045 0 200000 3.167042 scaffold_5 33001125 33193145 0.00418957231787 0.00111575780619 0.00425037456453 0.00113602439909 0.391812170883 0.39341467546 0.058825 0.0375117175294 0.026585 0.0242943443391 0.07 Chr2 157769492

N00045 200000 400000 1.814662 scaffold_5 32803619 33001125 0.00400306907646 0.00121190723046 0.00403749629772 0.00123033838969 0.37986885094 0.379799789132 0.102165 0.0816481524612 0.015655 0.0166577217907 0.029 Chr2 157769492

N00045 400000 600000 1.814662 scaffold_5 32604870 32803589 0.00405480097865 0.00108170187679 0.00408847351402 0.00108969615181 0.385042984726 0.386788222986 0.086455 0.0776674600818 0.009795 0.0095562075091 0.043 Chr2 157769492

N00045 600000 800000 1.814662 scaffold_5 32413300 32604870 0.00422365597353 0.00122596916368 0.00422365597353 0.00122625836863 0.386248811392 0.387156281876 0.07782 0.062050425432 0 0.0001722607924 NA Chr2 157769492

N00045 800000 1000000 1.814662 scaffold_5 32206638 32413300 0.00487941257652 0.00160940291563 0.00495311489545 0.00163316224455 0.380914411646 0.380688219028 0.1029 0.103628146442 0.018405 0.0162003658147 0.084 Chr2 157769492

N00045 1000000 1200000 1.814662 scaffold_5 32006437 32206638 0.00432988495824 0.00122318320363 0.00437941031599 0.00123287144316 0.387345382298 0.390355090279 0.064805 0.0463334348979 0.01369 0.0136962352835 NA Chr2 157769492

N00045 1200000 1400000 1.814662 scaffold_5 31803411 32006437 0.00404778522031 0.00121652171792 0.00411792317454 0.00124228782216 0.390603752308 0.392217651937 0.067745 0.0594800665925 0.034075 0.0330253268054 0.153 Chr2 157769492

N00045 1400000 1600000 1.814662 scaffold_5 31605813 31803411 0.00356658684977 0.000994495487046 0.00359631449293 0.00100547418604 0.377412202192 0.375924723453 0.12727 0.107339143109 0.010255 0.0105466654521 0.037 Chr2 157769492

N00045 1600000 1800000 1.814662 scaffold_5 31405310 31605813 0.00424864237372 0.00119810032784 0.00430383325198 0.00121430423349 0.382076918359 0.38268793985 0.093195 0.0681286564291 0.013015 0.0144037745071 0.111 Chr2 157769492

N00045 1800000 2000000 1.814662 scaffold_5 31203681 31405273 0.00483459611257 0.00117265688007 0.00486757726061 0.00118202049531 0.373514431239 0.375498909658 0.113155 0.0981735386325 0.008675 0.0079417834041 0.0855 Chr2 157769492

N00045 2000000 2200000 2.06523 scaffold_5 31007512 31203635 0.00468713499656 0.00121270268214 0.00473811623969 0.00122774736807 0.367443791554 0.368885784698 0.10789 0.0983566435349 0.01156 0.0119465845413 0.0465 Chr2 157769492

N00045 2200000 2400000 1.454452 scaffold_5 30811051 31007512 0.00437056244289 0.00117915400821 0.00438575443601 0.00118264638873 0.36961714085 0.369223031287 0.121195 0.118094685459 0.003665 0.00357831834308 0.076 Chr2 157769492

N00045 2400000 2600000 1.454452 scaffold_5 30620940 30811051 0.00401417661548 0.00107977311484 0.00402027620052 0.00108338455778 0.357695051636 0.358660542472 0.117775 0.0902367564212 0.00314 0.00332437365539 0.076 Chr2 157769492

N00045 2600000 2800000 1.454452 scaffold_5 30432307 30620273 0.00387043422426 0.00119966353302 0.00387043422426 0.00119966353302 0.352923899492 0.355282245348 0.12472 0.109918815105 0 0 NA Chr2 157769492

N00045 2800000 3000000 1.454452 scaffold_5 30248334 30432307 0.00304757916993 0.00103726665538 0.00304884769845 0.00103726665538 0.362744292505 0.364625687635 0.119735 0.0599055296158 0.00568 0 NA Chr2 157769492

N00045 3000000 3200000 0.7742607 scaffold_5 30063917 30248334 0.00277166036986 0.000852989417513 0.00286985946547 0.000893765423484 0.366178147569 0.369238021673 0.062585 0.0382231573011 0.058105 0.0633943725362 0.0585 Chr2 157769492

N00045 3600000 3800000 1.805139 scaffold_5 29461986 29659724 0.00355063463046 0.000968959235554 0.00355063463046 0.000968959235554 0.374264684491 0.375127206572 0.120225 0.113513841548 0 0 NA Chr2 157769492

N00045 3800000 4000000 2.481935 scaffold_5 29262128 29461986 0.00434350058985 0.00112525763121 0.00435839069162 0.00112884802139 0.367263320446 0.375809318965 0.08015 0.0970439011698 0.00402 0.00462828608312 NA Chr2 157769492

N00045 4000000 4200000 2.378059 scaffold_5 29062921 29262128 0.00450315625935 0.00129737852362 0.00452018923469 0.00129893270228 0.377165969573 0.380861040068 0.099725 0.105071608929 0.00465 0.00510022238174 0.133 Chr2 157769492

N00045 4400000 4600000 2.58083 scaffold_5 28672963 28872775 0.00423460340689 0.0011893802086 0.00424852637826 0.00119174113414 0.414053432729 0.411799608172 0.10027 0.0929123375973 0.006945 0.00710167557504 0.018 Chr2 157769492

N00045 4600000 4800000 2.435611 scaffold_5 28476341 28672963 0.00384038361683 0.00112238902092 0.00388653381766 0.00113715641138 0.402923235018 0.398545302493 0.073835 0.0438302936599 0.0174 0.0130504216212 0.095 Chr2 157769492

N00045 4800000 5000000 1.034005 scaffold_5 28282595 28476341 0.00401139317163 0.00131488365474 0.00403117408121 0.00132625318181 0.411568502564 0.408588075398 0.0866 0.0512939621979 0.005385 0.00775241811444 0.046 Chr2 157769492

N00045 5000000 5200000 1.034005 scaffold_5 28084456 28282595 0.00459100663046 0.00116805275276 0.00464920316829 0.00118660095571 0.39609616049 0.395895756393 0.12028 0.112522017372 0.018555 0.0186687123686 0.0596 Chr2 157769492

N00045 5800000 6000000 2.087146 scaffold_5 27298465 27512168 0.00528803722577 0.00132245083387 0.00528803722577 0.00132245083387 0.349349688885 0.345697921435 0.14759 0.153713331118 0 0 NA Chr2 157769492

N00045 6000000 6200000 2.087146 scaffold_5 27100647 27298465 0.0046571692426 0.00124606495315 0.0046571692426 0.00124606495315 0.357615822329 0.35741028962 0.08121 0.0616273544369 0 0 NA Chr2 157769492

N00045 6200000 6400000 1.929231 scaffold_5 26900232 27100647 0.00466410966559 0.0012896925687 0.00468615236208 0.0012960710899 0.362408481293 0.359878893779 0.084535 0.0968590175386 0.005175 0.00510440835267 NA Chr2 157769492

N00024 200000 400000 0.9115578 scaffold_5 26523187 26707363 0.00484762157384 0.00134915116944 0.00489408202034 0.00136271182492 0.387671473795 0.390886937642 0.104365 0.0523520979932 0.01124 0.010652853792 0.137666666667 Chr2 157769492

N00024 400000 600000 0.9115578 scaffold_5 26325216 26523185 0.00444462673497 0.00136248180423 0.004543904561 0.00139716702008 0.365187060739 0.364320630571 0.087765 0.0853517469907 0.0235 0.0241856048169 0.044 Chr2 157769492

N00024 600000 800000 3.349054 scaffold_5 26122586 26325216 0.00487700849111 0.00141300610057 0.00494728253394 0.00143654779642 0.360353793913 0.356808686013 0.101615 0.124532398954 0.01882 0.0194689828752 0.1095 Chr2 157769492

N00024 1000000 1200000 0.971841 scaffold_5 25723947 25919005 0.00474266406383 0.00121101599289 0.00477838728816 0.00121986739227 0.357462587431 0.358269495853 0.10779 0.103046273416 0.007115 0.00731064606425 0.051 Chr2 157769492

N00024 1200000 1400000 0 scaffold_5 25522332 25723947 0.00461645210926 0.00126206385003 0.00462355901344 0.00126358092003 0.356355752252 0.354933539242 0.089645 0.101505344344 0.002015 0.00200381916028 0.051 Chr2 157769492

N00024 1600000 1800000 0.9259552 scaffold_5 25119365 25321236 0.00451430558082 0.00104298879069 0.00458531623775 0.00105934486309 0.377548911738 0.376777774631 0.093055 0.0853713510113 0.023855 0.0244017218917 0.069 Chr2 157769492

N00024 1800000 2000000 2.139276 scaffold_5 24920797 25119365 0.00416706104189 0.00134600570778 0.00421555975177 0.00136864626957 0.384297747867 0.382047760107 0.07366 0.0597427581483 0.01992 0.0199427903791 0.069 Chr2 157769492

N00024 2200000 2400000 2.420444 scaffold_5 24513420 24720629 0.00467233872234 0.0012348611353 0.00472326043329 0.00125442706661 0.378582049202 0.382656986114 0.09274 0.0992041851464 0.01644 0.0161286430609 0.013 Chr2 157769492

N00024 2400000 2600000 3.309587 scaffold_5 24316023 24513420 0.00380917098913 0.000985384898054 0.00383529987062 0.000992698158272 0.39144432194 0.394246035016 0.0804 0.0698136243205 0.00912 0.00898189942096 0.006 Chr2 157769492

N00024 2600000 2800000 3.309587 scaffold_5 24128719 24315679 0.00377753969299 0.000965636069166 0.00377953175102 0.000966243327697 0.3916759839 0.400326690267 0.08432 0.0311136071887 0.000435 0.000508130081301 0.006 Chr2 157769492

N00024 2800000 3000000 3.309587 scaffold_5 23931747 24128719 0.00379360408126 0.0010219895635 0.00379677910689 0.0010226649852 0.390542878859 0.393386411267 0.05796 0.0540685985825 0.002895 0.0005787624637 0.015 Chr2 157769492

N00024 3000000 3200000 3.309587 scaffold_5 23738615 23931747 0.00420915686657 0.00107880542107 0.00421273123465 0.00107988240659 0.391958288472 0.394266012465 0.056575 0.0493030673322 0.000735 0.00076113746039 0.015 Chr2 157769492

N00024 3400000 3600000 3.002985 scaffold_5 23359552 23552129 0.00422645224534 0.00116799089319 0.00433198842343 0.00120705809009 0.411051367778 0.413144870684 0.08839 0.0567149763471 0.03469 0.0362192785224 0.11375 Chr2 157769492

N00024 3600000 3800000 1.882468 scaffold_5 23170579 23359552 0.00405375045289 0.00114972906744 0.00424061883216 0.0012201891239 0.394027987439 0.396294368052 0.127655 0.0810644906944 0.05653 0.0635328856503 0.0576 Chr2 157769492

N00024 4600000 4800000 2.43554 scaffold_5 22228076 22421011 0.00419136821133 0.00112712803176 0.00421683378612 0.00113460392995 0.402601204887 0.402885595983 0.09069 0.0607406639542 0.007465 0.00814782180527 NA Chr2 157769492

N00024 4800000 5000000 2.220368 scaffold_5 22029727 22228076 0.00442535938741 0.00111153896856 0.0044292904766 0.00111405720876 0.400735273451 0.39749115148 0.098265 0.0921254959692 0.002815 0.00285859772421 NA Chr2 157769492

N00024 5000000 5200000 0.3787313 scaffold_5 21833482 22029727 0.00448597277168 0.00129058791349 0.00456969309867 0.00131665704034 0.385563339576 0.387629974692 0.11054 0.0759713623277 0.02594 0.0267420826008 0.072 Chr2 157769492

N00024 5200000 5400000 0 scaffold_5 21633807 21833482 0.00424982215058 0.000921322113077 0.004304982197 0.000942396539783 0.410185232491 0.413701965072 0.087845 0.0737698760486 0.024985 0.0267835232252 0.0695 Chr2 157769492

N00024 5400000 5600000 2.458333 scaffold_5 21442280 21633807 0.00438812869098 0.00108992556598 0.00445001256332 0.00110505498253 0.382306173758 0.391367134629 0.097995 0.070402606421 0.01935 0.0151780166765 0.042 Chr2 157769492

N00024 5600000 5800000 4.1875 scaffold_5 21251121 21442280 0.00461221224816 0.00105416515346 0.00465123327834 0.00106731809802 0.364127359704 0.362181686437 0.06628 0.0648779288446 0.01487 0.0147887360783 NA Chr2 157769492

N00024 5800000 6000000 0.0625 scaffold_5 21056597 21251121 0.00444843858429 0.000866798352251 0.00449753837897 0.000877096488144 0.351112858111 0.351389815433 0.08359 0.0801803376447 0.016995 0.0174168740104 NA Chr2 157769492

N00024 6000000 6200000 4.778912 scaffold_5 20859870 21056597 0.00359301100785 0.000752534981175 0.00366123781254 0.00077650655448 0.372711994759 0.376513740436 0.06842 0.044808287627 0.05852 0.0606271635312 0.111666666667 Chr2 157769492

N00024 6200000 6400000 4.96615 scaffold_5 20660913 20858918 0.00412085110059 0.00100165534082 0.00421894593558 0.00102548000154 0.390182116938 0.395210150852 0.074645 0.0604479684856 0.03235 0.0313476932401 0.067 Chr2 157769492

N00024 6400000 6600000 2.780876 scaffold_5 20464054 20660913 0.00369337588546 0.00102413205434 0.00375563621655 0.00104070939616 0.423013184165 0.426448537475 0.09283 0.0601699693689 0.01341 0.0135630070253 0.0115 Chr2 157769492

N00024 6600000 6800000 2.780876 scaffold_5 20261014 20464054 0.0036013041751 0.00100267195421 0.00363518535406 0.00101376970584 0.426843352328 0.43017689346 0.057805 0.0560332939322 0.01144 0.0119533096927 0.112 Chr2 157769492

N00024 7000000 7200000 2.0864 scaffold_5 19872553 20064929 0.00349806551623 0.00107097009818 0.00367685807891 0.00113514632802 0.405116425931 0.408509834255 0.09579 0.0681685865181 0.057415 0.0657306524722 0.0715 Chr2 157769492

N00024 7200000 7400000 2.0864 scaffold_5 19679095 19872553 0.00448061530144 0.00107479266759 0.00458903902539 0.00110560748308 0.397969216576 0.398895418246 0.107365 0.0921698766657 0.03083 0.030916271232 0.0738 Chr2 157769492

N00024 7400000 7600000 0.9653493 scaffold_5 19485084 19679095 0.00449924196612 0.00128132803925 0.00452270483294 0.00128701047905 0.353322769423 0.354301762972 0.095445 0.0762637170057 0.00535 0.00525227950992 0.0415 Chr2 157769492

N00024 7600000 7800000 0 scaffold_5 19301769 19485084 0.00462605355375 0.00132469331227 0.0046863988011 0.0013422535284 0.345387299857 0.343769220631 0.07551 0.0425115238797 0.013975 0.0164743747102 0.055 Chr2 157769492

N00024 7800000 8000000 0 scaffold_5 19103387 19301769 0.00442195878575 0.00113500607323 0.0044876464156 0.00115380807641 0.379056227352 0.37771254909 0.10987 0.107676099646 0.01944 0.0213729068161 0.0613333333333 Chr2 157769492

N00024 8000000 8200000 0 scaffold_5 18909733 19103387 0.00435673578374 0.000979623006896 0.00440732871895 0.000993421105222 0.376863886567 0.376903759138 0.08746 0.0819089716711 0.0142 0.0149803257356 0.0435 Chr2 157769492

N00024 8200000 8400000 0 scaffold_5 18716844 18909733 0.00428162271068 0.000961747695135 0.00428162271068 0.000962773094861 0.363156433916 0.36479915024 0.09445 0.0798801383179 0 0.00103168143336 0.04 Chr2 157769492

N00024 8400000 8600000 2.080507 scaffold_5 18525477 18716472 0.00376354411358 0.0009623221688 0.00376546990028 0.00096386259691 0.367706464431 0.369325238768 0.065585 0.0469122228331 0.000455 0.00174350113877 0.04 Chr2 157769492

N00024 8800000 9000000 2.132171 scaffold_5 18111602 18313230 0.00541195746843 0.00114195971123 0.00541679644656 0.00114497062436 0.384470011511 0.387459891031 0.13716 0.123876644117 0.0011 0.00212768067927 0.054 Chr2 157769492

N00024 9000000 9200000 2.256781 scaffold_5 17914699 18111602 0.00516557668105 0.00131388901316 0.0052234062661 0.00133516470354 0.370530865054 0.371979122584 0.09256 0.0947217665551 0.018115 0.0188722365835 0.076 Chr2 157769492

N00024 9200000 9400000 2.256781 scaffold_5 17716583 17914699 0.00496810746208 0.00122568482613 0.00498820495024 0.00123089817209 0.363223254025 0.361477144202 0.06567 0.0695148296957 0.00453 0.00605705748148 0.053 Chr2 157769492

N00024 9600000 9800000 2.256781 scaffold_5 17314438 17525388 0.00472681380427 0.00119620386008 0.00475529478754 0.00120448164672 0.380139694988 0.381393027405 0.11898 0.129926522873 0.00946 0.0105807063285 NA Chr2 157769492

N00024 10000000 10200000 1.116114 scaffold_5 16939251 17136137 0.00512034444046 0.000965574320096 0.00522916296893 0.000983768842426 0.388004442799 0.389919418365 0.108065 0.101510518777 0.028355 0.0287831537032 0.0626 Chr2 157769492

N00014 2400000 2600000 1.100029 scaffold_5 14206251 14395799 0.00334728690809 0.000731102045783 0.00337820320332 0.000740033149621 0.380349026553 0.383272158634 0.06536 0.0230653976829 0.0135 0.0142444130247 0.032 Chr2 157769492

N00014 2600000 2800000 1.100029 scaffold_5 14018046 14206251 0.00346688217532 0.000620382037277 0.00346688217532 0.000620382037277 0.378239839113 0.383270751866 0.09962 0.068345686884 0 0 0.032 Chr2 157769492

N00014 2800000 3000000 1.100029 scaffold_5 13817574 14018046 0.00343147153137 0.000648966329335 0.00343147153137 0.000647990270474 0.399549771526 0.405291734421 0.092585 0.0849744602738 0 0.00188555010176 NA Chr2 157769492

N00014 3200000 3400000 1.100029 scaffold_5 13412350 13606259 0.004218592126 0.000967207969381 0.00423817667763 0.000969537420469 0.402042997981 0.406408786847 0.11694 0.112836433585 0.006295 0.00683825918343 0.07 Chr2 157769492

N00014 3400000 3600000 1.502489 scaffold_5 13217599 13412350 0.00379003785782 0.000870511431884 0.00381513104764 0.000877111750138 0.419522378653 0.41971258209 0.081835 0.0783461959117 0.008295 0.00905771985766 0.07 Chr2 157769492

N00014 3600000 3800000 1.939453 scaffold_5 13020631 13217599 0.00360664365626 0.000831742941507 0.00360738745441 0.000832818766716 0.420651289312 0.421704428284 0.0825 0.0751289549572 0.000875 0.00349295317006 NA Chr2 157769492

N00014 4200000 4400000 2.077699 scaffold_5 12432902 12640677 0.00416585188668 0.000909317670632 0.00428909269799 0.000936763713514 0.387652496207 0.383944357341 0.064115 0.102851642402 0.036785 0.0364143905667 0.198 Chr2 157769492

N00014 4400000 4600000 2.166008 scaffold_5 12239922 12432902 0.00464327236281 0.000932885111394 0.00473297585249 0.000949160909698 0.388275456151 0.390966499589 0.08737 0.0808322105918 0.029805 0.027531350399 0.1135 Chr2 157769492

N00014 4600000 4800000 1.357796 scaffold_5 12050273 12239922 0.00431506435649 0.000905739121621 0.00439188485886 0.000928931015461 0.392449060418 0.395707627473 0.074585 0.0721754398916 0.02632 0.0296284188158 0.084 Chr2 157769492

N00014 4800000 5000000 0 scaffold_5 11841345 12050273 0.00475662722402 0.00103033385655 0.00475662722402 0.00103033385655 0.404199398925 0.408951987729 0.12421 0.133787716342 0.008465 0 NA Chr2 157769492

N00014 5000000 5200000 0 scaffold_5 11649948 11841345 0.00410411432257 0.000713359788437 0.00413546362929 0.000719279396416 0.410199048104 0.419129423638 0.06266 0.0522996703188 0.008295 0.00868352168529 NA Chr2 157769492

N00014 5200000 5400000 1.677503 scaffold_5 11461642 11649948 0.00449300404025 0.000816508447292 0.00449300404025 0.000816508447292 0.394013759173 0.401020443302 0.128735 0.0975433602753 0 0 NA Chr2 157769492

N00014 5400000 5600000 2.232967 scaffold_5 11266745 11461642 0.00442630704927 0.000932397086502 0.00442630704927 0.000932397086502 0.411608533999 0.417508193449 0.098365 0.0760760812121 0 0 NA Chr2 157769492

N00014 5600000 5800000 3.914791 scaffold_5 11076342 11266745 0.00374388548909 0.000797655833423 0.00388298373816 0.000840554251425 0.421182625624 0.431001466402 0.072905 0.054941361218 0.057805 0.0595526330993 0.0736 Chr2 157769492

N00014 5800000 6000000 6.38479 scaffold_5 10877555 11075442 0.00395075750206 0.000953617020621 0.00408349800998 0.00100612664746 0.419404370395 0.420848505377 0.06237 0.0532728274217 0.051905 0.0536316180446 0.053 Chr2 157769492

N00014 6000000 6200000 0 scaffold_5 10671752 10877555 0.0047157546008 0.000918966589548 0.00480184142942 0.000939429141394 0.412366833888 0.41127032085 0.085195 0.101052948694 0.027765 0.027273654903 NA Chr2 157769492

N00014 6200000 6400000 0.6729041 scaffold_5 10475031 10671746 0.00360931230896 0.000882897361745 0.00368070550357 0.000902419813926 0.408535445383 0.407117772309 0.06595 0.0586279643139 0.02986 0.0307551533945 0.04275 Chr2 157769492

N00014 6400000 6600000 1.176119 scaffold_5 10273643 10475031 0.00391492731894 0.000856480779022 0.00400250726949 0.000883305750425 0.400876806831 0.403040475678 0.109505 0.123706477049 0.02824 0.0277871571295 0.075 Chr2 157769492

N00014 6600000 6800000 0.3455199 scaffold_5 10072133 10273643 0.00338114349648 0.000773881039332 0.00338663185121 0.000777390713724 0.40995242353 0.409438410187 0.08551 0.0770185102476 0.007085 0.00762245049873 0.091 Chr2 157769492

N00014 7000000 7200000 2.046891 scaffold_5 9649190 9842992 0.00463558518448 0.000887911693433 0.00463577040819 0.000888182330325 0.403767872362 0.406825710813 0.149475 0.116825419758 0.00447 0.00458199605783 0.079 Chr2 157769492

N00014 7400000 7600000 2.914783 scaffold_5 9266728 9463901 0.00397858758048 0.000895775567705 0.00397858758048 0.000895775567705 0.408123180124 0.411900522978 0.10075 0.115005604216 0 0 NA Chr2 157769492

N00014 7600000 7800000 1.455707 scaffold_5 9078012 9266704 0.00361572311017 0.000806960397532 0.00362944460608 0.000805461108623 0.455446246566 0.459212244595 0.116685 0.0734636338584 0.006535 0.0030207957942 0.032 Chr2 157769492

N00014 7800000 8000000 3.661765 scaffold_5 8883063 9078012 0.00360059130597 0.000931760975911 0.00360059130597 0.000931760975911 0.410752819398 0.416415732105 0.078435 0.0685153552981 0 0 NA Chr2 157769492

N00014 8000000 8200000 3.833958 scaffold_5 8690970 8883063 0.00409933819825 0.000718391550435 0.00409933819825 0.000718391550435 0.384952552842 0.384666378125 0.079795 0.0712363282368 0 0 NA Chr2 157769492

N00014 8200000 8400000 0.8954136 scaffold_5 8490995 8690970 0.0040756394137 0.00101470842126 0.00409838694224 0.00102026082359 0.410323166746 0.41319195115 0.0682 0.0822702837855 0.005245 0.00529066133267 0.037 Chr2 157769492

N00014 8400000 8600000 1.570062 scaffold_5 8300588 8490995 0.00435510275056 0.0009662875975 0.00442744496302 0.000981286499084 0.384371131184 0.384797682788 0.071285 0.0582804203627 0.016005 0.0169794177735 0.031 Chr2 157769492

N00014 8600000 8800000 2.843085 scaffold_5 8108124 8300588 0.00389114698878 0.00098587370453 0.00389277019493 0.000985991643226 0.414690968097 0.417244995872 0.102725 0.0915755673788 0.00094 0.000654667885942 0.031 Chr2 157769492

N00014 9200000 9400000 1.796458 scaffold_5 7522733 7711106 0.00459926607482 0.000931075372522 0.0046599608612 0.00095067014749 0.381756289431 0.382010526985 0.11014 0.0914727694521 0.02485 0.0220519925892 0.051 Chr2 157769492

N00014 9400000 9600000 1.287841 scaffold_5 7320166 7522733 0.00431728495607 0.00115385762594 0.00434655600679 0.00116809485147 0.404922120508 0.411090581145 0.08031 0.0990882029156 0.010395 0.0179249334788 0.0475 Chr2 157769492

N00014 9600000 9800000 1.445992 scaffold_5 7119755 7320166 0.00377883145181 0.000729529341552 0.00385787756253 0.000749800328959 0.391415530458 0.389527414346 0.105635 0.103452405307 0.02719 0.0249187918827 0.068 Chr2 157769492

N00014 9800000 10000000 3.352518 scaffold_5 6924863 7119755 0.00460404995822 0.00094679487212 0.00470650405244 0.000973062016009 0.40148685869 0.402212283908 0.073135 0.0736510477598 0.045805 0.0470003899596 0.105 Chr2 157769492

N00014 10000000 10200000 1.750451 scaffold_5 6737207 6924863 0.00430283861499 0.00103972819566 0.004337015769 0.00104890521139 0.426230093339 0.437315453107 0.094465 0.0782389052308 0.017 0.0176812891674 0.166666666667 Chr2 157769492

N00014 10200000 10400000 1.176737 scaffold_5 6547707 6737207 0.00408653607392 0.00125287703249 0.00408653607392 0.00125287703249 0.422469945095 0.431030340472 0.11794 0.106897097625 0 0 NA Chr2 157769492

N00014 10600000 10800000 1.12554 scaffold_5 6157744 6351528 0.00395004923365 0.00108094569315 0.00400378476557 0.00109436951121 0.385934428004 0.387729018229 0.1168 0.113002105437 0.018475 0.019769434009 0.087 Chr2 157769492

N00014 11200000 11400000 3.292754 scaffold_5 5574254 5769877 0.00414678924284 0.00109478976975 0.00415052233703 0.00109745359013 0.377037011059 0.377854604163 0.12017 0.119852982523 0.002115 0.00275018786135 0.103 Chr2 157769492

N00014 13200000 13400000 2.235687 scaffold_5 3592858 3790933 0.00349765938265 0.000976599181264 0.00350243046874 0.000975868384634 0.423683490319 0.426337210621 0.11444 0.0922630316799 0.00342 0.00346838318819 0.037 Chr2 157769492

N00014 13400000 13600000 1.173804 scaffold_5 3396282 3592858 0.00378436747824 0.0010107229793 0.00378436747824 0.00101186529536 0.384334862512 0.384950423177 0.08671 0.0688029057464 0 0.00124633729448 NA Chr2 157769492

N00014 13800000 14000000 2.194954 scaffold_5 3015102 3205639 0.00427127856922 0.000985525033071 0.00437798901642 0.00101109361413 0.399149983879 0.396897988225 0.081595 0.069723990616 0.03122 0.0310648325522 0.101 Chr2 157769492

N00359 0 200000 2.539762 scaffold_5 2539308 2725750 0.00372361119261 0.00100018668794 0.00372833124419 0.00100102965007 0.478040581393 0.478589008342 0.13362 0.0990978427608 0.007885 0.00872121088596 0.0795 Chr2 157769492

N00074 0 200000 3.093588 scaffold_43 4048201 4243618 0.00391006872117 0.00105609340925 0.00392687959866 0.00106290399599 0.425495427844 0.421377659714 0.040285 0.0289227651637 0.009185 0.00973814970038 0.0945 Chr3 116185509

N00074 200000 400000 7.622689 scaffold_43 4243618 4440921 0.00556693017724 0.00148086147626 0.00556910913597 0.00148086147626 0.416664050482 0.417102975924 0.03642 0.0231927542916 0.00164 0.000648748371794 0.0515 Chr3 116185509

N00074 600000 800000 5.686053 scaffold_43 3826685 4022461 0.00349990139808 0.00127192248305 0.00349990139808 0.00127192248305 0.452276566965 0.44902711573 0.040215 0.0293600849951 0 0 NA Chr3 116185509

N00074 800000 1000000 3.148387 scaffold_43 3639271 3826685 0.00322740969128 0.00113807747089 0.00324374245068 0.0011421331139 0.434781368225 0.43759741676 0.055445 0.0387537750648 0.00463 0.00563458439604 0.017 Chr3 116185509

N00074 1000000 1200000 3.148387 scaffold_43 3445254 3639271 0.00408582759096 0.00140281297675 0.00422792160706 0.00147140760221 0.415929710411 0.418010017961 0.047025 0.0122205786091 0.05693 0.0633191936789 0.1065 Chr3 116185509

N00074 1200000 1400000 2.865496 scaffold_43 3248097 3445254 0.00377307946526 0.0014824983978 0.00397918186015 0.0016329238482 0.388389506468 0.387223713087 0.04229 0.0134359926353 0.09725 0.118854516959 0.0906 Chr3 116185509

N00074 1400000 1600000 2.636126 scaffold_43 3062078 3248097 0.00341183381015 0.00122979973492 0.00346236854307 0.00125727410458 0.435666668621 0.442692479288 0.09484 0.0222719184599 0.019715 0.0257231788151 0.07225 Chr3 116185509

N00074 1600000 1800000 2.594542 scaffold_43 2862751 3062078 0.00299064898715 0.00102003121704 0.00299064898715 0.00102008255498 0.449515583051 0.458528021607 0.06454 0.0390764923969 0.01046 0.0100638649054 0.028 Chr3 116185509

N00074 1800000 2000000 2.571332 scaffold_43 2671008 2862751 0.00281026181086 0.00109931770767 0.00281026181086 0.00109931770767 0.448758153872 0.453440286717 0.05691 0.0237140338891 0 0 NA Chr3 116185509

N00074 2000000 2200000 2.571332 scaffold_43 2479898 2671008 0.00305442740664 0.00110238065045 0.00305442740664 0.00110238065045 0.42945365885 0.432426453159 0.050775 0.0213698916854 0 0 NA Chr3 116185509

N00074 2200000 2400000 2.571332 scaffold_43 2288820 2479898 0.00271117100466 0.00107731285042 0.00271117100466 0.00107731285042 0.406346524317 0.407884465262 0.051965 0.0266959042904 0 0 NA Chr3 116185509

N00074 2400000 2600000 2.571332 scaffold_43 2098880 2288820 0.00304210859622 0.00109349210732 0.00306817273561 0.00110510116751 0.39622852729 0.398077965764 0.085455 0.0303201010846 0.0108 0.0150679161841 0.046 Chr3 116185509

N00074 2600000 2800000 2.571332 scaffold_43 1894274 2098880 0.00323201956393 0.00124762839853 0.00323201956393 0.00124762839853 0.422932603189 0.424890128769 0.024995 0.0218126545654 0 0 NA Chr3 116185509

N00074 2800000 3000000 2.571332 scaffold_43 1701451 1894253 0.00434506884971 0.00134040360163 0.00434878872795 0.00134293908953 0.437282989616 0.450236051054 0.034955 0.0221211398222 0.007945 0.00703312206305 0.094 Chr3 116185509

N00074 3000000 3200000 2.571332 scaffold_43 1499131 1701451 0.00370136450244 0.00127505746748 0.00378759789884 0.00130829712518 0.422226583635 0.432236444841 0.046695 0.0240757216291 0.03412 0.0358788058521 0.085 Chr3 116185509

N00074 3200000 3400000 NA scaffold_43 1311355 1498560 0.0041447884736 0.0014561513784 0.00426371023488 0.00152185731604 0.414129725239 0.428693819503 0.06599 0.0128575625651 0.06076 0.0646563927246 0.143 Chr3 116185509

N00074 3400000 3600000 NA scaffold_43 1130723 1311355 0.0041272119761 0.00134465776585 0.00428547881115 0.00140789343336 0.431887081927 0.449691941383 0.046525 0.0207770494707 0.0643 0.072262943443 0.132666666667 Chr3 116185509

N00074 3600000 3800000 NA scaffold_43 934945 1130723 0.00432140893072 0.00140357605634 0.00445511518793 0.00145995051399 0.430892655367 0.444022696796 0.04395 0.0298092737693 0.04865 0.0479165176884 0.092 Chr3 116185509

N00105 400000 600000 7.994057 scaffold_43 6874031 7070851 0.00464425164827 0.000679825779146 0.00464425164827 0.000680057266251 0.38335088968 0.385048945233 0.11011 0.0964637740067 0 0.000533482369678 NA Chr3 116185509

N00105 600000 800000 6.497266 scaffold_43 6677316 6874031 0.00410949899375 0.000988429922663 0.00410949899375 0.000988987815027 0.384329838635 0.386033715757 0.05972 0.0640520550034 0 0.000620186564319 NA Chr3 116185509

N00105 800000 1000000 1.893004 scaffold_43 6489602 6677316 0.00446003316882 0.000972399337406 0.00446003316882 0.000973222833731 0.384956600312 0.385863279799 0.044515 0.0264338301885 0 0.000783106214774 NA Chr3 116185509

N00105 1000000 1200000 1.336072 scaffold_43 6297337 6489602 0.00411430978989 0.000918020759113 0.00414304756197 0.000925779235097 0.395038102918 0.395939710858 0.091945 0.0554495097912 0.01162 0.0112396952123 0.061 Chr3 116185509

N00105 1200000 1400000 3.905145 scaffold_43 6098471 6297337 0.00479277612038 0.00109285213612 0.00489273039097 0.00111912672011 0.393896060758 0.394536520644 0.079845 0.0696197439482 0.0308 0.0316896804884 0.2025 Chr3 116185509

N00105 1400000 1600000 3.134042 scaffold_43 5895530 6098095 0.00436251844557 0.00131962609782 0.00439766882827 0.00132914688086 0.408879102071 0.41103607208 0.05323 0.0383778046553 0.00829 0.00824920395922 0.065 Chr3 116185509

N00105 1600000 1800000 7.753956 scaffold_43 5692667 5895530 0.0044119016809 0.00115750813582 0.00442843391673 0.00116471210489 0.437044412817 0.437350635428 0.089695 0.0697367188694 0.00831 0.00771949542302 0.144 Chr3 116185509

N00105 1800000 2000000 4.426604 scaffold_43 5504453 5692667 0.00406432257481 0.00114454993121 0.00406432257481 0.00114454993121 0.431453362256 0.439033806694 0.097385 0.0447575631993 0 0 NA Chr3 116185509

N00105 2000000 2200000 4.335317 scaffold_43 5308803 5504453 0.00379057322685 0.00107372074846 0.00380151565729 0.00107458877521 0.414241320189 0.418016898476 0.054985 0.0347661640685 0.003385 0.00323025811398 0.058 Chr3 116185509

N00105 2200000 2400000 4.598054 scaffold_43 5116615 5308803 0.00403694753672 0.00100762264547 0.00408978445024 0.00103124148514 0.402791854013 0.402212635226 0.049275 0.0351010468916 0.03273 0.0348512914438 0.08 Chr3 116185509

N00105 2400000 2600000 6.834623 scaffold_43 4934665 5116615 0.00412752669821 0.00128636575385 0.00416689193428 0.0013061367319 0.449062828554 0.449431154437 0.103285 0.0385050838142 0.02252 0.0323055784556 0.07975 Chr3 116185509

N00105 2600000 2800000 4.621238 scaffold_43 4742977 4934665 0.00391544947941 0.00129436012861 0.00400439826635 0.00132482083788 0.421332912121 0.418321054359 0.03595 0.0268561412295 0.033685 0.0387922039982 0.0643333333333 Chr3 116185509

N00227 200000 400000 5.033012 scaffold_81 1100365 1294833 0.00357348681122 0.0010726938157 0.00359680161709 0.0010883551519 0.483565007904 0.485544951006 0.101475 0.073580229138 0.025815 0.0272075611412 0.103 Chr3 116185509

N00227 400000 600000 4.033835 scaffold_81 1294833 1484745 0.00393915232206 0.00107473967644 0.00413784925098 0.0011374991533 0.442390150841 0.444971245705 0.08 0.0403502674923 0.06675 0.0756666245419 0.068375 Chr3 116185509

N00092 0 200000 3.143885 scaffold_81 730513 924341 0.0040718057606 0.00125126009329 0.00419618810026 0.0013051887772 0.415327664989 0.413925413738 0.07199 0.0374300926595 0.046225 0.0503590812473 0.0915 Chr3 116185509

N00092 200000 400000 3.018755 scaffold_81 538215 730513 0.00396026404333 0.00125314042664 0.00401080038653 0.00126914206821 0.44165616708 0.441948980512 0.099695 0.0575304995372 0.013775 0.0263653288126 0.201 Chr3 116185509

N00092 400000 600000 0 scaffold_81 343269 538215 0.00289041701891 0.00104302868463 0.00293179994818 0.00105751120088 0.405207977208 0.408365398705 0.05577 0.0347019174541 0.023185 0.0214674833031 0.054 Chr3 116185509

N00092 600000 800000 1.05253 scaffold_81 153489 343269 0.00342022999408 0.00106938684183 0.00345624095292 0.00108701427796 0.399975934404 0.398586023562 0.108195 0.0511434292338 0.015935 0.0170355148066 0.1005 Chr3 116185509

N00092 1000000 1200000 2.386795 scaffold_80 2089961 2278209 0.0034273477462 0.00106038143079 0.00347573906322 0.00107519344314 0.433877434135 0.432564175263 0.06339 0.0283987080872 0.01862 0.0202551952743 0.0495 Chr3 116185509

N00092 1200000 1400000 2.42029 scaffold_80 1890785 2089961 0.00340960384995 0.00108273520028 0.00345894782991 0.00110532365931 0.434205025524 0.431545792718 0.051485 0.0368568502229 0.02779 0.0317859581476 0.055 Chr3 116185509

N00092 1400000 1600000 1.415772 scaffold_80 1699035 1890785 0.00335955513149 0.000981868562154 0.00335972049436 0.000981872532458 0.421572110581 0.415047599213 0.08352 0.0505606258149 8,00E-05 8.86571056063e-05 0.019 Chr3 116185509

N00092 1600000 1800000 1.14115 scaffold_80 1509141 1699035 0.00306130611833 0.000908686399051 0.00306130611833 0.000908686399051 0.409584166731 0.404248959228 0.078775 0.046831390144 0 0 NA Chr3 116185509

N00092 1800000 2000000 0.8645455 scaffold_80 1321570 1509141 0.00252108622052 0.000784088001487 0.00253084329247 0.000786739744802 0.425301024001 0.42488119428 0.08561 0.0324623742476 0.00585 0.00556589238208 NA Chr3 116185509

N00092 2000000 2200000 0.8886463 scaffold_80 1131157 1321570 0.00217436020406 0.000849421130124 0.00217436020406 0.000849421130124 0.406159769009 0.400132028202 0.060045 0.0286850162541 0 0 NA Chr3 116185509

N00092 2200000 2400000 0.8886463 scaffold_80 943768 1131157 0.00172059815012 0.000955079757333 0.0017332851597 0.00095895960893 0.389904502046 0.382548166276 0.120565 0.0591123278314 0.01285 0.010694331044 0.103 Chr3 116185509

N00092 2400000 2600000 0.1856873 scaffold_80 753710 943768 0.00210878378494 0.000989132881476 0.00210878378494 0.000989132881476 0.398570156956 0.391494264445 0.105055 0.0631701901525 0 0 NA Chr3 116185509

N00092 2600000 2800000 0.02215012 scaffold_80 557076 753710 0.00177354359242 0.00102593697447 0.00178535171624 0.0010403517327 0.389640238462 0.386704961265 0.13593 0.093590121749 0.0124 0.0124952958288 0.0785 Chr3 116185509

N00236 200000 400000 0.2885067 scaffold_18 5110642 5308518 0.000999410224367 0.00126339922196 0.00103662424742 0.00132726752298 0.447727731189 0.440936791799 0.0932 0.0436839232651 0.05521 0.0569447532798 0.0783333333333 Chr3 116185509

N00236 400000 600000 0.4426705 scaffold_18 4915764 5110642 0.00207427220853 0.00152938036433 0.00207427220853 0.00152938036433 0.394386631979 0.384983877865 0.134675 0.0722708566385 0 0 NA Chr3 116185509

N00011 0 200000 0.4426705 scaffold_18 4180317 4368224 0.0029613426588 0.00127462769298 0.00296242045156 0.00127487804549 0.36745088109 0.362874972319 0.083395 0.0594762302628 3,00E-04 0.000149009882548 0.05 Chr3 116185509

N00011 200000 400000 1.639858 scaffold_18 3992267 4180317 0.00309688372928 0.00175685578675 0.00311472103597 0.00177214449993 0.364821199601 0.359552651771 0.13173 0.0571443764956 0.00771 0.0083116192502 0.05 Chr3 116185509

N00011 400000 600000 3.457085 scaffold_18 3800880 3992267 0.00291082236835 0.00123709234468 0.00292393037493 0.00124291998564 0.377378116297 0.372825668919 0.083935 0.0515238757073 0.005995 0.00612371791187 0.05 Chr3 116185509

N00011 600000 800000 2.085546 scaffold_18 3608528 3800880 0.00323058521211 0.00138465594151 0.0032408734442 0.00138904407676 0.388994153751 0.384183005843 0.051305 0.0293732324073 0.00487 0.00461653635002 0.05 Chr3 116185509

N00011 800000 1000000 2.085546 scaffold_18 3414287 3608528 0.00342841573295 0.0013546512999 0.00342841573295 0.0013546512999 0.411463306317 0.406646840471 0.07847 0.0556473659011 0 0 NA Chr3 116185509

N00011 1000000 1200000 2.085546 scaffold_18 3224818 3414287 0.00336868639883 0.00125114246618 0.00337134128045 0.00125920658539 0.41310685982 0.408997068653 0.11387 0.0606959449831 0.00933 0.0109886049961 0.105 Chr3 116185509

N00011 1200000 1400000 2.085546 scaffold_18 3039154 3224818 0.00361932876764 0.0012918761078 0.00372414881176 0.00133463566775 0.415764183177 0.416460754525 0.10832 0.0453830575664 0.04581 0.0502035935884 0.085 Chr3 116185509

N00011 1400000 1600000 4.024911 scaffold_18 2837174 3039154 0.00330791346653 0.00109200480078 0.00335953165011 0.00111857676252 0.409621198843 0.407778954999 0.077105 0.0538469155362 0.02615 0.0312803247846 0.0625 Chr3 116185509

N00011 1600000 1800000 5.099726 scaffold_18 2644535 2837174 0.00400529296864 0.00112530894847 0.00408171735587 0.00114953061043 0.412288523223 0.410105001953 0.096025 0.0446534710002 0.03745 0.031847133758 0.092 Chr3 116185509

N00011 2000000 2200000 1.671345 scaffold_18 2260088 2452899 0.00382505693442 0.00108461296677 0.00382846345547 0.00108807750512 0.399867212571 0.397120964102 0.079735 0.0581605821245 8,00E-04 0.00347490547738 0.067 Chr3 116185509

N00011 2400000 2600000 5.709472 scaffold_18 1889214 2078454 0.00386711900176 0.00116955127336 0.00388215058917 0.00117346529788 0.435719814241 0.434845792752 0.081745 0.0331642358909 0.00492 0.00503064891144 0.1175 Chr3 116185509

N00011 2600000 2800000 4.925581 scaffold_18 1702244 1889214 0.00372643608864 0.0011116315682 0.00375914828764 0.00112199995343 0.461344338394 0.45784092193 0.09417 0.0497192062898 0.02074 0.0206824624271 0.128666666667 Chr3 116185509

N00011 2800000 3000000 2.418192 scaffold_18 1509235 1702244 0.00399564031428 0.00117065635097 0.00410293357261 0.00120981915277 0.46188663259 0.456215089894 0.04348 0.0246620623909 0.03988 0.0408996471667 0.078 Chr3 116185509

N00011 3000000 3200000 1.56802 scaffold_18 1314033 1509235 0.00438597687194 0.00128196257952 0.00442604449719 0.00129802204873 0.388977137807 0.383066184637 0.06917 0.0338008831877 0.012295 0.0121873751294 0.0595 Chr3 116185509

N00011 3200000 3400000 1.007772 scaffold_18 1119100 1314033 0.00381656585474 0.00112750560182 0.00389639768216 0.00116137324394 0.435121034708 0.429422510409 0.05386 0.0320109986508 0.034695 0.0353865174188 0.0515 Chr3 116185509

N00011 3600000 3800000 2.987013 scaffold_18 739535 935759 0.00379951374402 0.00123313197587 0.00390893012106 0.00127055568006 0.426192424597 0.420982478392 0.108745 0.053836431833 0.04114 0.0406830968689 0.0776 Chr3 116185509

N00011 3800000 4000000 3.67809 scaffold_18 543922 739535 0.00353974533245 0.00122710190574 0.00358605356443 0.00124273700896 0.409100211852 0.407076122433 0.08885 0.0383870192676 0.020015 0.0208575094702 0.101666666667 Chr3 116185509

N00011 4000000 4200000 4.047368 scaffold_18 343512 543922 0.00395606395099 0.00108052368838 0.00402942867193 0.00109774124019 0.392351166817 0.392160922113 0.08211 0.0468489596328 0.02295 0.0198193702909 0.069 Chr3 116185509

N00011 4600000 4800000 8.7976 scaffold_18 5682885 5879436 0.00392479929297 0.00114287962207 0.00400733313409 0.00118944897135 0.404305148958 0.404495210858 0.07939 0.0382140004375 0.03986 0.0618211049549 0.0763333333333 Chr3 116185509

N00011 4800000 5000000 3.390916 scaffold_18 5879436 6077107 0.0041114740159 0.00115622405418 0.0041114740159 0.00115628685992 0.429464540329 0.430491250951 0.079855 0.0568115707413 0 0.00269134066201 NA Chr3 116185509

N00011 5000000 5200000 2.452647 scaffold_18 6077107 6270239 0.0041639051183 0.00131518691125 0.00417160571953 0.00132007886419 0.448277191301 0.452724812533 0.06542 0.0427634985399 0.00721 0.00748710726343 0.084 Chr3 116185509

N00011 5200000 5400000 5.606156 scaffold_18 6270239 6464568 0.00375949549716 0.00105809905418 0.00375984070809 0.00105906369561 0.432980041294 0.438642804038 0.069235 0.0291207179577 0.00262 0.00242372471427 0.044 Chr3 116185509

N00011 5600000 5800000 3.594771 scaffold_18 6638985 6829587 0.00386000882986 0.00131441446809 0.00402835985604 0.00135738037614 0.393174157475 0.394879681013 0.107435 0.0576489228864 0.07047 0.0722605219253 0.792 Chr3 116185509

N00011 5800000 6000000 4.866835 scaffold_18 6829587 7021232 0.0040655850173 0.00110395594141 0.00407104780936 0.00110633443757 0.399866831127 0.398573275243 0.058295 0.0419160426831 0.001765 0.00185760129406 0.048 Chr3 116185509

N00011 6000000 6200000 5.56519 scaffold_18 7021232 7214241 0.00400470807486 0.00101030837077 0.00401072862431 0.00101160496998 0.399370337114 0.396142919287 0.06628 0.0561061919392 0.00177 0.00183929246823 0.048 Chr3 116185509

N00011 6200000 6400000 3.846821 scaffold_18 7214241 7408553 0.00395476768916 0.00121459627262 0.00397501195904 0.00122452591864 0.411217007992 0.417108987024 0.05412 0.0459621639425 0.00979 0.0101743587632 0.0935 Chr3 116185509

N00011 6400000 6600000 3.209302 scaffold_18 7408553 7595406 0.00404767044618 0.00108493150519 0.0040474534957 0.00108631718792 0.385770002229 0.38559728152 0.08071 0.0367561666123 0.001085 0.00117739613493 0.157 Chr3 116185509

N00011 6600000 6800000 1.900556 scaffold_18 7595406 7787484 0.00430971223687 0.00118604271768 0.00435183255988 0.00119798008406 0.358561571066 0.356270655393 0.056295 0.0440810504066 0.011985 0.0127083788877 0.1075 Chr3 116185509

N00011 6800000 7000000 4.047457 scaffold_18 7787484 7975512 0.00353086611159 0.0010548504851 0.00353086611159 0.0010548504851 0.404787123747 0.407900252593 0.069635 0.0307773310358 0 0 NA Chr3 116185509

N00011 7000000 7200000 3.842352 scaffold_18 7975512 8169449 0.00342767508507 0.0010440385314 0.00343088391327 0.00104565099673 0.396963548813 0.397088878142 0.036145 0.0182172561193 0.00236 0.00232549745536 0.045 Chr3 116185509

N00011 7200000 7400000 1.820221 scaffold_18 8169449 8357397 0.00383463667138 0.00109397493375 0.00386587734395 0.00110769128263 0.383558683365 0.383442715865 0.034835 0.0242886330262 0.01227 0.0132696277694 0.045 Chr3 116185509

N00011 7400000 7600000 4.254237 scaffold_18 8357397 8548078 0.00424457607997 0.00114406036605 0.00432461829602 0.00116910759839 0.359861820764 0.355557697542 0.0843 0.0963441559463 0.02881 0.0301236095888 NA Chr3 116185509

N00011 7600000 7800000 3.906857 scaffold_18 8548078 8752918 0.00406825586979 0.000988470780561 0.00416397429422 0.00102412012047 0.383375595844 0.380984008871 0.05943 0.0796133567663 0.05225 0.0521187268112 0.042 Chr3 116185509

N00011 7800000 8000000 2.768978 scaffold_18 8752918 8943866 0.00437983496152 0.00116723667931 0.00440224719219 0.00117355128811 0.386820942432 0.388402290419 0.07444 0.0492909064248 0.006325 0.00664578838218 0.032 Chr3 116185509

N00011 8000000 8200000 2.178977 scaffold_18 8944662 9136521 0.00441808923402 0.000990872351301 0.00449843450097 0.00103145486746 0.390944589067 0.3913016646 0.050865 0.0342491100235 0.05586 0.0591997247979 NA Chr3 116185509

N00011 8200000 8400000 4.051671 scaffold_18 9136521 9323071 0.00343378997701 0.000846275672683 0.00348246030546 0.000856829082004 0.412670163789 0.42205418538 0.07061 0.0327686947199 0.02185 0.0232538193514 0.0475 Chr3 116185509

N00011 8400000 8600000 1.292568 scaffold_18 9323113 9508202 0.00355805378597 0.00105041129837 0.00355805378597 0.00105041129837 0.41996027973 0.426929526547 0.08155 0.0396890144741 0 0 NA Chr3 116185509

N00011 8600000 8800000 1.153536 scaffold_18 9508202 9702801 0.00357529012257 0.000901947478724 0.00361639349459 0.000909536478226 0.407093154971 0.410149817789 0.06004 0.0337411805816 0.01381 0.013581775857 0.058 Chr3 116185509

N00011 8800000 9000000 4.458861 scaffold_18 9702801 9897234 0.00409411364848 0.00112274078354 0.00418753612436 0.00114922052599 0.42457848492 0.430281011089 0.09847 0.0713253408629 0.030935 0.028714261468 0.063875 Chr3 116185509

N00011 9000000 9200000 5.134348 scaffold_18 9897234 10086340 0.00450022067477 0.0010910676482 0.00458093828252 0.00111446557163 0.402906142043 0.404339359607 0.07156 0.0174505303904 0.02462 0.0245840956924 0.0504 Chr3 116185509

N00011 9200000 9400000 2.090615 scaffold_18 10086340 10285813 0.00415422923073 0.00131261952322 0.00424804724302 0.00134730896063 0.410955022505 0.411012493394 0.04485 0.0357642387692 0.034915 0.0312272838931 0.074 Chr3 116185509

N00011 9400000 9600000 2.090615 scaffold_18 10285813 10472792 0.0036888257023 0.000977889633847 0.00374633702653 0.000996787801799 0.415464130659 0.414847487609 0.10312 0.0409939084068 0.02671 0.0298215307601 0.104 Chr3 116185509

N00011 9600000 9800000 2.090615 scaffold_18 10472792 10669541 0.00424805642588 0.000992941150326 0.00425982555937 0.000996489982197 0.404184027589 0.399423153499 0.061445 0.0555377663927 0.009735 0.00985011359651 0.099 Chr3 116185509

N00011 9800000 10000000 2.948249 scaffold_18 10669541 10863086 0.00419834227239 0.00101232769126 0.00425293331224 0.00102756213404 0.400470328617 0.397443094967 0.084355 0.049647368829 0.01632 0.0172414683924 0.071 Chr3 116185509

N00011 10000000 10200000 2.974638 scaffold_18 10863086 11061722 0.0041298551671 0.00103733583206 0.00420359258434 0.00106296888557 0.400018781002 0.398256427342 0.06675 0.0435570591434 0.032805 0.0395446948187 0.08625 Chr3 116185509

N00011 10200000 10400000 2.974638 scaffold_18 11061722 11255059 0.00415165109981 0.00100870126969 0.00432264330135 0.00105138444249 0.391036292856 0.393900373541 0.0868 0.0485111489265 0.04822 0.0486611460817 0.062 Chr3 116185509

N00011 10400000 10600000 2.974638 scaffold_18 11255059 11445048 0.0037512754586 0.00124649588821 0.00375357608229 0.0012467145062 0.405594405594 0.407586102914 0.069555 0.0419971682571 0.000495 0.000678986678176 0.077 Chr3 116185509

N00011 10600000 10800000 2.798611 scaffold_18 11448409 11640107 0.00380768148401 0.00128695091273 0.00382207038163 0.00129319915327 0.401781935386 0.402915002822 0.04772 0.0265104487266 0.004405 0.00439754196705 0.077 Chr3 116185509

N00011 10800000 11000000 1.484475 scaffold_18 11640107 11826226 0.00439411032194 0.00118900593529 0.00440551937427 0.00119421593983 0.397519433331 0.397250692707 0.06728 0.0207125548708 0.003395 0.00376103460689 0.054 Chr3 116185509

N00011 11000000 11200000 4.374365 scaffold_18 11826226 12022057 0.00495463807934 0.00112660873478 0.00500195168505 0.00113685310502 0.39403300589 0.3927412577 0.048695 0.0486593031747 0.011315 0.0115916274747 0.054 Chr3 116185509

N00011 11200000 11400000 2.824194 scaffold_18 12022057 12215044 0.00476112556768 0.00115566433437 0.00482409288822 0.00117255408161 0.372959795515 0.374048582116 0.06152 0.0454227486826 0.01793 0.0155761787063 0.0603333333333 Chr3 116185509

N00011 11400000 11600000 2.841155 scaffold_18 12215044 12412518 0.00464419309612 0.00130545306688 0.00464713150408 0.00131236129896 0.387301166783 0.387339649535 0.04763 0.0632133850532 0.004325 0.00444109097907 0.095 Chr3 116185509

N00011 11600000 11800000 3.03856 scaffold_18 12412518 12609311 0.0044353177571 0.00137855995218 0.0044353177571 0.00137855995218 0.374974236589 0.375506726362 0.05521 0.062354860183 0 0 NA Chr3 116185509

N00011 11800000 12000000 2.137129 scaffold_18 12609311 12803193 0.0038091361506 0.00121771616974 0.0038091361506 0.00121771616974 0.39451345977 0.394902840912 0.03393 0.0351038260385 0 0 NA Chr3 116185509

N00011 12000000 12200000 3.863096 scaffold_18 12803193 12988682 0.00396676031108 0.000921400044044 0.00396676031108 0.000921400044044 0.39711809474 0.400433665776 0.03843 0.0301149933419 0 0 NA Chr3 116185509

N00011 12200000 12400000 3.631908 scaffold_18 12988682 13183838 0.00375289425399 0.000976212713914 0.00375289425399 0.000976212713914 0.412906558163 0.416739274478 0.041655 0.0275676894382 0 0 NA Chr3 116185509

N00011 12400000 12600000 3.631908 scaffold_18 13183838 13375467 0.00446138183772 0.00136348120141 0.0045370983808 0.0013961314854 0.379389224338 0.378953174798 0.070975 0.0422065553752 0.03152 0.0326516341472 0.068 Chr3 116185509

N00011 12600000 12800000 3.631908 scaffold_18 13375467 13561057 0.00376571205308 0.000894654382142 0.00395418164291 0.000948801568242 0.365765236723 0.373508393447 0.044265 0.0216660380408 0.064865 0.07186270812 0.077 Chr3 116185509

N00011 12800000 13000000 3.631908 scaffold_18 15524140 15717771 0.00398987007996 0.00109753713562 0.00406228460499 0.0011178348579 0.416619331499 0.410335978994 0.04721 0.0282909244904 0.031155 0.0251922471092 0.0955 Chr3 116185509

N00011 13000000 13200000 2.270391 scaffold_18 15333955 15524140 0.00387216392825 0.00108448259159 0.00389737092024 0.00108863300915 0.427308315335 0.426258552943 0.073895 0.0518021926019 0.00609 0.00732444724873 0.0325 Chr3 116185509

N00011 13200000 13400000 1.007843 scaffold_18 15141311 15333955 0.00407588649084 0.00113107787313 0.00410093832642 0.00114418637753 0.415672381716 0.409296327384 0.08875 0.0503882809742 0.00943 0.0113629285106 0.056 Chr3 116185509

N00011 13400000 13600000 0.7751412 scaffold_18 14948163 15140596 0.00418072896773 0.00111540563292 0.00421594107291 0.00113223414654 0.400692178945 0.395055462916 0.086925 0.0519037794973 0.016095 0.0193054205879 0.0673333333333 Chr3 116185509

N00011 13600000 13800000 0.7751412 scaffold_18 14747535 14948163 0.00406742936165 0.00094389452563 0.0041136649005 0.000958977098007 0.406533699414 0.405210910142 0.049885 0.0291584424906 0.016295 0.0174003628606 0.0575 Chr3 116185509

N00011 13800000 14000000 0.7751412 scaffold_18 14548636 14747535 0.00456734916202 0.00100069809788 0.00459092720166 0.00100414611136 0.379542331425 0.379389561752 0.05779 0.0436804609375 0.00631 0.00634492883323 0.054 Chr3 116185509

N00011 14000000 14200000 0.7751412 scaffold_18 14347651 14548636 0.00444024595393 0.00107034028873 0.00447695853056 0.00108283245722 0.379039976441 0.379927991843 0.043275 0.0284946637809 0.015065 0.0161952384506 0.068 Chr3 116185509

N00011 14200000 14400000 0.7751412 scaffold_18 14146470 14347651 0.00397046062781 0.000982480684269 0.00397046062781 0.000982641557871 0.405662599015 0.406552292023 0.057395 0.0355649887415 0.00454 0.00193855284545 0.007 Chr3 116185509

N00011 14400000 14600000 0.7751412 scaffold_18 13960502 14146470 0.00367926724185 0.00118908031566 0.00368106434995 0.00118908031566 0.418658768775 0.424209579467 0.09994 0.0280370816485 0.01106 0 0.007 Chr3 116185509

N00011 14600000 14800000 0.7751412 scaffold_18 13763774 13960502 0.00340405357453 0.000857404192369 0.00341125681849 0.000860155675054 0.357876071644 0.357307313961 0.056645 0.0595695579684 0.002965 0.0030295636615 NA Chr3 116185509

N00011 14800000 15000000 0.7751412 scaffold_18 13568193 13763774 0.0036784448692 0.000933998673852 0.00375113257552 0.000956229311074 0.38440228882 0.385643880095 0.10604 0.0852230022344 0.028645 0.0296092156191 0.0586666666667 Chr3 116185509

N00018 0 200000 2.162656 scaffold_18 15727433 15921729 0.003972389226 0.00101417339346 0.00399221149513 0.00101876793959 0.380345289849 0.377107035808 0.06226 0.0409426853872 0.006825 0.00650039115576 0.0375 Chr3 116185509

N00018 200000 400000 4.372788 scaffold_18 15921729 16110951 0.00349729756392 0.000875809460089 0.00352279031224 0.000881698005321 0.436784057287 0.436354694669 0.092155 0.0635285537622 0.00888 0.0110029489171 0.036 Chr3 116185509

N00018 400000 600000 3.419463 scaffold_18 16110951 16305411 0.00403268138621 0.00102701818235 0.0040627485365 0.00103424621991 0.426450704696 0.435039917168 0.06998 0.0540573896945 0.01264 0.014686825054 0.088 Chr3 116185509

N00018 800000 1000000 1.140333 scaffold_14 16674593 16868599 0.00442345610908 0.00127899305591 0.00442345610908 0.00127899305591 0.396610207068 0.400692690436 0.077195 0.0695648588188 0 0 NA Chr3 116185509

N00018 1000000 1200000 1.140333 scaffold_14 16483801 16674593 0.00438125389497 0.00125429655066 0.00440629169132 0.0012671353056 0.389807202662 0.388147176085 0.08926 0.0853232839951 0.01144 0.0120130823095 0.082 Chr3 116185509

N00018 1200000 1400000 1.140333 scaffold_14 16288879 16483801 0.00494271758872 0.00133737994303 0.00494277591743 0.00133737994303 0.389137459607 0.38387140203 0.0712 0.0484706703194 2,00E-05 0 NA Chr3 116185509

N00018 1400000 1600000 2.69153 scaffold_14 16092714 16288879 0.00392337256027 0.00110297607456 0.00397753301254 0.00112076663496 0.412293486182 0.40840792582 0.0382 0.0373257206943 0.025865 0.0255550174598 0.03 Chr3 116185509

N00018 1800000 2000000 1.584846 scaffold_14 15713078 15900221 0.00446482621905 0.00118117418005 0.0045026896044 0.00120217253291 0.400064774936 0.397641985902 0.06316 0.0313931058068 0.016055 0.0208183047189 0.0685 Chr3 116185509

N00018 2000000 2200000 1.584846 scaffold_14 15518492 15713078 0.00381311462616 0.00104447600217 0.00389889831856 0.00106901532374 0.394048070233 0.391060470287 0.090735 0.0583752171276 0.027795 0.0263174123524 0.055 Chr3 116185509

N00018 2200000 2400000 1.584846 scaffold_14 15324780 15518492 0.00480995069784 0.00124836301486 0.00504285936224 0.00132573863573 0.346258109713 0.34335514069 0.095725 0.0813785413397 0.06232 0.062809738168 0.0536666666667 Chr3 116185509

N00018 2400000 2600000 1.584846 scaffold_14 15126852 15324780 0.00375770123024 0.0010920149804 0.00377264712049 0.00109689008382 0.381184960519 0.375399866236 0.0316 0.029283375773 0.004015 0.00455721272382 0.044 Chr3 116185509

N00018 2600000 2800000 1.584846 scaffold_14 14934267 15126852 0.00371883267361 0.00120038192139 0.00371883267361 0.00120038736554 0.44429118092 0.444749212027 0.08248 0.0501544772438 0.000305 0.000659449074435 0.083 Chr3 116185509

N00018 2800000 3000000 1.584846 scaffold_14 14733865 14934267 0.00384908786883 0.00106756899758 0.00386034147503 0.00107874316194 0.474534993952 0.475907626208 0.055085 0.0578986237662 0.013895 0.0139569465375 0.083 Chr3 116185509

N00018 3000000 3200000 1.099418 scaffold_14 14538892 14733865 0.00350627780207 0.000918429333728 0.00363322785456 0.000976254125756 0.487850976941 0.488719122499 0.08493 0.0645268832095 0.07282 0.0821088048089 0.0866666666667 Chr3 116185509

N00018 3200000 3400000 1.074003 scaffold_14 14343400 14538892 0.00396551119309 0.00100114563497 0.00399484968207 0.00101575809367 0.523597085812 0.526822802571 0.062425 0.0227170421296 0.028565 0.0511478730587 0.07175 Chr3 116185509

N00018 3400000 3600000 1.074003 scaffold_14 14148183 14343400 0.00367715480332 0.000992563727969 0.00367715480332 0.000992563727969 0.45709009985 0.45897652837 0.06031 0.0359753505074 0 0 NA Chr3 116185509

N00018 3600000 3800000 1.074003 scaffold_14 13955589 14148183 0.00335251698443 0.000902042090298 0.00335639793445 0.00090498176428 0.473439272931 0.474923917348 0.07496 0.0390562530505 0.003315 0.0032555531325 0.0225 Chr3 116185509

N00018 3800000 4000000 1.074003 scaffold_14 13756396 13955589 0.00441461226081 0.00105632200022 0.00443870221639 0.00106570576729 0.402880217229 0.399713404837 0.06665 0.0538522940063 0.01217 0.0126410064611 0.0566666666667 Chr3 116185509

N00018 4000000 4200000 1.074003 scaffold_14 13553268 13756396 0.0048630698785 0.00136603978845 0.00486417818419 0.00136686216955 0.368714815507 0.36438568287 0.063315 0.0719398605805 0.000925 0.00100429285967 0.029 Chr3 116185509

N00018 4200000 4400000 1.074003 scaffold_14 13352756 13553268 0.00402986527736 0.00113390811206 0.00413334855968 0.0011717797636 0.361332999805 0.361286324503 0.06135 0.058265839451 0.03242 0.0343321097989 0.058 Chr3 116185509

N00018 4400000 4600000 1.729709 scaffold_14 13158193 13352756 0.00352065159599 0.000998880468105 0.00353233437868 0.00100867785645 0.376551956266 0.375953038154 0.062625 0.0344567055401 0.00806 0.00831607242898 0.0485 Chr3 116185509

N00018 4600000 4800000 4.636086 scaffold_14 12976788 13158193 0.00326749150054 0.000836685184833 0.00328723949922 0.000843508706528 0.435117255171 0.437508213206 0.11717 0.0303574873901 0.00796 0.00879799344009 0.005 Chr3 116185509

N00018 4800000 5000000 4.636086 scaffold_14 12778901 12976788 0.00368437857375 0.00117911055191 0.00375857091999 0.00120105161845 0.401098038336 0.399481200585 0.09248 0.0509179481219 0.02293 0.0229373329223 0.063 Chr3 116185509

N00018 5000000 5200000 4.636086 scaffold_14 12579627 12778178 0.00455520333352 0.00110924029524 0.00457174955159 0.00111257381475 0.394961523489 0.393657820226 0.049005 0.0508836520592 0.00444 0.00465371617368 0.107 Chr3 116185509

N00018 5200000 5400000 1.474335 scaffold_14 12385171 12579627 0.00414783393284 0.00129479000867 0.00419420646249 0.00131903210432 0.393652328725 0.394211484632 0.051235 0.0344808079977 0.02882 0.029636524458 0.093 Chr3 116185509

N00018 5400000 5600000 1.163343 scaffold_14 12180061 12385171 0.00488056031944 0.00119497633404 0.00488056031944 0.00119689978596 0.383618306325 0.381976837274 0.06311 0.0802398712886 0 0.00274974403978 NA Chr3 116185509

N00018 5600000 5800000 1.163343 scaffold_14 11984572 12180061 0.00459738558273 0.00127412030192 0.00465168317773 0.00129625827625 0.371753712091 0.373320346225 0.07741 0.0659116369719 0.01925 0.0241701579117 0.0926666666667 Chr3 116185509

N00018 6000000 6200000 1.163343 scaffold_14 11594337 11786396 0.00450220564653 0.00124674630919 0.00469187737908 0.00131116583797 0.381785548582 0.379812563323 0.11556 0.0667294945824 0.05612 0.0626317954379 0.0555 Chr3 116185509

N00018 6200000 6400000 0.7671089 scaffold_14 11393779 11593554 0.00483710126626 0.00113486823561 0.00483710126626 0.00113486823561 0.387035948934 0.38532902181 0.055265 0.0628957577274 0 0 NA Chr3 116185509

N00018 6400000 6600000 0.5507042 scaffold_14 11195275 11393779 0.00454241570686 0.00124196608609 0.00454241570686 0.00124196608609 0.380362275611 0.380004754855 0.07463 0.0711018417765 0 0 NA Chr3 116185509

N00018 6600000 6800000 0.5507042 scaffold_14 10989673 11195275 0.00479089458401 0.00126108487258 0.00479089458401 0.00126108487258 0.377862113917 0.380293586221 0.056645 0.0729662162819 0 0 NA Chr3 116185509

N00018 6800000 7000000 0.5507042 scaffold_14 10782090 10989673 0.00489054216689 0.00137305969162 0.00489054216689 0.00137305969162 0.38211651297 0.382456409106 0.086085 0.106516429573 0 0 NA Chr3 116185509

N00018 7000000 7200000 0.5507042 scaffold_14 10582402 10782090 0.00425837422519 0.00112420741978 0.0042580799438 0.00112420741978 0.392001288383 0.394685279766 0.078485 0.0671447458035 0.00081 0 NA Chr3 116185509

N00018 7200000 7400000 0.5507042 scaffold_14 10385644 10582402 0.00417986388772 0.0010609229916 0.0041962955908 0.00106510578259 0.376598234696 0.382766249745 0.08779 0.053476859899 0.0059 0.00543307006577 0.102 Chr3 116185509

N00018 7400000 7600000 0.5507042 scaffold_14 10195689 10385062 0.00429785506697 0.00128654447927 0.0043717881246 0.00132034930411 0.398903717013 0.395216437823 0.045765 0.0360030204939 0.028735 0.0314775601591 0.0795 Chr3 116185509

N00018 7600000 7800000 1.042714 scaffold_14 10008913 10195689 0.00416479224517 0.00109691725168 0.00434831749191 0.00115843112234 0.373118548639 0.373568063769 0.09558 0.0668608386516 0.05661 0.0644408275153 0.1202 Chr3 116185509

N00018 7800000 8000000 1.449741 scaffold_14 9811692 10007821 0.00383577268935 0.000972700186385 0.00385908772474 0.000980766276454 0.400083077179 0.400131966461 0.055125 0.0457403035757 0.01282 0.0111355281473 0.0795 Chr3 116185509

N00018 8000000 8200000 1.449741 scaffold_14 9619697 9811692 0.00370963537285 0.00112107910626 0.00382685731674 0.00115253959796 0.380169021718 0.37580845669 0.0522 0.0392822729759 0.03521 0.0339800515638 0.0446666666667 Chr3 116185509

N00018 8200000 8400000 1.449741 scaffold_14 9426144 9619697 0.0040931294262 0.00106545430247 0.00424823316838 0.0011033742729 0.365559015776 0.362136676157 0.061905 0.0398908826006 0.04938 0.0511849467588 0.0615 Chr3 116185509

N00018 8400000 8600000 1.449741 scaffold_14 9231073 9426144 0.00398522872177 0.00116597906992 0.00401587379052 0.00117564098292 0.379806903791 0.377259601112 0.05924 0.0448298311897 0.01592 0.0106063945948 0.0695 Chr3 116185509

N00018 8600000 8800000 0.4039079 scaffold_14 9043031 9231073 0.00354696800092 0.000988588062889 0.00362283837092 0.00101725538294 0.389621349178 0.393680914083 0.05378 0.043064847215 0.032935 0.036401442231 0.054 Chr3 116185509

N00018 8800000 9000000 0.1414951 scaffold_14 8854186 9043031 0.00317408476814 0.000941127478614 0.00329677869153 0.0010132092906 0.453177312093 0.45516469327 0.06032 0.028388360825 0.09613 0.105478037544 0.0595 Chr3 116185509

N00018 9000000 9200000 0.812586 scaffold_14 8653193 8854186 0.00412822458755 0.00118449236597 0.00427660889995 0.00124040186965 0.40322142374 0.406027871306 0.08405 0.065360485191 0.06031 0.0596388928968 0.0835714285714 Chr3 116185509

N00018 9200000 9400000 0.812586 scaffold_14 8443908 8653193 0.00388244216781 0.00111346706601 0.00389583943224 0.00111781561269 0.381798744523 0.382325660735 0.056765 0.0804883293117 0.00515 0.00495496571661 0.0655 Chr3 116185509

N00018 9400000 9600000 0.812586 scaffold_14 8244569 8443908 0.00390623785177 0.00110355378092 0.00390924943499 0.00110384228323 0.364979312128 0.364644082333 0.06622 0.0678943909621 0.000685 0.000687271432083 0.073 Chr3 116185509

N00018 9600000 9800000 0.812586 scaffold_14 8058411 8244569 0.00472719551445 0.00129348715602 0.00473245081369 0.00129730246993 0.359500085039 0.35724003459 0.086915 0.0795345888976 0.0028 0.00300819733775 0.073 Chr3 116185509

N00018 9800000 10000000 0.812586 scaffold_14 7867637 8058411 0.0046517483525 0.00133515701572 0.00465531302448 0.00133715971668 0.358651143667 0.358016880021 0.085365 0.0631322926604 0.001015 0.00141528719847 0.072 Chr3 116185509

N00018 10000000 10200000 0.812586 scaffold_14 7672604 7867637 0.00417293892876 0.000988515962299 0.0041828764691 0.000991215381181 0.343472589886 0.342071557138 0.111485 0.0657427204627 0.002285 0.00260468741187 0.071 Chr3 116185509

N00018 10200000 10400000 0.812586 scaffold_14 7481613 7672604 0.00410782177445 0.00126522995993 0.00411591311005 0.00126931922206 0.374377941073 0.375618331653 0.04946 0.0178123576504 0.003155 0.00334047154054 0.042 Chr3 116185509

N00018 10400000 10600000 0.812586 scaffold_14 7287614 7481613 0.00372738886599 0.00135000990107 0.00373583341501 0.00135274104399 0.413955755328 0.416949653034 0.04938 0.0420775364821 0.00237 0.00264949819329 0.013 Chr3 116185509

N00018 10600000 10800000 0.812586 scaffold_14 7087930 7287614 0.00395217517973 0.00101935761946 0.00395217517973 0.00101935761946 0.409808797615 0.411280744095 0.073025 0.085685382905 0 0 NA Chr3 116185509

N00018 10800000 11000000 0.812586 scaffold_14 6883261 7087897 0.00440084620701 0.00113054052806 0.00440084620701 0.00113054052806 0.384786450584 0.383427418489 0.068185 0.0901698625853 0 0 NA Chr3 116185509

N00018 11000000 11200000 0.812586 scaffold_14 6682694 6883261 0.00462650344621 0.00127007978855 0.00462958466868 0.00127086649765 0.383718334414 0.384979771187 0.06631 0.0613809849078 0.000605 0.000588332078557 0.047 Chr3 116185509

N00018 11200000 11400000 0.812586 scaffold_14 6482246 6682694 0.00454108947365 0.00115776198955 0.0045798692331 0.001171153575 0.372408004116 0.376091038468 0.064635 0.0514497525543 0.011315 0.0147120450192 0.047 Chr3 116185509

N00018 11400000 11600000 0.812586 scaffold_14 6295111 6482246 0.00418618906119 0.00116773327286 0.00418618906119 0.00116773327286 0.40328554497 0.404136579662 0.07627 0.0421513880354 0 0 NA Chr3 116185509

N00018 11600000 11800000 0.812586 scaffold_14 6105112 6295111 0.00474128709614 0.00117960530268 0.00478556701248 0.00119350374862 0.400999339844 0.404191128964 0.08479 0.0350054473971 0.01585 0.0199264206654 0.1475 Chr3 116185509

N00018 11800000 12000000 9.706044 scaffold_14 5897301 6105112 0.00496510089944 0.00135678547832 0.00497989612406 0.00136249766085 0.391772367825 0.39236554937 0.049215 0.0477212467098 0.003825 0.00467732699424 0.099 Chr3 116185509

N00018 12000000 12200000 1.683902 scaffold_14 5695812 5897301 0.00509701235803 0.00134854567138 0.00517419541364 0.00137211646795 0.392098106226 0.39194302625 0.084655 0.0714331799751 0.01857 0.0202442813255 0.078 Chr3 116185509

N00018 12200000 12400000 1.13658 scaffold_14 5503048 5695812 0.00423753308478 0.00124594120302 0.00425419772911 0.00125025350608 0.42566781548 0.425797849737 0.0555 0.0420773588429 0.003125 0.00326305741736 0.109 Chr3 116185509

N00018 12400000 12600000 1.13658 scaffold_14 5301740 5503048 0.00414444573773 0.00125055821116 0.00425343480016 0.00129580426328 0.38455197113 0.3800802236 0.063115 0.0380660480458 0.040615 0.0422486935442 0.078 Chr3 116185509

N00018 12600000 12800000 1.13658 scaffold_14 5105024 5301740 0.00428934905207 0.0013069023553 0.00431275879645 0.00131500092634 0.419652089617 0.418156533408 0.062355 0.0306482441693 0.013455 0.0143201366437 0.1305 Chr3 116185509

N00182 0 200000 1.372914 scaffold_14 4869107 5068708 0.00503303705878 0.00113742151636 0.00501316504915 0.00115076936188 0.386096245156 0.385390071694 0.05479 0.0495087699961 0.01468 0.014669265184 0.132333333333 Chr3 116185509

N00182 200000 400000 1.399028 scaffold_14 4661574 4869107 0.00442585856689 0.00147516829505 0.00442585856689 0.00147516829505 0.374478702181 0.376017417875 0.060735 0.0652715471756 0 0 NA Chr3 116185509

N00182 400000 600000 1.399028 scaffold_14 4468008 4661574 0.00497453239106 0.00132865636694 0.00504652132819 0.00135101224568 0.383690272704 0.383577953966 0.07115 0.0516619654278 0.017875 0.0186138061436 0.069 Chr3 116185509

N00182 600000 800000 1.399028 scaffold_14 4273788 4468008 0.00437516563284 0.00124823282748 0.00446592845087 0.00128909827481 0.383571699048 0.382947168945 0.12066 0.0687364843991 0.02974 0.033719493358 0.096 Chr3 116185509

N00182 800000 1000000 1.399028 scaffold_14 4077338 4273788 0.00361334560241 0.00094998767123 0.00361334560241 0.00094998767123 0.437348065118 0.440843722124 0.080635 0.045680834818 0 0 NA Chr3 116185509

N00182 1000000 1200000 1.399028 scaffold_14 3889230 4077338 0.00373370270311 0.00110560767112 0.00382409584826 0.00113815095481 0.409866256076 0.413057114084 0.087235 0.0376166882855 0.044245 0.0429646798648 0.07175 Chr3 116185509

N00206 200000 400000 0.8936722 scaffold_14 2938130 3139810 0.00428713146211 0.00120468660913 0.00428599042849 0.00120514929173 0.445714729324 0.448246233646 0.05114 0.0391263387545 3,00E-04 0.000242959143197 0.106 Chr3 116185509

N00206 400000 600000 0.8936722 scaffold_14 3139810 3330226 0.00399277023078 0.00116061145264 0.00415929566303 0.00122995852884 0.414282501535 0.415106991538 0.08844 0.0436202840097 0.06891 0.0732081337703 0.0808333333333 Chr3 116185509

N00206 600000 800000 0.8936722 scaffold_14 3330226 3529558 0.00354898499548 0.00108146779598 0.00357602717658 0.00109381316798 0.40356146975 0.403927931367 0.04522 0.0278329620934 0.011785 0.0115134549395 0.041 Chr3 116185509

N00206 800000 1000000 1.12498 scaffold_14 3529558 3732501 0.00401384489049 0.00115199662478 0.00407503514387 0.00118612725342 0.41625943037 0.416793075756 0.06403 0.0690735822374 0.0351 0.0336744800264 0.08875 Chr3 116185509

N00001 0 200000 2.224919 scaffold_1 23108417 23296483 0.0046119624361 0.0010418523368 0.00469617557696 0.00106536325994 0.402952554064 0.404292372249 0.06515 0.0435538587517 0.02607 0.0277243095509 0.1085 Chr3 116185509

N00001 200000 400000 2.417639 scaffold_1 22917969 23108417 0.00436278839495 0.00114691019481 0.00439284264792 0.00115325518008 0.391578302955 0.393357092583 0.09704 0.0666743678064 0.008595 0.00759262370831 0.0403333333333 Chr3 116185509

N00001 400000 600000 2.59384 scaffold_1 22714722 22917969 0.00416143434067 0.00111168350165 0.0043648153209 0.0011739848294 0.381075921197 0.379228096757 0.08961 0.0805030332551 0.065675 0.0643207525818 0.079 Chr3 116185509

N00001 600000 800000 2.59384 scaffold_1 22520049 22714722 0.00392427425891 0.00102527985395 0.00398932615244 0.00104910342389 0.403813713016 0.40448896267 0.085715 0.0745763408382 0.021615 0.0225968675677 0.051 Chr3 116185509

N00001 800000 1000000 2.59384 scaffold_1 22329598 22520049 0.00420788072524 0.00104544935818 0.00425310210424 0.0010642925053 0.413280833577 0.415768306059 0.074835 0.0489627253204 0.020995 0.0231450609343 0.07 Chr3 116185509

N00001 1000000 1200000 2.59384 scaffold_1 22137612 22329598 0.00510101832206 0.00115105070163 0.00514014454635 0.00116332439067 0.398211036649 0.398250526385 0.07251 0.059618930547 0.017825 0.0185742710406 0.095 Chr3 116185509

N00001 1200000 1400000 2.035195 scaffold_1 21937173 22137612 0.00511107671008 0.00110292185002 0.00511107671008 0.00110292185002 0.385348516559 0.386462991809 0.104395 0.0890196019737 0 0 NA Chr3 116185509

N00001 1400000 1600000 0.8925104 scaffold_1 21747675 21936170 0.00496819057301 0.001240973467 0.00496819057301 0.001240973467 0.371471111343 0.372223965127 0.082775 0.0816202021274 0 0 NA Chr3 116185509

N00001 1600000 1800000 0.8925104 scaffold_1 21557697 21747675 0.00472543913683 0.00111755852204 0.00472543913683 0.00111755852204 0.379074605049 0.37949214188 0.099075 0.0633494404615 0.0018 0 NA Chr3 116185509

N00001 1800000 2000000 0.8925104 scaffold_1 21364991 21557567 0.00415907233814 0.00115405607827 0.00418998075645 0.00116007861764 0.389961215584 0.392820597007 0.07809 0.0437074194084 0.008115 0.00715042372881 0.039 Chr3 116185509

N00001 2000000 2200000 0.8925104 scaffold_1 21167141 21364991 0.00429514956038 0.0011793665482 0.00431633788117 0.00118566855635 0.379381255101 0.377507964753 0.056275 0.0533939853424 0.005175 0.00524639878696 0.022 Chr3 116185509

N00001 2200000 2400000 0.8925104 scaffold_1 20964594 21166597 0.00461164119335 0.00140676426966 0.00461164119335 0.00140676426966 0.365388896309 0.364138461116 0.08897 0.0953451186369 0 0 0.022 Chr3 116185509

N00001 2400000 2600000 0.8925104 scaffold_1 20756091 20964594 0.00492815086403 0.00121217695234 0.00492815086403 0.00121217695234 0.361051334702 0.356112151623 0.100495 0.123072569699 0 0 NA Chr3 116185509

N00001 2600000 2800000 0.9208181 scaffold_1 20557731 20756091 0.00497435719651 0.00143278215504 0.00499795671365 0.00143901314108 0.369404618891 0.365037472578 0.089945 0.0956089937487 0.00566 0.0063369630974 0.168 Chr3 116185509

N00001 2800000 3000000 1.129587 scaffold_1 20358763 20557731 0.00450622398681 0.00105820222408 0.00454421707991 0.00106735703926 0.379140707177 0.373712729748 0.060615 0.0450725744843 0.021175 0.0213401149934 0.09175 Chr3 116185509

N00001 3200000 3400000 1.129587 scaffold_1 19982716 20179155 0.00453300312019 0.00110425070205 0.00458509692295 0.00113724026781 0.40526509853 0.408312638871 0.07903 0.0735902748436 0.03941 0.0382714226808 0.0925 Chr3 116185509

N00001 3400000 3600000 1.129587 scaffold_1 19793087 19982716 0.00422637285764 0.00122414827042 0.00427780807642 0.00125375163681 0.40232746574 0.401943219792 0.076475 0.0562888587716 0.02743 0.02771727953 0.065 Chr3 116185509

N00001 3600000 3800000 1.129587 scaffold_1 19586077 19791358 0.00422566263225 0.00125668475595 0.00423474356897 0.0012575687402 0.399135003213 0.399995609751 0.06282 0.0907731353608 0.002925 0.0177707630029 0.047 Chr3 116185509

N00001 3800000 4000000 1.129587 scaffold_1 19395525 19586077 0.0043999554084 0.00110352423235 0.0044376165944 0.00111516045164 0.394805735873 0.395041525619 0.051375 0.0391389227088 0.01357 0.0159326588018 0.0703333333333 Chr3 116185509

N00001 4000000 4200000 1.129587 scaffold_1 19207458 19395525 0.00452925205111 0.00114244889519 0.00465016823052 0.00117821501917 0.382198286829 0.38475704774 0.08584 0.0631370734898 0.03692 0.038784050365 0.0416666666667 Chr3 116185509

N00001 4200000 4400000 1.129587 scaffold_1 19016307 19207458 0.00455043995934 0.00127425367285 0.00457300033472 0.00128120592267 0.378930216878 0.380106620395 0.073425 0.0617940790265 0.00762 0.00710956259711 0.115 Chr3 116185509

N00001 4800000 5000000 1.129587 scaffold_1 18441948 18643409 0.00543505948228 0.00127004064507 0.00543505948228 0.00127004064507 0.370818945232 0.368406884601 0.10772 0.124560088553 0 0 NA Chr3 116185509

N00001 5000000 5200000 1.129587 scaffold_1 18245761 18441948 0.00466991953573 0.00126459778906 0.00473968838342 0.00129553512405 0.367166422678 0.367782412925 0.068435 0.0598357689347 0.02543 0.0258579824351 0.057 Chr3 116185509

N00001 5200000 5400000 1.129587 scaffold_1 18049547 18245674 0.00446865852959 0.00116552277611 0.00446865852959 0.00116552277611 0.3795099912 0.380503647767 0.062435 0.0497789697492 0 0 NA Chr3 116185509

N00001 5400000 5600000 1.129587 scaffold_1 17848125 18049547 0.00448806085522 0.00103972767814 0.00452314966349 0.00105428097184 0.385879945056 0.383726571605 0.087795 0.0724598107456 0.015515 0.0143430211198 0.0605 Chr3 116185509

N00001 5600000 5800000 1.129587 scaffold_1 17654025 17848125 0.00447868509273 0.00114144101943 0.00453645272284 0.00115568967851 0.396143782154 0.396980565518 0.086095 0.0414219474498 0.01442 0.0140597630088 0.0493333333333 Chr3 116185509

N00001 5800000 6000000 1.129587 scaffold_1 17466162 17654025 0.0050346355806 0.00124862677227 0.00503429936601 0.00125190081351 0.365190194811 0.366600272565 0.09331 0.0736973219846 0.00042 0.00383258012488 0.045 Chr3 116185509

N00001 6000000 6200000 1.129587 scaffold_1 17280148 17466162 0.00478366095219 0.00134905164178 0.00479198367798 0.00135146890114 0.365843330162 0.36607322358 0.102295 0.0598718376036 0.002365 0.00568236799381 0.084 Chr3 116185509

N00001 6200000 6400000 1.129587 scaffold_1 17077069 17279580 0.00411067890583 0.00125283645232 0.00411067890583 0.00125283645232 0.366189651116 0.363274497066 0.09183 0.0766131222501 0 0 NA Chr3 116185509

N00001 6400000 6600000 1.129587 scaffold_1 16889796 17077069 0.00442276379376 0.00125196599433 0.0044285474069 0.00125327713601 0.36029428857 0.358856160638 0.10294 0.0799794951755 0.001095 0.0011694157727 0.042 Chr3 116185509

N00001 6600000 6800000 1.129587 scaffold_1 16700282 16889796 0.00423070194659 0.000988638720963 0.00424261945258 0.000990914393373 0.393091505651 0.394174768032 0.092785 0.0602488470509 0.004555 0.00349842228015 0.0235 Chr3 116185509

N00001 6800000 7000000 1.129587 scaffold_1 16507753 16700282 0.00407937380326 0.00108750662464 0.00410546327093 0.00109595350546 0.394563492063 0.40397149891 0.06894 0.0684831895455 0.00875 0.00791569062323 0.082 Chr3 116185509

N00001 7000000 7200000 1.129587 scaffold_1 16313737 16507753 0.00403255702669 0.00113257187225 0.00404417684299 0.00113413264781 0.414938535984 0.415885916793 0.050085 0.0392338776183 0.006525 0.00733444664358 NA Chr3 116185509

N00001 7200000 7400000 1.129587 scaffold_1 16117829 16313737 0.00417182042452 0.000948386050746 0.00426295989001 0.000970555226826 0.402464608611 0.401443744797 0.07076 0.0830644996631 0.026125 0.0243889989179 0.115333333333 Chr3 116185509

N00001 7400000 7600000 1.129587 scaffold_1 15922628 16117829 0.00443334272019 0.00113969243479 0.00443334272019 0.00113969243479 0.389336256294 0.387413814447 0.070375 0.055906475889 0 0 0.233 Chr3 116185509

N00001 7600000 7800000 1.129587 scaffold_1 15732795 15922628 0.00409127058922 0.00102511364033 0.0041184672847 0.00103222572931 0.406388585385 0.406892792382 0.090485 0.0548218697487 0.011425 0.0118577907951 0.112333333333 Chr3 116185509

N00001 7800000 8000000 1.129587 scaffold_1 15534121 15732795 0.00458907662583 0.00125467025346 0.00462950409071 0.00128034748081 0.400771232266 0.399017071049 0.077745 0.0428641895769 0.016655 0.0292891873119 0.159 Chr3 116185509

N00001 8000000 8200000 0.6960363 scaffold_1 15338285 15534121 0.00413757554407 0.00119587765949 0.00419551547693 0.00121941950507 0.383027574438 0.380919986505 0.07116 0.0607855552605 0.024325 0.0258379460365 0.076 Chr3 116185509

N00001 8200000 8400000 0.6757136 scaffold_1 15146617 15337541 0.00451349260334 0.0013664386275 0.0045296678227 0.00136937179249 0.373283264765 0.372557418291 0.07042 0.0465630303157 0.00474 0.00497056420356 0.059 Chr3 116185509

N00001 8400000 8600000 0.6757136 scaffold_1 14955030 15146617 0.00421900460716 0.00120591848463 0.0042411583413 0.00121351064397 0.376739774615 0.376090136567 0.093225 0.0504366162631 0.00603 0.00762577836701 0.092 Chr3 116185509

N00001 8600000 8800000 0.6757136 scaffold_1 14762462 14955030 0.00421851008839 0.00109480617931 0.00425061204347 0.00110333065199 0.359168916702 0.358216628157 0.094335 0.084951809231 0.01171 0.0117568858793 NA Chr3 116185509

N00001 8800000 9000000 0.6757136 scaffold_1 14565393 14762462 0.00440008974856 0.00106856437137 0.00448897290437 0.00110063546544 0.375485721592 0.375652164422 0.052515 0.044106378984 0.031255 0.0430813572911 0.024 Chr3 116185509

N00001 9200000 9400000 0.6757136 scaffold_1 14189848 14386149 0.00465998942783 0.00120272047674 0.00470768373095 0.00121921814366 0.381891993958 0.381918522422 0.081955 0.0586853862181 0.016235 0.0167956352744 0.0763333333333 Chr3 116185509

N00001 9400000 9600000 0.6757136 scaffold_1 13991330 14189848 0.00438769859117 0.00103831847961 0.00443371333646 0.00105109509839 0.375765347189 0.374698234812 0.081955 0.0684723803383 0.01509 0.0173334407963 0.045 Chr3 116185509

N00001 9600000 9800000 0.6757136 scaffold_1 13793474 13991330 0.00413322198854 0.00112830210043 0.00415050732674 0.00114338667002 0.393874267196 0.392613403767 0.09 0.0688783761928 0.01716 0.018700469028 0.063 Chr3 116185509

N00001 9800000 10000000 0.6757136 scaffold_1 13597028 13793474 0.00410981053422 0.00112973001866 0.00425375940818 0.00118142093713 0.379796116389 0.381771548638 0.097535 0.0860592732863 0.050535 0.051082740295 0.078 Chr3 116185509

N00001 10000000 10200000 2.144198 scaffold_1 13400870 13597028 0.00403026313312 0.00120724452354 0.00403921380944 0.00121194433674 0.393921173514 0.395427112349 0.047655 0.0444947440329 0.004465 0.00456774640851 NA Chr3 116185509

N00001 10400000 10600000 6.042357 scaffold_1 13030618 13227453 0.0043585232931 0.00106444259786 0.0043585232931 0.00106444259786 0.396971834808 0.399786672355 0.06209 0.060471968908 0 0 NA Chr3 116185509

N00001 10600000 10800000 2.675472 scaffold_1 12836370 13030618 0.00399099668732 0.00123648909071 0.0040242184176 0.00124509869338 0.381546637047 0.386352946265 0.06529 0.0320724022899 0.00945 0.0103578930028 0.034 Chr3 116185509

N00001 11000000 11200000 1.673291 scaffold_1 12462179 12661624 0.00433633996217 0.00138943236531 0.00433633996217 0.00139007265526 0.407885040531 0.410045721187 0.072575 0.0450349720474 0 0.00181002281331 NA Chr3 116185509

N00001 11200000 11400000 2.850267 scaffold_1 12270974 12462179 0.00453154356872 0.00132740878272 0.00457086130509 0.00134485259343 0.39951789968 0.400576179109 0.073605 0.0631782641667 0.01487 0.0161397452995 0.066 Chr3 116185509

N00001 11400000 11600000 2.484688 scaffold_1 12076559 12270974 0.00373683300655 0.000960167951193 0.00376142543965 0.000981029721009 0.379571212586 0.382291231847 0.07639 0.0514672221794 0.01324 0.0265565928555 0.0586666666667 Chr3 116185509

N00001 11600000 11800000 2.33998 scaffold_1 11873030 12076559 0.00493181318195 0.000980243328254 0.0051886551709 0.00103989270439 0.405951401367 0.404064774444 0.05374 0.0318185614826 0.07583 0.0855308088774 0.112 Chr3 116185509

N00001 11800000 12000000 2.33998 scaffold_1 11679244 11873030 0.00417625753745 0.00116789737298 0.00432693958997 0.00121220284643 0.418699919232 0.419236609844 0.087135 0.04187609012 0.037035 0.0415200272466 0.045 Chr3 116185509

N00001 12000000 12200000 2.33998 scaffold_1 11480047 11679244 0.00454482695708 0.00113928946752 0.00465227976019 0.00116598876211 0.420982054822 0.419709112496 0.059695 0.0636154158948 0.03691 0.0382736687802 0.0426666666667 Chr3 116185509

N00001 12200000 12400000 2.33998 scaffold_1 11284703 11480047 0.00410500964687 0.00106524872761 0.0041914223452 0.00109361668587 0.395097415737 0.393529508878 0.08145 0.0659247276599 0.03082 0.0306894504054 0.0722 Chr3 116185509

N00001 12400000 12600000 1.118572 scaffold_1 11086155 11284591 0.00429470349627 0.00122148729062 0.00429822729054 0.00122647485897 0.40180878553 0.403975188069 0.04799 0.0283164345179 0.00472 0.00525106331512 0.0555 Chr3 116185509

N00001 12600000 12800000 0.5988235 scaffold_1 10902053 11086155 0.00445543927287 0.00102326853928 0.00455803651236 0.00105913342758 0.394105341621 0.394448733829 0.057775 0.0551705033079 0.036385 0.0400484514019 0.104 Chr3 116185509

N00001 12800000 13000000 0.5988235 scaffold_1 10702604 10902053 0.00407421562008 0.00103259230044 0.00420722538362 0.00108540995771 0.403739012009 0.404047929119 0.05871 0.0248735265657 0.061515 0.0603161710512 0.0703333333333 Chr3 116185509

N00001 13000000 13200000 0.5988235 scaffold_1 10504565 10702604 0.00414527129665 0.00117962490458 0.00416466426375 0.0011859666202 0.37336087361 0.371283636241 0.097345 0.078711768894 0.009525 0.00942238649963 0.092 Chr3 116185509

N00001 13200000 13400000 0.5988235 scaffold_1 10309680 10503971 0.00424925448408 0.00109656326237 0.00454309101247 0.00120061891528 0.365137073794 0.363776468226 0.05762 0.0445826106202 0.09579 0.117334307817 NA Chr3 116185509

N00001 13400000 13600000 0.5988235 scaffold_1 10113276 10309680 0.00468820592006 0.00131917353018 0.00475281599133 0.00134538753389 0.373418941317 0.378600733001 0.063105 0.041063318466 0.022835 0.0244750616077 0.1195 Chr3 116185509

N00001 13800000 14000000 0.5988235 scaffold_1 9707002 9906954 0.00480927462131 0.00135208199539 0.00480927462131 0.00135208199539 0.364228426368 0.36408407352 0.090245 0.0976934464271 0 0 NA Chr3 116185509

N00001 14200000 14400000 2.977507 scaffold_1 9286317 9481118 0.0047229660816 0.0012067333777 0.00475103052075 0.00121717480861 0.398759865332 0.401817106607 0.062565 0.0397328555808 0.007125 0.00887059101339 NA Chr3 116185509

N00001 14400000 14600000 1.078962 scaffold_1 9093798 9286317 0.00389913579197 0.00107450044852 0.00398295845368 0.00110184576723 0.393283262287 0.395588980244 0.07651 0.0474031134589 0.025225 0.0270362925218 0.078 Chr3 116185509

N00001 14600000 14800000 1.078962 scaffold_1 8901291 9093798 0.00441494128544 0.00123691710728 0.00450736649083 0.00126788587376 0.384652335775 0.385337795205 0.07868 0.0496657264411 0.033775 0.0384609390827 0.061 Chr3 116185509

N00001 14800000 15000000 1.078962 scaffold_1 8704852 8901291 0.00407655173229 0.00102895162754 0.00407655173229 0.00102895162754 0.390462823944 0.390638855896 0.063805 0.0620803404619 0 0 NA Chr3 116185509

N00001 15000000 15200000 1.078962 scaffold_1 8509119 8704852 0.00351700871022 0.000910360406282 0.00351700871022 0.000910360406282 0.407317484771 0.40791262239 0.058035 0.0614408403284 0 0 NA Chr3 116185509

N00001 15200000 15400000 1.078962 scaffold_1 8323213 8509119 0.00316593046171 0.000888501726829 0.00316997685347 0.000889780394835 0.395641235766 0.395784968478 0.07808 0.0318063967812 0.00291 0.00238830376642 0.026 Chr3 116185509

N00001 15400000 15600000 1.078962 scaffold_1 8129670 8323213 0.00287824002073 0.000804965676355 0.00289227923357 0.000809570444957 0.395499356584 0.397061735663 0.05925 0.0372268694812 0.006525 0.00674268767147 0.026 Chr3 116185509

N00001 15600000 15800000 1.078962 scaffold_1 7935444 8129670 0.00419351040109 0.00107723032452 0.00424866194265 0.00109602265962 0.382841692068 0.381876379691 0.072285 0.032106926982 0.02188 0.0237455335537 0.0463333333333 Chr3 116185509

N00001 15800000 16000000 1.078962 scaffold_1 7736544 7935444 0.00438923213252 0.00100263994099 0.00448253847825 0.00102540979422 0.38720018225 0.383971736705 0.064295 0.0425691302162 0.02191 0.0217094017094 0.0603333333333 Chr3 116185509

N00001 16000000 16200000 5.543408 scaffold_1 7535717 7736544 0.00391360670464 0.000896574612036 0.00399800528931 0.000917045217163 0.382637217501 0.381825512103 0.05782 0.0529361091885 0.02966 0.0295976138667 0.0536666666667 Chr3 116185509

N00001 16200000 16400000 0.7210973 scaffold_1 7333860 7535717 0.00392985997991 0.00101525020166 0.00399238943361 0.00104672164937 0.406981973229 0.404807316937 0.06608 0.0439816305602 0.037285 0.0411380333603 0.102666666667 Chr3 116185509

N00001 16400000 16600000 0.7210973 scaffold_1 7135791 7333860 0.00421589007464 0.00101364557696 0.00432720492849 0.0010463303456 0.4135385098 0.412465845861 0.049465 0.0472663566737 0.045755 0.0444945953178 0.0828 Chr3 116185509

N00001 16600000 16800000 0.7210973 scaffold_1 6945679 7135791 0.00377213546324 0.000954436083378 0.0038120613279 0.000964577622799 0.412418308056 0.412890863741 0.075615 0.0450944706278 0.015135 0.0181419373843 0.059 Chr3 116185509

N00001 16800000 17000000 0.7210973 scaffold_1 6752787 6945679 0.00368617253005 0.000925014283453 0.00369081164658 0.000926259480666 0.398563121995 0.39974668957 0.075435 0.0489496713187 0.002425 0.001897434834 0.023 Chr3 116185509

N00001 17000000 17200000 0.7210973 scaffold_1 6556805 6752787 0.00395363542409 0.000972807642052 0.00409661425864 0.00101909009992 0.398271945389 0.400469379772 0.067985 0.043555020359 0.05 0.0524282842302 0.0835 Chr3 116185509

N00001 17600000 17800000 0.9037675 scaffold_1 5986511 6182546 0.00471318477539 0.00138390819309 0.00471318477539 0.00138390819309 0.356965525024 0.360453106406 0.10911 0.0945341393119 0 0 NA Chr3 116185509

N00001 17800000 18000000 0.9658754 scaffold_1 5790361 5986511 0.00453084721257 0.00127456295836 0.00453084721257 0.00127456295836 0.372206627935 0.372524265035 0.073255 0.0616568952332 0 0 NA Chr3 116185509

N00001 18000000 18200000 0.9658754 scaffold_1 5598749 5790361 0.00415237374719 0.00100913985315 0.00415237374719 0.00100913985315 0.384067677511 0.386291210691 0.05897 0.0493392898148 0.002165 0 NA Chr3 116185509

N00001 18200000 18400000 0.9493937 scaffold_1 5398025 5598749 0.00360118469686 0.00103106033613 0.00362240943118 0.00104280017702 0.401010832684 0.402877177763 0.08027 0.0548265279688 0.02072 0.0169586098324 0.072 Chr3 116185509

N00001 18400000 18600000 0.9222857 scaffold_1 5200259 5398025 0.00423026806978 0.00117105757779 0.00429540943877 0.00118553485323 0.389082658164 0.390032172177 0.06966 0.0337520099512 0.01519 0.0170403406046 0.0556666666667 Chr3 116185509

N00001 18600000 18800000 0.9222857 scaffold_1 4989595 5200259 0.00402810155641 0.00117080912405 0.00415452604362 0.00122201897821 0.407150827095 0.409314079422 0.04646 0.0630482664338 0.0534 0.0627681995975 0.0595 Chr3 116185509

N00001 18800000 19000000 0.9222857 scaffold_1 4792692 4989595 0.00432845202065 0.00116429255771 0.00438213937034 0.00118341133311 0.413474468928 0.415193126245 0.094205 0.0549052071324 0.022405 0.0212185695495 0.08 Chr3 116185509

N00001 19000000 19200000 0.9496842 scaffold_1 4588424 4792692 0.00408190408793 0.00104057283666 0.00408652102278 0.00104249343928 0.428670517725 0.433288577254 0.08978 0.0555153034249 0.005195 0.00420525975679 0.115 Chr3 116185509

N00001 19200000 19400000 6.429379 scaffold_1 4395022 4588424 0.00446264483299 0.00107772151279 0.00451055930166 0.00110443273409 0.394007996322 0.396823237305 0.089435 0.0634481546209 0.02674 0.0285415869536 0.073 Chr3 116185509

N00001 19400000 19600000 5.062463 scaffold_1 4196646 4395022 0.00427730696651 0.00105109028588 0.00436930149475 0.00108081601702 0.385986893898 0.387717747328 0.086345 0.0492146227366 0.03119 0.0366929467274 0.102666666667 Chr3 116185509

N00001 19600000 19800000 0.5836322 scaffold_1 4002015 4196646 0.00334700508526 0.000942375859175 0.00342255627715 0.000966716294003 0.387635407082 0.388307388024 0.073955 0.0464828316147 0.02666 0.0307864625882 0.053 Chr3 116185509

N00001 19800000 20000000 0.5836322 scaffold_1 3798831 4002015 0.00415252435726 0.00114359407892 0.00415252435726 0.00114367085787 0.375923106402 0.37610184008 0.058685 0.0495363808174 0.00129 0.00010335459485 NA Chr3 116185509

N00001 20000000 20200000 0.5836322 scaffold_1 3604609 3798424 0.00427533795859 0.00120764420554 0.00437770272473 0.00124195390082 0.395051907243 0.399270973853 0.09619 0.0505120862678 0.031965 0.0329850630756 0.063 Chr3 116185509

N00001 20200000 20400000 0.5836322 scaffold_1 3394426 3604609 0.00487239024796 0.00129129797663 0.00493102128844 0.00131356249712 0.369728907927 0.367614331964 0.060455 0.0545286726329 0.02062 0.0188264512354 NA Chr3 116185509

N00001 20400000 20600000 0.5836322 scaffold_1 3182980 3394426 0.00516316822065 0.00141029498794 0.00517597499133 0.00141029498794 0.386253396397 0.387530335134 0.05523 0.0515261579789 0.0032 0 NA Chr3 116185509

N00001 20600000 20800000 0.5836322 scaffold_1 2975777 3182978 0.00402234368897 0.000982971873185 0.0041549889408 0.0010156243002 0.381083821541 0.383838383838 0.10028 0.0799561778177 0.04435 0.042688017915 0.08275 Chr3 116185509

N00001 20800000 21000000 0.5836322 scaffold_1 2764877 2975658 0.00410164509843 0.0011802579886 0.00412578236844 0.00118850390817 0.386594085751 0.380265092485 0.07772 0.0880060346995 0.01425 0.0135353755794 0.058 Chr3 116185509

N00001 21000000 21200000 0.5836322 scaffold_1 2566847 2764877 0.0042765465761 0.00118423256471 0.00432203145659 0.00119682271523 0.376205128205 0.376067501918 0.094105 0.0826389940918 0.02627 0.0264858859769 0.062 Chr3 116185509

N00001 21200000 21400000 0.5836322 scaffold_1 2370793 2566847 0.00400548118705 0.00114514644677 0.00406841422615 0.00116035730771 0.371073772441 0.369929620412 0.07268 0.0547145174289 0.01723 0.014842849419 0.051 Chr3 116185509

N00001 21400000 21600000 0.5836322 scaffold_1 2171608 2370793 0.0044592497623 0.00131192026084 0.0044592497623 0.00131192026084 0.377508685177 0.374144341083 0.058745 0.0409920425735 0.001185 0 NA Chr3 116185509

N00001 21600000 21800000 0.5836322 scaffold_1 1967947 2171608 0.0044599271658 0.00115602516113 0.0044599271658 0.00115619149418 0.377498425978 0.377746990515 0.083315 0.0725028355944 0 0.000220955411198 NA Chr3 116185509

N00001 21800000 22000000 0.5836322 scaffold_1 1782658 1967947 0.00411795836119 0.00111792699546 0.00418126361516 0.00114029208301 0.408822832849 0.407106288258 0.063605 0.040336987085 0.017395 0.0220358467043 0.049 Chr3 116185509

N00001 22000000 22200000 0.5836322 scaffold_1 1577859 1782658 0.00424076086061 0.00102441249599 0.00426059311451 0.0010304726798 0.399123545321 0.398078103758 0.079945 0.0905668484709 0.00463 0.00483400797855 0.043 Chr3 116185509

N00001 22200000 22400000 0.5836322 scaffold_1 1389350 1577859 0.00379509056377 0.00117121379657 0.00385247306438 0.00119633686015 0.399247728719 0.399057739144 0.093735 0.0411014858707 0.023585 0.0320515200866 0.017 Chr3 116185509

N00001 22400000 22600000 0.5836322 scaffold_1 1182287 1389350 0.00433467979443 0.0011279589606 0.00440081735694 0.00115125675178 0.375824199349 0.376070144438 0.06494 0.0493231528569 0.03704 0.0356461560008 0.0633333333333 Chr3 116185509

N00001 22600000 22800000 0.4619926 scaffold_1 986024 1182287 0.00450113993213 0.00114779959316 0.00451874979584 0.001149766883 0.361592324831 0.360163120371 0.083575 0.0897520164269 0.004285 0.0048251580787 0.047 Chr3 116185509

N00001 22800000 23000000 0.4478013 scaffold_1 798056 986024 0.00351659747068 0.00105917836507 0.00353570899466 0.00106962964817 0.379221207163 0.380750864482 0.07635 0.0432839632278 0.010225 0.0132628958121 0.1205 Chr3 116185509

N00001 23000000 23200000 0.4478013 scaffold_1 605510 798056 0.00451002861459 0.00132267491628 0.00454052753186 0.00134950158346 0.366584152896 0.371943529462 0.047325 0.0352383326582 0.01487 0.0314210630187 0.078 Chr3 116185509

N00001 23200000 23400000 0.4478013 scaffold_1 415378 605510 0.00466023310287 0.00119965358759 0.00466023310287 0.00120065268332 0.37105715178 0.372455340828 0.06692 0.0561872804157 0 0.000720552037532 NA Chr3 116185509

N00001 23400000 23600000 0.4478013 scaffold_1 216955 415378 0.0047138527526 0.00121432335043 0.0047138527526 0.00121432335043 0.370804269914 0.372631297757 0.090385 0.0790079779058 0 0 NA Chr3 116185509

N00001 23600000 23800000 0.4478013 scaffold_1 1206 216955 0.0043535077969 0.00092720509612 0.0043535077969 0.00092720509612 0.36600728027 0.368085914194 0.078005 0.0770617708541 0 0 NA Chr3 116185509

N00001 23800000 24000000 0.4478013 scaffold_14 4990 204880 0.00449349296809 0.000735858507802 0.00459182754817 0.000758297455188 0.366089231265 0.363316599331 0.05484 0.0708789834409 0.026665 0.0265896242934 0.057 Chr3 116185509

N00001 24000000 24200000 0.4478013 scaffold_14 204880 398960 0.00451882042246 0.00121382594149 0.00470002020507 0.00126667513405 0.347312371717 0.348910350811 0.07724 0.0859851607585 0.04655 0.0479544517725 0.057 Chr3 116185509

N00001 24200000 24400000 0.4478013 scaffold_14 398960 597367 0.00378052873814 0.00111649688832 0.00384545817597 0.0011371564594 0.372165670275 0.371669919032 0.053295 0.0393736108101 0.01911 0.0197775280106 0.053 Chr3 116185509

N00001 24400000 24600000 0.4478013 scaffold_14 597367 799185 0.00325206351722 0.00130105503732 0.00338756905497 0.00136778263909 0.385951717157 0.383087023166 0.073945 0.0542568056368 0.058535 0.0618032088317 0.0678333333333 Chr3 116185509

N00001 24600000 24800000 0.4478013 scaffold_14 799185 996455 0.00386059407145 0.00117200446858 0.00401104185388 0.00121839405297 0.395035883502 0.392925087849 0.05791 0.0333198154813 0.04849 0.0507071526334 0.053 Chr3 116185509

N00001 24800000 25000000 0.4478013 scaffold_14 996455 1190751 0.00327035948093 0.00105843336156 0.00342773267986 0.00112433031368 0.372072196611 0.376842274814 0.072165 0.060243134187 0.058665 0.0612570510973 0.0556666666667 Chr3 116185509

N00001 25000000 25200000 0.4478013 scaffold_14 1190751 1385920 0.00334991459982 0.000897207735061 0.00340997554575 0.000923165037407 0.43396709244 0.434520591934 0.04296 0.0241944161214 0.0233 0.0273404075442 0.0693333333333 Chr3 116185509

N00001 25200000 25400000 0.4478013 scaffold_14 1385920 1589193 0.00373403574636 0.00106630959878 0.00380011599486 0.00108836958251 0.444186955102 0.444470203793 0.06441 0.0317356461507 0.02995 0.024356407393 0.054 Chr3 116185509

N00001 25400000 25600000 0.4500195 scaffold_14 1589193 1789247 0.00391785472792 0.00100320912877 0.00393409175604 0.00100714360515 0.400353547805 0.395660286826 0.0558 0.0328061423416 0.005265 0.00557349515631 0.034 Chr3 116185509

N00001 25600000 25800000 0.8936722 scaffold_14 1789247 1988317 0.00388535684372 0.00134463676175 0.00393894773225 0.00137335429807 0.396997935383 0.39223010833 0.0773 0.060541518059 0.02585 0.0232430803235 0.051 Chr3 116185509

N00001 25800000 26000000 0.8936722 scaffold_14 1988317 2181384 0.0041162662181 0.00110564220279 0.00417686304341 0.00112494530357 0.394012719271 0.390406628511 0.07452 0.0601449237829 0.02391 0.0247893218416 0.0435 Chr3 116185509

N00001 26000000 26200000 0.8936722 scaffold_14 2181384 2375486 0.00348792713082 0.00108384040652 0.00354889215751 0.00111236232706 0.376059592685 0.375247910864 0.069975 0.0481757014353 0.032765 0.0322150209684 0.066 Chr3 116185509

N00001 26200000 26400000 0.8936722 scaffold_14 2375486 2574379 0.0041812786907 0.00125244680329 0.00418547646621 0.00125413563242 0.428193794652 0.427647266389 0.06275 0.0636472877376 0.00202 0.00221224477483 0.066 Chr3 116185509

N00001 26400000 26600000 0.8936722 scaffold_14 2574379 2769309 0.0045273730853 0.00110275729217 0.00453906708674 0.00111121615464 0.389153416041 0.38785941114 0.060335 0.0476478735956 0.012365 0.0128558969887 NA Chr3 116185509

N00108 200000 400000 1.518975 scaffold_1 25726055 25923012 0.00456687014702 0.00115706047474 0.00462139733539 0.00117334164566 0.384382515683 0.383226143165 0.066435 0.0718735561569 0.01363 0.0138456617434 0.044 Chr3 116185509

N00108 600000 800000 1.518975 scaffold_1 25351730 25545022 0.00451437020313 0.001120721584 0.00454578259344 0.00113090464841 0.398997915619 0.397192476776 0.091115 0.0652949941022 0.00966 0.0108333505784 0.028 Chr3 116185509

N00108 800000 1000000 2.601608 scaffold_1 25161800 25351730 0.00354372729648 0.00115720710144 0.00354372729648 0.00115720710144 0.409501685443 0.411808414282 0.078115 0.046880429632 0 0 NA Chr3 116185509

N00108 1000000 1200000 4.308837 scaffold_1 24974420 25161800 0.00350690571332 0.000923537530481 0.0035378073483 0.000934779507114 0.406808647395 0.411952033486 0.094095 0.0353879816416 0.0146 0.0150442950155 0.033 Chr3 116185509

N00108 1200000 1400000 4.308837 scaffold_1 24779994 24974420 0.00431052170445 0.00130671627713 0.00441232149248 0.00135225715463 0.385437470281 0.386304519816 0.088525 0.0732823799286 0.03271 0.0442070504974 0.0485 Chr3 116185509

N00108 1400000 1600000 4.308837 scaffold_1 24583681 24779994 0.00407048920347 0.00106602418305 0.00413604158896 0.00108462634954 0.396408747409 0.396464660462 0.071405 0.0464920815229 0.019845 0.0202839343294 0.0563333333333 Chr3 116185509

N00108 1600000 1800000 4.308837 scaffold_1 24387721 24583681 0.00364503834097 0.000938026760708 0.00366682249886 0.000942271189572 0.410011821554 0.410368403089 0.047755 0.0383802816901 0.014885 0.0138803837518 0.0485 Chr3 116185509

N00108 1800000 2000000 4.277734 scaffold_1 24193421 24387721 0.00452895528678 0.0010574278265 0.00458305018636 0.00107526270256 0.423269644367 0.422332570104 0.0897 0.0693618116315 0.017155 0.0176170869789 0.1055 Chr3 116185509

N00108 2200000 2400000 2.224919 scaffold_1 23819497 24013672 0.00370201469014 0.000972031698156 0.00370767778587 0.000977224742665 0.428868327402 0.429199820688 0.100195 0.0686133642333 0.00714 0.00732329084589 0.046 Chr3 116185509

N00108 2400000 2600000 2.224919 scaffold_1 23636283 23819497 0.00403887704009 0.00102286529567 0.00410833636481 0.0010491224735 0.388592573234 0.387778724148 0.059645 0.043599288264 0.024575 0.029610182628 0.1155 Chr3 116185509

N00108 2600000 2800000 2.224919 scaffold_1 23438645 23636283 0.00449162499837 0.00105000897667 0.00455850609538 0.0010861861627 0.386235126584 0.380750334971 0.099755 0.0921887491272 0.02659 0.0523836509173 0.0845 Chr3 116185509

N00153 0 200000 1.518975 scaffold_1 27576268 27770917 0.00385386633626 0.000966577196808 0.00393183915229 0.00099367476205 0.363000192654 0.363848211654 0.072985 0.0470179656715 0.028 0.0295249397634 0.059 Chr3 116185509

N00153 400000 600000 1.518975 scaffold_1 27197582 27387581 0.00534615518603 0.00127961814509 0.00535805179037 0.00128241228342 0.356052077019 0.355865856832 0.090945 0.0827109616366 0.002135 0.0029473839336 0.03 Chr3 116185509

N00153 800000 1000000 1.518975 scaffold_1 26811812 27010536 0.00522888455123 0.00146706040124 0.0052460673002 0.00147198720856 0.342142126117 0.34212068292 0.12601 0.115315714257 0.00319 0.00456411907973 0.03 Chr3 116185509

N00153 1400000 1600000 1.518975 scaffold_1 26214193 26413723 0.00531248276076 0.00129605004711 0.00531248276076 0.00129605004711 0.352363646852 0.350350056665 0.13075 0.12624166792 0 0 NA Chr3 116185509

N00121 0 200000 1.518975 scaffold_1 27771357 27964940 0.00475630143375 0.00108250001687 0.00476320392556 0.00108815873515 0.37156134139 0.374746360236 0.064995 0.0603513738293 0.00567 0.0103159884907 0.094 Chr3 116185509

N00121 200000 400000 1.370508 scaffold_1 27964940 28173498 0.00507675997586 0.0013276171721 0.00507675997586 0.0013276171721 0.383755859159 0.379042125974 0.07239 0.08856529119 0 0 NA Chr3 116185509

N00121 400000 600000 0.1625324 scaffold_1 28174377 28367936 0.00373541873228 0.00105251738454 0.00386390263947 0.00109236629929 0.408248573481 0.409922667006 0.098235 0.0589845990112 0.036945 0.0406904354745 0.0393333333333 Chr3 116185509

N00121 600000 800000 0.1625324 scaffold_1 28367936 28572476 0.0038431303666 0.0010064471614 0.0038668038058 0.001013647824 0.403302740572 0.406932337965 0.063535 0.0597829275447 0.0093 0.00910824288648 0.047 Chr3 116185509

N00121 800000 1000000 0.1625324 scaffold_1 28572476 28760464 0.00365436319716 0.000981495176834 0.00365734988578 0.000981495176834 0.413883844401 0.42203227926 0.09328 0.0344543268719 0.00087 0 NA Chr3 116185509

N00121 1000000 1200000 0.1625324 scaffold_1 28760464 28959749 0.00361785538513 0.00089312042624 0.00361785538513 0.00089312042624 0.376493527356 0.378107486124 0.076585 0.0901422585744 0 0 NA Chr3 116185509

N00121 1200000 1400000 0.1625324 scaffold_1 28959762 29146764 0.00349344841849 0.00106413726754 0.00349344841849 0.00106413726754 0.369877830785 0.369392836621 0.086325 0.060662452808 0 0 NA Chr3 116185509

N00121 1400000 1600000 0.1625324 scaffold_1 29146802 29347406 0.00369468929519 0.0010644564169 0.003706584021 0.00107456966968 0.359848464112 0.355323659792 0.08174 0.0767880999382 0.010285 0.0102540328209 0.081 Chr3 116185509

N00121 1600000 1800000 0.1625324 scaffold_1 29348054 29544859 0.00344067557522 0.000957801870292 0.0034752293597 0.000976481032438 0.377665676879 0.376717563735 0.094615 0.0665023754478 0.02045 0.0207515052971 0.174666666667 Chr3 116185509

N00121 1800000 2000000 0.1625324 scaffold_1 29544859 29744569 0.00352810297151 0.0013611418788 0.00356843645724 0.00138884412505 0.376127735973 0.373404766965 0.08486 0.0794251664914 0.021055 0.0257923989785 0.0626666666667 Chr3 116185509

N00121 2000000 2200000 0.1625324 scaffold_1 29744569 29946541 0.00283759054084 0.00131606440669 0.00284465556531 0.00132150381879 0.366914006638 0.366681179753 0.063785 0.0563791020538 0.005385 0.00571861446141 NA Chr3 116185509

N00035 200000 400000 0.9442971 scaffold_1 37896105 38088968 0.00483493874218 0.00122180765883 0.00502081099887 0.00128169870793 0.373034807989 0.37854659474 0.104015 0.0882336166087 0.04604 0.0486511150402 0.056 Chr3 116185509

N00035 400000 600000 0.9939395 scaffold_1 37711523 37896105 0.00422583600802 0.00104333506931 0.00429999115052 0.00106679842341 0.391413288789 0.392378894502 0.09937 0.0494089347824 0.02669 0.0270286376786 0.07625 Chr3 116185509

N00035 600000 800000 1.851399 scaffold_1 37518057 37711523 0.00412171265878 0.00122508327189 0.00424273786006 0.00128043319703 0.364604286606 0.361899447575 0.06073 0.0567748338209 0.041245 0.0467627386724 0.085 Chr3 116185509

N00035 800000 1000000 1.851399 scaffold_1 37319606 37518057 0.00498368955553 0.00110020565703 0.00500304049946 0.00110957979262 0.37046647029 0.373691993363 0.1013 0.110536102111 0.008615 0.00857642440703 0.166 Chr3 116185509

N00035 1000000 1200000 1.851399 scaffold_1 37122102 37319606 0.00459137947303 0.00108867849401 0.00459137947303 0.00108928584787 0.384514886126 0.387373975107 0.079565 0.0717555087492 0.005655 0.00641505994815 0.013 Chr3 116185509

N00035 1200000 1400000 1.851399 scaffold_1 36928982 37122102 0.00496532015776 0.00116097951307 0.00499000949885 0.00116944268843 0.369980038972 0.368473637468 0.122535 0.0995495028998 0.005545 0.00870961060481 NA Chr3 116185509

N00035 1400000 1600000 1.851399 scaffold_1 36726832 36928982 0.00474944606003 0.00118814275671 0.00474944606003 0.00118814275671 0.386034093013 0.384449703215 0.10587 0.115389562206 0 0 NA Chr3 116185509

N00035 1600000 1800000 1.851399 scaffold_1 36530734 36726832 0.00392448070059 0.000968141106553 0.00402388481403 0.000995527458708 0.368704148058 0.368281331001 0.092285 0.0658293302328 0.0305 0.0278432212465 0.073 Chr3 116185509

N00035 1800000 2000000 1.851399 scaffold_1 36337832 36530734 0.0046499644545 0.00105840059682 0.00469975294144 0.00106790813852 0.395944261924 0.393926503734 0.08156 0.0601756332231 0.013285 0.0123015831873 0.0303333333333 Chr3 116185509

N00035 2200000 2400000 1.851399 scaffold_1 35938668 36150117 0.00459863649864 0.00122277643826 0.00460543093335 0.00122876795811 0.380901991492 0.381207314269 0.0609 0.0947840850512 0.004165 0.00395367204385 0.063 Chr3 116185509

N00035 2400000 2600000 1.960886 scaffold_1 35740336 35938668 0.00471999893535 0.00120007259551 0.00471999893535 0.00120007259551 0.379517423233 0.379455433308 0.089665 0.104627594135 0 0 NA Chr3 116185509

N00035 2600000 2800000 2.329806 scaffold_1 35547713 35740336 0.00476644637781 0.00130339844319 0.00476644637781 0.00130339844319 0.379936147026 0.381286872374 0.05699 0.0507260296019 0 0 NA Chr3 116185509

N00035 2800000 3000000 2.329806 scaffold_1 35353423 35547713 0.0044677646467 0.0010082478566 0.00448406675659 0.00101015784276 0.398758925536 0.402924361132 0.09674 0.0751042256421 0.006135 0.00503371249164 NA Chr3 116185509

N00035 3000000 3200000 2.329806 scaffold_1 35157712 35353423 0.00465901004424 0.000993981809985 0.00473091588657 0.00101605750226 0.384809056712 0.386831684325 0.117345 0.097516235674 0.026375 0.0238974814906 0.0535 Chr3 116185509

N00035 3200000 3400000 2.329806 scaffold_1 34960444 35157712 0.00480784605234 0.00134304662661 0.00485050289556 0.00135752688595 0.387387488245 0.387408308249 0.07404 0.0764138126812 0.01077 0.0109445018959 0.048 Chr3 116185509

N00035 3400000 3600000 2.329806 scaffold_1 34765602 34960444 0.00469421505084 0.00107762475338 0.00487153102966 0.00112650641855 0.374115197376 0.369899476143 0.06383 0.0575337966147 0.053125 0.054505702056 0.0705 Chr3 116185509

N00035 3600000 3800000 2.329806 scaffold_1 34578321 34764143 0.00475527395759 0.00113406744974 0.00482063895742 0.00115010747047 0.39390066941 0.394370885212 0.09473 0.0552087481568 0.02058 0.021612080378 0.082 Chr3 116185509

N00035 3800000 4000000 2.329806 scaffold_1 34382043 34578321 0.00461816368602 0.00116844813634 0.00468462483064 0.00120363315039 0.389002597812 0.391435637552 0.064825 0.0530064500352 0.02938 0.0333098971866 0.092 Chr3 116185509

N00035 4000000 4200000 2.329806 scaffold_1 34185852 34382043 0.00403414704367 0.00104366234401 0.00404779747534 0.00104989250948 0.405153454784 0.409885659451 0.06976 0.0374736863567 0.008325 0.00674342859764 0.118 Chr3 116185509

N00035 4200000 4400000 3.093003 scaffold_1 33983693 34185852 0.00419247576772 0.00108594250119 0.00420740862517 0.00108588468624 0.413467188761 0.414393839818 0.08182 0.0832216225842 0.005175 0.0058468828991 0.074 Chr3 116185509

N00035 4400000 4600000 3.387755 scaffold_1 33780034 33983693 0.00442585140561 0.00112256520524 0.00453098001085 0.00115583584709 0.390986703989 0.394816740753 0.072305 0.0648387746184 0.02932 0.0328932185663 0.0466666666667 Chr3 116185509

N00035 4600000 4800000 3.387755 scaffold_1 33582050 33780034 0.00471571558047 0.00121859412897 0.00472490944497 0.00122108359363 0.414766653327 0.4155884946 0.08714 0.0720007677388 0.005455 0.00553074995959 0.119 Chr3 116185509

N00035 4800000 5000000 3.387755 scaffold_1 33370452 33582050 0.00434996843835 0.00101698103068 0.00455193102701 0.0010785113226 0.400085036424 0.399527995547 0.0742 0.0882049924858 0.063175 0.0634788608588 0.0755 Chr3 116185509

N00035 5000000 5200000 3.387755 scaffold_1 33172890 33370452 0.00414791774982 0.000936520565312 0.00419731383864 0.000947432233035 0.416830324265 0.4142770515 0.068665 0.0578957491825 0.01607 0.0168099128375 0.05 Chr3 116185509

N00035 5200000 5400000 3.387755 scaffold_1 32971745 33172890 0.00368716673868 0.000889934403649 0.00369437255307 0.000893962532723 0.419897702131 0.419055839294 0.080005 0.0756320067613 0.00817 0.00706952695816 0.05 Chr3 116185509

N00035 5400000 5600000 4.777258 scaffold_1 32778430 32971745 0.00440381339296 0.000916515741099 0.00443754260241 0.000924196915149 0.370247943256 0.369008741547 0.07385 0.0746708739622 0.008995 0.00935778392779 0.04 Chr3 116185509

N00035 5600000 5800000 5.040359 scaffold_1 32577024 32778430 0.00459059961033 0.00124398897664 0.00469038035448 0.00127943710832 0.404785840148 0.404988379344 0.07718 0.0717952791873 0.03638 0.0319652840531 0.0568333333333 Chr3 116185509

N00035 5800000 6000000 5.040359 scaffold_1 32377703 32577024 0.00484133230212 0.00123035097217 0.00513508005328 0.001331263925 0.396831093327 0.39429767732 0.073025 0.0485598607272 0.09428 0.0990111428299 0.061 Chr3 116185509

N00035 6000000 6200000 3.361905 scaffold_1 32182627 32377703 0.00381283986568 0.000973461402317 0.00386692692932 0.0010060386292 0.421683501684 0.423530785008 0.05422 0.0363601878242 0.04623 0.0468689126289 0.057 Chr3 116185509

N00035 6200000 6400000 1.666667 scaffold_1 31984336 32182627 0.00372672446733 0.000927673468225 0.00375642704885 0.000938353277728 0.406550766539 0.409532547966 0.07371 0.0432949553938 0.00985 0.00999036769193 0.036 Chr3 116185509

N00035 6400000 6600000 1.666667 scaffold_1 31794367 31984336 0.00383400627008 0.00124596402944 0.00383400627008 0.00124596402944 0.387309093233 0.39055908049 0.090635 0.0895988292827 0 0 NA Chr3 116185509

N00035 6600000 6800000 1.264186 scaffold_1 31593104 31794367 0.00477280012502 0.00126804966312 0.00477280012502 0.00126804966312 0.371390943735 0.369499905768 0.097875 0.112589000462 0 0 NA Chr3 116185509

N00035 6800000 7000000 0.7149171 scaffold_1 31388607 31593104 0.00446003659753 0.0013745801451 0.00446003659753 0.0013745801451 0.3583769561 0.356780199442 0.094975 0.111967412725 0 0 NA Chr3 116185509

N00035 7400000 7600000 0.7149171 scaffold_1 30812791 31004687 0.00346980155949 0.00105523040767 0.00350016107936 0.00106339331599 0.357784544444 0.357543616065 0.11298 0.0951244424063 0.008275 0.00982302914078 0.017 Chr3 116185509

N00035 7600000 7800000 0.2202442 scaffold_1 30612508 30812791 0.00329220701888 0.0010566105335 0.00331342084387 0.00106329793584 0.366580166615 0.370109833801 0.09565 0.0871217227623 0.006615 0.006610645936 0.017 Chr3 116185509

N00035 7800000 8000000 0.1625324 scaffold_1 30420195 30612508 0.00382976361459 0.00137973656738 0.00382976361459 0.00137973656738 0.357314681807 0.355806660307 0.088 0.0776962555833 0 0 NA Chr3 116185509

N00085 1000000 1200000 0.8024986 scaffold_1 40752719 40953924 0.00425285689391 0.00105359680287 0.00425285689391 0.00105680886157 0.362033810663 0.366692487347 0.13991 0.126656892224 0 0.00374742178375 0.044 Chr3 116185509

N00085 1200000 1400000 0.8024986 scaffold_1 40554758 40752719 0.00439757820904 0.00107555290262 0.00442618578237 0.00107928462956 0.352974572955 0.351191427233 0.083785 0.074590449634 0.00869 0.00868858007385 0.044 Chr3 116185509

N00085 1400000 1600000 1.853844 scaffold_1 40362951 40554758 0.00446357854305 0.00109462607837 0.00450381722687 0.00110684590833 0.369758896075 0.368268960477 0.09967 0.0950955908804 0.010425 0.0114855036573 0.044 Chr3 116185509

N00085 1600000 1800000 2.174709 scaffold_1 40167781 40362951 0.00455295232987 0.00142163811994 0.00457405297825 0.00143481389957 0.368960163148 0.369612629105 0.102595 0.0924168673464 0.008065 0.0144950555926 0.07 Chr3 116185509

N00085 1800000 2000000 2.174709 scaffold_1 39963382 40165604 0.00494805344706 0.00122031911364 0.00501822070697 0.00124858744422 0.37005679457 0.369475939648 0.078505 0.0760748088734 0.030165 0.0253829949264 0.0695 Chr3 116185509

N00085 2000000 2200000 2.174709 scaffold_1 39770610 39963382 0.00434474871081 0.00110016652906 0.00442611054006 0.00112978073285 0.377836734937 0.375777012083 0.11646 0.0804421803996 0.024165 0.0285933641815 0.055 Chr3 116185509

N00085 2400000 2600000 2.174709 scaffold_1 39382853 39576023 0.0039082789192 0.00103896863993 0.00394390980179 0.00104878397114 0.391993699062 0.389141161528 0.100135 0.0680592224465 0.010175 0.0120360304395 0.049 Chr3 116185509

N00085 2800000 3000000 0.05762082 scaffold_1 38989666 39191627 0.00455595824343 0.00122419032912 0.00461741507925 0.00124072087948 0.371218050076 0.366818352515 0.07775 0.0512574209872 0.01618 0.0171666806958 0.03 Chr3 116185509

N00085 3000000 3200000 0.1193794 scaffold_1 38800053 38989666 0.00424907128968 0.00111128557183 0.00424907128968 0.00111128557183 0.375609504842 0.379682316497 0.089355 0.0653119775543 0 0 NA Chr3 116185509

N00085 3200000 3400000 0.9442971 scaffold_1 38607078 38800053 0.00415390173122 0.00109092718511 0.00425895624783 0.00112647399686 0.383401757147 0.387452891672 0.080975 0.0590024614587 0.03664 0.0354294597746 0.067 Chr3 116185509

N00085 3400000 3600000 0.9442971 scaffold_1 38417301 38607078 0.0046414169606 0.00156486770668 0.00474910800008 0.0016081842838 0.387497812263 0.386913156509 0.084695 0.0433087255041 0.03246 0.0389509793073 0.0626666666667 Chr3 116185509

N00022 400000 600000 0.8024986 scaffold_1 42578132 42786348 0.00504885769566 0.00142608691212 0.00505209386328 0.00143344813963 0.350852644899 0.348575116955 0.120155 0.130955354055 0.00145 0.00684865716371 0.081 Chr3 116185509

N00022 600000 800000 0.8024986 scaffold_1 42786348 42982960 0.00406290438018 0.00132197169455 0.00419267264661 0.00136977973627 0.364241851125 0.361916561982 0.07785 0.0691463389824 0.044695 0.0463603442313 0.065 Chr3 116185509

N00022 1600000 1800000 0.8024986 scaffold_1 43768125 43964488 0.00501678404537 0.00130843800398 0.00501678404537 0.00130843800398 0.379905711328 0.378329277643 0.092845 0.0946104917933 0 0 NA Chr3 116185509

N00022 2000000 2200000 0.8024986 scaffold_1 44168055 44351931 0.00480403635538 0.00120274317034 0.00489631626616 0.00123153046078 0.383917785991 0.389227813543 0.08748 0.0492288281233 0.022355 0.024810198177 0.0495 Chr3 116185509

N00022 2200000 2400000 0.8024986 scaffold_1 44351931 44548545 0.00388876679072 0.00111538948556 0.00411217280707 0.00119090874724 0.377360005045 0.374989544124 0.06645 0.0398293102221 0.09667 0.0682759111762 0.053 Chr3 116185509

N00022 2400000 2600000 0.8024986 scaffold_1 44548545 44758564 0.00463702313694 0.00116211644062 0.00469503527277 0.00118056796351 0.364654652571 0.365075914655 0.08064 0.0855160723554 0.02117 0.0201696037025 0.0775 Chr3 116185509

N00022 2600000 2800000 0.8024986 scaffold_1 44758564 44946474 0.00399338763286 0.0011347714399 0.00403885722782 0.001148431768 0.365406022714 0.370208018304 0.08662 0.0538715342451 0.01391 0.0174924165824 0.074 Chr3 116185509

N00022 2800000 3000000 0.8024986 scaffold_1 44946474 45140008 0.00437501580906 0.0010859179196 0.0044272817218 0.00110491307532 0.371874981097 0.374186544542 0.13774 0.111938987465 0.01867 0.0180175059679 0.0775 Chr3 116185509

N00022 3200000 3400000 0.8024986 scaffold_1 45341004 45533268 0.00414159452852 0.00119859843116 0.00417769717409 0.00122348595175 0.396554862786 0.394784128458 0.069825 0.0588305663047 0.009805 0.0212520284609 0.05 Chr3 116185509

N00022 3400000 3600000 0.8024986 scaffold_1 45533268 45732025 0.00398708438697 0.00119569105919 0.00407687343518 0.00122502329657 0.413849025785 0.411686971843 0.11008 0.0949199273485 0.025655 0.0308064621623 0.06725 Chr3 116185509

N00022 3800000 4000000 0.8024986 scaffold_1 45891911 46088657 0.00435059157919 0.0011073894979 0.00438739813616 0.00112362807865 0.362568934965 0.359947958804 0.07413 0.0540646315554 0.01114 0.0182824555518 0.035 Chr3 116185509

N00022 4400000 4600000 0.8024986 scaffold_1 46488289 46688652 0.00445733825694 0.0011265743277 0.00445733825694 0.00112588160731 0.367120833262 0.371518330482 0.0938 0.102968112875 0 0.000733668391869 NA Chr3 116185509

N00022 6400000 6600000 1.15658 scaffold_1 48502933 48700496 0.00435243231063 0.00107953152042 0.00443601381959 0.00110653047715 0.388712642745 0.389711744691 0.07597 0.0790077089334 0.032505 0.0250249287569 0.0606666666667 Chr3 116185509

N00022 6600000 6800000 1.15658 scaffold_1 48700496 48894903 0.00380741929269 0.00108115115856 0.00385517069825 0.00109677922506 0.37149652355 0.370152154255 0.094185 0.0787574521494 0.015565 0.0158584824621 0.059 Chr3 116185509

N00022 6800000 7000000 1.15658 scaffold_1 48894903 49095104 0.00447031869719 0.00103177225268 0.00447268672929 0.00103233881169 0.368426829402 0.367913430822 0.102315 0.0849346406861 0.00045 0.0004545431841 0.059 Chr3 116185509

N00022 7400000 7600000 1.15658 scaffold_1 49478772 49689486 0.0043000866929 0.000944269627308 0.00433176906038 0.000952530798555 0.381486073229 0.386273662575 0.1343 0.11530795296 0.00903 0.00995187790085 0.062 Chr3 116185509

N00022 8400000 8600000 1.15658 scaffold_17 15532869 15751248 0.0046208718909 0.00109277042633 0.00473568264342 0.00112597585168 0.363729633204 0.362302527314 0.0734 0.123024649806 0.033755 0.0306806057359 0.094 Chr3 116185509

N00022 8600000 8800000 1.15658 scaffold_17 15329755 15532869 0.00352737386392 0.000828475511614 0.00356693171669 0.000844211620031 0.370002144155 0.370431827835 0.101585 0.0898362495938 0.017205 0.0179455872072 NA Chr3 116185509

N00022 9200000 9400000 1.15658 scaffold_17 14750422 14955329 0.00430013628609 0.00107829613132 0.00434662114897 0.0010937085901 0.371000032465 0.367174354828 0.06927 0.0756635937279 0.01498 0.0145675843187 0.0595 Chr3 116185509

N00022 9400000 9600000 1.135265 scaffold_17 14541800 14750422 0.00477872435372 0.00104975488125 0.00482518587081 0.00106471258203 0.377905031892 0.381862974468 0.096425 0.128495556557 0.01656 0.0154729606657 0.1194 Chr3 116185509

N00022 10000000 10200000 1.103025 scaffold_17 13953649 14151314 0.00442727806719 0.00118011024088 0.00442727806719 0.00118011024088 0.376172838129 0.379351987739 0.076665 0.0759820909114 0 0 NA Chr3 116185509

N00022 10200000 10400000 1.103025 scaffold_17 13765993 13953649 0.00375222048462 0.0010838849011 0.00375222048462 0.0010838849011 0.386050574766 0.388984096326 0.098765 0.0645223174319 0.00604 0 0.013 Chr3 116185509

N00022 10400000 10600000 1.094478 scaffold_17 13572603 13765993 0.00444259915869 0.00110995647189 0.00444259915869 0.00110995647189 0.388714575183 0.389834336935 0.09191 0.0753193029629 0 0 NA Chr3 116185509

N00078 200000 400000 1.024939 scaffold_17 13131539 13329214 0.00455629386576 0.000845327512649 0.00456271800661 0.000847552056258 0.381219682809 0.385554379406 0.10769 0.112153787783 0.004215 0.00444669280384 0.0605 Chr3 116185509

N00078 400000 600000 1.024939 scaffold_17 12932716 13131539 0.00419656205627 0.000976058165634 0.00419656205627 0.000976058165634 0.378370692254 0.379884189184 0.109425 0.106934308405 0 0 NA Chr3 116185509

N00078 600000 800000 1.024939 scaffold_17 12727907 12932716 0.00366825055802 0.000829084773753 0.00366825055802 0.000829084773753 0.381975988247 0.383363421437 0.10322 0.116498786674 0 0 NA Chr3 116185509

N00078 800000 1000000 1.024939 scaffold_17 12536318 12727907 0.00302730712415 0.000899862852411 0.00302730712415 0.000899862852411 0.404295992684 0.404170825802 0.073245 0.052565648341 0 0 NA Chr3 116185509

N00078 1200000 1400000 1.024939 scaffold_17 12144143 12344524 0.00409282889638 0.000899973501255 0.00416254632192 0.000917657575621 0.377833383228 0.378259916721 0.088725 0.071908015231 0.0203 0.0200567918116 0.03075 Chr3 116185509

N00078 1400000 1600000 1.024939 scaffold_17 11946237 12144143 0.0040892978288 0.00104895386277 0.00416589299972 0.00107625524518 0.421030871003 0.421938183185 0.079755 0.0695178519095 0.028845 0.0293017897386 0.062 Chr3 116185509

N00078 1800000 2000000 1.108843 scaffold_17 11570576 11763546 0.0040955791075 0.00103480621971 0.00411230650528 0.00104057068757 0.432952087585 0.438298915709 0.075365 0.0515520547235 0.00595 0.00469503031559 0.039 Chr3 116185509

N00078 2000000 2200000 1.46875 scaffold_17 11373993 11570576 0.0037673352352 0.00100743224488 0.00380025619315 0.0010153323209 0.441570623731 0.442409791589 0.092655 0.0810802561768 0.00978 0.00999577786482 0.0265 Chr3 116185509

N00078 2200000 2400000 1.46875 scaffold_17 11165714 11373993 0.00430468893591 0.000896786918842 0.00441889375981 0.000928090829615 0.40163925242 0.400757681272 0.073285 0.0891640539853 0.029195 0.0325908997066 0.04325 Chr3 116185509

N00078 2400000 2600000 1.46875 scaffold_17 10960665 11165714 0.00428178948778 0.00104245708708 0.00432495765412 0.00105438645988 0.402075212737 0.399278486385 0.09537 0.094455471619 0.009425 0.013704041473 0.035 Chr3 116185509

N00078 2600000 2800000 1.46875 scaffold_17 10760270 10960665 0.00401479846793 0.000982003810693 0.00413399005955 0.00102182459287 0.371792664026 0.369196366014 0.10395 0.106439781432 0.040085 0.0399211557175 0.04 Chr3 116185509

N00078 2800000 3000000 1.612825 scaffold_17 10554253 10760270 0.00383490208603 0.000883533572265 0.0038826390908 0.000896970097938 0.398002706914 0.399235499598 0.054925 0.0799011732042 0.018945 0.0176975686472 0.064 Chr3 116185509

N00078 3000000 3200000 1.672687 scaffold_17 10363997 10554253 0.00416562833776 0.000866691638757 0.00416562833776 0.000866691638757 0.376367262716 0.37518117839 0.086765 0.0855531494408 0 0 NA Chr3 116185509

N00078 3200000 3400000 1.672687 scaffold_17 10170210 10363997 0.00364257573365 0.00099000498158 0.00365111230876 0.000993505919045 0.391975274079 0.39834336939 0.07733 0.062191994303 0.005145 0.00532543462668 0.031 Chr3 116185509

N00163 400000 600000 1.672687 scaffold_17 8472991 8664361 0.00417351458299 0.000877958826814 0.00417351458299 0.000877958826814 0.375363812414 0.378171225404 0.086445 0.0714166274756 0 0 NA Chr3 116185509

N00163 600000 800000 1.672687 scaffold_17 8664361 8857667 0.00400890640138 0.000868253312042 0.00402157563782 0.000870970808991 0.381897652769 0.383248688329 0.101795 0.0660300249346 0.00696 0.00440751968382 0.074 Chr3 116185509

N00163 800000 1000000 1.672687 scaffold_17 8857667 9047532 0.00450643687606 0.00097575266791 0.00455040277301 0.000987997123228 0.373515367075 0.376422180649 0.09948 0.0759961024939 0.011095 0.0124667526927 0.067 Chr3 116185509

N00163 1000000 1200000 1.672687 scaffold_17 9047532 9242749 0.00452155437274 0.00107681044979 0.00460965661625 0.00111720938547 0.359124365404 0.358358045887 0.128735 0.129676206478 0.03316 0.0358677779087 0.075 Chr3 116185509

N00163 1200000 1400000 1.672687 scaffold_17 9242749 9457038 0.00482527536931 0.000878411078839 0.00482527536931 0.000878411078839 0.36223723506 0.3628243325 0.121705 0.164595476203 0 0 NA Chr3 116185509

N00048 200000 400000 2.426288 scaffold_17 1942345 2132985 0.00358666234619 0.000801040740717 0.00359546797013 0.000802968170265 0.420228310502 0.425005538734 0.11567 0.0756609315988 0.004055 0.00532941670164 0.011 Chr3 116185509

N00048 400000 600000 2.426288 scaffold_17 2133559 2323093 0.00398177466667 0.00118959771954 0.00401609015222 0.00120313687683 0.405871607671 0.412090924305 0.11274 0.0839532748742 0.020625 0.0245655133116 0.098 Chr3 116185509

N00048 800000 1000000 2.426288 scaffold_17 2518848 2708580 0.0041638749611 0.00119075995977 0.00419489863457 0.00120204253279 0.443191819287 0.449460510299 0.135295 0.0942329180107 0.018705 0.0169818480805 0.106 Chr3 116185509

N00048 1000000 1200000 2.426288 scaffold_17 2708580 2894796 0.00468268280764 0.0014175074022 0.00476986474872 0.00145366490891 0.393859075836 0.400093257704 0.11268 0.0799018344288 0.030515 0.0328006186364 0.097 Chr3 116185509

N00048 1200000 1400000 19.51389 scaffold_17 2894796 3085673 0.00467130785855 0.00120053232971 0.00467130785855 0.00120222283498 0.401255009308 0.408163619935 0.13356 0.0855210423466 0.003 0.003143385531 0.045 Chr3 116185509

N00048 1600000 1800000 1.019328 scaffold_17 3270722 3465279 0.00382256887324 0.00113337868238 0.00382688596019 0.00113855985895 0.432677515991 0.43498943806 0.10349 0.100741684956 0.00411 0.00694912030922 NA Chr3 116185509

N00048 1800000 2000000 1.019328 scaffold_17 3466074 3666408 0.00372589488228 0.00119098545843 0.00384798044953 0.00125233269027 0.411294120488 0.409530288689 0.106085 0.109467189793 0.04791 0.0550630447153 0.055 Chr3 116185509

N00048 2000000 2200000 1.019328 scaffold_17 3666408 3861598 0.00347246189965 0.00110807044462 0.00356850931999 0.00114372001626 0.393195029536 0.395564664702 0.09031 0.0603258363646 0.041055 0.0461857677135 0.067 Chr3 116185509

N00048 2200000 2400000 1.019328 scaffold_17 3861598 4050375 0.00300099268151 0.00078936260787 0.00305116666265 0.000805988970323 0.403167438865 0.403068248869 0.054675 0.0474740037187 0.02126 0.0229953860905 0.044 Chr3 116185509

N00048 2400000 2600000 1.019328 scaffold_17 4050375 4242359 0.00337300883299 0.000917290097875 0.00337300883299 0.00091881848619 0.409297993056 0.412558505386 0.103625 0.0679171180932 0 0.00304712892741 NA Chr3 116185509

N00048 2600000 2800000 1.313908 scaffold_17 4242359 4435084 0.0034417103518 0.000799571834471 0.0034417103518 0.000799571834471 0.400633261817 0.402053982583 0.12255 0.0896562459463 0 0 NA Chr3 116185509

N00048 2800000 3000000 1.334278 scaffold_17 4435084 4631454 0.00364347116037 0.000874875156671 0.00364347116037 0.000874875156671 0.381192550005 0.382972721374 0.113415 0.110317258237 0 0 NA Chr3 116185509

N00048 3000000 3200000 1.334278 scaffold_17 4631454 4823379 0.00419967466246 0.00100496007773 0.00419967466246 0.00100496007773 0.366966288146 0.368915654295 0.13481 0.119494594243 0 0 NA Chr3 116185509

N00048 3400000 3600000 2.488127 scaffold_17 5006848 5222007 0.00390132799642 0.000778961702988 0.00390132799642 0.000778961702988 0.365616176596 0.365664897268 0.12583 0.150195901636 0 0 NA Chr3 116185509

N00048 3600000 3800000 1.213116 scaffold_17 5222007 5429341 0.00420130710416 0.000706813072893 0.00420130710416 0.000706813072893 0.369459091144 0.370280788431 0.12753 0.148234250051 0 0 NA Chr3 116185509

N00048 3800000 4000000 0 scaffold_17 5429341 5621936 0.00361756995028 0.000809490294299 0.00364553095681 0.000826986448743 0.374682469914 0.379184232958 0.115085 0.109016329604 0.01641 0.0305459643293 0.104 Chr3 116185509

N00048 4000000 4200000 0 scaffold_17 5621936 5814852 0.00368941114782 0.000859690419066 0.0038526591808 0.00092172554672 0.375352225326 0.378360762588 0.10868 0.0855087188206 0.061425 0.0719535963839 0.108 Chr3 116185509

N00048 4400000 4600000 1.36693 scaffold_17 6001008 6188472 0.00366525706544 0.00106724304672 0.00367243099747 0.0010701532693 0.439508858462 0.445243294697 0.104375 0.0801273844578 0.002565 0.002757862843 0.097 Chr3 116185509

N00048 4800000 5000000 1.36693 scaffold_17 6378458 6567932 0.00403128684992 0.00110829086379 0.00407338316895 0.00112449341106 0.420730624948 0.433479815114 0.10818 0.0720626576733 0.01337 0.0194749675417 0.0913333333333 Chr3 116185509

N00048 5000000 5200000 1.36693 scaffold_17 6567932 6759491 0.00384627024608 0.000911898744249 0.00390688862537 0.000926934473417 0.406715066651 0.412185479278 0.078225 0.0652279454372 0.026355 0.0233296269035 0.0806666666667 Chr3 116185509

N00048 5200000 5400000 1.36693 scaffold_17 6759492 6955427 0.00422256011741 0.000830915169754 0.00422571270949 0.000832057377604 0.389318885449 0.391683437556 0.069035 0.050465715671 0.003895 0.0039962232373 NA Chr3 116185509

N00048 5400000 5600000 1.36693 scaffold_17 6955427 7144413 0.00381698848836 0.000917860485057 0.00381698848836 0.000917860485057 0.374651968862 0.375845448844 0.11499 0.0687669986137 0.00234 0 NA Chr3 116185509

N00048 5600000 5800000 1.108157 scaffold_17 7144413 7344536 0.00371058388443 0.000899821916181 0.00371058388443 0.000899821916181 0.378969365574 0.377246883884 0.085385 0.104300854974 0 0 NA Chr3 116185509

N00048 5800000 6000000 0.7326203 scaffold_17 7344536 7551057 0.00415214761026 0.000878502638618 0.00416833653697 0.000883368377185 0.383966356467 0.3873945585 0.07907 0.0708741483917 0.00814 0.00796044954266 0.0675 Chr3 116185509

N00048 6000000 6200000 0.7326203 scaffold_17 7551057 7746055 0.00459909183782 0.00129197422541 0.00464242557266 0.00131481632203 0.38310005184 0.390731967176 0.093895 0.0911547810747 0.023475 0.023277161817 0.149333333333 Chr3 116185509

N00048 6200000 6400000 0.7326203 scaffold_17 7746055 7950469 0.0039204661082 0.00106692342676 0.00401202106979 0.00111414093942 0.377771352024 0.382218674236 0.10171 0.102400031309 0.0483 0.0534063224632 0.0803333333333 Chr3 116185509

N00042 0 200000 3.247232 scaffold_41 7773567 7985902 0.00134598044268 0.000297222763584 0.00139342898703 0.000310314584768 0.433466889996 0.440582422347 0.08377 0.0281300774719 0.039865 0.0410247957237 0.0864 Chr4A 21340098

N00042 1400000 1600000 4.253275 scaffold_41 6381145 6584992 0.00383205129155 0.00130707846862 0.00383205129155 0.00130707846862 0.395371673264 0.405784376044 0.07181 0.0294191231659 0 0 NA Chr4A 21340098

N00042 1600000 1800000 2.484125 scaffold_41 6163691 6380935 0.00386401515424 0.00157775828186 0.00390747961901 0.00159452342814 0.392531758039 0.395586118663 0.04633 0.0260812726704 0.015865 0.0154802894441 0.267 Chr4A 21340098

N00042 2000000 2200000 7.570997 scaffold_41 5717094 5925025 0.0040396608162 0.00146807100632 0.0040396608162 0.00146807100632 0.388680092835 0.395682718511 0.069775 0.0353819295824 0 0 NA Chr4A 21340098

N00042 2200000 2400000 5.515744 scaffold_41 5514238 5717094 0.00376206122854 0.00138161773912 0.00376206122854 0.00138261039336 0.391393846823 0.395108725123 0.07794 0.0233909768506 0 0.00193240525299 0.066 Chr4A 21340098

N00042 2400000 2600000 2.876611 scaffold_41 5318114 5514238 0.00378942897169 0.00133785901699 0.00378942897169 0.00133962092583 0.401848136023 0.407239989859 0.07288 0.0330046297241 0 0.00138177887459 0.066 Chr4A 21340098

N00042 2600000 2800000 2.876611 scaffold_41 5115002 5318114 0.00381769319328 0.00118137119864 0.00383366604064 0.00119164185958 0.385344635866 0.386558811232 0.05175 0.0271623537753 0.015135 0.0151689708141 0.066 Chr4A 21340098

N00042 2800000 3000000 0.801444 scaffold_41 4903353 5115002 0.00445303974792 0.00196173083282 0.00445303974792 0.00196173083282 0.385308463999 0.394560222293 0.063765 0.0429815401915 0 0 NA Chr4A 21340098

N00042 3000000 3200000 0.1034278 scaffold_41 4688323 4903353 0.00401584447975 0.00145976592195 0.00401584447975 0.00145976592195 0.38856073793 0.391796255408 0.051025 0.0355252755429 0 0 NA Chr4A 21340098

N00042 3200000 3400000 1.889908 scaffold_41 4479067 4688323 0.0041761790596 0.00149067066412 0.0041761790596 0.00149067066412 0.387697233709 0.389343531738 0.074255 0.0477883549337 0 0 NA Chr4A 21340098

N00042 3400000 3600000 2.956897 scaffold_41 4285646 4479067 0.00412470347613 0.0016315359431 0.00412470347613 0.0016315359431 0.373787067489 0.380191040225 0.092755 0.0462824615735 0 0 NA Chr4A 21340098

N00042 3600000 3800000 3.872198 scaffold_41 4080766 4285646 0.00438159793361 0.00159010092437 0.00440400910037 0.00160253288428 0.383296861752 0.385521657332 0.065575 0.0280261616556 0.010785 0.0106452557595 0.202 Chr4A 21340098

N00042 3800000 4000000 2.937984 scaffold_41 3887543 4080766 0.00395889480545 0.0012416771034 0.00396929297613 0.00124798595138 0.397145354539 0.397510494497 0.068045 0.0225853029919 0.00573 0.00631394813247 0.094 Chr4A 21340098

N00042 4000000 4200000 6.533114 scaffold_41 3690686 3887543 0.00377135709081 0.00126299212798 0.00378477824157 0.00126318489578 0.396410834976 0.403643729001 0.052485 0.0203751962084 0.005965 0.00786357609839 0.094 Chr4A 21340098

N00042 4200000 4400000 5.901331 scaffold_41 3498307 3690686 0.00400638064125 0.00125583936811 0.00410210647665 0.00129247277458 0.396215735883 0.410390974949 0.055075 0.033595142921 0.041195 0.0409400194408 0.1 Chr4A 21340098

N00042 4400000 4600000 5.223381 scaffold_41 3306913 3498307 0.00415349925307 0.00140625836694 0.00419005012684 0.00142302527281 0.41352599932 0.415852408609 0.10568 0.0351891908837 0.014845 0.0154080065206 0.1285 Chr4A 21340098

N00042 4600000 4800000 8.544735 scaffold_41 3121046 3306913 0.00347669722937 0.00110520429507 0.0035207622611 0.0011201483963 0.428762686497 0.431750158203 0.109645 0.0398725970721 0.010705 0.0120731490797 0.026 Chr4A 21340098

N00042 4800000 5000000 5.697095 scaffold_41 2934676 3121046 0.00391981792243 0.00108507566409 0.00394090438009 0.00109383570957 0.431135628911 0.434042930637 0.12925 0.0693727531255 0.01978 0.0235982185974 0.103 Chr4A 21340098

N00042 6600000 6800000 3.627802 scaffold_41 1314819 1506720 0.00418734683671 0.000879568036618 0.00425954499555 0.000916449631545 0.463795724837 0.46904892927 0.086035 0.0284938588126 0.050625 0.0684102740476 0.075 Chr4A 21340098

N00042 7000000 7200000 3.240803 scaffold_41 938788 1126285 0.00351214201499 0.000774462813122 0.00363185024426 0.000806163226595 0.435896838389 0.440332364786 0.0758 0.0228856995045 0.04113 0.0412166594666 0.0443333333333 Chr4A 21340098

N00079 200000 400000 0.3911587 scaffold_61 2950055 3145760 0.0031464309587 0.00100431082912 0.0031464309587 0.00100431082912 0.364580014422 0.362230052222 0.08754 0.0686543522138 0 0 NA Chr4A 21340098

N00079 400000 600000 0.3911587 scaffold_61 2760303 2950055 0.00310661760983 0.00104687230785 0.00312748883566 0.00105517356626 0.378735583803 0.374195218221 0.060535 0.0462709220456 0.012215 0.0136704751465 0.067 Chr4A 21340098

N00079 600000 800000 0.3911587 scaffold_61 2564786 2760303 0.00272325021205 0.000961221203125 0.0028129613823 0.00100220659876 0.400176779197 0.39648809994 0.064935 0.0498524424986 0.048875 0.0497194617348 0.0555 Chr4A 21340098

N00079 800000 1000000 0.3911587 scaffold_61 2369981 2564786 0.00273021184842 0.000889544798572 0.00282934901128 0.000928634656385 0.395044415953 0.397081470371 0.08527 0.039896306563 0.04153 0.0427863761197 0.063 Chr4A 21340098

N00079 1200000 1400000 1.373083 scaffold_61 1988724 2201293 0.00332479935757 0.000929824667823 0.00332479935757 0.000927491408238 0.39079920837 0.389206257113 0.103065 0.10040504495 0 0.00508070320696 NA Chr4A 21340098

N00079 1400000 1600000 1.373083 scaffold_61 1787960 1988724 0.00350628852001 0.000796513948536 0.00350628852001 0.000796513948536 0.401981101686 0.396400474784 0.08977 0.071387300512 0 0 NA Chr4A 21340098

N00079 1800000 2000000 4.056509 scaffold_61 1423096 1604503 0.00337985607895 0.000354277985517 0.00349306332997 0.00037536312725 0.409300166351 0.415635704448 0.09059 0.0292546594123 0.05998 0.0667339187573 0.0524444444444 Chr4A 21340098

N00079 2000000 2200000 0.786087 scaffold_61 1217053 1423096 0.00373682421734 0.000529761626526 0.003762826686 0.000533829582691 0.410114487633 0.411742854767 0.091215 0.111030221847 0.006945 0.00751784821615 0.049 Chr4A 21340098

N00079 2200000 2400000 0.786087 scaffold_61 1027825 1217053 0.00394879799481 0.000410707880679 0.00395235278174 0.000412456384046 0.407759173911 0.40997311798 0.0951 0.0909062083835 0.001785 0.00578667004883 NA Chr4A 21340098

N00079 2400000 2600000 1.113727 scaffold_61 827615 1027825 0.00386930763512 0.000455863290124 0.00388396546325 0.000459323139391 0.412731305166 0.421764484882 0.072005 0.0929074471805 0.01017 0.00984965785925 0.058 Chr4A 21340098

N00079 3400000 3600000 1.845092 scaffold_41 131347 346995 0.00306436022213 0.000458103605791 0.00308470268366 0.000461879131338 0.456206854208 0.465460350682 0.054465 0.080742691794 0.009065 0.00970563139932 0.011 Chr4A 21340098

N00079 3600000 3800000 1.845092 scaffold_41 346995 547584 0.00352577835549 0.000522472626623 0.00353544742496 0.000525575021937 0.44041060622 0.449765812442 0.074835 0.0678451958981 0.009935 0.00677006216692 NA Chr4A 21340098

N00161 400000 600000 0.3911587 scaffold_61 4146860 4342703 0.000706152069614 0.000914741408064 0.000724675256466 0.000943146963759 0.38949981622 0.38248382112 0.10509 0.0989976664982 0.03261 0.0333685656368 0.0715 Chr4A 21340098

N00161 1000000 1200000 0.3911587 scaffold_61 3614592 3798596 0.00265538131942 0.00119161186995 0.00265538131942 0.00119161186995 0.401434464436 0.396418531554 0.09629 0.0527814612726 0 0 NA Chr4A 21340098

N00161 1200000 1400000 0.3911587 scaffold_61 3429830 3614592 0.00259779980767 0.00100203413812 0.00259779980767 0.00100203413812 0.38823722707 0.378754633746 0.13107 0.0631569262078 0 0 NA Chr4A 21340098

N00047 800000 1000000 3.777834 scaffold_44 2215033 2408010 0.00386689894833 0.00122716824041 0.00387934390259 0.00123238335214 0.418881749829 0.417439095735 0.062505 0.0361079299606 0.00594 0.00576752670007 0.051 Chr4A 21340098

N00047 1000000 1200000 3.019188 scaffold_44 2408010 2602382 0.00390112555569 0.00113341346255 0.00411674194191 0.00120906551638 0.417631513907 0.419682998855 0.08405 0.0350153314263 0.080405 0.0819253801988 0.0858333333333 Chr4A 21340098

N00047 2600000 2800000 5.894939 scaffold_44 3811419 4007349 0.00353506294014 0.00119195290233 0.00378171101065 0.00129800960287 0.466794535213 0.468896001177 0.077465 0.0417751237687 0.107215 0.11501046292 0.0621111111111 Chr4A 21340098

N00047 3200000 3400000 2.153153 scaffold_44 4372725 4561029 0.00349361937305 0.00132753720877 0.00349361937305 0.00132839506091 0.420554177704 0.421351158057 0.088775 0.0493988444218 0.002355 0.00321820035687 0.059 Chr4A 21340098

N00047 3400000 3600000 2.458579 scaffold_44 4561029 4749273 0.00338899381349 0.00124025470936 0.00343473466484 0.00126090174985 0.417030924811 0.415428163131 0.069525 0.0335097001764 0.020445 0.0246222987187 0.0943333333333 Chr4A 21340098

N00047 3800000 4000000 1.11623 scaffold_44 4925524 5107173 0.00402228883036 0.00132651340356 0.00402228883036 0.00132651340356 0.381539661384 0.379192619277 0.11225 0.0530748861816 0 0 NA Chr4A 21340098

N00047 4000000 4200000 1.11623 scaffold_44 5107173 5291737 0.00385107256181 0.00128664893967 0.00385107256181 0.00128664893967 0.383866210949 0.381525410503 0.09865 0.0520253137123 0 0 NA Chr4A 21340098

N00047 4200000 4400000 1.11623 scaffold_44 5291737 5478163 0.00374742854236 0.00145764964883 0.00378545232732 0.00147026659606 0.380684286434 0.382815490356 0.086075 0.0453209316297 0.012555 0.0134852434746 0.04 Chr4A 21340098

N00047 4600000 4800000 0.7725459 scaffold_44 5647003 5840951 0.00320803448655 0.00127327954333 0.00322639344873 0.00128417745194 0.382953462221 0.38170264284 0.08901 0.0734629900798 0.014485 0.0132561305092 0.041 Chr4A 21340098

N00047 5200000 5400000 0.7725459 scaffold_44 6201201 6390840 0.00253266673549 0.00100278182873 0.00254581504938 0.0010099738625 0.396626952226 0.390288365926 0.086905 0.0310537389461 0.00796 0.00891694219016 0.036 Chr4A 21340098

N00047 5400000 5600000 0.7725459 scaffold_44 6390840 6582766 0.00231194216691 0.000983938713174 0.00232402164372 0.000989349103921 0.398708951626 0.391305040464 0.053135 0.0209612037973 0.00526 0.00642435105197 0.071 Chr4A 21340098

N00047 5600000 5800000 0.4266298 scaffold_44 6582766 6775500 0.00234185051043 0.00101482949439 0.00236177464688 0.00102438699546 0.411972790599 0.403188456952 0.05222 0.0329936596553 0.012845 0.0136613155956 0.07 Chr4A 21340098

N00047 5800000 6000000 0 scaffold_44 6775500 6967811 0.00180109397129 0.00103423343168 0.00181855012906 0.00104791307004 0.392598266433 0.385720853411 0.1039 0.0455928158036 0.015125 0.0153969351727 0.0573333333333 Chr4A 21340098

N00047 6000000 6200000 0.0797886 scaffold_44 6967823 7162135 0.000979357236179 0.000981989592126 0.000998793060512 0.00100314420558 0.40887399001 0.400808799562 0.06109 0.025911935444 0.033865 0.0336263329079 0.0533333333333 Chr4A 21340098

N00190 0 200000 0 scaffold_109 622426 819626 0.00277736463336 0.00120722528436 0.0028141120735 0.00123453090037 0.448371470628 0.458049766057 0.03058 0.0151369168357 0.024365 0.0256338742394 0.1288 Chr4 72322651

N00190 600000 800000 0 scaffold_86 467914 665135 0.00361798534282 0.00159861222723 0.0037085102617 0.00167175507261 0.45288103398 0.466876792135 0.06316 0.0260063583493 0.056945 0.0644911038885 0.162166666667 Chr4 72322651
[truncated: 336,640 more chars]
